# Mapping the sevoflurane-binding sites of calmodulin

**DOI:** 10.1002/prp2.25

**Published:** 2014-02-12

**Authors:** Ulrika Brath, Kelvin Lau, Filip Van Petegem, Máté Erdélyi

**Affiliations:** 1 Department of Chemistry and Molecular Biology and the Swedish NMR Centre, University of GothenburgSE-412 96, Gothenburg, Sweden; 2 Department of Biochemistry and Molecular Biology, University of British ColumbiaVancouver, British Columbia, V6T 1Z3, Canada

**Keywords:** Anesthesia, anesthetic binding, calmodulin, ITC, NMR, sevoflurane

## Abstract

General anesthetics, with sevoflurane (SF) being the first choice inhalational anesthetic agent, provide reversible, broad depressor effects on the nervous system yet have a narrow margin of safety. As characterization of low-affinity binding interactions of volatile substances is exceptionally challenging with the existing methods, none of the numerous cellular targets proposed as chief protagonists in anesthesia could yet be confirmed. The recognition that most critical functions modulated by volatile anesthetics are under the control of intracellular Ca^2+^ concentration, which in turn is primarily regulated by calmodulin (CaM), motivated us for characterization of the SF–CaM interaction. Solution NMR (Nuclear Magnetic Resonance) spectroscopy was used to identify SF-binding sites using chemical shift displacement, NOESY and heteronuclear Overhauser enhancement spectroscopy (HOESY) experiments. Binding affinities were measured using ITC (isothermal titration calorimetry). SF binds to both lobes of (Ca^2+^)_4_-CaM with low mmol/L affinity whereas no interaction was observed in the absence of Ca^2+^. SF does not affect the calcium binding of CaM. The structurally closely related SF and isoflurane are shown to bind to the same clefts. The SF-binding clefts overlap with the binding sites of physiologically relevant ion channels and bioactive small molecules, but the binding affinity suggests it could only interfere with very weak CaM targets.

## Introduction

**Figure 1:**
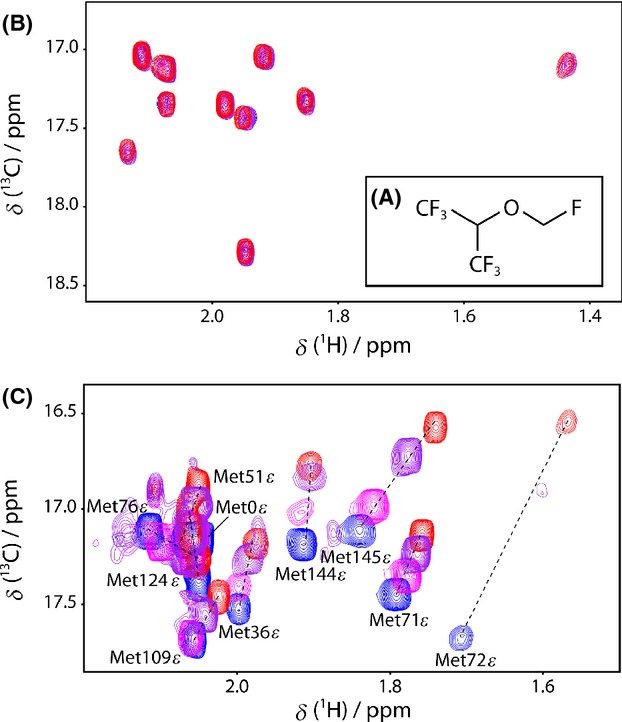
Representative sections of the ^1^H,^13^C-HSQC spectra of titrations of CaM with SF. Sevoflurane (SF, A) titrations to methionine methyl region of *apo*-CaM (B) and (Ca^2+^)_4_-CaM (C) in blue, overlaid with spectra taken after successive additions of 2.4 (magenta), 5.0 (purple), and 10 (red) mmol/L SF.

Hundreds of millions of patients are anesthetized yearly despite the surprisingly little understanding of the molecular mechanisms of anesthetic action (Arhem et al. 2003; Orser 2007). The high volatility, low aqueous solubility, and low binding affinity of general anesthetics make their experimental investigation cumbersome. Accordingly, neither the affected crucial brain structures and cellular processes nor the molecular basis of their bioactivity have yet been identified. (Arhem et al. 2003). Following a long detour into the lipid bilayer, (Meyer 1899; Franks and Lieb 1978) regulation of neurotransmission by selective binding to proteins that regulate ion homeostasis has become the widely accepted explanation for anesthetic action (Franks and Lieb 1984). Numerous soluble and membrane proteins involved in neurotransmission were pointed out as possible targets, typical examples being the presynaptic voltage-gated Na^+^ channels (Na_v_), the postsynaptic GABA and nACh receptors, and intracellular regulatory proteins such as calmodulin (CaM). Despite being the general anesthetic agent of choice in modern anesthesiology (Michel and Constantin 2009), no structure for a sevoflurane (SF, Fig. 1A)–protein complex has yet been reported.

CaM is the principal intracellular receptor for Ca^2+^ and its vast importance as a signaling protein is attributable to its ability to undergo interconversion between the *apo*- and partially or fully Ca^2+^-loaded states in electrically or chemically stimulated cells. Considering that Ca^2+^ is recognized as a foremost mediator of rapid cellular responses to electrical stimuli, (Rasmussen 1986; Brini et al. 2008) its role in the regulation of consciousness, pain, skeletal muscle relaxation, and alveolar ventilation in relation to a possibly CaM-mediated route of anesthesia was raised based on numerous arguments (Landers et al. 1989; Kress and Tas 1993): (1) CaM regulates neurotransmitter release in all types of neurons by binding to P/Q- and N-type voltage-gated Ca^2+^ channels, and (2) it increases neuronal excitability by binding to Na_v_ channels. (3) The pathogenesis of anesthesia related disorders, such as malignant hyperthermia, originates from volatile anesthetics-induced imbalance of intracellular Ca^2+^ homeostasis. (4) Through modulation of the function of a large assembly of regulatory proteins and second messenger systems, CaM is simultaneously a direct and indirect regulator of cellular responses, (Chin and Means 2000; Yamniuk and Vogel 2004) and, therefore, even a slight detuning of its function has major consequences. The hypothesis that volatile anesthetics act directly on CaM, either (1) by altering its Ca^2+^-binding affinity or (2) by affecting its regulatory effect on ion channels, secondary messengers or regulatory proteins has been supported by observations of CaM's ability to bind anesthetics, such as halothane and isoflurane (Levin and Blanck 1995; Streiff et al. 2004). Despite intense efforts, neither the structure of a CaM–anesthetic complex nor the localization of an anesthetic-binding site on the protein surface has yet been disclosed. Our present understanding of the interaction of CaM with anesthetics is based on indirect sources: theoretical predictions, (Streiff et al. 2006) information derived from related systems in which anesthetics are complexed with proteins (Zhang and Johansson 2003; Bertaccini et al. 2007) or CaM with drugs that are not directly related to anesthetic action, (Vandonselaar et al. 1994; Ohashi et al. 2004) and saturation transfer NMR experiments on halothane–CaM mixtures (Streiff et al. 2004). On the basis of the latter, Streiff et al. (2004) proposed low mmol/L halothane affinity, but originating from the inherent experimental limitations, the structure of the interacting complex was not disclosed.

Triggered by the recognition of the regulatory role of CaM and by the fact that its regulatory function may be modulated by anesthetics (Landers et al. 1989; Kress and Tas 1993; Streiff et al. 2006), the aim of this study is characterization of the CaM-binding site(s) for SF.

## Materials and Methods

### Protein expression

U-[^13^C,^15^N] labeled CaM was recombinantly expressed using a modified pET28 vector containing an N-terminal hexahistidine tag, maltose-binding protein and a tobacco etch virus (TEV) protease cleavage site to create a HMT (Hexahistidine-Maltose binding protein-Tev protease cleavege site)-CaM construct. Expression was done in *Escherichia coli* Rosetta (DE3) plasmid that encodes the lac repressor protein (pLacI) at 37°C, induced at OD_600_ ∼ 0.6 by addition of 0.2 mmol/L isopropyl-*β*-d-1-thiogalactopyranoside (IPTG) for 4 h using minimal media with ^13^C-glucose and ^15^NH_4_Cl (Sigma-Aldrich, St. Louis, MO) as the sole carbon and nitrogen sources. The CaM construct contained an additional four residues (Ser-Asn-Ala-Met) prior to the native human sequence, but impeccable ^1^H,^15^N-HSQC (heteronuclear single quantum correlation) spectral overlap with previously published data sets for native *apo*- and (Ca^2+^)_4_-CaM indicated that this sequence addition does not alter the protein structural properties (Tjandra et al. 1995; Kainosho et al. 2006). In order to comply with previous nomenclature, we use the conventional sequence numbering, thus renumbering the first four residues Ser-3 to Met-0.

### Protein purification

Cells were lysed by sonication in buffer A (250 mmol/L KCl, 10 mmol/L CaCl_2_ and 10 mmol/L HEPES (2-[4-(2-hydroxyethyl)piperazin-1-yl]ethanesulfonic acid), pH 7.4) with 25 mg mL^−1^ DNase I, 25 mg mL^−1^ lysozyme, 1 mmol/L iodoacetamide, and 1 mmol/L phenylmethylsulphonyl fluoride. The lysate was applied to a 25 mL Poros MC column (Tosoh Bioscience, King of Prussia, PA), washed with five column volumes of buffer A and five column volumes of buffer A plus 2% (v/v) buffer B (250 mmol/L KCl, 10 mmol/L CaCl_2_ and 500 mmol/L imidazole, pH 7.4) and eluted with 30% (v/v) buffer B. The protein was then cleaved with his-tagged TEV protease overnight at room temperature while in dialysis against buffer A. The tagged protease and cleavage product were removed with an additional Poros MC column. The flow through was applied to a 25 mL amylose column (New England Biolabs, Ipswich, MA). The protein was then washed with five column volumes of buffer A to remove additional cleaved tag products. The flow through contained the protein and was applied to a Phenyl-Sepharose HP column (GE Healthcare Bio-Sciences AB, Uppsala, Sweden) equilibrated with 150 mmol/L KCl, 20 mmol/L HEPES, pH 7.4, 10 mmol/L CaCl_2_. The protein was eluted with the same buffer containing 10 mmol/L EDTA (ethylenediaminetetraacetic acid) instead of CaCl_2_. The protein was applied to a HiLoad Q-Sepharose HP column equilibrated with 20 mmol/L HEPES pH 7.4, 10 mmol/L EDTA, and eluted with a gradient of 20% to 40% of buffer containing an additional 1 mol/L of KCl over 14 CV. The molecular weight for CaM was confirmed by MALDI-TOF on a Voyager-DE STR (Applied Biosystems, Foster City, CA). CaM was dialyzed into 111 mmol/L KCl, 16.5 mmol/L CaCl_2_ and 0.11 mmol/L NaN_3_, pH 6.3. The CaM concentration was determined using the calculated extinction coefficient, *ε* = 2980 L mol^−1^ cm^−1^, at 280 nm in the presence of 6 mol/L Guanidine (Edelhoch 1967). CaM in its *apo* state was prepared by dialysis against Ca^2+^-free buffer, and subsequent addition of EDTA to achieve completely Ca^2+^-depleted CaM. The individual CaM lobes were grown as HT-construct and purified with the same protocol, with the exception that the amylose column was not used.

### NMR

All CaM NMR samples were prepared with 100 mmol/L KCl, 0.2 mmol/L NaN_3_, 0.1 mmol/L 2,2-dimethyl-2-silapentane-5-sulfonic acid (DSS) at pH 6.3. The *apo*-CaM sample contained 0.18 mmol/L U–[^13^C,^15^N] CaM and 1.18 mmol/L EDTA in 90%/10% v/v H_2_O/D_2_O, and the (Ca^2+^)_4_-CaM sample used for titrations was obtained by the addition of 14.2 mmol/L CaCl_2_. The sample used for assignment contained 2.3 mmol/L U–[^13^C,^15^N] CaM and 15 mmol/L CaCl_2_ in 90%/10% v/v H_2_O/D_2_O. The sample used for the ^1^H,^1^H-NOESY, ^19^F,^1^H-HOESY (heteronuclear Overhauser enhancement spectroscopy), and 3D-^1^H,^13^C,^1^H-HSQC (heteronuclear single quantum correlation)–NOESY experiments contained 4 mmol/L U–[^13^C,^15^N] CaM, 15 mmol/L CaCl_2_ and 2 mmol/L SF in 99.9% D_2_O.

All NMR experiments were run at 25°C. The NMR assignment was made using HNCA (Kay et al. 1990), HNCO (Kay et al. 2011) HNCACO (Yamazaki et al. 1994) HNCOCA (Yamazaki et al. 1994), and HCCH–TOCSY (Baldisseri 1991), 2D-^1^H,^1^H-NOESY (Jeener et al. 1979; Kumar et al. 1980) on a Varian Inova 800 MHz spectrometer equipped with a 5 mm triple resonance probe. Methionine methyl assignments were made based on published assignments (Siivari et al. 1995), 3D-^1^H,^13^C,^1^H-HSQC–NOESY, (Fesik and Zuiderweg 1988), 2D-^19^F,^1^H-HOESY (Bauer 1996), and titration experiments (^1^H,^15^N-HSQC and ^1^H,^13^C-CT-HSQC) were run on a Varian VNMR–S 500 MHz spectrometer equipped with a ^1^H–^19^F/^15^N–^31^P 5 mm pulsed field gradient capable dual broadband probe. ^1^H,^15^N-HSQC spectra were acquired with 128 × 1024 complex points (*F*_1_ × *F*_2_) and spectral widths of 1914 and 13008 Hz, respectively. ^1^H,^13^C-HSQC spectra were acquired with 122 × 1024 complex points (*F*_1_ × *F*_2_) and spectral widths of 4425 and 13008 Hz, respectively. ^19^F,^1^H-HOESY spectra were acquired with 64 × 2048 complex points (*F*_1_ × *F*_2_) and spectral widths of 4099 and 108696 Hz, respectively. Mixing times of 100, 250, 400, and 500 msec were evaluated. ^1^H,^1^H-NOESY spectra were acquired with 300 × 2400 complex points (*F*_1_ × *F*_2_), spectral widths of 8000 Hz and a mixing time of 900 msec. Full methyl region 3D-^1^H,^13^C,^1^H-HSQC–NOESY spectra were acquired with 50 × 20 × 512 complex points (*F*_1_ × *F*_2_ × *F*_3_) and spectral widths of 5787, 2187, and 5787 Hz, respectively. Methionine methyl region 3D-^1^H,^13^C,^1^H-HSQC–NOESY spectra were acquired with 70 × 14 × 512 complex points (*F*_1_ × *F*_2_ × *F*_3_) and spectral widths of 3400, 350, and 5787 Hz, respectively, both employing mixing times of 700 msec. All data were processed using NMRPipe (Delaglio et al. 1995). Assignments and chemical shifts determinations were obtained using Sparky (T. D. Goddard, D. G. Kneller, *UCSF*). Chemical shift changes were calculated as the weighted average between the ^1^H and ^13^C or ^15^N resonance frequencies according to equation (1): (Cavanagh et al. 1996)


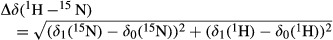
(1)

where Δ*δ* denotes the chemical shift difference (in Hz) between CaM in the free state (*δ*_0_) and with the addition of 10 mmol/L SF (*δ*_1_). The equivalent equation can be written for analysis of the ^1^H,^13^C-HSQC experiments.

SF (Abbott Laboratories, Solna, Sweden) was added in 1 *μ*L aliquots to the CaM NMR samples described above. The concentration of SF was estimated in 1D ^1^H spectra where signal intensities were compared with those of DSS, used as intern reference.

### Computation

**Figure 2:**
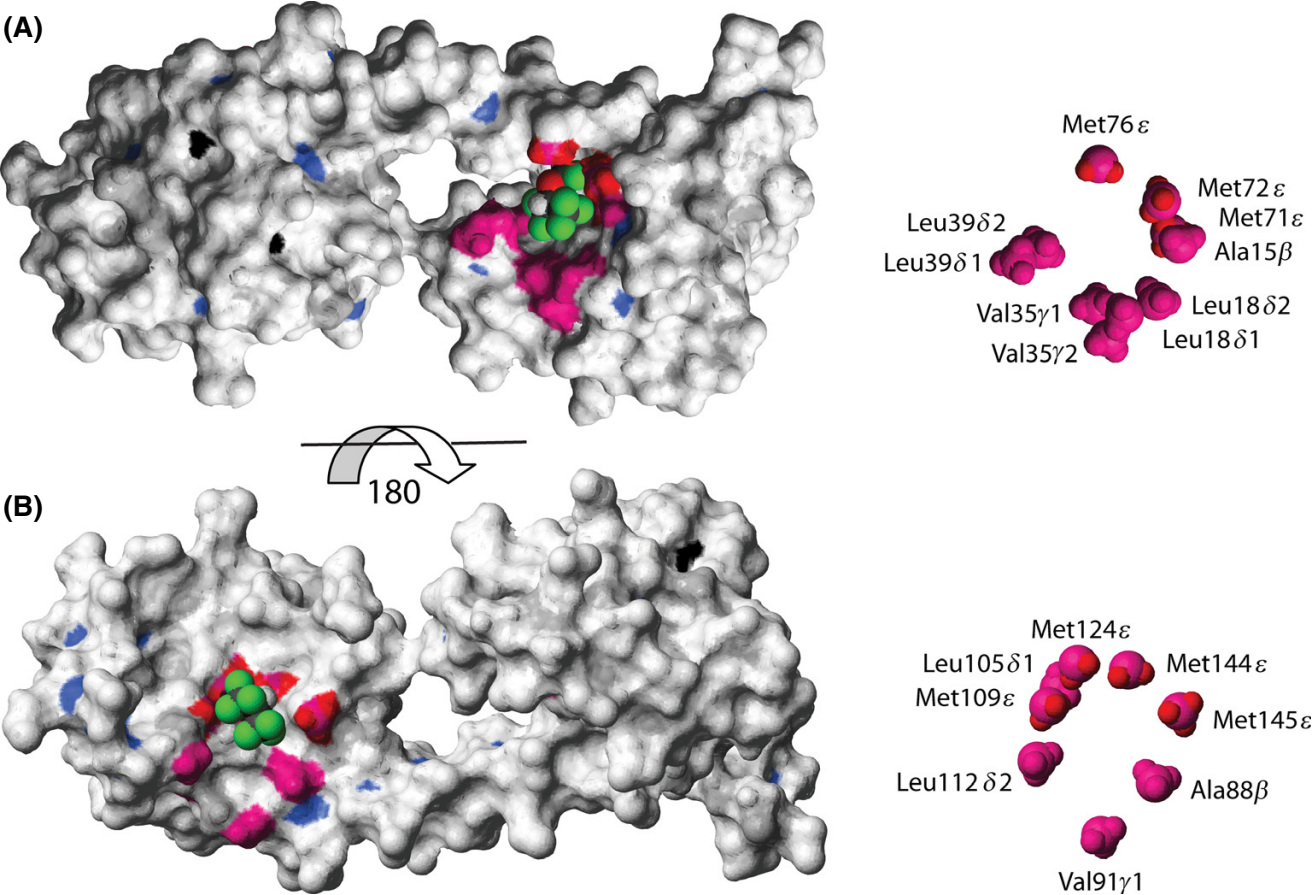
Structural identification of the sevoflurane (SF)-binding sites to (Ca^2+^)_4_-CaM. Surface representation of (Ca^2+^)_4_-CaM (pdb id 1X02) (Kainosho et al. 2006) color coded accordingly: amide atoms with Δ*δ*(^1^H,^15^N)>50 Hz in blue, methyl atoms with Δ*δ*(^1^H,^13^C)>50 Hz in magenta, Met H*ε* protons with NOEs to SF in red and Ca^2+^ ions in black. SF, docked using Glide (Schrödinger, Inc.), is shown in CPK representation. The N-terminal binding site for SF to (Ca^2+^)_4_-CaM is shown in (A); a view of the C-terminal binding site is obtained by turning the structure 180 degrees (B). A close-up of the methyl groups of each binding interface with assignments is shown, to the right.

Docking of SF to (Ca^2+^)_4_-CaM (pdb id 1X02) (Kainosho et al. 2006) was performed using the program Glide (Grid-based Ligand Docking with Energetics, Schrödinger, Inc., San Diego, CA), with the protein treated as a rigid body. This presumption is supported by the absence of any experimental indication for substantial conformational changes upon SF binding and is further motivated by the lack of a suitable theoretical model for description of internal motion of the ligand and the protein side chains of the binding pocket. Two receptor grids were prepared, one for each domain, with dimensions 10 × 10 × 10 Å^3^ centered on the methionine residues with intermolecular NOEs (nuclear Overhauser effects) to SF. Docking to the C-terminal domain was performed with alteration of the Met109–Met145 Cβ-Cγ-S*δ*-C*ε* dihedral angles to the theoretically available *trans* and *gauche* 180° or ±60°. Such rotation of *S*-methyl groups is thermodynamically allowed and is motivated by side chain rotations upon the (Ca^2+^)_4_-CaM–SF interaction, as seen in pronounced Δ*δ*(^1^H,^13^C) for all the included methionines. The resulting docked ensemble was filtered to retain docked structures fulfilling the intermolecular NOE distance restraints to be <5 Å. The structure with the lowest docking score is shown in Figure 2.

Figures 2 and 6 were prepared using Molmol (Koradi et al. 1996). Figure 6 displays an overlay of SF, W-7, and ion-channel-derived peptides from skeletal muscle isoform ryanodine receptor (RyR1) and NaV1.5 DIII-IV linker on the native (Ca^2+^)_4_-CaM structure (Kainosho et al. 2006; Maximciuc et al. 2006; Sarhan et al. 2012). The N-terminal and C-terminal domains of the four pdb (protein data bank) structures (1X02, 1MUX, 2BCX, and 4DJC) were overlaid individually using the backbone atoms of the residues defining the entries to the SF-binding sites.

### Isothermal titration calorimetry

For the SF affinity measurements, CaM lobes were dialyzed against 150 mmol/L KCl, 10 mmol/L HEPES, pH 7.4, 10 mmol/L CaCl_2_, and 0.1% NaN_3_ at 4°C. The structures of the N- and C-terminal CaM lobes are known to well represent those of the intact protein (Aulabaugh et al. 1984; Kainosho et al. 2006). Titrations consisted of 40 injections of 1 *μ*L W-7 (Sigma, St. Louis, MO) at 1.6 mmol/L in dialysis buffer into the cell containing either the N-lobe (residues −3–78) or C-lobe (79–148) (Ca^2+^)_4_-CaM at a 20-fold lower concentration with or without 20 mmol/L SF. Experiments were run on an ITC200 instrument (GE Healthcare) at 25°C. The data were processed using Origin 7.0 and fit using a two-site binding model. The second binding site was of very low affinity and thus differed appreciably. The apparent affinity of SF was calculated with equation (2) using the highest affinity W-7 binding site.


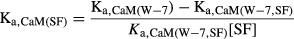
(2)

In order to measure Ca^2+^ affinities, CaM lobes were first dialyzed against 250 mmol/L KCl, 10 mmol/L HEPES, pH 7.4, 10 mmol/L EDTA, and 0.1% NaN_3_ at 4°C. The EDTA was then removed by extensive dialysis into the same buffer without EDTA. Solutions containing 0.4 mmol/L and 20 mmol/L SF were prepared by addition of the compound to the buffer. Background titrations were performed by titrating W-7 into buffer. We did not subtract them from the primary data because they were comparatively small (0.07 *μ*cal/sec) and the subtraction increased overall noise that rendered the refinement unstable. This may have a small impact on the apparent *K*_d_ values.

## Results

### NMR spectroscopic identification of the SF-binding clefts

Ligand-binding-induced protein NMR chemical shift changes report on alteration of the local environment in the vicinity of the monitored nuclei. The weighted chemical shift changes, (Cavanagh et al. 1996) Δ*δ*, of uniformly ^13^C and ^15^N isotopically enriched human *apo*- and (Ca^2+^)_4_-CaM backbone amide and side chain methyl nuclei were monitored upon successive additions of 0–10 mmol/L SF, using ^1^H,^15^N- and ^1^H,^13^C-HSQC experiments, respectively. Identical SF-titration experiments for *apo*- and (Ca^2+^)_4_-CaM allowed the monitoring of 150 backbone amide groups and 82 methyl groups, of which the majority (126 NH pairs and 64 methyl groups) are devoid of spectral overlap at both 0 and 10 mmol/L SF, and thus are available for analysis.

Additions of SF to samples of 0.2 mmol/L *apo*-CaM neither perturb the resonance frequencies of the backbone amides nor of the side chain methyl groups, even at 50 times excess of SF (Δ*δ*(^1^H,^15^N) and Δ*δ*(^1^H,^13^C) <11 Hz). The lack of substantial chemical shift displacements upon SF addition (Fig. 1B), with regard to the clinically used flurane concentrations of 0.3–0.7 mmol/L, (Franks and Lieb 1994; Streiff et al. 2004) precludes any biologically relevant *apo*-CaM–SF interaction.

Contrary to the observations for *apo*-CaM, additions of up to 10 mmol/L SF to (Ca^2+^)_4_-CaM in nine steps, displaced a significant population of the cross peaks in both protein domains locating two binding sites to one face of the N- and C-terminus, respectively. The backside of the respective domains is largely unperturbed, as indicated by the <50 Hz Δ*δ*(^1^H,^13^C) for their methyl groups. This magnitude limit was employed in the further analysis of Δ*δ*(^1^H,^13^C). With the addition of 10 mmol/L SF, the resonance frequencies of 49 amide and 29 methyl resonances are displaced by a weighted average above 50 Hz. The circumference of the entry to the N-terminal binding pocket is lined with hydrophobic methyl groups Ala15*β*, Leu18*δ*1/*δ*2, Val35*γ*1/*γ*2, Leu39*δ*1/*δ*2, Met72*ε*, and Met76*ε* (Figs. 2A, S1). The entry to the C-terminal binding pocket is guarded by the methyl groups of Ala88*β*, Val91*γ*2, Met109*ε*, Leu105*δ*1, Leu112*δ*2, Met124*ε*, Met144*ε*, and Met145*ε* (Fig. 2B). All of these 18 methyl groups exhibit significant Δ*δ*(^1^H,^13^C) of 52–253 Hz. Figure 1C displays the overlay of the methionine methyl region of the ^1^H,^13^C-HSQC spectra of (Ca^2+^)_4_-CaM with the addition of 0, 2.4, 5.0, and 10 mmol/L SF, clearly showing the described Δ*δ*(^1^H,^13^C) displacements in both (Ca^2+^)_4_-CaM domains.

Modest Δ*δ*(^1^H,^15^N) observed upon SF addition (Fig. S2) indicates lack of substantial structural rearrangements, which is in line with the conclusions of previous studies on the nature of protein-anesthetic interactions (Östergren 1944; Eckenhoff and Johansson 1997). The largest Δ*δ*(^1^H,^15^N) were detected for amino acids (Phe141, Gln143, Thr70, Val55) that are in close proximity to residues with significant Δ*δ*(^1^H,^13^C).

**Figure 3:**
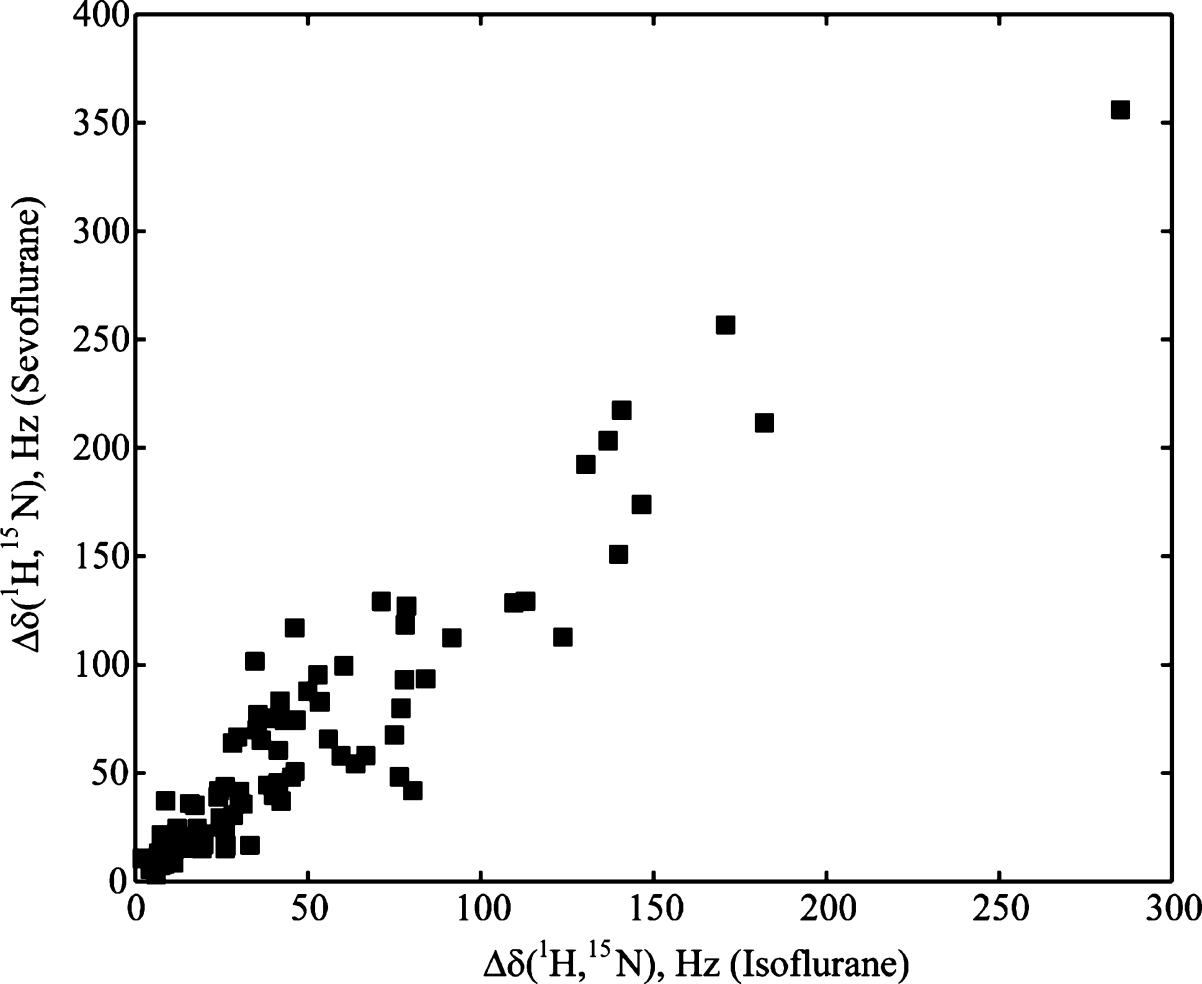
Correlation of the residue-specific chemical shift changes of (Ca^2+^)_4_-CaM upon titration with isoflurane and sevoflurane. Residue-specific Δ*δ* (^1^H,^15^N), in Hz, for 0.2 mmol/L U-[^13^C,^15^N]-(Ca^2+^)_4_-CaM with 0/10 mmol/L sevoflurane versus Δ*δ* (^1^H,^15^N), in Hz, for 0.1 mmol/L U-[^15^N]-(Ca^2+^)_4_-CaM with 0/13 mmol/L isoflurane, is shown as measured for 88 backbone amides.

The large structural resemblance along with a similar mode of action suggests the existence of a common intracellular binding site for sevo-, iso-, and desflurane, which are the volatile anesthetics in clinical use to date (Ebert and Schmid 2009). In a further attempt, the NMR titration of (Ca^2+^)_4_-CaM with isoflurane, analogous to that described above for SF, revealed comparable chemical shift changes for the very same ^1^H,^15^N-HSQC cross peaks (Fig. 3), supporting the presumption of the existence of a common volatile anesthetic-binding site (Franks and Lieb 1990; Dubois et al. 1993; Streiff et al. 2006).

Further atom-specific (Ca^2+^)_4_-CaM–SF distance restraints were obtained from 2D-^1^H,^1^H-NOESY and 3D-^1^H,^13^C,^1^H-HSQC–NOESY experiments. The presence of NMR active ^19^F nuclei in SF also enabled the recording of 2D-^19^F,^1^H-HOESY experiments. All three spectra (Fig. S3–5) show clear intermolecular cross peaks between the SF resonances, *δ*(^1^H) 5.47 ppm and *δ*(^19^F) -74.4 ppm, and (Ca^2+^)_4_-CaM protons at *δ*(^1^H) 0.2–2.1 ppm, corresponding to the protein methyl region. As the methionine methyl ^1^H resonances suffer little spectral overlap, assignment of intermolecular cross peaks was carried out using a 3D-^1^H,^13^C,^1^H-HSQC–NOESY experiment, which enabled the unambiguous assignment of intermolecular cross peaks between the SF protons at *δ*(^1^H) 5.47 ppm and the methyl protons of (Ca^2+^)_4_-CaM Met71, Met72, Met76, Met109, Met124, Met144, and Met145. These methionine methyl groups also all exhibit significant Δ*δ*(^1^H,^13^C). Thus, seven intermolecular NOEs confirm the location of the binding pockets obtained from the chemical shift displacements, and unambiguously define two SF-binding sites, one in each (Ca^2+^)_4_-CaM lobe. Docking of SF to (Ca^2+^)_4_-CaM (pdb id 1X02) (Kainosho et al. 2006) was performed using the program Glide (Schrödinger, Inc.). The resulting complex is shown in Figure 2. Although the locations of the binding clefts are unambiguous, the possibility that each binding site could be occupied by more than one SF molecule cannot be excluded based on the NMR spectroscopic evidence obtained here. Furthermore, the size of the binding pocket and the hydrophobic nature of the ligand-protein interaction may allow SF to reorient in the binding pocket without a significant energetic penalty.

Ca^2+^ binding to *apo*-CaM exposes hydrophobic regions necessary for target enzyme activation (Tanaka and Hidaka 1980). These N- and C-terminal patches, containing 18 and 19 methyl groups, respectively, (Babu et al. 1988) interact with peptides derived from various target enzymes (Meador et al. 1993) and ion channels (Saimi and Kung 2002; Sarhan et al. 2012). Besides the methyl groups lining the circumferences of the SF-binding sites, all other methyl groups observed in this study to experience significant Δ*δ*(^1^H,^13^C) (Met36*ε*, Met51*ε*, Ile52*δ*1, Val55*γ*1/*γ*2, Ile63*δ*1, Met71*ε*, Ile100*δ*1/*γ*2, Val108*γ*2, and Ile125*δ*1) are part of these hydrophobic regions.

### Isothermal titration calorimetry analysis of binding affinities

**Figure 4:**
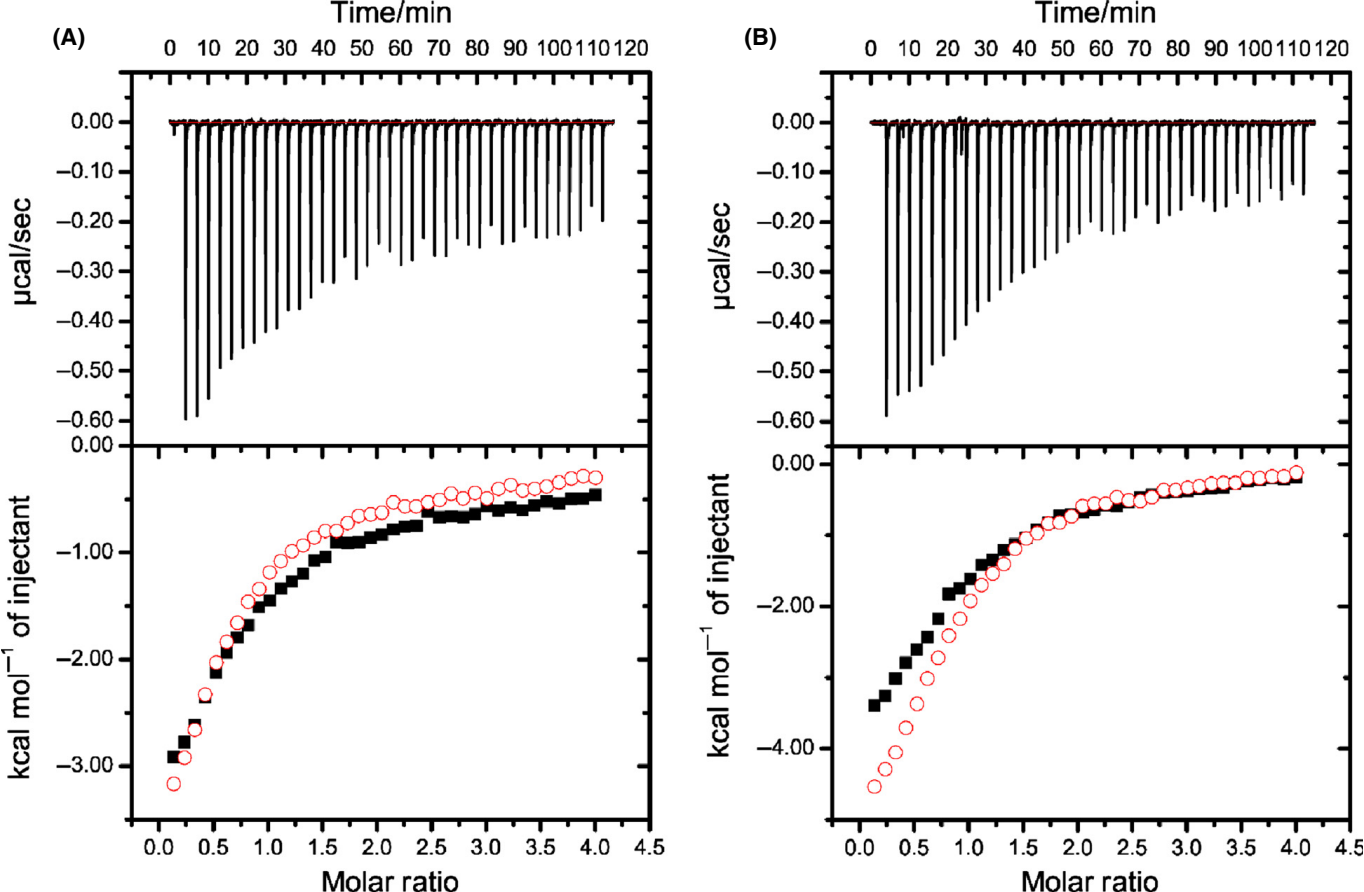
Determination of the sevoflurane (SF) affinity for the individual (Ca^2+^)_2_-loaded CaM lobes by isothermal titration calorimetry (ITC). Representative ITC experiments showing binding of antagonist W-7 with the (Ca^2+^)_2_-loaded CaM N-lobe (A) and C-lobe (B) in the presence of 20 mmol/L SF. The upper panels represent ITC raw data with each peak corresponding to an injection event. The lower panels show integrated values for W-7 binding to (Ca^2+^)_2_-CaM lobes in the presence (black squares) and absence (open red circles) of 20 mmol/L SF. Integrated values are fit to a two-site binding model, with the first high-affinity site used to determine the affinity of SF. The second binding-site is too weak to quantify accurately. The W-7 binding affinities of (Ca2+)_4_-CaM were observed to alter from *K*_d_ 5.5 to 18.0 *μ*mol/L and from *K*_d_ 15.6 to 32.6 *μ*mol/L for the C and N-lobes, respectively, upon changing the SF concentration from 0 to 20 mmol/L. The aqueous SF concentration (20 mmol/L) of the solutions utilized in the ITC experiments was confirmed by ^1^H NMR (Fig. S6). SF binding affinities are given in the text.

**Figure 5:**
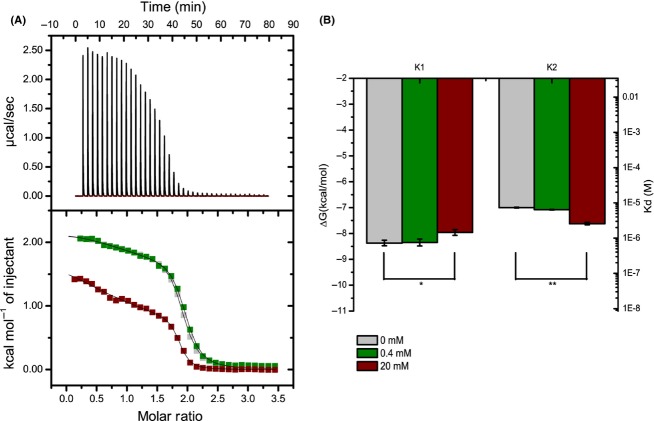
The sevoflurane (SF)-dependent Ca^2+^ affinity for the individual lobes assessed by isothermal titration calorimetry (ITC). ITC experiments were utilized to measure the affinity of CaM lobes for Ca^2+^ in the presence and absence of SF. (A) Upper panel: Typical ITC curve for CaM–Ca^2+^ interaction. Lower panel: Overlay of integrated ITC data for the N-lobe. Titrations consisted of 40 injections of 1 *μ*L CaCl_2_ at 1.6 mmol/L into the cell containing the N-lobe at a 20-fold lower concentration (0.5 mmol/L), and with SF concentrations at 0 (gray), 0.4 (green), or 20 mmol/L SF (red) The measurement performed with 0.4 mmol/L SF is pharmacologically relevant as it corresponds to the clinical blood concentration of SF at anesthesia (Franks and Lieb 1994; Streiff et al. 2004). (B) Bar graphs indicating the average ΔG and *K*_d_ for the N-lobe binding sites. The error bars indicate standard errors based on three independent measurements for each titration. Two-tailed paired *t*-test: *P = 0.047; **P = 0.0075.

The SF–CaM affinity and the Ca^2+^–CaM affinity in the presence of SF were assessed using isothermal titration calorimetry (ITC). The SF affinity was found too weak to be accurately detected in a conventional ITC experiment, but assuming a competitive binding model, the affinity of SF for the individual CaM domains could be estimated from ITC competition experiments of the antagonist W-7 (Osawa et al. 1998) and SF, both shown to bind to the same hydrophobic pockets in (Ca^2+^)_4_-CaM (Streiff et al. 2004). Using a competition experiment involving W-7, the apparent SF *K*_d_ for the C-lobe was determined to be 8.8 ± 5.5 mmol/L, whereas that for the N-lobe to be 18.3 ± 8.7 mmol/L (Fig. 4). Because of the weak affinity of SF for the lobes, there is only a small difference in apparent *K*_d_ for W-7, resulting in a significant contribution of any noise in the experiments. However, the data clearly indicate that the SF affinities are indeed weak, and the determined *K*_d_ of the domains agrees well with the comparable NMR chemical shift changes observed throughout the (Ca^2+^)_4_-CaM SF-titration experiments. As volatile anesthetic binding to CaM may regulate cellular excitability by modulation of its Ca^2+^ affinity, the impact of SF on the Ca^2+^-affinity of CaM was assessed. Upon addition of clinically relevant (Franks and Lieb 1994; Streiff et al. 2004) 0.4 mmol/L SF, no significant alteration of the Ca^2+^-binding affinities of the N- and the C-lobes were observed. At saturating SF level (20 mmol/L), no significant alteration of the C-lobe affinities yet a slight increase and decrease in affinities for the individual EF hands in the N-lobe were observed (Fig. 5).

## Discussion and Conclusions

SF interacts with CaM in its Ca^2+^ bound form only, and in line with previous predictions (Östergren 1944; Eckenhoff and Johansson 1997; Streiff et al. 2006) without substantial perturbation of its structure. On the contrary, small hydrophobic pharmaceutics previously reported to bind CaM with high affinity yet without anesthetic activity, such as trifluoperazine, were shown to cause significant conformational change in the protein (Vandonselaar et al. 1994; Horváth et al. 2005). Additional studies of isoflurane binding to (Ca^2+^)_4_-CaM indicate a common interaction site (Franks and Lieb 1990) for both fluranes, which belong to the family of general anesthetics in current clinical practice. This observation is in line with the hypothesis of a common mechanism of action of volatile anesthetics, (Franks and Lieb 1990; Dubois et al. 1993; Streiff et al. 2006), which notion is likely applicable to the structurally closely related fluranes, but may have limitations when considering different compound classes (Villars et al. 2004).

The precise nature of the binding force(s) behind anesthetic–protein interaction remains a matter of debate with the possibly dominant role of hydrophobic forces, van der Waals and electrostatic interactions or hydrogen bonding and the impact of their interplay having been argued for (Di Paolo and Sándorfy 1974; Eckenhoff and Johansson 1997; Sandorfy 2004; Trogdon et al. 2007). Lately the possible importance of halogen bonding in anesthetic action has also been raised (Metrangolo et al. 2005). Consequently, as polyfluorinated compound SF is overall hydrophobic (log *P* = 2.8) and is likely to act as fluorous hydrogen bond acceptor, whereas its activated CH proton may function as hydrogen bond donor. Its high density in electron withdrawing functionalities may allow its involvement in fluorine-centered halogen bond interaction with the protein backbone and with polar amino acid side chains (Metrangolo et al. 2011). Upon SF titration of (Ca^2+^)_4_-CaM the largest chemical shift changes were observed for specific side chain methyl groups whereas the Δ*δ*(^1^H,^15^N) of backbone amides were smaller and somewhat more diffusely localized, the latter being indicative of minor local dislocations rather than of direct hydrogen or halogen bonding events. Comparable, less localized ^15^N chemical shift changes were previously detected upon noble gas binding to proteins (Nisius et al. 2005), which interaction exclusively proceeds through hydrophobic forces. The likely importance of hydrophobic forces in the (Ca^2+^)_4_-CaM–SF interaction was further supported by the detection of ^1^H-^1^H and ^19^F-^1^H nuclear Overhauser effects in the amino acid side chain methyl region, and by the overall weak nature of the interaction (*K*_d_ ∼ mmol/L). These observations are in agreement with the computed surface electrostatic potential of SF, (Tang et al. 2001) which predicts a hydrophobic SF surface without sign of formation of a partially positively charged sigma hole on any of the fluorines (Metrangolo et al. 2005, 2011). It should also be noted that the SF-binding sites identified in this study were previously pointed out as highly hydrophobic regions of CaM (Tanaka and Hidaka 1980). Hence, our observations rather indicate the dominant role of hydrophobic forces in the (Ca^2+^)_4_-CaM–SF interaction than a hydrogen bond breakage-mediated mechanism (Di Paolo and Sándorfy 1974). The hydrophobicity of the binding cleft may further be significant for the drug interactions of anesthetics with hydrophobic pharmaceuticals (Glass 1998).

SF has only minor effects on the Ca^2+^-binding affinity of *apo*-CaM. Previous studies of the effect of volatile anesthetics (halothane, isoflurane) on the Ca^2+^ affinity of CaM have reported ambiguous behavior, including both increase and decrease in affinity, which depending on the experimental conditions were observed reversible or irreversible (Levin and Blanck 1995; Blanck 1999a,b; Streiff et al. 2006). All in all, administration of volatile anesthetics at physiologically relevant concentration does not cause significant overall increase or decrease in the Ca^2+^ affinity of CaM.

**Figure 6:**
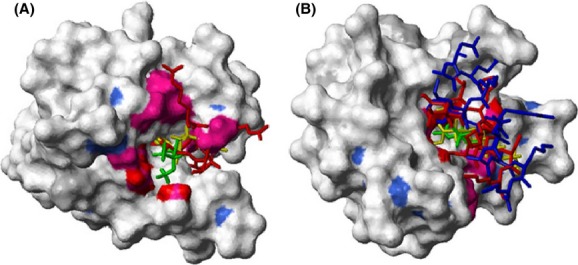
Superimposed structures of (Ca^2+^)_4_-CaM binding sevoflurane (SF), W-7, and ion channel domains. Surface representation of (Ca^2+^)_4_-CaM (pdb id 1X02) (Kainosho et al. 2006) N-terminal domain (A, residues 13–80 for clarity) and C-terminal domain (B, residues 81–148). For color coding scheme, see Figure 2. Ligands are shown in stick representation with SF (docked using Glide, Schrödinger, Inc.) in green, W-7 in yellow, skeletal muscle isoform ryanodine receptor (RYR1) peptide representing (residues Arg3629–Met3638 and Ala3618–Lys3626 interacting with the N- and C-terminal domain, respectively) in red and human NaV1.5 DIII-IV linker, residues Asn1489–Lys1500, in blue.

The SF-binding sites identified above overlap with the binding clefts of physiologically important peptides, (Saimi and Kung 2002; Maximciuc et al. 2006; Sarhan et al. 2012) including those derived from ion channels such as RyR1 (Fig. 6) (Maximciuc et al. 2006). Malignant hyperthermia is the anesthetic treatment-induced opening of the RyR1 receptor that causes an uncontrolled increase in skeletal muscle oxidative metabolism (Robinson et al. 2006). As (Ca^2+^)_4_-CaM inhibits RyR1 and thereby decreases Ca^2+^ release from the sarcoplasmic reticulum, one could argue that SF competition with RyR1 for the (Ca^2+^)_4_-CaM-binding site may indirectly affect Ca^2+^ homeostasis and thereby contribute to the pathogenesis of malignant hyperthermia (Brath et al. 2013). Similarly, one could argue decreased cellular excitability due to dissociation of (Ca^2+^)_4_-CaM from voltage-gated Na^+^ channels through competition with SF (Fig. 6B), (Sarhan et al. 2012) or modulation of the function of other cellular receptors, such as GABA_A_ channels (Mody et al. 1991; Kress and Tas 1993). The low millimolar SF-binding affinity is in line with previous observations and predictions, (Eckenhoff and Johansson 1997; Streiff et al. 2004, 2006) and with the clinically used thousand times higher blood concentration of anesthetics as compared to other typical high-affinity pharmacons (Franks and Lieb 1994). However, with a millimolar affinity for (Ca^2+^)_4_-CaM, SF is unlikely to impact the binding of CaM targets with nanomolar affinities. Since the clinical concentrations for SF are ∼10-fold below the measured *K*_d_ values, it would only be an efficient competitor at overdose concentrations, or if the presence of other proteins in the cytosol increase the affinity through allosteric interactions. Even in those situations, any CaM-mediated effect of SF would likely be limited to very weak CaM targets. We suspect that SF may have the largest effect on those CaM targets where the binding requires Ca^2+^ and where both CaM lobes are involved. The physiological relevance of the (Ca^2+^)_4_-CaM–anesthetic interaction (Landers et al. 1989) and its interplay with additional regulatory proteins should therefore be the target for future studies.

In conclusion, on the basis of NMR evidences we report the first structure of the clinically utilized anesthetic SF bound to a protein target. The interaction is of low mmol/L affinity. Undoubtedly, many more targets for SF reside in the cell, and the structure of the SF-(Ca^2+^)_4_-CaM complex will aid in prediction of other SF-binding pockets of other proteins. Whether SF is also able to bind to other EF-hand containing proteins remains to be discovered.

## Abbreviations

CaM: calmodulin
HOESY: heteronuclear Overhauser enhancement spectroscopy
HSQC: heteronuclear single quantum correlation
IPTG: isopropyl-*β*-d-1-thiogalactopyranoside
ITC: isothermal titration calorimetry
pLacI: plasmid that encodes the lac repressor protein
RyR: skeletal muscle isoform ryanodine receptor
SF: sevoflurane

## Supporting Information

**Figure S1.** Plot of Δ*δ*(^1^H,^13^C), in Hz, plotted for each methyl group, in sequence order, comparing samples of 0.2 mmol/L (Ca^2+^)_4_-CaM with 0 or 10 mmol/L SF, further details are given in the main text. **Figure S2.** Plot of Δ*δ*(^1^H,^15^N), in Hz, versus residue number, comparing samples of 0.2 mmol/L (Ca^2+^)_4_-CaM with 0 or 10 mmol/L SF, further details are given in the main text. **Figure S3.** Expansion of ^1^H,^1^H-NOESY spectrum of (Ca^2+^)_4_-CaM with SF (details given above) spanning offdiagonal cross peaks between SF signals *δ*(*F*_2_,^1^H) 5.44 and 5.50 ppm (doublet) and *δ*(*F*_2_,^1^H) 5.26 and *δ*(*F*_1_,^1^H) 0.6–2.4, where the latter corresponds to the (Ca^2+^)_4_-CaM methyl region. **Figure S4.** ^19^F,^1^H-HOESY spectrum of (Ca^2+^)_4_-CaM with SF (details given above) spanning off-diagonal cross peaks between the SF signal at *δ*(*F*_2_,^19^F) -74.4 ppm and *δ*(*F*_1_,^1^H) 0.6–2.4, where the latter corresponds to the (Ca^2+^)_4_-CaM methyl region. **Figure S5.** 2D plane of 3D-^1^H,^13^C,^1^H-HSQC–NOESY acquired on (Ca^2+^)_4_-CaM with SF. The *F*_1_ × *F*_2_ plane at *δ*(*F*_3_,^1^H) 5.41 ppm corresponding to one of the SF doublet signals is shown. Cross peaks available in these plane, assigned to Met71*ε*, Met72*ε*, Met76*ε*, Met109*ε*, Met124*ε*, Met144*ε*, and Met145*ε*, report on (Ca^2+^)_4_-CaM–SF NOE interactions. **Figure S6.** The concentration of sevoflurane (SF) in its saturated aqueous solution used in the ITC studies was determined by ^1^H NMR (400 MHz) using a D_2_O solution of SF and 4,4-dimethyl-4-silapentane-1-sulfonic acid (DSS, 0.1 mmol/L) as internal reference (the signal corresponds to 9H and its integral is set to 1.00 here). Long *d*_1_ relaxation time (25 sec) was used and no presaturation of the solvent signal. The aqueous SF concentration was determined to 19.7 mmol/L. **Data S1**. PDB coordinates of the (Ca^2+^)_4_-CaM–SF complex.
