# Supplementary material for: Mapping the sevoflurane-binding sites of calmodulin
Source: Pharmacol Res Perspect. 2014 Feb 12;2(1):5. doi: 10.1002/prp2.25 (PMC4186402; doi:10.1002/prp2.25)
Supplement: Supplementary file 1 [file prp20002-e00025-SD1.pdf]

Supplemental Information

Mapping the Sevoflurane Binding Sites of Calmodulin

Ulrika Brath, Kelvin Lau, Filip Van Petegem, Máté Erdélyi*

Table of Contents

S2 Figure S1. The $\Delta\delta$(^{1}H,^{13}C) of $(Ca^{2+})_4$-CaM, in Hz, upon addition of sevoflurane.

Figure S2. The $\Delta\delta$(^{1}H,^{15}N) of $(Ca^{2+})_4$-CaM, in Hz, upon addition of sevoflurane.

S3 Figure S3. ^{1}H,^{1}H-NOESY spectrum of $(Ca^{2+})_4$-CaM with sevoflurane.

Figure S4. ^{19}F,^{1}H-HOESY spectrum of $(Ca^{2+})_4$-CaM with sevoflurane.

S4 Figure S5. 2D plane of 3D-^{1}H,^{13}C,^{1}H-HSQC–NOESY acquired on $(Ca^{2+})_4$-CaM with SF.

Figure S6. ^{1}H NMR determination of the aqueous concentration of sevoflurane.

S5 PDB coordinates of the $(Ca^{2+})_4$-CaM – SF complex.

S65 References

Supplemental Figures

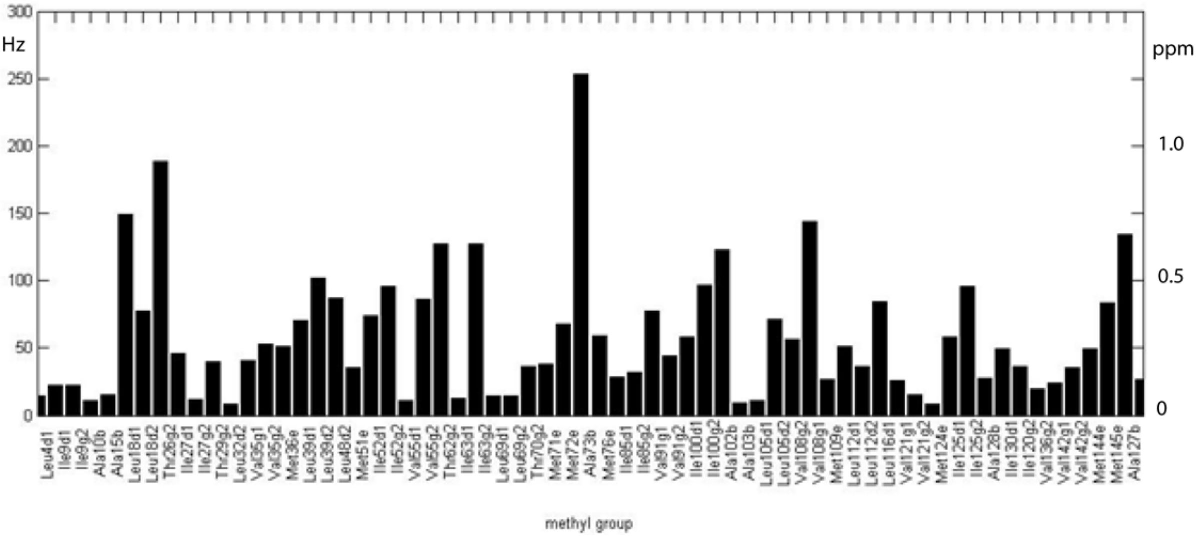

Supplemental Figure S1. $\Delta\delta$ (^{1}H,^{13}C), in Hz, plotted for each methyl group, in sequence order, comparing samples of 0.2 mM (Ca^{2+})$_4$-CaM with 0 or 10 mM SF, further details are given in the main text.

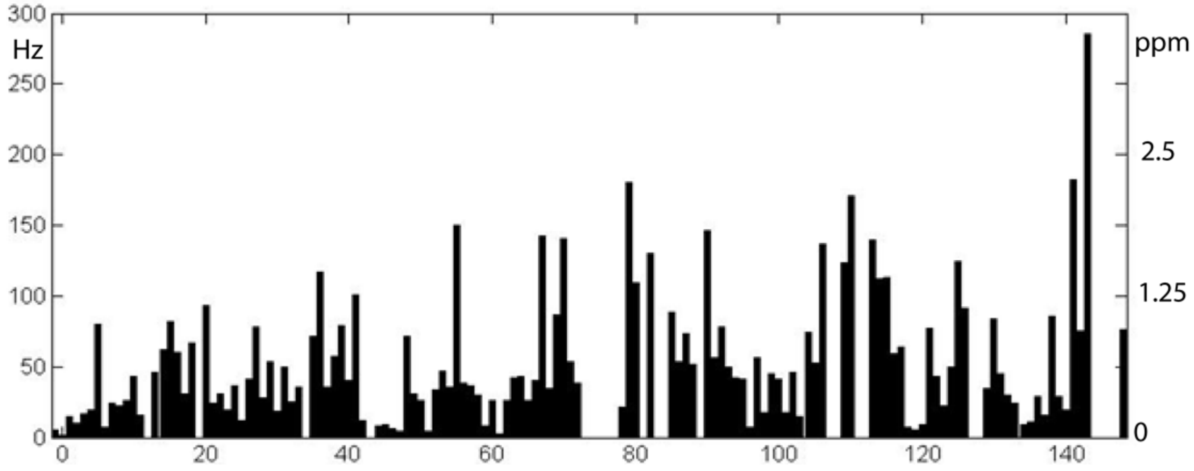

Supplemental Figure S2. Plot of $\Delta\delta$ (^{1}H,^{15}N), in Hz, versus residue number, comparing samples of 0.2 mM (Ca^{2+})$_4$-CaM with 0 or 10 mM SF, further details are given in the main text.

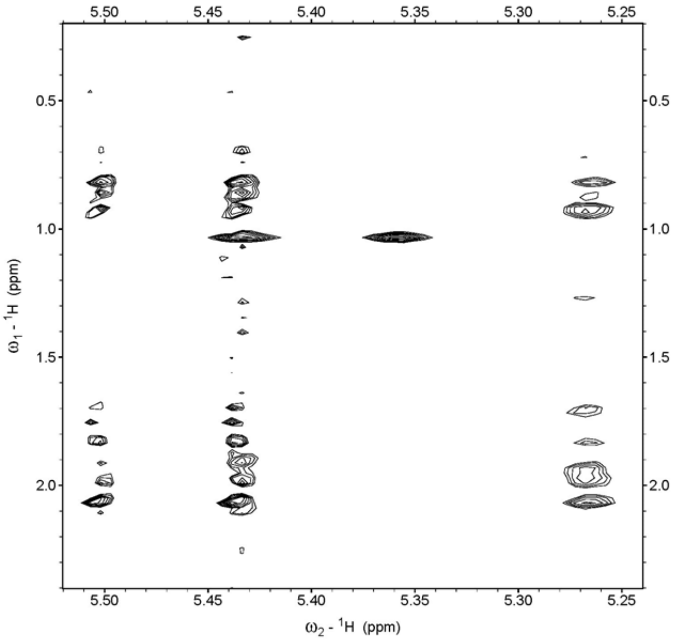

Supplemental Figure S3. Expansion of ^{1}H,^{1}H-NOESY spectrum of $(Ca^{2+})_4$-CaM with SF (details given above) spanning off-diagonal cross peaks between SF signals $\delta(F_2, {}^1H)$ 5.44 and 5.50 ppm (doublet) and $\delta(F_2, {}^1H)$ 5.26 and $\delta(F_1, {}^1H)$ 0.6 – 2.4, where the latter corresponds to the $(Ca^{2+})_4$-CaM methyl region.

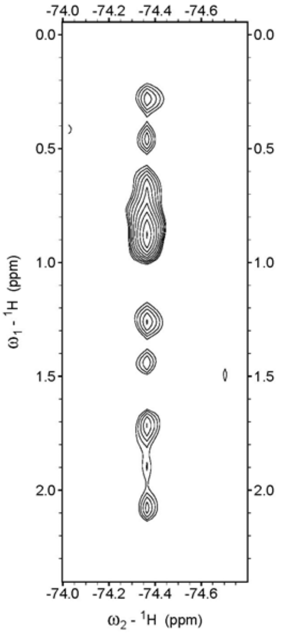

Supplemental Figure S4. ^{19}F,^{1}H-HOESY spectrum of $(Ca^{2+})_4$-CaM with SF (details given above) spanning off-diagonal cross peaks between the SF signal at $\delta(F_2, {}^{19}F)$ -74.4 ppm and $\delta(F_1, {}^1H)$ 0.6 – 2.4, where the latter corresponds to the $(Ca^{2+})_4$-CaM methyl region.

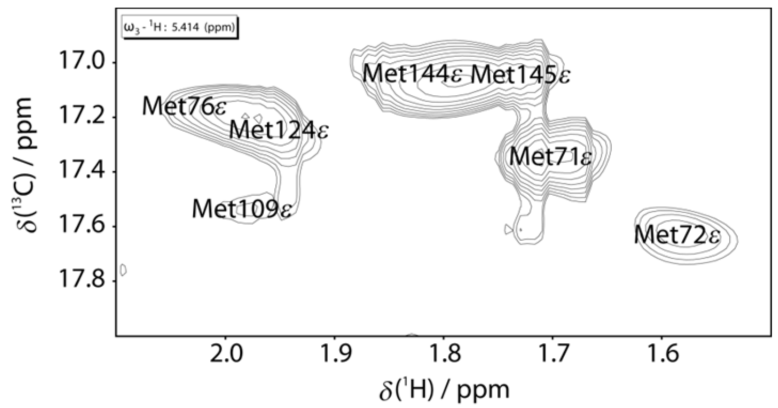

Supplementary Figure S5. 2D plane of 3D-^{1}H,^{13}C,^{1}H-HSQC–NOESY acquired on $(Ca^{2+})_4$-CaM with SF. The F_1xF_2 plane at $\delta(F_3$, ^{1}H) 5.41 ppm corresponding to one of the SF doublet signals is shown. Cross peaks available in these plane, assigned to Met71ε, Met72ε, Met76ε, Met109ε, Met124ε, Met144ε and Met145ε, report on $(Ca^{2+})_4$-CaM – SF NOE interactions.

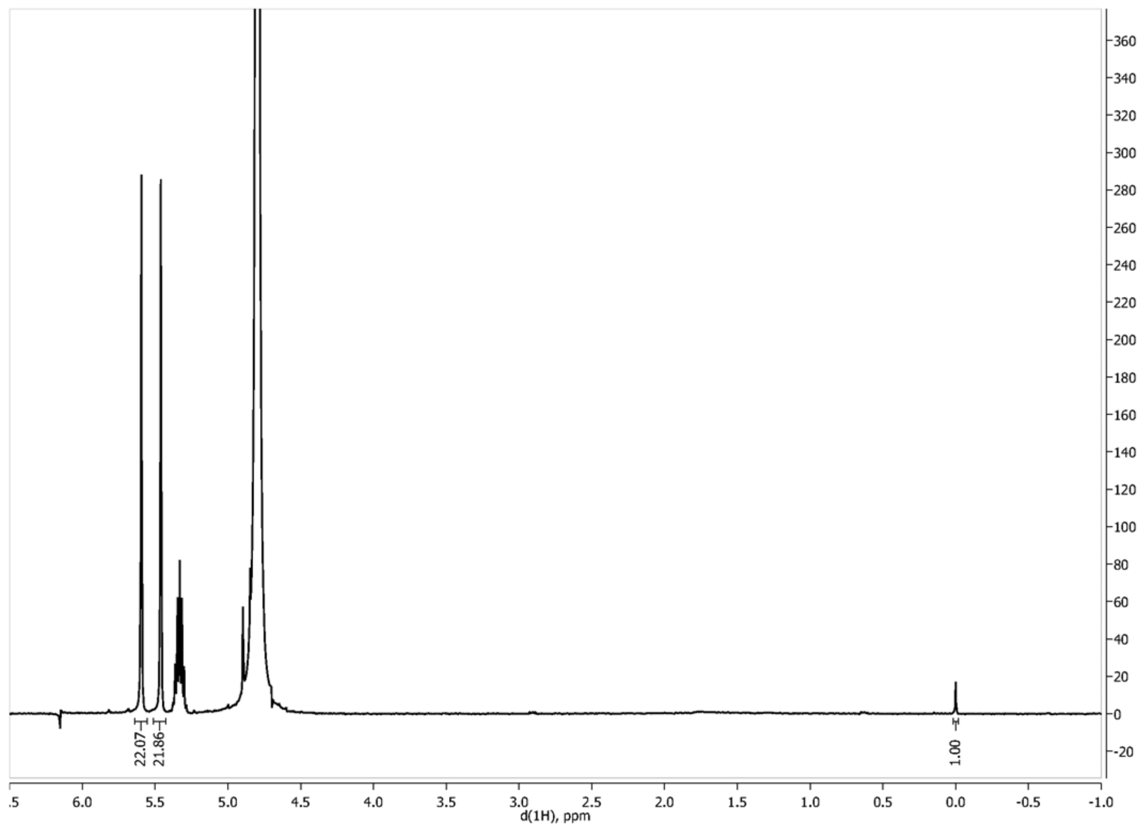

Supplemental Figure S6. The concentration of sevoflurane (SF) in its saturated aqueous solution used in the ITC studies was determined by ^{1}H NMR (400 MHz) using a D_2O solution of SF and 4,4-dimethyl-4-silapentane-1-sulfonic acid (DSS, 0.1 mM) as internal reference (the signal corresponds to 9H and its integral is set to 1.00 here). Long d_1 relaxation time (25 sec) was used and no presaturation of the solvent signal. The aqueous SF concentration was determined to 19.7 mM.

PDB coordinates of the $(Ca^{2+})_4$-CaM – SF complex

Docking of sevoflurane to $(Ca^{2+})_4$-CaM (pdb id 1X02) was performed using the program Glide (Grid-based Ligand Docking with Energetics, Schrödinger, Inc.), as described in detail in the experimental session of the main text.

```
TITLE     1x02_reduced_withoutplus_cacorrect
REMARK 888
REMARK 888 WRITTEN BY MAESTRO (A PRODUCT OF SCHRODINGER, LLC)
HELIX   49  49 GLU      6  SER     17  1                                  12
HELIX   50  50 THR     29  LEU     39  1                                  11
HELIX   51  51 GLU     45  VAL     55  1                                  11
HELIX   52  52 PHE     65  ASP     78  1                                  14
HELIX   53  53 ASP     80  PHE     92  1                                  13
HELIX   54  54 ALA    102  GLY    113  1                                  12
HELIX   55  55 ASP    118  ALA    128  1                                  11
HELIX   56  56 TYR    138  MET    145  1                                   8
MODEL        1
ATOM      1  N   ALA     1      32.285  14.223   7.039  1.00  0.00           N
ATOM      2  CA  ALA     1      31.935  12.933   7.622  1.00  0.00           C
ATOM      3  C   ALA     1      32.819  12.616   8.822  1.00  0.00           C
ATOM      4  O   ALA     1      33.074  11.451   9.128  1.00  0.00           O
ATOM      5  CB  ALA     1      32.050  11.834   6.575  1.00  0.00           C
ATOM      6  HA  ALA     1      30.906  12.981   7.947  1.00  0.00           H
ATOM      7 1HB  ALA     1      33.077  11.746   6.256  1.00  0.00           H
ATOM      8 2HB  ALA     1      31.724  10.897   7.001  1.00  0.00           H
ATOM      9 3HB  ALA     1      31.428  12.079   5.727  1.00  0.00           H
ATOM     10 1H   ALA     1      32.353  14.305   6.065  1.00  0.00           H
ATOM     11  H1  ALA     1      31.592  14.911   7.301  1.00  0.00           H
ATOM     12  H2  ALA     1      33.191  14.513   7.379  1.00  0.00           H
ATOM     13  N   ASP     2      33.284  13.660   9.501  1.00  0.00           N
ATOM     14  CA  ASP     2      34.139  13.492  10.670  1.00  0.00           C
ATOM     15  C   ASP     2      33.316  13.107  11.895  1.00  0.00           C
ATOM     16  O   ASP     2      33.809  12.436  12.800  1.00  0.00           O
ATOM     17  CB  ASP     2      34.920  14.778  10.946  1.00  0.00           C
ATOM     18  CG  ASP     2      35.961  15.065   9.882  1.00  0.00           C
ATOM     19  OD1 ASP     2      36.455  14.100   9.262  1.00  0.00           O
ATOM     20  OD2 ASP     2      36.282  16.254   9.670  1.00  0.00           O
ATOM     21  H   ASP     2      33.045  14.565   9.208  1.00  0.00           H
ATOM     22  HA  ASP     2      34.840  12.697  10.457  1.00  0.00           H
ATOM     23 2HB3 ASP     2      35.419  14.691  11.900  1.00  0.00           H
ATOM     24  HB2 ASP     2      34.232  15.625  10.963  1.00  0.00           H
ATOM     25  N   GLN     3      32.060  13.542  11.917  1.00  0.00           N
ATOM     26  CA  GLN     3      31.170  13.244  13.033  1.00  0.00           C
ATOM     27  C   GLN     3      30.420  11.938  12.798  1.00  0.00           C
ATOM     28  O   GLN     3      29.274  11.781  13.220  1.00  0.00           O
ATOM     29  CB  GLN     3      30.173  14.388  13.236  1.00  0.00           C
ATOM     30  CG  GLN     3      30.767  15.591  13.951  1.00  0.00           C
ATOM     31  CD  GLN     3      31.160  15.285  15.383  1.00  0.00           C
ATOM     32  OE1 GLN     3      30.363  15.448  16.306  1.00  0.00           O
ATOM     33  NE2 GLN     3      32.395  14.836  15.574  1.00  0.00           N
ATOM     34  H   GLN     3      31.725  14.075  11.167  1.00  0.00           H
ATOM     35  HA  GLN     3      31.774  13.144  13.921  1.00  0.00           H
ATOM     36 2HB3 GLN     3      29.341  14.024  13.820  1.00  0.00           H
ATOM     37 2HG3 GLN     3      30.034  16.386  13.957  1.00  0.00           H
ATOM     38 1HE2 GLN     3      32.975  14.729  14.790  1.00  0.00           H
ATOM     39 2HE2 GLN     3      32.676  14.629  16.489  1.00  0.00           H
ATOM     40  HB2 GLN     3      29.826  14.745  12.266  1.00  0.00           H
ATOM     41  HG2 GLN     3      31.668  15.915  13.431  1.00  0.00           H
ATOM     42  N   LEU     4      31.075  11.000  12.120  1.00  0.00           N
ATOM     43  CA  LEU     4      30.470   9.705  11.829  1.00  0.00           C
ATOM     44  C   LEU     4      31.451   8.570  12.106  1.00  0.00           C
ATOM     45  O   LEU     4      31.678   7.710  11.253  1.00  0.00           O
ATOM     46  CB  LEU     4      30.008   9.651  10.371  1.00  0.00           C
ATOM     47  CG  LEU     4      28.937  10.665   9.968  1.00  0.00           C
ATOM     48  CD1 LEU     4      29.125  11.096   8.523  1.00  0.00           C
ATOM     49  CD2 LEU     4      27.546  10.082  10.177  1.00  0.00           C
ATOM     50  H   LEU     4      31.985  11.181  11.811  1.00  0.00           H
ATOM     51  HA  LEU     4      29.612   9.586  12.475  1.00  0.00           H
ATOM     52 2HB3 LEU     4      29.616   8.661  10.186  1.00  0.00           H
ATOM     53  HG  LEU     4      29.029  11.543  10.593  1.00  0.00           H
ATOM     54 1HD1 LEU     4      28.815  12.124   8.410  1.00  0.00           H
ATOM     55 2HD1 LEU     4      28.527  10.468   7.880  1.00  0.00           H
ATOM     56 3HD1 LEU     4      30.166  11.002   8.253  1.00  0.00           H
ATOM     57 1HD2 LEU     4      27.242  10.236  11.202  1.00  0.00           H
```

ATOM	58	2HD2	LEU	4	27.566	9.024	9.962	1.00	0.00	H
ATOM	59	3HD2	LEU	4	26.847	10.574	9.516	1.00	0.00	H
ATOM	60	HB2	LEU	4	30.850	9.873	9.714	1.00	0.00	H
ATOM	61	N	THR	5	32.031	8.574	13.302	1.00	0.00	N
ATOM	62	CA	THR	5	32.988	7.545	13.691	1.00	0.00	C
ATOM	63	C	THR	5	32.281	6.247	14.057	1.00	0.00	C
ATOM	64	O	THR	5	31.062	6.130	13.914	1.00	0.00	O
ATOM	65	CB	THR	5	33.847	8.001	14.884	1.00	0.00	C
ATOM	66	OG1	THR	5	33.126	8.955	15.674	1.00	0.00	O
ATOM	67	CG2	THR	5	35.155	8.618	14.406	1.00	0.00	C
ATOM	68	H	THR	5	31.808	9.286	13.936	1.00	0.00	H
ATOM	69	HA	THR	5	33.641	7.364	12.850	1.00	0.00	H
ATOM	70	HB	THR	5	34.076	7.140	15.495	1.00	0.00	H
ATOM	71	1HG	THR	5	33.583	9.093	16.507	1.00	0.00	H
ATOM	72	1HG2	THR	5	35.488	9.355	15.121	1.00	0.00	H
ATOM	73	2HG2	THR	5	34.999	9.090	13.447	1.00	0.00	H
ATOM	74	3HG2	THR	5	35.902	7.845	14.311	1.00	0.00	H
ATOM	75	N	GLU	6	33.048	5.271	14.530	1.00	0.00	N
ATOM	76	CA	GLU	6	32.494	3.979	14.915	1.00	0.00	C
ATOM	77	C	GLU	6	31.081	4.138	15.472	1.00	0.00	C
ATOM	78	O	GLU	6	30.216	3.292	15.246	1.00	0.00	O
ATOM	79	CB	GLU	6	33.390	3.305	15.955	1.00	0.00	C
ATOM	80	CG	GLU	6	34.713	2.812	15.393	1.00	0.00	C
ATOM	81	CD	GLU	6	34.533	1.741	14.335	1.00	0.00	C
ATOM	82	OE1	GLU	6	33.746	0.800	14.570	1.00	0.00	O
ATOM	83	OE2	GLU	6	35.180	1.844	13.272	1.00	0.00	O
ATOM	84	H	GLU	6	34.012	5.424	14.622	1.00	0.00	H
ATOM	85	HA	GLU	6	32.452	3.360	14.033	1.00	0.00	H
ATOM	86	2HB3	GLU	6	32.862	2.457	16.370	1.00	0.00	H
ATOM	87	2HG3	GLU	6	35.304	2.406	16.199	1.00	0.00	H
ATOM	88	HB2	GLU	6	33.638	4.019	16.740	1.00	0.00	H
ATOM	89	HG2	GLU	6	35.240	3.643	14.922	1.00	0.00	H
ATOM	90	N	GLU	7	30.857	5.229	16.199	1.00	0.00	N
ATOM	91	CA	GLU	7	29.551	5.497	16.787	1.00	0.00	C
ATOM	92	C	GLU	7	28.459	5.473	15.723	1.00	0.00	C
ATOM	93	O	GLU	7	27.475	4.744	15.846	1.00	0.00	O
ATOM	94	CB	GLU	7	29.558	6.855	17.498	1.00	0.00	C
ATOM	95	CG	GLU	7	30.560	7.839	16.921	1.00	0.00	C
ATOM	96	CD	GLU	7	31.904	7.777	17.618	1.00	0.00	C
ATOM	97	OE1	GLU	7	32.722	6.905	17.255	1.00	0.00	O
ATOM	98	OE2	GLU	7	32.139	8.601	18.529	1.00	0.00	O
ATOM	99	H	GLU	7	31.588	5.867	16.343	1.00	0.00	H
ATOM	100	HA	GLU	7	29.347	4.723	17.513	1.00	0.00	H
ATOM	101	2HB3	GLU	7	29.795	6.699	18.540	1.00	0.00	H
ATOM	102	2HG3	GLU	7	30.162	8.838	17.023	1.00	0.00	H
ATOM	103	HB2	GLU	7	28.578	7.323	17.396	1.00	0.00	H
ATOM	104	HG2	GLU	7	30.733	7.608	15.870	1.00	0.00	H
ATOM	105	N	GLN	8	28.639	6.278	14.680	1.00	0.00	N
ATOM	106	CA	GLN	8	27.668	6.349	13.595	1.00	0.00	C
ATOM	107	C	GLN	8	27.825	5.165	12.644	1.00	0.00	C
ATOM	108	O	GLN	8	26.844	4.525	12.268	1.00	0.00	O
ATOM	109	CB	GLN	8	27.827	7.663	12.824	1.00	0.00	C
ATOM	110	CG	GLN	8	27.441	8.891	13.631	1.00	0.00	C
ATOM	111	CD	GLN	8	25.957	8.949	13.931	1.00	0.00	C
ATOM	112	OE1	GLN	8	25.180	9.526	13.170	1.00	0.00	O
ATOM	113	NE2	GLN	8	25.554	8.349	15.046	1.00	0.00	N
ATOM	114	H	GLN	8	29.443	6.834	14.639	1.00	0.00	H
ATOM	115	HA	GLN	8	26.681	6.316	14.031	1.00	0.00	H
ATOM	116	2HB3	GLN	8	27.205	7.626	11.942	1.00	0.00	H
ATOM	117	2HG3	GLN	8	27.716	9.773	13.071	1.00	0.00	H
ATOM	118	1HE2	GLN	8	26.229	7.909	15.603	1.00	0.00	H
ATOM	119	2HE2	GLN	8	24.600	8.371	15.265	1.00	0.00	H
ATOM	120	HB2	GLN	8	28.873	7.796	12.544	1.00	0.00	H
ATOM	121	HG2	GLN	8	27.960	8.874	14.590	1.00	0.00	H
ATOM	122	N	ILE	9	29.064	4.883	12.259	1.00	0.00	N
ATOM	123	CA	ILE	9	29.350	3.778	11.352	1.00	0.00	C
ATOM	124	C	ILE	9	28.551	2.536	11.734	1.00	0.00	C
ATOM	125	O	ILE	9	28.002	1.851	10.871	1.00	0.00	O
ATOM	126	CB	ILE	9	30.850	3.428	11.347	1.00	0.00	C
ATOM	127	CG2	ILE	9	31.110	2.231	10.445	1.00	0.00	C
ATOM	128	CG1	ILE	9	31.676	4.634	10.894	1.00	0.00	C
ATOM	129	CD1	ILE	9	33.165	4.448	11.080	1.00	0.00	C
ATOM	130	H	ILE	9	29.806	5.432	12.591	1.00	0.00	H
ATOM	131	HA	ILE	9	29.069	4.083	10.356	1.00	0.00	H
ATOM	132	HB	ILE	9	31.138	3.159	12.352	1.00	0.00	H
ATOM	133	1HG2	ILE	9	32.037	1.759	10.733	1.00	0.00	H
ATOM	134	2HG2	ILE	9	30.301	1.524	10.542	1.00	0.00	H

ATOM	135	3HG2	ILE	9	31.178	2.561	9.419	1.00	0.00	H
ATOM	136	2HG3	ILE	9	31.375	5.501	11.464	1.00	0.00	H
ATOM	137	1HD1	ILE	9	33.680	5.356	10.797	1.00	0.00	H
ATOM	138	2HD1	ILE	9	33.375	4.224	12.116	1.00	0.00	H
ATOM	139	3HD1	ILE	9	33.508	3.633	10.460	1.00	0.00	H
ATOM	140	HG2	ILE	9	31.508	4.811	9.832	1.00	0.00	H
ATOM	141	N	ALA	10	28.489	2.254	13.032	1.00	0.00	N
ATOM	142	CA	ALA	10	27.752	1.097	13.527	1.00	0.00	C
ATOM	143	C	ALA	10	26.266	1.211	13.201	1.00	0.00	C
ATOM	144	O	ALA	10	25.641	0.242	12.775	1.00	0.00	O
ATOM	145	CB	ALA	10	27.957	0.945	15.026	1.00	0.00	C
ATOM	146	H	ALA	10	28.947	2.839	13.669	1.00	0.00	H
ATOM	147	HA	ALA	10	28.150	0.216	13.043	1.00	0.00	H
ATOM	148	1HB	ALA	10	28.589	1.745	15.385	1.00	0.00	H
ATOM	149	2HB	ALA	10	27.001	0.989	15.526	1.00	0.00	H
ATOM	150	3HB	ALA	10	28.425	-0.005	15.230	1.00	0.00	H
ATOM	151	N	GLU	11	25.711	2.401	13.406	1.00	0.00	N
ATOM	152	CA	GLU	11	24.298	2.640	13.135	1.00	0.00	C
ATOM	153	C	GLU	11	23.996	2.488	11.648	1.00	0.00	C
ATOM	154	O	GLU	11	23.146	1.686	11.254	1.00	0.00	O
ATOM	155	CB	GLU	11	23.893	4.037	13.609	1.00	0.00	C
ATOM	156	CG	GLU	11	23.565	4.105	15.091	1.00	0.00	C
ATOM	157	CD	GLU	11	22.191	3.550	15.410	1.00	0.00	C
ATOM	158	OE1	GLU	11	22.081	2.326	15.625	1.00	0.00	O
ATOM	159	OE2	GLU	11	21.224	4.340	15.445	1.00	0.00	O
ATOM	160	H	GLU	11	26.264	3.136	13.747	1.00	0.00	H
ATOM	161	HA	GLU	11	23.726	1.905	13.684	1.00	0.00	H
ATOM	162	2HB3	GLU	11	23.023	4.353	13.054	1.00	0.00	H
ATOM	163	2HG3	GLU	11	23.601	5.137	15.408	1.00	0.00	H
ATOM	164	HB2	GLU	11	24.720	4.730	13.448	1.00	0.00	H
ATOM	165	HG2	GLU	11	24.291	3.515	15.652	1.00	0.00	H
ATOM	166	N	PHE	12	24.697	3.262	10.825	1.00	0.00	N
ATOM	167	CA	PHE	12	24.502	3.214	9.381	1.00	0.00	C
ATOM	168	C	PHE	12	24.769	1.812	8.841	1.00	0.00	C
ATOM	169	O	PHE	12	24.206	1.410	7.823	1.00	0.00	O
ATOM	170	CB	PHE	12	25.422	4.223	8.689	1.00	0.00	C
ATOM	171	CG	PHE	12	24.917	5.635	8.749	1.00	0.00	C
ATOM	172	CD1	PHE	12	24.084	6.129	7.755	1.00	0.00	C
ATOM	173	CE1	PHE	12	23.618	7.431	7.808	1.00	0.00	C
ATOM	174	CZ	PHE	12	23.980	8.251	8.859	1.00	0.00	C
ATOM	175	CE2	PHE	12	24.807	7.770	9.853	1.00	0.00	C
ATOM	176	CD2	PHE	12	25.272	6.470	9.796	1.00	0.00	C
ATOM	177	H	PHE	12	25.359	3.880	11.200	1.00	0.00	H
ATOM	178	HA	PHE	12	23.476	3.478	9.177	1.00	0.00	H
ATOM	179	2HB3	PHE	12	25.527	3.949	7.650	1.00	0.00	H
ATOM	180	1HD	PHE	12	23.801	5.488	6.934	1.00	0.00	H
ATOM	181	1HE	PHE	12	22.971	7.803	7.029	1.00	0.00	H
ATOM	182	HZ	PHE	12	23.616	9.266	8.901	1.00	0.00	H
ATOM	183	2HE	PHE	12	25.091	8.409	10.677	1.00	0.00	H
ATOM	184	2HD	PHE	12	25.919	6.093	10.576	1.00	0.00	H
ATOM	185	HB2	PHE	12	26.397	4.219	9.178	1.00	0.00	H
ATOM	186	N	LYS	13	25.629	1.072	9.532	1.00	0.00	N
ATOM	187	CA	LYS	13	25.971	-0.285	9.125	1.00	0.00	C
ATOM	188	C	LYS	13	24.713	-1.113	8.879	1.00	0.00	C
ATOM	189	O	LYS	13	24.532	-1.676	7.801	1.00	0.00	O
ATOM	190	CB	LYS	13	26.836	-0.957	10.193	1.00	0.00	C
ATOM	191	CG	LYS	13	27.469	-2.259	9.732	1.00	0.00	C
ATOM	192	CD	LYS	13	28.691	-2.611	10.565	1.00	0.00	C
ATOM	193	CE	LYS	13	28.300	-3.265	11.879	1.00	0.00	C
ATOM	194	NZ	LYS	13	29.479	-3.469	12.768	1.00	0.00	N
ATOM	195	H	LYS	13	26.046	1.450	10.337	1.00	0.00	H
ATOM	196	HA	LYS	13	26.531	-0.224	8.205	1.00	0.00	H
ATOM	197	2HB3	LYS	13	26.223	-1.164	11.058	1.00	0.00	H
ATOM	198	2HG3	LYS	13	27.765	-2.160	8.699	1.00	0.00	H
ATOM	199	2HD3	LYS	13	29.245	-1.705	10.773	1.00	0.00	H
ATOM	200	2HE3	LYS	13	27.849	-4.224	11.669	1.00	0.00	H
ATOM	201	1HZ	LYS	13	30.134	-2.666	12.683	1.00	0.00	H
ATOM	202	2HZ	LYS	13	29.979	-4.341	12.503	1.00	0.00	H
ATOM	203	3HZ	LYS	13	29.170	-3.549	13.758	1.00	0.00	H
ATOM	204	HB2	LYS	13	27.661	-0.296	10.463	1.00	0.00	H
ATOM	205	HG2	LYS	13	26.749	-3.070	9.837	1.00	0.00	H
ATOM	206	HD2	LYS	13	29.317	-3.311	10.012	1.00	0.00	H
ATOM	207	HE2	LYS	13	27.595	-2.626	12.409	1.00	0.00	H
ATOM	208	N	GLU	14	23.848	-1.178	9.888	1.00	0.00	N
ATOM	209	CA	GLU	14	22.607	-1.936	9.778	1.00	0.00	C
ATOM	210	C	GLU	14	21.827	-1.530	8.532	1.00	0.00	C
ATOM	211	O	GLU	14	21.323	-2.379	7.797	1.00	0.00	O

ATOM	212	CB	GLU	14	21.743	-1.724	11.025	1.00	0.00	C
ATOM	213	CG	GLU	14	22.437	-2.110	12.320	1.00	0.00	C
ATOM	214	CD	GLU	14	22.869	-3.565	12.338	1.00	0.00	C
ATOM	215	OE1	GLU	14	22.042	-4.433	11.995	1.00	0.00	O
ATOM	216	OE2	GLU	14	24.035	-3.830	12.696	1.00	0.00	O
ATOM	217	H	GLU	14	24.048	-0.705	10.722	1.00	0.00	H
ATOM	218	HA	GLU	14	22.862	-2.983	9.704	1.00	0.00	H
ATOM	219	2HB3	GLU	14	20.847	-2.318	10.930	1.00	0.00	H
ATOM	220	2HG3	GLU	14	21.757	-1.941	13.142	1.00	0.00	H
ATOM	221	HB2	GLU	14	21.491	-0.666	11.117	1.00	0.00	H
ATOM	222	HG2	GLU	14	23.336	-1.504	12.445	1.00	0.00	H
ATOM	223	N	ALA	15	21.735	-0.224	8.299	1.00	0.00	N
ATOM	224	CA	ALA	15	21.018	0.296	7.140	1.00	0.00	C
ATOM	225	C	ALA	15	21.733	-0.071	5.844	1.00	0.00	C
ATOM	226	O	ALA	15	21.164	0.037	4.757	1.00	0.00	O
ATOM	227	CB	ALA	15	20.861	1.804	7.249	1.00	0.00	C
ATOM	228	H	ALA	15	22.158	0.404	8.918	1.00	0.00	H
ATOM	229	HA	ALA	15	20.032	-0.146	7.131	1.00	0.00	H
ATOM	230	1HB	ALA	15	20.356	2.178	6.370	1.00	0.00	H
ATOM	231	2HB	ALA	15	20.281	2.042	8.129	1.00	0.00	H
ATOM	232	3HB	ALA	15	21.836	2.263	7.325	1.00	0.00	H
ATOM	233	N	PHE	16	22.983	-0.505	5.964	1.00	0.00	N
ATOM	234	CA	PHE	16	23.777	-0.886	4.801	1.00	0.00	C
ATOM	235	C	PHE	16	23.979	-2.397	4.752	1.00	0.00	C
ATOM	236	O	PHE	16	24.518	-2.932	3.783	1.00	0.00	O
ATOM	237	CB	PHE	16	25.133	-0.179	4.829	1.00	0.00	C
ATOM	238	CG	PHE	16	25.998	-0.492	3.641	1.00	0.00	C
ATOM	239	CD1	PHE	16	25.835	0.199	2.450	1.00	0.00	C
ATOM	240	CE1	PHE	16	26.628	-0.090	1.354	1.00	0.00	C
ATOM	241	CZ	PHE	16	27.596	-1.071	1.443	1.00	0.00	C
ATOM	242	CE2	PHE	16	27.767	-1.765	2.623	1.00	0.00	C
ATOM	243	CD2	PHE	16	26.972	-1.475	3.716	1.00	0.00	C
ATOM	244	H	PHE	16	23.382	-0.570	6.858	1.00	0.00	H
ATOM	245	HA	PHE	16	23.237	-0.578	3.917	1.00	0.00	H
ATOM	246	2HB3	PHE	16	25.669	-0.477	5.718	1.00	0.00	H
ATOM	247	1HD	PHE	16	25.081	0.967	2.381	1.00	0.00	H
ATOM	248	1HE	PHE	16	26.493	0.454	0.431	1.00	0.00	H
ATOM	249	HZ	PHE	16	28.216	-1.297	0.587	1.00	0.00	H
ATOM	250	2HE	PHE	16	28.522	-2.534	2.696	1.00	0.00	H
ATOM	251	2HD	PHE	16	27.104	-2.019	4.639	1.00	0.00	H
ATOM	252	HB2	PHE	16	24.982	0.900	4.828	1.00	0.00	H
ATOM	253	N	SER	17	23.545	-3.081	5.806	1.00	0.00	N
ATOM	254	CA	SER	17	23.684	-4.531	5.887	1.00	0.00	C
ATOM	255	C	SER	17	22.461	-5.225	5.294	1.00	0.00	C
ATOM	256	O	SER	17	22.563	-6.322	4.742	1.00	0.00	O
ATOM	257	CB	SER	17	23.878	-4.967	7.342	1.00	0.00	C
ATOM	258	OG	SER	17	25.252	-4.993	7.687	1.00	0.00	O
ATOM	259	H	SER	17	23.127	-2.598	6.551	1.00	0.00	H
ATOM	260	HA	SER	17	24.555	-4.813	5.317	1.00	0.00	H
ATOM	261	2HB3	SER	17	23.465	-5.954	7.476	1.00	0.00	H
ATOM	262	HG	SER	17	25.673	-5.748	7.266	1.00	0.00	H
ATOM	263	HB2	SER	17	23.369	-4.265	8.003	1.00	0.00	H
ATOM	264	N	LEU	18	21.308	-4.581	5.415	1.00	0.00	N
ATOM	265	CA	LEU	18	20.063	-5.136	4.891	1.00	0.00	C
ATOM	266	C	LEU	18	20.016	-5.030	3.371	1.00	0.00	C
ATOM	267	O	LEU	18	19.550	-5.943	2.689	1.00	0.00	O
ATOM	268	CB	LEU	18	18.861	-4.412	5.504	1.00	0.00	C
ATOM	269	CG	LEU	18	18.397	-3.151	4.773	1.00	0.00	C
ATOM	270	CD1	LEU	18	16.920	-2.894	5.039	1.00	0.00	C
ATOM	271	CD2	LEU	18	19.231	-1.950	5.193	1.00	0.00	C
ATOM	272	H	LEU	18	21.287	-3.711	5.867	1.00	0.00	H
ATOM	273	HA	LEU	18	20.024	-6.179	5.170	1.00	0.00	H
ATOM	274	2HB3	LEU	18	19.123	-4.131	6.513	1.00	0.00	H
ATOM	275	HG	LEU	18	18.523	-3.291	3.708	1.00	0.00	H
ATOM	276	1HD1	LEU	18	16.785	-2.610	6.071	1.00	0.00	H
ATOM	277	2HD1	LEU	18	16.356	-3.793	4.835	1.00	0.00	H
ATOM	278	3HD1	LEU	18	16.573	-2.098	4.397	1.00	0.00	H
ATOM	279	1HD2	LEU	18	18.647	-1.050	5.086	1.00	0.00	H
ATOM	280	2HD2	LEU	18	20.108	-1.887	4.566	1.00	0.00	H
ATOM	281	3HD2	LEU	18	19.533	-2.065	6.223	1.00	0.00	H
ATOM	282	HB2	LEU	18	17.993	-5.072	5.492	1.00	0.00	H
ATOM	283	N	PHE	19	20.502	-3.911	2.846	1.00	0.00	N
ATOM	284	CA	PHE	19	20.520	-3.685	1.407	1.00	0.00	C
ATOM	285	C	PHE	19	21.480	-4.649	0.714	1.00	0.00	C
ATOM	286	O	PHE	19	21.138	-5.268	-0.291	1.00	0.00	O
ATOM	287	CB	PHE	19	20.919	-2.240	1.098	1.00	0.00	C
ATOM	288	CG	PHE	19	19.746	-1.308	0.982	1.00	0.00	C

ATOM	289	CD1	PHE	19	18.913	-1.356	-0.125	1.00	0.00	C
ATOM	290	CE1	PHE	19	17.835	-0.498	-0.235	1.00	0.00	C
ATOM	291	CZ	PHE	19	17.580	0.419	0.765	1.00	0.00	C
ATOM	292	CE2	PHE	19	18.401	0.476	1.872	1.00	0.00	C
ATOM	293	CD2	PHE	19	19.480	-0.383	1.978	1.00	0.00	C
ATOM	294	H	PHE	19	20.862	-3.218	3.443	1.00	0.00	H
ATOM	295	HA	PHE	19	19.522	-3.862	1.033	1.00	0.00	H
ATOM	296	2HB3	PHE	19	21.458	-2.215	0.165	1.00	0.00	H
ATOM	297	1HD	PHE	19	19.113	-2.074	-0.908	1.00	0.00	H
ATOM	298	1HE	PHE	19	17.195	-0.547	-1.102	1.00	0.00	H
ATOM	299	HZ	PHE	19	16.736	1.088	0.680	1.00	0.00	H
ATOM	300	2HE	PHE	19	18.204	1.195	2.655	1.00	0.00	H
ATOM	301	2HD	PHE	19	20.121	-0.335	2.845	1.00	0.00	H
ATOM	302	HB2	PHE	19	21.544	-1.857	1.903	1.00	0.00	H
ATOM	303	N	ASP	20	22.684	-4.768	1.265	1.00	0.00	N
ATOM	304	CA	ASP	20	23.696	-5.655	0.700	1.00	0.00	C
ATOM	305	C	ASP	20	23.301	-7.116	0.887	1.00	0.00	C
ATOM	306	O	ASP	20	23.650	-7.743	1.887	1.00	0.00	O
ATOM	307	CB	ASP	20	25.052	-5.393	1.355	1.00	0.00	C
ATOM	308	CG	ASP	20	26.102	-6.404	0.936	1.00	0.00	C
ATOM	309	OD1	ASP	20	26.169	-6.723	-0.270	1.00	0.00	O
ATOM	310	OD2	ASP	20	26.855	-6.877	1.812	1.00	0.00	O
ATOM	311	H	ASP	20	22.897	-4.248	2.067	1.00	0.00	H
ATOM	312	HA	ASP	20	23.769	-5.445	-0.354	1.00	0.00	H
ATOM	313	2HB3	ASP	20	24.943	-5.439	2.429	1.00	0.00	H
ATOM	314	HB2	ASP	20	25.414	-4.408	1.061	1.00	0.00	H
ATOM	315	N	LYS	21	22.570	-7.654	-0.084	1.00	0.00	N
ATOM	316	CA	LYS	21	22.125	-9.042	-0.028	1.00	0.00	C
ATOM	317	C	LYS	21	23.275	-9.993	-0.351	1.00	0.00	C
ATOM	318	O	LYS	21	23.420	-11.040	0.277	1.00	0.00	O
ATOM	319	CB	LYS	21	20.972	-9.269	-1.007	1.00	0.00	C
ATOM	320	CG	LYS	21	19.791	-8.339	-0.782	1.00	0.00	C
ATOM	321	CD	LYS	21	19.080	-8.649	0.525	1.00	0.00	C
ATOM	322	CE	LYS	21	17.606	-8.281	0.458	1.00	0.00	C
ATOM	323	NZ	LYS	21	16.832	-8.886	1.577	1.00	0.00	N
ATOM	324	H	LYS	21	22.323	-7.104	-0.857	1.00	0.00	H
ATOM	325	HA	LYS	21	21.781	-9.241	0.975	1.00	0.00	H
ATOM	326	2HB3	LYS	21	20.625	-10.288	-0.906	1.00	0.00	H
ATOM	327	2HG3	LYS	21	19.092	-8.455	-1.598	1.00	0.00	H
ATOM	328	2HD3	LYS	21	19.548	-8.085	1.320	1.00	0.00	H
ATOM	329	2HE3	LYS	21	17.203	-8.632	-0.480	1.00	0.00	H
ATOM	330	1HZ	LYS	21	17.472	-9.141	2.356	1.00	0.00	H
ATOM	331	2HZ	LYS	21	16.343	-9.743	1.248	1.00	0.00	H
ATOM	332	3HZ	LYS	21	16.124	-8.211	1.929	1.00	0.00	H
ATOM	333	HB2	LYS	21	21.316	-9.084	-2.025	1.00	0.00	H
ATOM	334	HG2	LYS	21	20.144	-7.309	-0.738	1.00	0.00	H
ATOM	335	HD2	LYS	21	19.149	-9.716	0.734	1.00	0.00	H
ATOM	336	HE2	LYS	21	17.496	-7.200	0.535	1.00	0.00	H
ATOM	337	N	ASP	22	24.087	-9.621	-1.334	1.00	0.00	N
ATOM	338	CA	ASP	22	25.224	-10.440	-1.737	1.00	0.00	C
ATOM	339	C	ASP	22	26.145	-10.714	-0.552	1.00	0.00	C
ATOM	340	O	ASP	22	26.621	-11.832	-0.368	1.00	0.00	O
ATOM	341	CB	ASP	22	26.004	-9.750	-2.857	1.00	0.00	C
ATOM	342	CG	ASP	22	25.097	-9.179	-3.928	1.00	0.00	C
ATOM	343	OD1	ASP	22	24.018	-9.760	-4.164	1.00	0.00	O
ATOM	344	OD2	ASP	22	25.466	-8.149	-4.533	1.00	0.00	O
ATOM	345	H	ASP	22	23.918	-8.774	-1.797	1.00	0.00	H
ATOM	346	HA	ASP	22	24.841	-11.379	-2.105	1.00	0.00	H
ATOM	347	2HB3	ASP	22	26.670	-10.464	-3.317	1.00	0.00	H
ATOM	348	HB2	ASP	22	26.578	-8.920	-2.443	1.00	0.00	H
ATOM	349	N	GLY	23	26.391	-9.682	0.250	1.00	0.00	N
ATOM	350	CA	GLY	23	27.256	-9.832	1.406	1.00	0.00	C
ATOM	351	C	GLY	23	28.710	-9.545	1.086	1.00	0.00	C
ATOM	352	O	GLY	23	29.596	-10.314	1.455	1.00	0.00	O
ATOM	353	H	GLY	23	25.983	-8.812	0.055	1.00	0.00	H
ATOM	354	2HA	GLY	23	26.927	-9.151	2.178	1.00	0.00	H
ATOM	355	3HA	GLY	23	27.174	-10.843	1.774	1.00	0.00	H
ATOM	356	N	ASP	24	28.953	-8.436	0.396	1.00	0.00	N
ATOM	357	CA	ASP	24	30.307	-8.050	0.023	1.00	0.00	C
ATOM	358	C	ASP	24	30.632	-6.647	0.530	1.00	0.00	C
ATOM	359	O	ASP	24	31.698	-6.410	1.093	1.00	0.00	O
ATOM	360	CB	ASP	24	30.482	-8.110	-1.495	1.00	0.00	C
ATOM	361	CG	ASP	24	29.479	-7.240	-2.226	1.00	0.00	C
ATOM	362	OD1	ASP	24	28.271	-7.352	-1.932	1.00	0.00	O
ATOM	363	OD2	ASP	24	29.902	-6.447	-3.094	1.00	0.00	O
ATOM	364	H	ASP	24	28.202	-7.864	0.129	1.00	0.00	H
ATOM	365	HA	ASP	24	30.991	-8.750	0.483	1.00	0.00	H

ATOM	366	2HB3	ASP	24	30.354	-9.129	-1.825	1.00	0.00	H
ATOM	367	HB2	ASP	24	31.477	-7.756	-1.761	1.00	0.00	H
ATOM	368	N	GLY	25	29.702	-5.720	0.324	1.00	0.00	N
ATOM	369	CA	GLY	25	29.906	-4.352	0.764	1.00	0.00	C
ATOM	370	C	GLY	25	29.521	-3.338	-0.296	1.00	0.00	C
ATOM	371	O	GLY	25	30.077	-2.241	-0.348	1.00	0.00	O
ATOM	372	H	GLY	25	28.870	-5.968	-0.133	1.00	0.00	H
ATOM	373	2HA	GLY	25	29.314	-4.177	1.647	1.00	0.00	H
ATOM	374	3HA	GLY	25	30.950	-4.218	1.009	1.00	0.00	H
ATOM	375	N	THR	26	28.565	-3.706	-1.143	1.00	0.00	N
ATOM	376	CA	THR	26	28.107	-2.823	-2.209	1.00	0.00	C
ATOM	377	C	THR	26	26.610	-2.980	-2.448	1.00	0.00	C
ATOM	378	O	THR	26	25.979	-3.893	-1.912	1.00	0.00	O
ATOM	379	CB	THR	26	28.857	-3.093	-3.526	1.00	0.00	C
ATOM	380	OG1	THR	26	28.628	-4.441	-3.951	1.00	0.00	O
ATOM	381	CG2	THR	26	30.350	-2.854	-3.356	1.00	0.00	C
ATOM	382	H	THR	26	28.160	-4.593	-1.051	1.00	0.00	H
ATOM	383	HA	THR	26	28.308	-1.803	-1.907	1.00	0.00	H
ATOM	384	HB	THR	26	28.483	-2.417	-4.281	1.00	0.00	H
ATOM	385	1HG	THR	26	29.228	-4.655	-4.670	1.00	0.00	H
ATOM	386	1HG2	THR	26	30.894	-3.468	-4.059	1.00	0.00	H
ATOM	387	2HG2	THR	26	30.644	-3.112	-2.350	1.00	0.00	H
ATOM	388	3HG2	THR	26	30.570	-1.814	-3.542	1.00	0.00	H
ATOM	389	N	ILE	27	26.047	-2.087	-3.255	1.00	0.00	N
ATOM	390	CA	ILE	27	24.624	-2.129	-3.566	1.00	0.00	C
ATOM	391	C	ILE	27	24.373	-1.823	-5.039	1.00	0.00	C
ATOM	392	O	ILE	27	24.961	-0.898	-5.600	1.00	0.00	O
ATOM	393	CB	ILE	27	23.827	-1.132	-2.703	1.00	0.00	C
ATOM	394	CG2	ILE	27	22.337	-1.262	-2.982	1.00	0.00	C
ATOM	395	CG1	ILE	27	24.120	-1.363	-1.220	1.00	0.00	C
ATOM	396	CD1	ILE	27	23.853	-0.150	-0.355	1.00	0.00	C
ATOM	397	H	ILE	27	26.601	-1.384	-3.652	1.00	0.00	H
ATOM	398	HA	ILE	27	24.266	-3.127	-3.351	1.00	0.00	H
ATOM	399	HB	ILE	27	24.135	-0.133	-2.972	1.00	0.00	H
ATOM	400	1HG2	ILE	27	21.849	-1.711	-2.131	1.00	0.00	H
ATOM	401	2HG2	ILE	27	21.919	-0.281	-3.159	1.00	0.00	H
ATOM	402	3HG2	ILE	27	22.186	-1.880	-3.853	1.00	0.00	H
ATOM	403	2HG3	ILE	27	25.158	-1.634	-1.103	1.00	0.00	H
ATOM	404	1HD1	ILE	27	24.447	0.680	-0.708	1.00	0.00	H
ATOM	405	2HD1	ILE	27	22.807	0.107	-0.406	1.00	0.00	H
ATOM	406	3HD1	ILE	27	24.119	-0.372	0.669	1.00	0.00	H
ATOM	407	HG2	ILE	27	23.481	-2.163	-0.843	1.00	0.00	H
ATOM	408	N	THR	28	23.494	-2.604	-5.659	1.00	0.00	N
ATOM	409	CA	THR	28	23.165	-2.415	-7.067	1.00	0.00	C
ATOM	410	C	THR	28	21.658	-2.474	-7.291	1.00	0.00	C
ATOM	411	O	THR	28	20.890	-2.734	-6.364	1.00	0.00	O
ATOM	412	CB	THR	28	23.845	-3.478	-7.950	1.00	0.00	C
ATOM	413	OG1	THR	28	23.587	-3.204	-9.331	1.00	0.00	O
ATOM	414	CG2	THR	28	23.347	-4.872	-7.602	1.00	0.00	C
ATOM	415	H	THR	28	23.057	-3.324	-5.159	1.00	0.00	H
ATOM	416	HA	THR	28	23.526	-1.442	-7.366	1.00	0.00	H
ATOM	417	HB	THR	28	24.912	-3.438	-7.776	1.00	0.00	H
ATOM	418	1HG	THR	28	23.945	-3.914	-9.872	1.00	0.00	H
ATOM	419	1HG2	THR	28	23.880	-5.239	-6.738	1.00	0.00	H
ATOM	420	2HG2	THR	28	23.514	-5.533	-8.438	1.00	0.00	H
ATOM	421	3HG2	THR	28	22.291	-4.831	-7.383	1.00	0.00	H
ATOM	422	N	THR	29	21.239	-2.229	-8.529	1.00	0.00	N
ATOM	423	CA	THR	29	19.824	-2.254	-8.877	1.00	0.00	C
ATOM	424	C	THR	29	19.147	-3.506	-8.334	1.00	0.00	C
ATOM	425	O	THR	29	17.995	-3.464	-7.895	1.00	0.00	O
ATOM	426	CB	THR	29	19.617	-2.192	-10.402	1.00	0.00	C
ATOM	427	OG1	THR	29	20.093	-3.398	-11.009	1.00	0.00	O
ATOM	428	CG2	THR	29	20.344	-0.996	-10.998	1.00	0.00	C
ATOM	429	H	THR	29	21.899	-2.026	-9.226	1.00	0.00	H
ATOM	430	HA	THR	29	19.358	-1.384	-8.435	1.00	0.00	H
ATOM	431	HB	THR	29	18.559	-2.090	-10.601	1.00	0.00	H
ATOM	432	1HG	THR	29	19.951	-4.136	-10.411	1.00	0.00	H
ATOM	433	1HG2	THR	29	20.120	-0.928	-12.052	1.00	0.00	H
ATOM	434	2HG2	THR	29	21.408	-1.119	-10.864	1.00	0.00	H
ATOM	435	3HG2	THR	29	20.020	-0.094	-10.501	1.00	0.00	H
ATOM	436	N	LYS	30	19.867	-4.622	-8.364	1.00	0.00	N
ATOM	437	CA	LYS	30	19.337	-5.889	-7.874	1.00	0.00	C
ATOM	438	C	LYS	30	19.014	-5.804	-6.384	1.00	0.00	C
ATOM	439	O	LYS	30	17.949	-6.237	-5.945	1.00	0.00	O
ATOM	440	CB	LYS	30	20.339	-7.018	-8.125	1.00	0.00	C
ATOM	441	CG	LYS	30	19.927	-8.345	-7.513	1.00	0.00	C
ATOM	442	CD	LYS	30	18.899	-9.062	-8.371	1.00	0.00	C

ATOM	443	CE	LYS	30	19.561	-9.853	-9.489	1.00	0.00	C
ATOM	444	NZ	LYS	30	18.576	-10.290	-10.517	1.00	0.00	N
ATOM	445	H	LYS	30	20.778	-4.594	-8.726	1.00	0.00	H
ATOM	446	HA	LYS	30	18.427	-6.101	-8.414	1.00	0.00	H
ATOM	447	2HB3	LYS	30	21.294	-6.733	-7.707	1.00	0.00	H
ATOM	448	2HG3	LYS	30	19.504	-8.164	-6.534	1.00	0.00	H
ATOM	449	2HD3	LYS	30	18.233	-8.329	-8.806	1.00	0.00	H
ATOM	450	2HE3	LYS	30	20.036	-10.724	-9.064	1.00	0.00	H
ATOM	451	1HZ	LYS	30	18.990	-10.206	-11.467	1.00	0.00	H
ATOM	452	2HZ	LYS	30	17.723	-9.701	-10.468	1.00	0.00	H
ATOM	453	3HZ	LYS	30	18.307	-11.282	-10.356	1.00	0.00	H
ATOM	454	HB2	LYS	30	20.430	-7.192	-9.197	1.00	0.00	H
ATOM	455	HG2	LYS	30	20.801	-8.992	-7.426	1.00	0.00	H
ATOM	456	HD2	LYS	30	18.334	-9.759	-7.755	1.00	0.00	H
ATOM	457	HE2	LYS	30	20.300	-9.227	-9.990	1.00	0.00	H
ATOM	458	N	GLU	31	19.941	-5.242	-5.615	1.00	0.00	N
ATOM	459	CA	GLU	31	19.753	-5.101	-4.175	1.00	0.00	C
ATOM	460	C	GLU	31	18.846	-3.916	-3.858	1.00	0.00	C
ATOM	461	O	GLU	31	17.751	-4.084	-3.316	1.00	0.00	O
ATOM	462	CB	GLU	31	21.103	-4.921	-3.478	1.00	0.00	C
ATOM	463	CG	GLU	31	21.995	-6.150	-3.554	1.00	0.00	C
ATOM	464	CD	GLU	31	23.471	-5.801	-3.516	1.00	0.00	C
ATOM	465	OE1	GLU	31	23.996	-5.329	-4.546	1.00	0.00	O
ATOM	466	OE2	GLU	31	24.101	-6.002	-2.457	1.00	0.00	O
ATOM	467	H	GLU	31	20.769	-4.917	-6.024	1.00	0.00	H
ATOM	468	HA	GLU	31	19.287	-6.003	-3.810	1.00	0.00	H
ATOM	469	2HB3	GLU	31	20.931	-4.694	-2.437	1.00	0.00	H
ATOM	470	2HG3	GLU	31	21.789	-6.672	-4.479	1.00	0.00	H
ATOM	471	HB2	GLU	31	21.655	-4.111	-3.955	1.00	0.00	H
ATOM	472	HG2	GLU	31	21.794	-6.800	-2.702	1.00	0.00	H
ATOM	473	N	LEU	32	19.303	-2.717	-4.201	1.00	0.00	N
ATOM	474	CA	LEU	32	18.533	-1.503	-3.954	1.00	0.00	C
ATOM	475	C	LEU	32	17.110	-1.645	-4.486	1.00	0.00	C
ATOM	476	O	LEU	32	16.154	-1.200	-3.852	1.00	0.00	O
ATOM	477	CB	LEU	32	19.217	-0.302	-4.608	1.00	0.00	C
ATOM	478	CG	LEU	32	18.479	1.033	-4.494	1.00	0.00	C
ATOM	479	CD1	LEU	32	18.397	1.476	-3.043	1.00	0.00	C
ATOM	480	CD2	LEU	32	19.165	2.097	-5.340	1.00	0.00	C
ATOM	481	H	LEU	32	20.180	-2.646	-4.633	1.00	0.00	H
ATOM	482	HA	LEU	32	18.493	-1.349	-2.887	1.00	0.00	H
ATOM	483	2HB3	LEU	32	19.341	-0.523	-5.660	1.00	0.00	H
ATOM	484	HG	LEU	32	17.470	0.912	-4.862	1.00	0.00	H
ATOM	485	1HD1	LEU	32	18.405	0.607	-2.401	1.00	0.00	H
ATOM	486	2HD1	LEU	32	17.484	2.032	-2.885	1.00	0.00	H
ATOM	487	3HD1	LEU	32	19.245	2.104	-2.808	1.00	0.00	H
ATOM	488	1HD2	LEU	32	19.995	2.516	-4.788	1.00	0.00	H
ATOM	489	2HD2	LEU	32	18.459	2.879	-5.576	1.00	0.00	H
ATOM	490	3HD2	LEU	32	19.530	1.649	-6.252	1.00	0.00	H
ATOM	491	HB2	LEU	32	20.180	-0.124	-4.128	1.00	0.00	H
ATOM	492	N	GLY	33	16.977	-2.270	-5.650	1.00	0.00	N
ATOM	493	CA	GLY	33	15.667	-2.459	-6.246	1.00	0.00	C
ATOM	494	C	GLY	33	14.835	-3.481	-5.497	1.00	0.00	C
ATOM	495	O	GLY	33	13.612	-3.523	-5.641	1.00	0.00	O
ATOM	496	H	GLY	33	17.775	-2.603	-6.112	1.00	0.00	H
ATOM	497	2HA	GLY	33	15.144	-1.515	-6.246	1.00	0.00	H
ATOM	498	3HA	GLY	33	15.792	-2.792	-7.266	1.00	0.00	H
ATOM	499	N	THR	34	15.497	-4.311	-4.696	1.00	0.00	N
ATOM	500	CA	THR	34	14.810	-5.341	-3.925	1.00	0.00	C
ATOM	501	C	THR	34	14.196	-4.758	-2.658	1.00	0.00	C
ATOM	502	O	THR	34	13.209	-5.280	-2.139	1.00	0.00	O
ATOM	503	CB	THR	34	15.766	-6.485	-3.542	1.00	0.00	C
ATOM	504	OG1	THR	34	15.062	-7.733	-3.543	1.00	0.00	O
ATOM	505	CG2	THR	34	16.377	-6.242	-2.170	1.00	0.00	C
ATOM	506	H	THR	34	16.471	-4.228	-4.624	1.00	0.00	H
ATOM	507	HA	THR	34	14.023	-5.748	-4.542	1.00	0.00	H
ATOM	508	HB	THR	34	16.561	-6.529	-4.271	1.00	0.00	H
ATOM	509	1HG	THR	34	14.675	-7.882	-2.676	1.00	0.00	H
ATOM	510	1HG2	THR	34	17.374	-6.656	-2.142	1.00	0.00	H
ATOM	511	2HG2	THR	34	15.769	-6.719	-1.416	1.00	0.00	H
ATOM	512	3HG2	THR	34	16.423	-5.181	-1.978	1.00	0.00	H
ATOM	513	N	VAL	35	14.786	-3.675	-2.164	1.00	0.00	N
ATOM	514	CA	VAL	35	14.295	-3.020	-0.955	1.00	0.00	C
ATOM	515	C	VAL	35	13.441	-1.805	-1.297	1.00	0.00	C
ATOM	516	O	VAL	35	12.455	-1.517	-0.621	1.00	0.00	O
ATOM	517	CB	VAL	35	15.457	-2.580	-0.045	1.00	0.00	C
ATOM	518	CG1	VAL	35	15.049	-1.380	0.794	1.00	0.00	C
ATOM	519	CG2	VAL	35	15.905	-3.733	0.841	1.00	0.00	C

ATOM	520	H	VAL	35	15.569	-3.307	-2.621	1.00	0.00	H
ATOM	521	HA	VAL	35	13.690	-3.734	-0.413	1.00	0.00	H
ATOM	522	HB	VAL	35	16.288	-2.290	-0.670	1.00	0.00	H
ATOM	523	1HG1	VAL	35	14.020	-1.491	1.106	1.00	0.00	H
ATOM	524	2HG1	VAL	35	15.686	-1.316	1.667	1.00	0.00	H
ATOM	525	3HG1	VAL	35	15.152	-0.478	0.207	1.00	0.00	H
ATOM	526	1HG2	VAL	35	15.042	-4.192	1.298	1.00	0.00	H
ATOM	527	2HG2	VAL	35	16.427	-4.467	0.243	1.00	0.00	H
ATOM	528	3HG2	VAL	35	16.568	-3.361	1.609	1.00	0.00	H
ATOM	529	N	MET	36	13.829	-1.095	-2.352	1.00	0.00	N
ATOM	530	CA	MET	36	13.097	0.091	-2.784	1.00	0.00	C
ATOM	531	C	MET	36	11.654	-0.260	-3.136	1.00	0.00	C
ATOM	532	O	MET	36	10.728	0.483	-2.806	1.00	0.00	O
ATOM	533	CB	MET	36	13.787	0.729	-3.991	1.00	0.00	C
ATOM	534	CG	MET	36	14.984	1.589	-3.622	1.00	0.00	C
ATOM	535	SD	MET	36	15.252	2.942	-4.782	1.00	0.00	S
ATOM	536	CE	MET	36	13.726	3.860	-4.589	1.00	0.00	C
ATOM	537	H	MET	36	14.624	-1.374	-2.852	1.00	0.00	H
ATOM	538	HA	MET	36	13.096	0.794	-1.967	1.00	0.00	H
ATOM	539	2HB3	MET	36	13.073	1.351	-4.513	1.00	0.00	H
ATOM	540	2HG3	MET	36	15.866	0.965	-3.606	1.00	0.00	H
ATOM	541	1HE	MET	36	12.991	3.488	-5.285	1.00	0.00	H
ATOM	542	2HE	MET	36	13.359	3.741	-3.579	1.00	0.00	H
ATOM	543	3HE	MET	36	13.910	4.908	-4.783	1.00	0.00	H
ATOM	544	HB2	MET	36	14.162	-0.052	-4.651	1.00	0.00	H
ATOM	545	HG2	MET	36	14.821	2.038	-2.641	1.00	0.00	H
ATOM	546	N	ARG	37	11.471	-1.393	-3.804	1.00	0.00	N
ATOM	547	CA	ARG	37	10.140	-1.840	-4.201	1.00	0.00	C
ATOM	548	C	ARG	37	9.343	-2.316	-2.989	1.00	0.00	C
ATOM	549	O	ARG	37	8.118	-2.414	-3.042	1.00	0.00	O
ATOM	550	CB	ARG	37	10.243	-2.964	-5.232	1.00	0.00	C
ATOM	551	CG	ARG	37	11.073	-4.148	-4.762	1.00	0.00	C
ATOM	552	CD	ARG	37	10.247	-5.105	-3.918	1.00	0.00	C
ATOM	553	NE	ARG	37	10.709	-6.487	-4.048	1.00	0.00	N
ATOM	554	CZ	ARG	37	9.928	-7.543	-3.850	1.00	0.00	C
ATOM	555	NH1	ARG	37	8.655	-7.380	-3.515	1.00	0.00	N
ATOM	556	NH2	ARG	37	10.422	-8.768	-3.986	1.00	0.00	N
ATOM	557	H	ARG	37	12.248	-1.943	-4.038	1.00	0.00	H
ATOM	558	HA	ARG	37	9.628	-1.000	-4.647	1.00	0.00	H
ATOM	559	2HB3	ARG	37	10.692	-2.572	-6.131	1.00	0.00	H
ATOM	560	2HG3	ARG	37	11.900	-3.783	-4.172	1.00	0.00	H
ATOM	561	2HD3	ARG	37	9.217	-5.049	-4.235	1.00	0.00	H
ATOM	562	HE	ARG	37	11.646	-6.630	-4.293	1.00	0.00	H
ATOM	563	1HH1	ARG	37	8.282	-6.458	-3.412	1.00	0.00	H
ATOM	564	2HH1	ARG	37	8.071	-8.178	-3.367	1.00	0.00	H
ATOM	565	1HH2	ARG	37	11.380	-8.896	-4.239	1.00	0.00	H
ATOM	566	2HH2	ARG	37	9.834	-9.563	-3.838	1.00	0.00	H
ATOM	567	HB2	ARG	37	9.248	-3.356	-5.445	1.00	0.00	H
ATOM	568	HG2	ARG	37	11.445	-4.697	-5.626	1.00	0.00	H
ATOM	569	HD2	ARG	37	10.333	-4.829	-2.866	1.00	0.00	H
ATOM	570	N	SER	38	10.048	-2.611	-1.902	1.00	0.00	N
ATOM	571	CA	SER	38	9.408	-3.080	-0.679	1.00	0.00	C
ATOM	572	C	SER	38	8.543	-1.985	-0.065	1.00	0.00	C
ATOM	573	O	SER	38	7.521	-2.264	0.565	1.00	0.00	O
ATOM	574	CB	SER	38	10.462	-3.541	0.329	1.00	0.00	C
ATOM	575	OG	SER	38	11.314	-4.523	-0.235	1.00	0.00	O
ATOM	576	H	SER	38	11.023	-2.512	-1.924	1.00	0.00	H
ATOM	577	HA	SER	38	8.779	-3.917	-0.937	1.00	0.00	H
ATOM	578	2HB3	SER	38	9.969	-3.962	1.194	1.00	0.00	H
ATOM	579	HG	SER	38	10.820	-5.051	-0.866	1.00	0.00	H
ATOM	580	HB2	SER	38	11.071	-2.690	0.632	1.00	0.00	H
ATOM	581	N	LEU	39	8.958	-0.737	-0.250	1.00	0.00	N
ATOM	582	CA	LEU	39	8.221	0.402	0.286	1.00	0.00	C
ATOM	583	C	LEU	39	7.091	0.810	-0.652	1.00	0.00	C
ATOM	584	O	LEU	39	5.995	1.155	-0.209	1.00	0.00	O
ATOM	585	CB	LEU	39	9.166	1.583	0.510	1.00	0.00	C
ATOM	586	CG	LEU	39	10.123	1.465	1.697	1.00	0.00	C
ATOM	587	CD1	LEU	39	11.130	2.603	1.687	1.00	0.00	C
ATOM	588	CD2	LEU	39	9.347	1.449	3.006	1.00	0.00	C
ATOM	589	H	LEU	39	9.778	-0.578	-0.760	1.00	0.00	H
ATOM	590	HA	LEU	39	7.798	0.105	1.233	1.00	0.00	H
ATOM	591	2HB3	LEU	39	8.559	2.468	0.660	1.00	0.00	H
ATOM	592	HG	LEU	39	10.668	0.535	1.618	1.00	0.00	H
ATOM	593	1HD1	LEU	39	11.492	2.773	2.689	1.00	0.00	H
ATOM	594	2HD1	LEU	39	10.656	3.501	1.319	1.00	0.00	H
ATOM	595	3HD1	LEU	39	11.958	2.345	1.043	1.00	0.00	H
ATOM	596	1HD2	LEU	39	8.288	1.412	2.797	1.00	0.00	H

ATOM	597	2HD2	LEU	39	9.572	2.342	3.568	1.00	0.00	H
ATOM	598	3HD2	LEU	39	9.630	0.579	3.582	1.00	0.00	H
ATOM	599	HB2	LEU	39	9.821	1.696	-0.354	1.00	0.00	H
ATOM	600	N	GLY	40	7.363	0.766	-1.953	1.00	0.00	N
ATOM	601	CA	GLY	40	6.357	1.131	-2.933	1.00	0.00	C
ATOM	602	C	GLY	40	6.885	2.104	-3.971	1.00	0.00	C
ATOM	603	O	GLY	40	6.161	2.993	-4.420	1.00	0.00	O
ATOM	604	H	GLY	40	8.253	0.483	-2.249	1.00	0.00	H
ATOM	605	2HA	GLY	40	6.017	0.237	-3.435	1.00	0.00	H
ATOM	606	3HA	GLY	40	5.521	1.585	-2.423	1.00	0.00	H
ATOM	607	N	GLN	41	8.147	1.935	-4.348	1.00	0.00	N
ATOM	608	CA	GLN	41	8.771	2.809	-5.337	1.00	0.00	C
ATOM	609	C	GLN	41	8.802	2.143	-6.709	1.00	0.00	C
ATOM	610	O	GLN	41	8.541	2.784	-7.724	1.00	0.00	O
ATOM	611	CB	GLN	41	10.192	3.172	-4.902	1.00	0.00	C
ATOM	612	CG	GLN	41	10.240	4.114	-3.710	1.00	0.00	C
ATOM	613	CD	GLN	41	9.295	5.289	-3.856	1.00	0.00	C
ATOM	614	OE1	GLN	41	9.035	5.759	-4.964	1.00	0.00	O
ATOM	615	NE2	GLN	41	8.773	5.774	-2.735	1.00	0.00	N
ATOM	616	H	GLN	41	8.671	1.209	-3.953	1.00	0.00	H
ATOM	617	HA	GLN	41	8.181	3.710	-5.400	1.00	0.00	H
ATOM	618	2HB3	GLN	41	10.697	3.648	-5.730	1.00	0.00	H
ATOM	619	2HG3	GLN	41	11.247	4.491	-3.607	1.00	0.00	H
ATOM	620	1HE2	GLN	41	9.026	5.350	-1.887	1.00	0.00	H
ATOM	621	2HE2	GLN	41	8.158	6.534	-2.800	1.00	0.00	H
ATOM	622	HB2	GLN	41	10.724	2.269	-4.603	1.00	0.00	H
ATOM	623	HG2	GLN	41	9.944	3.577	-2.808	1.00	0.00	H
ATOM	624	N	ASN	42	9.119	0.852	-6.728	1.00	0.00	N
ATOM	625	CA	ASN	42	9.185	0.101	-7.975	1.00	0.00	C
ATOM	626	C	ASN	42	9.851	0.924	-9.073	1.00	0.00	C
ATOM	627	O	ASN	42	9.322	1.080	-10.174	1.00	0.00	O
ATOM	628	CB	ASN	42	7.780	-0.317	-8.418	1.00	0.00	C
ATOM	629	CG	ASN	42	7.363	-1.652	-7.833	1.00	0.00	C
ATOM	630	OD1	ASN	42	7.904	-2.697	-8.197	1.00	0.00	O
ATOM	631	ND2	ASN	42	6.396	-1.625	-6.924	1.00	0.00	N
ATOM	632	H	ASN	42	9.316	0.396	-5.883	1.00	0.00	H
ATOM	633	HA	ASN	42	9.774	-0.787	-7.798	1.00	0.00	H
ATOM	634	2HB3	ASN	42	7.754	-0.392	-9.494	1.00	0.00	H
ATOM	635	1HD2	ASN	42	6.009	-0.757	-6.683	1.00	0.00	H
ATOM	636	2HD2	ASN	42	6.107	-2.475	-6.529	1.00	0.00	H
ATOM	637	HB2	ASN	42	7.056	0.425	-8.078	1.00	0.00	H
ATOM	638	N	PRO	43	11.041	1.462	-8.768	1.00	0.00	N
ATOM	639	CA	PRO	43	11.806	2.277	-9.715	1.00	0.00	C
ATOM	640	C	PRO	43	12.367	1.454	-10.870	1.00	0.00	C
ATOM	641	O	PRO	43	12.820	0.324	-10.678	1.00	0.00	O
ATOM	642	CD	PRO	43	11.730	1.316	-7.475	1.00	0.00	C
ATOM	643	CB	PRO	43	12.944	2.842	-8.859	1.00	0.00	C
ATOM	644	CG	PRO	43	13.105	1.859	-7.750	1.00	0.00	C
ATOM	645	HA	PRO	43	11.215	3.090	-10.110	1.00	0.00	H
ATOM	646	2HB3	PRO	43	12.671	3.816	-8.487	1.00	0.00	H
ATOM	647	2HG3	PRO	43	13.494	2.357	-6.873	1.00	0.00	H
ATOM	648	2HD3	PRO	43	11.241	1.901	-6.709	1.00	0.00	H
ATOM	649	HD2	PRO	43	11.774	0.262	-7.203	1.00	0.00	H
ATOM	650	HB2	PRO	43	13.846	2.931	-9.466	1.00	0.00	H
ATOM	651	HG2	PRO	43	13.761	1.045	-8.059	1.00	0.00	H
ATOM	652	N	THR	44	12.330	2.023	-12.070	1.00	0.00	N
ATOM	653	CA	THR	44	12.833	1.341	-13.256	1.00	0.00	C
ATOM	654	C	THR	44	14.356	1.376	-13.307	1.00	0.00	C
ATOM	655	O	THR	44	14.991	2.177	-12.619	1.00	0.00	O
ATOM	656	CB	THR	44	12.273	1.969	-14.546	1.00	0.00	C
ATOM	657	OG1	THR	44	12.623	3.355	-14.607	1.00	0.00	O
ATOM	658	CG2	THR	44	10.760	1.819	-14.607	1.00	0.00	C
ATOM	659	H	THR	44	11.955	2.924	-12.159	1.00	0.00	H
ATOM	660	HA	THR	44	12.508	0.311	-13.211	1.00	0.00	H
ATOM	661	HB	THR	44	12.705	1.458	-15.394	1.00	0.00	H
ATOM	662	1HG	THR	44	12.769	3.608	-15.522	1.00	0.00	H
ATOM	663	1HG2	THR	44	10.407	2.136	-15.577	1.00	0.00	H
ATOM	664	2HG2	THR	44	10.306	2.429	-13.840	1.00	0.00	H
ATOM	665	3HG2	THR	44	10.495	0.785	-14.448	1.00	0.00	H
ATOM	666	N	GLU	45	14.937	0.506	-14.127	1.00	0.00	N
ATOM	667	CA	GLU	45	16.388	0.440	-14.266	1.00	0.00	C
ATOM	668	C	GLU	45	16.991	1.838	-14.341	1.00	0.00	C
ATOM	669	O	GLU	45	17.847	2.201	-13.535	1.00	0.00	O
ATOM	670	CB	GLU	45	16.765	-0.358	-15.516	1.00	0.00	C
ATOM	671	CG	GLU	45	16.939	-1.845	-15.258	1.00	0.00	C
ATOM	672	CD	GLU	45	17.628	-2.561	-16.404	1.00	0.00	C
ATOM	673	OE1	GLU	45	17.044	-2.615	-17.507	1.00	0.00	O

ATOM	674	OE2	GLU	45	18.752	-3.064	-16.197	1.00	0.00	O
ATOM	675	H	GLU	45	14.378	-0.105	-14.648	1.00	0.00	H
ATOM	676	HA	GLU	45	16.782	-0.062	-13.396	1.00	0.00	H
ATOM	677	2HB3	GLU	45	17.695	0.027	-15.906	1.00	0.00	H
ATOM	678	2HG3	GLU	45	15.965	-2.289	-15.112	1.00	0.00	H
ATOM	679	HB2	GLU	45	15.973	-0.266	-16.260	1.00	0.00	H
ATOM	680	HG2	GLU	45	17.556	-1.993	-14.374	1.00	0.00	H
ATOM	681	N	ALA	46	16.538	2.620	-15.316	1.00	0.00	N
ATOM	682	CA	ALA	46	17.033	3.981	-15.497	1.00	0.00	C
ATOM	683	C	ALA	46	17.019	4.747	-14.179	1.00	0.00	C
ATOM	684	O	ALA	46	17.932	5.523	-13.895	1.00	0.00	O
ATOM	685	CB	ALA	46	16.203	4.709	-16.542	1.00	0.00	C
ATOM	686	H	ALA	46	15.855	2.275	-15.928	1.00	0.00	H
ATOM	687	HA	ALA	46	18.050	3.922	-15.857	1.00	0.00	H
ATOM	688	1HB	ALA	46	16.690	4.636	-17.503	1.00	0.00	H
ATOM	689	2HB	ALA	46	15.221	4.260	-16.600	1.00	0.00	H
ATOM	690	3HB	ALA	46	16.107	5.749	-16.265	1.00	0.00	H
ATOM	691	N	GLU	47	15.980	4.528	-13.380	1.00	0.00	N
ATOM	692	CA	GLU	47	15.848	5.202	-12.094	1.00	0.00	C
ATOM	693	C	GLU	47	16.936	4.742	-11.126	1.00	0.00	C
ATOM	694	O	GLU	47	17.672	5.558	-10.569	1.00	0.00	O
ATOM	695	CB	GLU	47	14.468	4.933	-11.492	1.00	0.00	C
ATOM	696	CG	GLU	47	13.320	5.435	-12.353	1.00	0.00	C
ATOM	697	CD	GLU	47	13.069	6.921	-12.181	1.00	0.00	C
ATOM	698	OE1	GLU	47	13.809	7.722	-12.793	1.00	0.00	O
ATOM	699	OE2	GLU	47	12.134	7.283	-11.438	1.00	0.00	O
ATOM	700	H	GLU	47	15.284	3.899	-13.664	1.00	0.00	H
ATOM	701	HA	GLU	47	15.959	6.262	-12.262	1.00	0.00	H
ATOM	702	2HB3	GLU	47	14.408	5.422	-10.529	1.00	0.00	H
ATOM	703	2HG3	GLU	47	12.422	4.900	-12.081	1.00	0.00	H
ATOM	704	HB2	GLU	47	14.322	3.859	-11.381	1.00	0.00	H
ATOM	705	HG2	GLU	47	13.555	5.268	-13.405	1.00	0.00	H
ATOM	706	N	LEU	48	17.030	3.431	-10.931	1.00	0.00	N
ATOM	707	CA	LEU	48	18.026	2.861	-10.031	1.00	0.00	C
ATOM	708	C	LEU	48	19.434	3.287	-10.436	1.00	0.00	C
ATOM	709	O	LEU	48	20.229	3.712	-9.600	1.00	0.00	O
ATOM	710	CB	LEU	48	17.923	1.336	-10.024	1.00	0.00	C
ATOM	711	CG	LEU	48	16.611	0.753	-9.500	1.00	0.00	C
ATOM	712	CD1	LEU	48	16.362	-0.625	-10.092	1.00	0.00	C
ATOM	713	CD2	LEU	48	16.626	0.687	-7.980	1.00	0.00	C
ATOM	714	H	LEU	48	16.416	2.832	-11.404	1.00	0.00	H
ATOM	715	HA	LEU	48	17.824	3.233	-9.036	1.00	0.00	H
ATOM	716	2HB3	LEU	48	18.725	0.953	-9.408	1.00	0.00	H
ATOM	717	HG	LEU	48	15.794	1.397	-9.799	1.00	0.00	H
ATOM	718	1HD1	LEU	48	16.732	-1.381	-9.416	1.00	0.00	H
ATOM	719	2HD1	LEU	48	16.877	-0.707	-11.040	1.00	0.00	H
ATOM	720	3HD1	LEU	48	15.303	-0.766	-10.246	1.00	0.00	H
ATOM	721	1HD2	LEU	48	17.470	1.246	-7.605	1.00	0.00	H
ATOM	722	2HD2	LEU	48	16.711	-0.344	-7.665	1.00	0.00	H
ATOM	723	3HD2	LEU	48	15.711	1.108	-7.592	1.00	0.00	H
ATOM	724	HB2	LEU	48	18.000	0.962	-11.046	1.00	0.00	H
ATOM	725	N	GLN	49	19.732	3.170	-11.726	1.00	0.00	N
ATOM	726	CA	GLN	49	21.044	3.544	-12.243	1.00	0.00	C
ATOM	727	C	GLN	49	21.360	5.001	-11.924	1.00	0.00	C
ATOM	728	O	GLN	49	22.462	5.324	-11.482	1.00	0.00	O
ATOM	729	CB	GLN	49	21.104	3.317	-13.756	1.00	0.00	C
ATOM	730	CG	GLN	49	21.306	1.861	-14.144	1.00	0.00	C
ATOM	731	CD	GLN	49	22.772	1.493	-14.279	1.00	0.00	C
ATOM	732	OE1	GLN	49	23.277	1.303	-15.387	1.00	0.00	O
ATOM	733	NE2	GLN	49	23.463	1.389	-13.151	1.00	0.00	N
ATOM	734	H	GLN	49	19.056	2.825	-12.345	1.00	0.00	H
ATOM	735	HA	GLN	49	21.780	2.914	-11.764	1.00	0.00	H
ATOM	736	2HB3	GLN	49	21.920	3.893	-14.161	1.00	0.00	H
ATOM	737	2HG3	GLN	49	20.817	1.683	-15.090	1.00	0.00	H
ATOM	738	1HE2	GLN	49	22.994	1.554	-12.304	1.00	0.00	H
ATOM	739	2HE2	GLN	49	24.410	1.153	-13.208	1.00	0.00	H
ATOM	740	HB2	GLN	49	20.161	3.624	-14.207	1.00	0.00	H
ATOM	741	HG2	GLN	49	20.881	1.217	-13.374	1.00	0.00	H
ATOM	742	N	ASP	50	20.386	5.876	-12.150	1.00	0.00	N
ATOM	743	CA	ASP	50	20.560	7.299	-11.884	1.00	0.00	C
ATOM	744	C	ASP	50	20.849	7.544	-10.408	1.00	0.00	C
ATOM	745	O	ASP	50	21.698	8.367	-10.059	1.00	0.00	O
ATOM	746	CB	ASP	50	19.313	8.075	-12.308	1.00	0.00	C
ATOM	747	CG	ASP	50	19.359	8.494	-13.764	1.00	0.00	C
ATOM	748	OD1	ASP	50	20.329	9.177	-14.154	1.00	0.00	O
ATOM	749	OD2	ASP	50	18.425	8.141	-14.514	1.00	0.00	O
ATOM	750	H	ASP	50	19.529	5.557	-12.503	1.00	0.00	H

ATOM	751	HA	ASP	50	21.401	7.643	-12.466	1.00	0.00	H
ATOM	752	2HB3	ASP	50	19.224	8.962	-11.700	1.00	0.00	H
ATOM	753	HB2	ASP	50	18.433	7.446	-12.179	1.00	0.00	H
ATOM	754	N	MET	51	20.139	6.829	-9.542	1.00	0.00	N
ATOM	755	CA	MET	51	20.321	6.969	-8.102	1.00	0.00	C
ATOM	756	C	MET	51	21.669	6.403	-7.667	1.00	0.00	C
ATOM	757	O	MET	51	22.336	6.964	-6.796	1.00	0.00	O
ATOM	758	CB	MET	51	19.191	6.260	-7.353	1.00	0.00	C
ATOM	759	CG	MET	51	17.869	7.012	-7.398	1.00	0.00	C
ATOM	760	SD	MET	51	16.591	6.228	-6.397	1.00	0.00	S
ATOM	761	CE	MET	51	15.915	5.055	-7.568	1.00	0.00	C
ATOM	762	H	MET	51	19.478	6.188	-9.881	1.00	0.00	H
ATOM	763	HA	MET	51	20.295	8.022	-7.867	1.00	0.00	H
ATOM	764	2HB3	MET	51	19.478	6.144	-6.319	1.00	0.00	H
ATOM	765	2HG3	MET	51	17.530	7.054	-8.423	1.00	0.00	H
ATOM	766	1HE	MET	51	16.699	4.706	-8.224	1.00	0.00	H
ATOM	767	2HE	MET	51	15.492	4.216	-7.036	1.00	0.00	H
ATOM	768	3HE	MET	51	15.145	5.535	-8.156	1.00	0.00	H
ATOM	769	HB2	MET	51	19.006	5.288	-7.808	1.00	0.00	H
ATOM	770	HG2	MET	51	18.008	8.019	-7.004	1.00	0.00	H
ATOM	771	N	ILE	52	22.062	5.287	-8.274	1.00	0.00	N
ATOM	772	CA	ILE	52	23.331	4.648	-7.949	1.00	0.00	C
ATOM	773	C	ILE	52	24.508	5.512	-8.379	1.00	0.00	C
ATOM	774	O	ILE	52	25.460	5.706	-7.623	1.00	0.00	O
ATOM	775	CB	ILE	52	23.449	3.265	-8.615	1.00	0.00	C
ATOM	776	CG2	ILE	52	24.858	2.715	-8.447	1.00	0.00	C
ATOM	777	CG1	ILE	52	22.421	2.299	-8.023	1.00	0.00	C
ATOM	778	CD1	ILE	52	22.093	1.135	-8.933	1.00	0.00	C
ATOM	779	H	ILE	52	21.485	4.889	-8.959	1.00	0.00	H
ATOM	780	HA	ILE	52	23.369	4.513	-6.877	1.00	0.00	H
ATOM	781	HB	ILE	52	23.258	3.380	-9.671	1.00	0.00	H
ATOM	782	1HG2	ILE	52	25.162	2.814	-7.415	1.00	0.00	H
ATOM	783	2HG2	ILE	52	24.874	1.673	-8.727	1.00	0.00	H
ATOM	784	3HG2	ILE	52	25.539	3.269	-9.078	1.00	0.00	H
ATOM	785	2HG3	ILE	52	21.505	2.836	-7.828	1.00	0.00	H
ATOM	786	1HD1	ILE	52	21.990	0.235	-8.344	1.00	0.00	H
ATOM	787	2HD1	ILE	52	21.168	1.333	-9.453	1.00	0.00	H
ATOM	788	3HD1	ILE	52	22.890	1.004	-9.652	1.00	0.00	H
ATOM	789	HG2	ILE	52	22.809	1.876	-7.096	1.00	0.00	H
ATOM	790	N	ASN	53	24.440	6.030	-9.601	1.00	0.00	N
ATOM	791	CA	ASN	53	25.502	6.875	-10.135	1.00	0.00	C
ATOM	792	C	ASN	53	25.601	8.183	-9.353	1.00	0.00	C
ATOM	793	O	ASN	53	26.693	8.702	-9.130	1.00	0.00	O
ATOM	794	CB	ASN	53	25.253	7.172	-11.616	1.00	0.00	C
ATOM	795	CG	ASN	53	25.687	6.029	-12.514	1.00	0.00	C
ATOM	796	OD1	ASN	53	26.878	5.753	-12.653	1.00	0.00	O
ATOM	797	ND2	ASN	53	24.718	5.361	-13.130	1.00	0.00	N
ATOM	798	H	ASN	53	23.656	5.840	-10.159	1.00	0.00	H
ATOM	799	HA	ASN	53	26.434	6.339	-10.035	1.00	0.00	H
ATOM	800	2HB3	ASN	53	25.803	8.056	-11.899	1.00	0.00	H
ATOM	801	1HD2	ASN	53	23.792	5.637	-12.970	1.00	0.00	H
ATOM	802	2HD2	ASN	53	24.970	4.618	-13.715	1.00	0.00	H
ATOM	803	HB2	ASN	53	24.187	7.329	-11.781	1.00	0.00	H
ATOM	804	N	GLU	54	24.452	8.707	-8.941	1.00	0.00	N
ATOM	805	CA	GLU	54	24.410	9.952	-8.185	1.00	0.00	C
ATOM	806	C	GLU	54	25.196	9.826	-6.883	1.00	0.00	C
ATOM	807	O	GLU	54	25.897	10.751	-6.475	1.00	0.00	O
ATOM	808	CB	GLU	54	22.962	10.343	-7.882	1.00	0.00	C
ATOM	809	CG	GLU	54	22.290	11.108	-9.011	1.00	0.00	C
ATOM	810	CD	GLU	54	22.722	12.560	-9.072	1.00	0.00	C
ATOM	811	OE1	GLU	54	23.935	12.823	-8.935	1.00	0.00	O
ATOM	812	OE2	GLU	54	21.848	13.432	-9.255	1.00	0.00	O
ATOM	813	H	GLU	54	23.612	8.245	-9.150	1.00	0.00	H
ATOM	814	HA	GLU	54	24.862	10.724	-8.790	1.00	0.00	H
ATOM	815	2HB3	GLU	54	22.946	10.963	-6.998	1.00	0.00	H
ATOM	816	2HG3	GLU	54	21.220	11.070	-8.867	1.00	0.00	H
ATOM	817	HB2	GLU	54	22.367	9.444	-7.725	1.00	0.00	H
ATOM	818	HG2	GLU	54	22.557	10.656	-9.965	1.00	0.00	H
ATOM	819	N	VAL	55	25.073	8.672	-6.234	1.00	0.00	N
ATOM	820	CA	VAL	55	25.772	8.422	-4.978	1.00	0.00	C
ATOM	821	C	VAL	55	27.179	7.894	-5.229	1.00	0.00	C
ATOM	822	O	VAL	55	28.109	8.191	-4.479	1.00	0.00	O
ATOM	823	CB	VAL	55	25.005	7.415	-4.101	1.00	0.00	C
ATOM	824	CG1	VAL	55	25.809	7.069	-2.856	1.00	0.00	C
ATOM	825	CG2	VAL	55	23.639	7.971	-3.724	1.00	0.00	C
ATOM	826	H	VAL	55	24.499	7.972	-6.609	1.00	0.00	H
ATOM	827	HA	VAL	55	25.840	9.358	-4.443	1.00	0.00	H

ATOM	828	HB	VAL	55	24.858	6.511	-4.671	1.00	0.00	H
ATOM	829	1HG1	VAL	55	25.237	7.320	-1.977	1.00	0.00	H
ATOM	830	2HG1	VAL	55	26.034	6.013	-2.856	1.00	0.00	H
ATOM	831	3HG1	VAL	55	26.731	7.634	-2.858	1.00	0.00	H
ATOM	832	1HG2	VAL	55	23.684	8.391	-2.729	1.00	0.00	H
ATOM	833	2HG2	VAL	55	23.356	8.740	-4.428	1.00	0.00	H
ATOM	834	3HG2	VAL	55	22.909	7.175	-3.745	1.00	0.00	H
ATOM	835	N	ASP	56	27.329	7.105	-6.288	1.00	0.00	N
ATOM	836	CA	ASP	56	28.626	6.534	-6.639	1.00	0.00	C
ATOM	837	C	ASP	56	29.704	7.613	-6.666	1.00	0.00	C
ATOM	838	O	ASP	56	29.642	8.547	-7.464	1.00	0.00	O
ATOM	839	CB	ASP	56	28.548	5.839	-8.000	1.00	0.00	C
ATOM	840	CG	ASP	56	29.613	4.773	-8.166	1.00	0.00	C
ATOM	841	OD1	ASP	56	29.579	3.778	-7.412	1.00	0.00	O
ATOM	842	OD2	ASP	56	30.480	4.935	-9.049	1.00	0.00	O
ATOM	843	H	ASP	56	26.550	6.904	-6.848	1.00	0.00	H
ATOM	844	HA	ASP	56	28.881	5.806	-5.885	1.00	0.00	H
ATOM	845	2HB3	ASP	56	28.674	6.575	-8.780	1.00	0.00	H
ATOM	846	HB2	ASP	56	27.579	5.350	-8.104	1.00	0.00	H
ATOM	847	N	ALA	57	30.692	7.476	-5.787	1.00	0.00	N
ATOM	848	CA	ALA	57	31.784	8.437	-5.711	1.00	0.00	C
ATOM	849	C	ALA	57	33.110	7.790	-6.100	1.00	0.00	C
ATOM	850	O	ALA	57	33.819	8.286	-6.977	1.00	0.00	O
ATOM	851	CB	ALA	57	31.873	9.025	-4.309	1.00	0.00	C
ATOM	852	H	ALA	57	30.686	6.709	-5.177	1.00	0.00	H
ATOM	853	HA	ALA	57	31.572	9.241	-6.399	1.00	0.00	H
ATOM	854	1HB	ALA	57	32.777	8.679	-3.832	1.00	0.00	H
ATOM	855	2HB	ALA	57	31.889	10.102	-4.372	1.00	0.00	H
ATOM	856	3HB	ALA	57	31.016	8.709	-3.734	1.00	0.00	H
ATOM	857	N	ASP	58	33.442	6.687	-5.442	1.00	0.00	N
ATOM	858	CA	ASP	58	34.684	5.973	-5.718	1.00	0.00	C
ATOM	859	C	ASP	58	34.967	5.939	-7.216	1.00	0.00	C
ATOM	860	O	ASP	58	36.092	6.177	-7.652	1.00	0.00	O
ATOM	861	CB	ASP	58	34.611	4.549	-5.167	1.00	0.00	C
ATOM	862	CG	ASP	58	33.706	3.654	-5.990	1.00	0.00	C
ATOM	863	OD1	ASP	58	32.649	4.138	-6.444	1.00	0.00	O
ATOM	864	OD2	ASP	58	34.053	2.469	-6.178	1.00	0.00	O
ATOM	865	H	ASP	58	32.835	6.341	-4.752	1.00	0.00	H
ATOM	866	HA	ASP	58	35.486	6.501	-5.225	1.00	0.00	H
ATOM	867	2HB3	ASP	58	34.235	4.580	-4.156	1.00	0.00	H
ATOM	868	HB2	ASP	58	35.606	4.102	-5.183	1.00	0.00	H
ATOM	869	N	GLY	59	33.935	5.639	-8.003	1.00	0.00	N
ATOM	870	CA	GLY	59	34.096	5.579	-9.444	1.00	0.00	C
ATOM	871	C	GLY	59	34.102	4.156	-9.966	1.00	0.00	C
ATOM	872	O	GLY	59	34.861	3.826	-10.876	1.00	0.00	O
ATOM	873	H	GLY	59	33.062	5.459	-7.600	1.00	0.00	H
ATOM	874	2HA	GLY	59	33.285	6.119	-9.908	1.00	0.00	H
ATOM	875	3HA	GLY	59	35.029	6.053	-9.712	1.00	0.00	H
ATOM	876	N	ASN	60	33.254	3.312	-9.388	1.00	0.00	N
ATOM	877	CA	ASN	60	33.165	1.915	-9.800	1.00	0.00	C
ATOM	878	C	ASN	60	31.796	1.610	-10.400	1.00	0.00	C
ATOM	879	O	ASN	60	31.695	0.993	-11.461	1.00	0.00	O
ATOM	880	CB	ASN	60	33.430	0.993	-8.609	1.00	0.00	C
ATOM	881	CG	ASN	60	32.248	0.921	-7.661	1.00	0.00	C
ATOM	882	OD1	ASN	60	31.776	1.942	-7.162	1.00	0.00	O
ATOM	883	ND2	ASN	60	31.766	-0.290	-7.406	1.00	0.00	N
ATOM	884	H	ASN	60	32.673	3.635	-8.667	1.00	0.00	H
ATOM	885	HA	ASN	60	33.923	1.744	-10.552	1.00	0.00	H
ATOM	886	2HB3	ASN	60	34.287	1.357	-8.062	1.00	0.00	H
ATOM	887	1HD2	ASN	60	32.194	-1.058	-7.840	1.00	0.00	H
ATOM	888	2HD2	ASN	60	31.003	-0.365	-6.798	1.00	0.00	H
ATOM	889	HB2	ASN	60	33.620	-0.018	-8.967	1.00	0.00	H
ATOM	890	N	GLY	61	30.745	2.048	-9.715	1.00	0.00	N
ATOM	891	CA	GLY	61	29.395	1.812	-10.196	1.00	0.00	C
ATOM	892	C	GLY	61	28.431	1.476	-9.076	1.00	0.00	C
ATOM	893	O	GLY	61	27.399	2.129	-8.917	1.00	0.00	O
ATOM	894	H	GLY	61	30.887	2.533	-8.875	1.00	0.00	H
ATOM	895	2HA	GLY	61	29.046	2.699	-10.704	1.00	0.00	H
ATOM	896	3HA	GLY	61	29.415	0.991	-10.897	1.00	0.00	H
ATOM	897	N	THR	62	28.766	0.453	-8.296	1.00	0.00	N
ATOM	898	CA	THR	62	27.921	0.029	-7.187	1.00	0.00	C
ATOM	899	C	THR	62	28.228	0.826	-5.925	1.00	0.00	C
ATOM	900	O	THR	62	29.391	1.005	-5.560	1.00	0.00	O
ATOM	901	CB	THR	62	28.099	-1.471	-6.887	1.00	0.00	C
ATOM	902	OG1	THR	62	29.466	-1.749	-6.566	1.00	0.00	O
ATOM	903	CG2	THR	62	27.673	-2.315	-8.079	1.00	0.00	C
ATOM	904	H	THR	62	29.602	-0.028	-8.472	1.00	0.00	H

ATOM	905	HA	THR	62	26.892	0.199	-7.468	1.00	0.00	H
ATOM	906	HB	THR	62	27.478	-1.731	-6.042	1.00	0.00	H
ATOM	907	1HG	THR	62	29.817	-2.391	-7.187	1.00	0.00	H
ATOM	908	1HG2	THR	62	26.596	-2.414	-8.085	1.00	0.00	H
ATOM	909	2HG2	THR	62	28.123	-3.296	-8.007	1.00	0.00	H
ATOM	910	3HG2	THR	62	27.994	-1.838	-8.991	1.00	0.00	H
ATOM	911	N	ILE	63	27.180	1.304	-5.262	1.00	0.00	N
ATOM	912	CA	ILE	63	27.340	2.081	-4.040	1.00	0.00	C
ATOM	913	C	ILE	63	28.190	1.334	-3.020	1.00	0.00	C
ATOM	914	O	ILE	63	28.183	0.104	-2.972	1.00	0.00	O
ATOM	915	CB	ILE	63	25.976	2.421	-3.408	1.00	0.00	C
ATOM	916	CG2	ILE	63	26.174	3.203	-2.117	1.00	0.00	C
ATOM	917	CG1	ILE	63	25.115	3.213	-4.393	1.00	0.00	C
ATOM	918	CD1	ILE	63	24.068	2.374	-5.089	1.00	0.00	C
ATOM	919	H	ILE	63	26.280	1.128	-5.603	1.00	0.00	H
ATOM	920	HA	ILE	63	27.836	3.007	-4.297	1.00	0.00	H
ATOM	921	HB	ILE	63	25.477	1.496	-3.166	1.00	0.00	H
ATOM	922	1HG2	ILE	63	25.218	3.343	-1.633	1.00	0.00	H
ATOM	923	2HG2	ILE	63	26.833	2.657	-1.461	1.00	0.00	H
ATOM	924	3HG2	ILE	63	26.606	4.167	-2.344	1.00	0.00	H
ATOM	925	2HG3	ILE	63	25.753	3.645	-5.151	1.00	0.00	H
ATOM	926	1HD1	ILE	63	24.538	1.766	-5.847	1.00	0.00	H
ATOM	927	2HD1	ILE	63	23.578	1.737	-4.368	1.00	0.00	H
ATOM	928	3HD1	ILE	63	23.336	3.024	-5.551	1.00	0.00	H
ATOM	929	HG2	ILE	63	24.583	4.002	-3.859	1.00	0.00	H
ATOM	930	N	ASP	64	28.921	2.084	-2.203	1.00	0.00	N
ATOM	931	CA	ASP	64	29.775	1.492	-1.180	1.00	0.00	C
ATOM	932	C	ASP	64	29.286	1.861	0.216	1.00	0.00	C
ATOM	933	O	ASP	64	28.241	2.494	0.373	1.00	0.00	O
ATOM	934	CB	ASP	64	31.223	1.953	-1.364	1.00	0.00	C
ATOM	935	CG	ASP	64	31.711	1.767	-2.786	1.00	0.00	C
ATOM	936	OD1	ASP	64	31.013	1.094	-3.571	1.00	0.00	O
ATOM	937	OD2	ASP	64	32.797	2.296	-3.115	1.00	0.00	O
ATOM	938	H	ASP	64	28.885	3.060	-2.289	1.00	0.00	H
ATOM	939	HA	ASP	64	29.732	0.419	-1.292	1.00	0.00	H
ATOM	940	2HB3	ASP	64	31.861	1.384	-0.706	1.00	0.00	H
ATOM	941	HB2	ASP	64	31.297	3.015	-1.129	1.00	0.00	H
ATOM	942	N	PHE	65	30.045	1.459	1.230	1.00	0.00	N
ATOM	943	CA	PHE	65	29.687	1.743	2.614	1.00	0.00	C
ATOM	944	C	PHE	65	29.653	3.248	2.870	1.00	0.00	C
ATOM	945	O	PHE	65	28.694	3.790	3.421	1.00	0.00	O
ATOM	946	CB	PHE	65	30.680	1.076	3.569	1.00	0.00	C
ATOM	947	CG	PHE	65	30.077	0.691	4.890	1.00	0.00	C
ATOM	948	CD1	PHE	65	29.377	1.620	5.643	1.00	0.00	C
ATOM	949	CE1	PHE	65	28.821	1.269	6.859	1.00	0.00	C
ATOM	950	CZ	PHE	65	28.959	-0.020	7.333	1.00	0.00	C
ATOM	951	CE2	PHE	65	29.653	-0.955	6.592	1.00	0.00	C
ATOM	952	CD2	PHE	65	30.208	-0.599	5.377	1.00	0.00	C
ATOM	953	H	PHE	65	30.866	0.957	1.043	1.00	0.00	H
ATOM	954	HA	PHE	65	28.703	1.339	2.792	1.00	0.00	H
ATOM	955	2HB3	PHE	65	31.496	1.757	3.762	1.00	0.00	H
ATOM	956	1HD	PHE	65	29.268	2.631	5.273	1.00	0.00	H
ATOM	957	1HE	PHE	65	28.280	2.003	7.438	1.00	0.00	H
ATOM	958	HZ	PHE	65	28.526	-0.297	8.282	1.00	0.00	H
ATOM	959	2HE	PHE	65	29.763	-1.964	6.959	1.00	0.00	H
ATOM	960	2HD	PHE	65	30.750	-1.331	4.797	1.00	0.00	H
ATOM	961	HB2	PHE	65	31.058	0.159	3.118	1.00	0.00	H
ATOM	962	N	PRO	66	30.726	3.942	2.456	1.00	0.00	N
ATOM	963	CA	PRO	66	30.842	5.392	2.626	1.00	0.00	C
ATOM	964	C	PRO	66	29.878	6.162	1.732	1.00	0.00	C
ATOM	965	O	PRO	66	29.143	7.031	2.201	1.00	0.00	O
ATOM	966	CD	PRO	66	31.903	3.362	1.790	1.00	0.00	C
ATOM	967	CB	PRO	66	32.291	5.680	2.225	1.00	0.00	C
ATOM	968	CG	PRO	66	32.656	4.569	1.302	1.00	0.00	C
ATOM	969	HA	PRO	66	30.688	5.685	3.656	1.00	0.00	H
ATOM	970	2HB3	PRO	66	32.919	5.686	3.104	1.00	0.00	H
ATOM	971	2HG3	PRO	66	33.720	4.390	1.346	1.00	0.00	H
ATOM	972	2HD3	PRO	66	32.504	2.802	2.491	1.00	0.00	H
ATOM	973	HD2	PRO	66	31.581	2.738	0.957	1.00	0.00	H
ATOM	974	HB2	PRO	66	32.346	6.655	1.740	1.00	0.00	H
ATOM	975	HG2	PRO	66	32.343	4.807	0.285	1.00	0.00	H
ATOM	976	N	GLU	67	29.884	5.838	0.444	1.00	0.00	N
ATOM	977	CA	GLU	67	29.011	6.499	-0.517	1.00	0.00	C
ATOM	978	C	GLU	67	27.547	6.355	-0.107	1.00	0.00	C
ATOM	979	O	GLU	67	26.789	7.325	-0.121	1.00	0.00	O
ATOM	980	CB	GLU	67	29.219	5.917	-1.916	1.00	0.00	C
ATOM	981	CG	GLU	67	30.673	5.898	-2.357	1.00	0.00	C

ATOM	982	CD	GLU	67	30.899	5.034	-3.583	1.00	0.00	C
ATOM	983	OE1	GLU	67	29.909	4.717	-4.275	1.00	0.00	O
ATOM	984	OE2	GLU	67	32.065	4.676	-3.850	1.00	0.00	O
ATOM	985	H	GLU	67	30.493	5.135	0.131	1.00	0.00	H
ATOM	986	HA	GLU	67	29.266	7.548	-0.529	1.00	0.00	H
ATOM	987	2HB3	GLU	67	28.657	6.509	-2.625	1.00	0.00	H
ATOM	988	2HG3	GLU	67	31.275	5.512	-1.547	1.00	0.00	H
ATOM	989	HB2	GLU	67	28.878	4.881	-1.934	1.00	0.00	H
ATOM	990	HG2	GLU	67	30.989	6.910	-2.609	1.00	0.00	H
ATOM	991	N	PHE	68	27.157	5.139	0.256	1.00	0.00	N
ATOM	992	CA	PHE	68	25.783	4.867	0.668	1.00	0.00	C
ATOM	993	C	PHE	68	25.297	5.915	1.665	1.00	0.00	C
ATOM	994	O	PHE	68	24.196	6.450	1.532	1.00	0.00	O
ATOM	995	CB	PHE	68	25.682	3.471	1.287	1.00	0.00	C
ATOM	996	CG	PHE	68	24.386	3.229	2.008	1.00	0.00	C
ATOM	997	CD1	PHE	68	23.218	2.991	1.300	1.00	0.00	C
ATOM	998	CE1	PHE	68	22.025	2.767	1.957	1.00	0.00	C
ATOM	999	CZ	PHE	68	21.988	2.780	3.340	1.00	0.00	C
ATOM	1000	CE2	PHE	68	23.143	3.015	4.057	1.00	0.00	C
ATOM	1001	CD2	PHE	68	24.334	3.240	3.392	1.00	0.00	C
ATOM	1002	H	PHE	68	27.807	4.404	0.247	1.00	0.00	H
ATOM	1003	HA	PHE	68	25.159	4.908	-0.212	1.00	0.00	H
ATOM	1004	2HB3	PHE	68	26.485	3.341	1.997	1.00	0.00	H
ATOM	1005	1HD	PHE	68	23.248	2.980	0.217	1.00	0.00	H
ATOM	1006	1HE	PHE	68	21.123	2.582	1.393	1.00	0.00	H
ATOM	1007	HZ	PHE	68	21.054	2.604	3.857	1.00	0.00	H
ATOM	1008	2HE	PHE	68	23.116	3.026	5.136	1.00	0.00	H
ATOM	1009	2HD	PHE	68	25.238	3.424	3.955	1.00	0.00	H
ATOM	1010	HB2	PHE	68	25.756	2.719	0.501	1.00	0.00	H
ATOM	1011	N	LEU	69	26.125	6.205	2.662	1.00	0.00	N
ATOM	1012	CA	LEU	69	25.781	7.189	3.682	1.00	0.00	C
ATOM	1013	C	LEU	69	25.236	8.464	3.045	1.00	0.00	C
ATOM	1014	O	LEU	69	24.402	9.155	3.630	1.00	0.00	O
ATOM	1015	CB	LEU	69	27.005	7.516	4.539	1.00	0.00	C
ATOM	1016	CG	LEU	69	27.733	6.318	5.154	1.00	0.00	C
ATOM	1017	CD1	LEU	69	28.980	6.774	5.896	1.00	0.00	C
ATOM	1018	CD2	LEU	69	26.804	5.553	6.087	1.00	0.00	C
ATOM	1019	H	LEU	69	26.991	5.747	2.712	1.00	0.00	H
ATOM	1020	HA	LEU	69	25.016	6.760	4.310	1.00	0.00	H
ATOM	1021	2HB3	LEU	69	26.682	8.157	5.345	1.00	0.00	H
ATOM	1022	HG	LEU	69	28.040	5.646	4.365	1.00	0.00	H
ATOM	1023	1HD1	LEU	69	29.754	7.012	5.183	1.00	0.00	H
ATOM	1024	2HD1	LEU	69	29.320	5.983	6.547	1.00	0.00	H
ATOM	1025	3HD1	LEU	69	28.748	7.649	6.484	1.00	0.00	H
ATOM	1026	1HD2	LEU	69	25.960	6.177	6.346	1.00	0.00	H
ATOM	1027	2HD2	LEU	69	27.340	5.283	6.987	1.00	0.00	H
ATOM	1028	3HD2	LEU	69	26.453	4.660	5.594	1.00	0.00	H
ATOM	1029	HB2	LEU	69	27.760	8.007	3.924	1.00	0.00	H
ATOM	1030	N	THR	70	25.715	8.770	1.844	1.00	0.00	N
ATOM	1031	CA	THR	70	25.277	9.962	1.127	1.00	0.00	C
ATOM	1032	C	THR	70	23.798	9.875	0.770	1.00	0.00	C
ATOM	1033	O	THR	70	23.041	10.821	0.986	1.00	0.00	O
ATOM	1034	CB	THR	70	26.093	10.176	-0.161	1.00	0.00	C
ATOM	1035	OG1	THR	70	27.477	10.350	0.159	1.00	0.00	O
ATOM	1036	CG2	THR	70	25.588	11.389	-0.926	1.00	0.00	C
ATOM	1037	H	THR	70	26.380	8.180	1.430	1.00	0.00	H
ATOM	1038	HA	THR	70	25.432	10.815	1.772	1.00	0.00	H
ATOM	1039	HB	THR	70	25.983	9.301	-0.787	1.00	0.00	H
ATOM	1040	1HG	THR	70	27.708	9.785	0.900	1.00	0.00	H
ATOM	1041	1HG2	THR	70	25.717	11.225	-1.986	1.00	0.00	H
ATOM	1042	2HG2	THR	70	26.147	12.263	-0.626	1.00	0.00	H
ATOM	1043	3HG2	THR	70	24.542	11.539	-0.711	1.00	0.00	H
ATOM	1044	N	MET	71	23.392	8.735	0.221	1.00	0.00	N
ATOM	1045	CA	MET	71	22.000	8.525	-0.164	1.00	0.00	C
ATOM	1046	C	MET	71	21.084	8.595	1.053	1.00	0.00	C
ATOM	1047	O	MET	71	20.163	9.412	1.100	1.00	0.00	O
ATOM	1048	CB	MET	71	21.841	7.173	-0.859	1.00	0.00	C
ATOM	1049	CG	MET	71	20.413	6.872	-1.285	1.00	0.00	C
ATOM	1050	SD	MET	71	20.217	5.201	-1.932	1.00	0.00	S
ATOM	1051	CE	MET	71	20.478	4.230	-0.447	1.00	0.00	C
ATOM	1052	H	MET	71	24.043	8.016	0.072	1.00	0.00	H
ATOM	1053	HA	MET	71	21.726	9.309	-0.853	1.00	0.00	H
ATOM	1054	2HB3	MET	71	22.165	6.393	-0.186	1.00	0.00	H
ATOM	1055	2HG3	MET	71	20.124	7.577	-2.050	1.00	0.00	H
ATOM	1056	1HE	MET	71	21.536	4.092	-0.289	1.00	0.00	H
ATOM	1057	2HE	MET	71	20.050	4.748	0.398	1.00	0.00	H
ATOM	1058	3HE	MET	71	20.000	3.267	-0.560	1.00	0.00	H

ATOM	1059	HB2	MET	71	22.442	7.159	-1.770	1.00	0.00	H
ATOM	1060	HG2	MET	71	19.752	6.957	-0.423	1.00	0.00	H
ATOM	1061	N	MET	72	21.341	7.737	2.032	1.00	0.00	N
ATOM	1062	CA	MET	72	20.537	7.704	3.250	1.00	0.00	C
ATOM	1063	C	MET	72	20.164	9.115	3.692	1.00	0.00	C
ATOM	1064	O	MET	72	19.175	9.312	4.399	1.00	0.00	O
ATOM	1065	CB	MET	72	21.296	6.989	4.369	1.00	0.00	C
ATOM	1066	CG	MET	72	21.198	5.473	4.296	1.00	0.00	C
ATOM	1067	SD	MET	72	21.283	4.694	5.920	1.00	0.00	S
ATOM	1068	CE	MET	72	19.675	5.121	6.585	1.00	0.00	C
ATOM	1069	H	MET	72	22.088	7.111	1.937	1.00	0.00	H
ATOM	1070	HA	MET	72	19.631	7.155	3.033	1.00	0.00	H
ATOM	1071	2HB3	MET	72	20.897	7.308	5.319	1.00	0.00	H
ATOM	1072	2HG3	MET	72	22.012	5.103	3.691	1.00	0.00	H
ATOM	1073	1HE	MET	72	19.435	6.140	6.319	1.00	0.00	H
ATOM	1074	2HE	MET	72	18.929	4.455	6.175	1.00	0.00	H
ATOM	1075	3HE	MET	72	19.695	5.024	7.661	1.00	0.00	H
ATOM	1076	HB2	MET	72	22.356	7.232	4.302	1.00	0.00	H
ATOM	1077	HG2	MET	72	20.242	5.191	3.857	1.00	0.00	H
ATOM	1078	N	ALA	73	20.961	10.092	3.276	1.00	0.00	N
ATOM	1079	CA	ALA	73	20.715	11.484	3.629	1.00	0.00	C
ATOM	1080	C	ALA	73	19.676	12.109	2.702	1.00	0.00	C
ATOM	1081	O	ALA	73	18.727	12.748	3.161	1.00	0.00	O
ATOM	1082	CB	ALA	73	22.011	12.280	3.584	1.00	0.00	C
ATOM	1083	H	ALA	73	21.735	9.871	2.716	1.00	0.00	H
ATOM	1084	HA	ALA	73	20.341	11.509	4.643	1.00	0.00	H
ATOM	1085	1HB	ALA	73	22.810	11.643	3.228	1.00	0.00	H
ATOM	1086	2HB	ALA	73	21.895	13.119	2.914	1.00	0.00	H
ATOM	1087	3HB	ALA	73	22.248	12.637	4.574	1.00	0.00	H
ATOM	1088	N	ARG	74	19.861	11.922	1.401	1.00	0.00	N
ATOM	1089	CA	ARG	74	18.940	12.469	0.411	1.00	0.00	C
ATOM	1090	C	ARG	74	17.665	11.635	0.336	1.00	0.00	C
ATOM	1091	O	ARG	74	16.575	12.166	0.121	1.00	0.00	O
ATOM	1092	CB	ARG	74	19.609	12.521	-0.963	1.00	0.00	C
ATOM	1093	CG	ARG	74	20.922	13.287	-0.975	1.00	0.00	C
ATOM	1094	CD	ARG	74	21.891	12.719	-1.999	1.00	0.00	C
ATOM	1095	NE	ARG	74	21.779	13.391	-3.290	1.00	0.00	N
ATOM	1096	CZ	ARG	74	22.432	14.507	-3.595	1.00	0.00	C
ATOM	1097	NH1	ARG	74	23.239	15.072	-2.709	1.00	0.00	N
ATOM	1098	NH2	ARG	74	22.278	15.060	-4.792	1.00	0.00	N
ATOM	1099	H	ARG	74	20.635	11.403	1.098	1.00	0.00	H
ATOM	1100	HA	ARG	74	18.684	13.471	0.716	1.00	0.00	H
ATOM	1101	2HB3	ARG	74	18.935	12.997	-1.660	1.00	0.00	H
ATOM	1102	2HG3	ARG	74	21.371	13.227	0.007	1.00	0.00	H
ATOM	1103	2HD3	ARG	74	21.679	11.668	-2.131	1.00	0.00	H
ATOM	1104	HE	ARG	74	21.188	12.991	-3.960	1.00	0.00	H
ATOM	1105	1HH1	ARG	74	23.357	14.657	-1.807	1.00	0.00	H
ATOM	1106	2HH1	ARG	74	23.729	15.913	-2.943	1.00	0.00	H
ATOM	1107	1HH2	ARG	74	21.672	14.638	-5.464	1.00	0.00	H
ATOM	1108	2HH2	ARG	74	22.769	15.899	-5.021	1.00	0.00	H
ATOM	1109	HB2	ARG	74	19.842	11.509	-1.295	1.00	0.00	H
ATOM	1110	HG2	ARG	74	20.733	14.328	-1.234	1.00	0.00	H
ATOM	1111	HD2	ARG	74	22.913	12.855	-1.647	1.00	0.00	H
ATOM	1112	N	LYS	75	17.806	10.327	0.514	1.00	0.00	N
ATOM	1113	CA	LYS	75	16.666	9.420	0.469	1.00	0.00	C
ATOM	1114	C	LYS	75	15.733	9.658	1.652	1.00	0.00	C
ATOM	1115	O	LYS	75	14.593	9.194	1.656	1.00	0.00	O
ATOM	1116	CB	LYS	75	17.147	7.965	0.468	1.00	0.00	C
ATOM	1117	CG	LYS	75	16.022	6.954	0.317	1.00	0.00	C
ATOM	1118	CD	LYS	75	15.327	7.092	-1.028	1.00	0.00	C
ATOM	1119	CE	LYS	75	16.234	6.665	-2.171	1.00	0.00	C
ATOM	1120	NZ	LYS	75	17.032	7.805	-2.701	1.00	0.00	N
ATOM	1121	H	LYS	75	18.701	9.963	0.682	1.00	0.00	H
ATOM	1122	HA	LYS	75	16.127	9.610	-0.448	1.00	0.00	H
ATOM	1123	2HB3	LYS	75	17.657	7.768	1.400	1.00	0.00	H
ATOM	1124	2HG3	LYS	75	15.299	7.115	1.105	1.00	0.00	H
ATOM	1125	2HD3	LYS	75	15.042	8.124	-1.171	1.00	0.00	H
ATOM	1126	2HE3	LYS	75	15.624	6.262	-2.966	1.00	0.00	H
ATOM	1127	1HZ	LYS	75	16.582	8.187	-3.556	1.00	0.00	H
ATOM	1128	2HZ	LYS	75	17.992	7.487	-2.942	1.00	0.00	H
ATOM	1129	3HZ	LYS	75	17.096	8.558	-1.989	1.00	0.00	H
ATOM	1130	HB2	LYS	75	17.820	7.804	-0.374	1.00	0.00	H
ATOM	1131	HG2	LYS	75	16.428	5.946	0.382	1.00	0.00	H
ATOM	1132	HD2	LYS	75	14.443	6.453	-1.048	1.00	0.00	H
ATOM	1133	HE2	LYS	75	16.935	5.909	-1.816	1.00	0.00	H
ATOM	1134	N	MET	76	16.224	10.385	2.648	1.00	0.00	N
ATOM	1135	CA	MET	76	15.432	10.687	3.835	1.00	0.00	C

ATOM	1136	C	MET	76	14.319	11.677	3.509	1.00	0.00	C
ATOM	1137	O	MET	76	13.246	11.644	4.113	1.00	0.00	O
ATOM	1138	CB	MET	76	16.324	11.253	4.941	1.00	0.00	C
ATOM	1139	CG	MET	76	16.778	10.211	5.950	1.00	0.00	C
ATOM	1140	SD	MET	76	15.415	9.548	6.926	1.00	0.00	S
ATOM	1141	CE	MET	76	15.239	7.918	6.202	1.00	0.00	C
ATOM	1142	H	MET	76	17.140	10.727	2.586	1.00	0.00	H
ATOM	1143	HA	MET	76	14.987	9.766	4.179	1.00	0.00	H
ATOM	1144	2HB3	MET	76	15.779	12.019	5.471	1.00	0.00	H
ATOM	1145	2HG3	MET	76	17.495	10.663	6.617	1.00	0.00	H
ATOM	1146	1HE	MET	76	15.156	8.009	5.129	1.00	0.00	H
ATOM	1147	2HE	MET	76	16.105	7.321	6.446	1.00	0.00	H
ATOM	1148	3HE	MET	76	14.351	7.446	6.593	1.00	0.00	H
ATOM	1149	HB2	MET	76	17.231	11.668	4.500	1.00	0.00	H
ATOM	1150	HG2	MET	76	17.228	9.367	5.425	1.00	0.00	H
ATOM	1151	N	LYS	77	14.578	12.557	2.547	1.00	0.00	N
ATOM	1152	CA	LYS	77	13.600	13.557	2.139	1.00	0.00	C
ATOM	1153	C	LYS	77	12.686	13.008	1.046	1.00	0.00	C
ATOM	1154	O	LYS	77	11.513	13.371	0.964	1.00	0.00	O
ATOM	1155	CB	LYS	77	14.307	14.820	1.640	1.00	0.00	C
ATOM	1156	CG	LYS	77	14.757	14.733	0.190	1.00	0.00	C
ATOM	1157	CD	LYS	77	13.676	15.230	-0.757	1.00	0.00	C
ATOM	1158	CE	LYS	77	13.602	16.749	-0.770	1.00	0.00	C
ATOM	1159	NZ	LYS	77	14.773	17.356	-1.463	1.00	0.00	N
ATOM	1160	H	LYS	77	15.452	12.534	2.101	1.00	0.00	H
ATOM	1161	HA	LYS	77	13.000	13.807	3.000	1.00	0.00	H
ATOM	1162	2HB3	LYS	77	15.177	14.994	2.255	1.00	0.00	H
ATOM	1163	2HG3	LYS	77	14.984	13.704	-0.045	1.00	0.00	H
ATOM	1164	2HD3	LYS	77	12.721	14.833	-0.442	1.00	0.00	H
ATOM	1165	2HE3	LYS	77	13.575	17.104	0.249	1.00	0.00	H
ATOM	1166	1HZ	LYS	77	15.378	17.849	-0.775	1.00	0.00	H
ATOM	1167	2HZ	LYS	77	14.450	18.040	-2.175	1.00	0.00	H
ATOM	1168	3HZ	LYS	77	15.331	16.617	-1.936	1.00	0.00	H
ATOM	1169	HB2	LYS	77	13.623	15.667	1.699	1.00	0.00	H
ATOM	1170	HG2	LYS	77	15.641	15.354	0.047	1.00	0.00	H
ATOM	1171	HD2	LYS	77	13.901	14.898	-1.770	1.00	0.00	H
ATOM	1172	HE2	LYS	77	12.705	17.065	-1.302	1.00	0.00	H
ATOM	1173	N	ASP	78	13.231	12.132	0.210	1.00	0.00	N
ATOM	1174	CA	ASP	78	12.464	11.531	-0.875	1.00	0.00	C
ATOM	1175	C	ASP	78	11.198	10.869	-0.344	1.00	0.00	C
ATOM	1176	O	ASP	78	10.169	10.838	-1.021	1.00	0.00	O
ATOM	1177	CB	ASP	78	13.317	10.503	-1.622	1.00	0.00	C
ATOM	1178	CG	ASP	78	14.184	11.138	-2.694	1.00	0.00	C
ATOM	1179	OD1	ASP	78	14.617	12.293	-2.498	1.00	0.00	O
ATOM	1180	OD2	ASP	78	14.425	10.480	-3.728	1.00	0.00	O
ATOM	1181	H	ASP	78	14.172	11.881	0.328	1.00	0.00	H
ATOM	1182	HA	ASP	78	12.185	12.319	-1.559	1.00	0.00	H
ATOM	1183	2HB3	ASP	78	12.668	9.780	-2.092	1.00	0.00	H
ATOM	1184	HB2	ASP	78	13.984	10.003	-0.919	1.00	0.00	H
ATOM	1185	N	THR	79	11.279	10.338	0.873	1.00	0.00	N
ATOM	1186	CA	THR	79	10.141	9.675	1.494	1.00	0.00	C
ATOM	1187	C	THR	79	9.213	10.683	2.163	1.00	0.00	C
ATOM	1188	O	THR	79	8.049	10.386	2.435	1.00	0.00	O
ATOM	1189	CB	THR	79	10.596	8.640	2.542	1.00	0.00	C
ATOM	1190	OG1	THR	79	11.425	7.650	1.923	1.00	0.00	O
ATOM	1191	CG2	THR	79	9.397	7.969	3.196	1.00	0.00	C
ATOM	1192	H	THR	79	12.126	10.394	1.362	1.00	0.00	H
ATOM	1193	HA	THR	79	9.593	9.155	0.720	1.00	0.00	H
ATOM	1194	HB	THR	79	11.165	9.150	3.305	1.00	0.00	H
ATOM	1195	1HG	THR	79	11.755	7.985	1.087	1.00	0.00	H
ATOM	1196	1HG2	THR	79	9.740	7.201	3.872	1.00	0.00	H
ATOM	1197	2HG2	THR	79	8.773	7.526	2.436	1.00	0.00	H
ATOM	1198	3HG2	THR	79	8.830	8.704	3.746	1.00	0.00	H
ATOM	1199	N	ASP	80	9.736	11.877	2.425	1.00	0.00	N
ATOM	1200	CA	ASP	80	8.953	12.929	3.059	1.00	0.00	C
ATOM	1201	C	ASP	80	7.538	12.972	2.494	1.00	0.00	C
ATOM	1202	O	ASP	80	6.585	13.296	3.202	1.00	0.00	O
ATOM	1203	CB	ASP	80	9.634	14.286	2.867	1.00	0.00	C
ATOM	1204	CG	ASP	80	9.239	15.289	3.934	1.00	0.00	C
ATOM	1205	OD1	ASP	80	8.186	15.941	3.774	1.00	0.00	O
ATOM	1206	OD2	ASP	80	9.983	15.420	4.927	1.00	0.00	O
ATOM	1207	H	ASP	80	10.669	12.052	2.184	1.00	0.00	H
ATOM	1208	HA	ASP	80	8.899	12.713	4.116	1.00	0.00	H
ATOM	1209	2HB3	ASP	80	9.358	14.686	1.903	1.00	0.00	H
ATOM	1210	HB2	ASP	80	10.717	14.162	2.920	1.00	0.00	H
ATOM	1211	N	SER	81	7.407	12.645	1.213	1.00	0.00	N
ATOM	1212	CA	SER	81	6.110	12.649	0.550	1.00	0.00	C

ATOM	1213	C	SER	81	5.019	12.136	1.486	1.00	0.00	C
ATOM	1214	O	SER	81	3.859	12.536	1.384	1.00	0.00	O
ATOM	1215	CB	SER	81	6.154	11.792	-0.716	1.00	0.00	C
ATOM	1216	OG	SER	81	7.103	12.298	-1.640	1.00	0.00	O
ATOM	1217	H	SER	81	8.206	12.396	0.699	1.00	0.00	H
ATOM	1218	HA	SER	81	5.879	13.668	0.276	1.00	0.00	H
ATOM	1219	2HB3	SER	81	5.180	11.791	-1.184	1.00	0.00	H
ATOM	1220	HG	SER	81	7.909	11.779	-1.588	1.00	0.00	H
ATOM	1221	HB2	SER	81	6.434	10.771	-0.454	1.00	0.00	H
ATOM	1222	N	GLU	82	5.401	11.246	2.397	1.00	0.00	N
ATOM	1223	CA	GLU	82	4.457	10.676	3.352	1.00	0.00	C
ATOM	1224	C	GLU	82	3.383	11.693	3.727	1.00	0.00	C
ATOM	1225	O	GLU	82	2.190	11.415	3.621	1.00	0.00	O
ATOM	1226	CB	GLU	82	5.191	10.205	4.610	1.00	0.00	C
ATOM	1227	CG	GLU	82	4.268	9.626	5.669	1.00	0.00	C
ATOM	1228	CD	GLU	82	4.974	9.399	6.994	1.00	0.00	C
ATOM	1229	OE1	GLU	82	5.945	10.130	7.283	1.00	0.00	O
ATOM	1230	OE2	GLU	82	4.553	8.491	7.740	1.00	0.00	O
ATOM	1231	H	GLU	82	6.341	10.967	2.429	1.00	0.00	H
ATOM	1232	HA	GLU	82	3.984	9.827	2.882	1.00	0.00	H
ATOM	1233	2HB3	GLU	82	5.717	11.044	5.039	1.00	0.00	H
ATOM	1234	2HG3	GLU	82	3.886	8.680	5.316	1.00	0.00	H
ATOM	1235	HB2	GLU	82	5.887	9.410	4.348	1.00	0.00	H
ATOM	1236	HG2	GLU	82	3.447	10.319	5.855	1.00	0.00	H
ATOM	1237	N	GLU	83	3.817	12.870	4.166	1.00	0.00	N
ATOM	1238	CA	GLU	83	2.894	13.926	4.558	1.00	0.00	C
ATOM	1239	C	GLU	83	1.952	14.281	3.410	1.00	0.00	C
ATOM	1240	O	GLU	83	0.731	14.285	3.574	1.00	0.00	O
ATOM	1241	CB	GLU	83	3.665	15.172	5.001	1.00	0.00	C
ATOM	1242	CG	GLU	83	4.589	14.926	6.181	1.00	0.00	C
ATOM	1243	CD	GLU	83	4.788	16.167	7.034	1.00	0.00	C
ATOM	1244	OE1	GLU	83	4.902	17.269	6.459	1.00	0.00	O
ATOM	1245	OE2	GLU	83	4.832	16.032	8.275	1.00	0.00	O
ATOM	1246	H	GLU	83	4.782	13.031	4.228	1.00	0.00	H
ATOM	1247	HA	GLU	83	2.307	13.564	5.390	1.00	0.00	H
ATOM	1248	2HB3	GLU	83	2.957	15.939	5.278	1.00	0.00	H
ATOM	1249	2HG3	GLU	83	5.550	14.604	5.811	1.00	0.00	H
ATOM	1250	HB2	GLU	83	4.300	15.517	4.185	1.00	0.00	H
ATOM	1251	HG2	GLU	83	4.159	14.161	6.827	1.00	0.00	H
ATOM	1252	N	GLU	84	2.528	14.575	2.251	1.00	0.00	N
ATOM	1253	CA	GLU	84	1.741	14.931	1.074	1.00	0.00	C
ATOM	1254	C	GLU	84	0.483	14.071	0.983	1.00	0.00	C
ATOM	1255	O	GLU	84	-0.531	14.495	0.426	1.00	0.00	O
ATOM	1256	CB	GLU	84	2.579	14.770	-0.196	1.00	0.00	C
ATOM	1257	CG	GLU	84	3.699	15.786	-0.319	1.00	0.00	C
ATOM	1258	CD	GLU	84	3.195	17.174	-0.666	1.00	0.00	C
ATOM	1259	OE1	GLU	84	3.033	17.461	-1.872	1.00	0.00	O
ATOM	1260	OE2	GLU	84	2.962	17.971	0.265	1.00	0.00	O
ATOM	1261	H	GLU	84	3.505	14.554	2.182	1.00	0.00	H
ATOM	1262	HA	GLU	84	1.447	15.965	1.173	1.00	0.00	H
ATOM	1263	2HB3	GLU	84	1.931	14.874	-1.054	1.00	0.00	H
ATOM	1264	2HG3	GLU	84	4.380	15.463	-1.092	1.00	0.00	H
ATOM	1265	HB2	GLU	84	3.055	13.792	-0.195	1.00	0.00	H
ATOM	1266	HG2	GLU	84	4.226	15.864	0.633	1.00	0.00	H
ATOM	1267	N	ILE	85	0.558	12.862	1.527	1.00	0.00	N
ATOM	1268	CA	ILE	85	-0.571	11.942	1.506	1.00	0.00	C
ATOM	1269	C	ILE	85	-1.482	12.165	2.708	1.00	0.00	C
ATOM	1270	O	ILE	85	-2.706	12.059	2.602	1.00	0.00	O
ATOM	1271	CB	ILE	85	-0.103	10.475	1.493	1.00	0.00	C
ATOM	1272	CG2	ILE	85	-1.298	9.539	1.384	1.00	0.00	C
ATOM	1273	CG1	ILE	85	0.874	10.238	0.342	1.00	0.00	C
ATOM	1274	CD1	ILE	85	1.487	8.856	0.343	1.00	0.00	C
ATOM	1275	H	ILE	85	1.395	12.582	1.955	1.00	0.00	H
ATOM	1276	HA	ILE	85	-1.134	12.128	0.603	1.00	0.00	H
ATOM	1277	HB	ILE	85	0.397	10.272	2.430	1.00	0.00	H
ATOM	1278	1HG2	ILE	85	-1.965	9.897	0.612	1.00	0.00	H
ATOM	1279	2HG2	ILE	85	-0.956	8.547	1.130	1.00	0.00	H
ATOM	1280	3HG2	ILE	85	-1.821	9.510	2.327	1.00	0.00	H
ATOM	1281	2HG3	ILE	85	1.677	10.957	0.407	1.00	0.00	H
ATOM	1282	1HD1	ILE	85	1.059	8.272	1.146	1.00	0.00	H
ATOM	1283	2HD1	ILE	85	1.286	8.370	-0.599	1.00	0.00	H
ATOM	1284	3HD1	ILE	85	2.556	8.934	0.488	1.00	0.00	H
ATOM	1285	HG2	ILE	85	0.351	10.352	-0.607	1.00	0.00	H
ATOM	1286	N	ARG	86	-0.880	12.473	3.853	1.00	0.00	N
ATOM	1287	CA	ARG	86	-1.636	12.709	5.075	1.00	0.00	C
ATOM	1288	C	ARG	86	-2.902	13.511	4.784	1.00	0.00	C
ATOM	1289	O	ARG	86	-4.010	13.064	5.074	1.00	0.00	O

ATOM	1290	CB	ARG	86	-0.775	13.452	6.099	1.00	0.00	C
ATOM	1291	CG	ARG	86	0.266	12.570	6.772	1.00	0.00	C
ATOM	1292	CD	ARG	86	-0.274	11.952	8.052	1.00	0.00	C
ATOM	1293	NE	ARG	86	-0.596	12.965	9.054	1.00	0.00	N
ATOM	1294	CZ	ARG	86	-0.625	12.726	10.360	1.00	0.00	C
ATOM	1295	NH1	ARG	86	-0.350	11.513	10.821	1.00	0.00	N
ATOM	1296	NH2	ARG	86	-0.929	13.700	11.210	1.00	0.00	N
ATOM	1297	H	ARG	86	0.098	12.542	3.873	1.00	0.00	H
ATOM	1298	HA	ARG	86	-1.918	11.751	5.483	1.00	0.00	H
ATOM	1299	2HB3	ARG	86	-1.415	13.862	6.863	1.00	0.00	H
ATOM	1300	2HG3	ARG	86	1.133	13.168	7.006	1.00	0.00	H
ATOM	1301	2HD3	ARG	86	0.473	11.286	8.457	1.00	0.00	H
ATOM	1302	HE	ARG	86	-0.804	13.868	8.736	1.00	0.00	H
ATOM	1303	1HH1	ARG	86	-0.121	10.777	10.183	1.00	0.00	H
ATOM	1304	2HH1	ARG	86	-0.371	11.335	11.805	1.00	0.00	H
ATOM	1305	1HH2	ARG	86	-1.136	14.613	10.866	1.00	0.00	H
ATOM	1306	2HH2	ARG	86	-0.949	13.517	12.191	1.00	0.00	H
ATOM	1307	HB2	ARG	86	-0.221	14.247	5.599	1.00	0.00	H
ATOM	1308	HG2	ARG	86	0.543	11.758	6.099	1.00	0.00	H
ATOM	1309	HD2	ARG	86	-1.190	11.405	7.835	1.00	0.00	H
ATOM	1310	N	GLU	87	-2.725	14.699	4.211	1.00	0.00	N
ATOM	1311	CA	GLU	87	-3.853	15.563	3.883	1.00	0.00	C
ATOM	1312	C	GLU	87	-5.057	14.739	3.435	1.00	0.00	C
ATOM	1313	O	GLU	87	-6.161	14.902	3.954	1.00	0.00	O
ATOM	1314	CB	GLU	87	-3.463	16.554	2.785	1.00	0.00	C
ATOM	1315	CG	GLU	87	-2.404	17.554	3.217	1.00	0.00	C
ATOM	1316	CD	GLU	87	-2.980	18.701	4.023	1.00	0.00	C
ATOM	1317	OE1	GLU	87	-3.739	19.509	3.449	1.00	0.00	O
ATOM	1318	OE2	GLU	87	-2.671	18.791	5.231	1.00	0.00	O
ATOM	1319	H	GLU	87	-1.817	15.000	4.005	1.00	0.00	H
ATOM	1320	HA	GLU	87	-4.121	16.114	4.772	1.00	0.00	H
ATOM	1321	2HB3	GLU	87	-4.343	17.101	2.483	1.00	0.00	H
ATOM	1322	2HG3	GLU	87	-1.928	17.958	2.335	1.00	0.00	H
ATOM	1323	HB2	GLU	87	-3.043	16.011	1.938	1.00	0.00	H
ATOM	1324	HG2	GLU	87	-1.667	17.055	3.846	1.00	0.00	H
ATOM	1325	N	ALA	88	-4.836	13.854	2.469	1.00	0.00	N
ATOM	1326	CA	ALA	88	-5.902	13.004	1.954	1.00	0.00	C
ATOM	1327	C	ALA	88	-6.566	12.214	3.074	1.00	0.00	C
ATOM	1328	O	ALA	88	-7.792	12.192	3.190	1.00	0.00	O
ATOM	1329	CB	ALA	88	-5.355	12.059	0.892	1.00	0.00	C
ATOM	1330	H	ALA	88	-3.934	13.769	2.097	1.00	0.00	H
ATOM	1331	HA	ALA	88	-6.639	13.640	1.488	1.00	0.00	H
ATOM	1332	1HB	ALA	88	-4.410	11.655	1.225	1.00	0.00	H
ATOM	1333	2HB	ALA	88	-6.056	11.255	0.732	1.00	0.00	H
ATOM	1334	3HB	ALA	88	-5.210	12.600	-0.030	1.00	0.00	H
ATOM	1335	N	PHE	89	-5.752	11.565	3.902	1.00	0.00	N
ATOM	1336	CA	PHE	89	-6.261	10.774	5.013	1.00	0.00	C
ATOM	1337	C	PHE	89	-7.240	11.586	5.855	1.00	0.00	C
ATOM	1338	O	PHE	89	-8.293	11.087	6.255	1.00	0.00	O
ATOM	1339	CB	PHE	89	-5.105	10.280	5.888	1.00	0.00	C
ATOM	1340	CG	PHE	89	-5.511	9.981	7.303	1.00	0.00	C
ATOM	1341	CD1	PHE	89	-6.134	8.788	7.621	1.00	0.00	C
ATOM	1342	CE1	PHE	89	-6.509	8.511	8.923	1.00	0.00	C
ATOM	1343	CZ	PHE	89	-6.259	9.432	9.923	1.00	0.00	C
ATOM	1344	CE2	PHE	89	-5.636	10.626	9.618	1.00	0.00	C
ATOM	1345	CD2	PHE	89	-5.266	10.897	8.314	1.00	0.00	C
ATOM	1346	H	PHE	89	-4.782	11.623	3.756	1.00	0.00	H
ATOM	1347	HA	PHE	89	-6.779	9.920	4.602	1.00	0.00	H
ATOM	1348	2HB3	PHE	89	-4.336	11.037	5.913	1.00	0.00	H
ATOM	1349	1HD	PHE	89	-6.330	8.067	6.842	1.00	0.00	H
ATOM	1350	1HE	PHE	89	-6.994	7.576	9.160	1.00	0.00	H
ATOM	1351	HZ	PHE	89	-6.550	9.218	10.940	1.00	0.00	H
ATOM	1352	2HE	PHE	89	-5.441	11.347	10.396	1.00	0.00	H
ATOM	1353	2HD	PHE	89	-4.779	11.834	8.077	1.00	0.00	H
ATOM	1354	HB2	PHE	89	-4.708	9.353	5.477	1.00	0.00	H
ATOM	1355	N	ARG	90	-6.885	12.839	6.120	1.00	0.00	N
ATOM	1356	CA	ARG	90	-7.732	13.720	6.916	1.00	0.00	C
ATOM	1357	C	ARG	90	-9.118	13.854	6.292	1.00	0.00	C
ATOM	1358	O	ARG	90	-10.132	13.647	6.957	1.00	0.00	O
ATOM	1359	CB	ARG	90	-7.085	15.099	7.049	1.00	0.00	C
ATOM	1360	CG	ARG	90	-5.864	15.117	7.952	1.00	0.00	C
ATOM	1361	CD	ARG	90	-4.958	16.298	7.642	1.00	0.00	C
ATOM	1362	NE	ARG	90	-4.040	16.586	8.742	1.00	0.00	N
ATOM	1363	CZ	ARG	90	-4.363	17.332	9.793	1.00	0.00	C
ATOM	1364	NH1	ARG	90	-5.575	17.861	9.885	1.00	0.00	N
ATOM	1365	NH2	ARG	90	-3.474	17.550	10.752	1.00	0.00	N
ATOM	1366	H	ARG	90	-6.035	13.178	5.772	1.00	0.00	H

```
ATOM   1367  HA  ARG    90      -7.834  13.284   7.899  1.00  0.00           H
ATOM   1368 2HB3 ARG    90      -7.815  15.788   7.452  1.00  0.00           H
ATOM   1369 2HG3 ARG    90      -5.310  14.200   7.810  1.00  0.00           H
ATOM   1370 2HD3 ARG    90      -5.570  17.167   7.460  1.00  0.00           H
ATOM   1371  HE  ARG    90      -3.138  16.206   8.693  1.00  0.00           H
ATOM   1372 1HH1 ARG    90      -6.249  17.696   9.165  1.00  0.00           H
ATOM   1373 2HH1 ARG    90      -5.817  18.421  10.679  1.00  0.00           H
ATOM   1374 1HH2 ARG    90      -2.558  17.152  10.685  1.00  0.00           H
ATOM   1375 2HH2 ARG    90      -3.719  18.111  11.541  1.00  0.00           H
ATOM   1376  HB2 ARG    90      -6.745  15.438   6.070  1.00  0.00           H
ATOM   1377  HG2 ARG    90      -6.181  15.203   8.992  1.00  0.00           H
ATOM   1378  HD2 ARG    90      -4.354  16.071   6.763  1.00  0.00           H
ATOM   1379  N   VAL    91      -9.153  14.199   5.009  1.00  0.00           N
ATOM   1380  CA  VAL    91     -10.413  14.360   4.294  1.00  0.00           C
ATOM   1381  C   VAL    91     -11.371  13.217   4.609  1.00  0.00           C
ATOM   1382  O   VAL    91     -12.509  13.442   5.023  1.00  0.00           O
ATOM   1383  CB  VAL    91     -10.191  14.426   2.772  1.00  0.00           C
ATOM   1384  CG1 VAL    91     -11.520  14.536   2.042  1.00  0.00           C
ATOM   1385  CG2 VAL    91      -9.278  15.591   2.416  1.00  0.00           C
ATOM   1386  H   VAL    91      -8.309  14.351   4.532  1.00  0.00           H
ATOM   1387  HA  VAL    91     -10.861  15.291   4.610  1.00  0.00           H
ATOM   1388  HB  VAL    91      -9.709  13.511   2.459  1.00  0.00           H
ATOM   1389 1HG1 VAL    91     -12.086  15.360   2.448  1.00  0.00           H
ATOM   1390 2HG1 VAL    91     -11.340  14.704   0.989  1.00  0.00           H
ATOM   1391 3HG1 VAL    91     -12.077  13.620   2.170  1.00  0.00           H
ATOM   1392 1HG2 VAL    91      -9.757  16.206   1.667  1.00  0.00           H
ATOM   1393 2HG2 VAL    91      -9.089  16.182   3.300  1.00  0.00           H
ATOM   1394 3HG2 VAL    91      -8.345  15.214   2.028  1.00  0.00           H
ATOM   1395  N   PHE    92     -10.906  11.990   4.404  1.00  0.00           N
ATOM   1396  CA  PHE    92     -11.723  10.810   4.666  1.00  0.00           C
ATOM   1397  C   PHE    92     -12.181  10.776   6.121  1.00  0.00           C
ATOM   1398  O   PHE    92     -13.360  10.571   6.408  1.00  0.00           O
ATOM   1399  CB  PHE    92     -10.938   9.538   4.336  1.00  0.00           C
ATOM   1400  CG  PHE    92     -10.870   9.241   2.865  1.00  0.00           C
ATOM   1401  CD1 PHE    92      -9.886   9.814   2.075  1.00  0.00           C
ATOM   1402  CE1 PHE    92      -9.820   9.543   0.722  1.00  0.00           C
ATOM   1403  CZ  PHE    92     -10.740   8.691   0.143  1.00  0.00           C
ATOM   1404  CE2 PHE    92     -11.725   8.113   0.919  1.00  0.00           C
ATOM   1405  CD2 PHE    92     -11.786   8.388   2.272  1.00  0.00           C
ATOM   1406  H   PHE    92      -9.992  11.874   4.072  1.00  0.00           H
ATOM   1407  HA  PHE    92     -12.592  10.861   4.028  1.00  0.00           H
ATOM   1408 2HB3 PHE    92     -11.410   8.697   4.824  1.00  0.00           H
ATOM   1409 1HD  PHE    92      -9.166  10.481   2.526  1.00  0.00           H
ATOM   1410 1HE  PHE    92      -9.049   9.997   0.116  1.00  0.00           H
ATOM   1411  HZ  PHE    92     -10.691   8.476  -0.914  1.00  0.00           H
ATOM   1412 2HE  PHE    92     -12.447   7.445   0.470  1.00  0.00           H
ATOM   1413 2HD  PHE    92     -12.557   7.934   2.880  1.00  0.00           H
ATOM   1414  HB2 PHE    92      -9.912   9.643   4.684  1.00  0.00           H
ATOM   1415  N   ASP    93     -11.238  10.977   7.036  1.00  0.00           N
ATOM   1416  CA  ASP    93     -11.543  10.969   8.462  1.00  0.00           C
ATOM   1417  C   ASP    93     -12.412  12.166   8.838  1.00  0.00           C
ATOM   1418  O   ASP    93     -11.909  13.269   9.052  1.00  0.00           O
ATOM   1419  CB  ASP    93     -10.251  10.985   9.281  1.00  0.00           C
ATOM   1420  CG  ASP    93     -10.456  10.466  10.691  1.00  0.00           C
ATOM   1421  OD1 ASP    93     -11.329   9.597  10.883  1.00  0.00           O
ATOM   1422  OD2 ASP    93      -9.740  10.931  11.604  1.00  0.00           O
ATOM   1423  H   ASP    93     -10.316  11.135   6.745  1.00  0.00           H
ATOM   1424  HA  ASP    93     -12.087  10.063   8.681  1.00  0.00           H
ATOM   1425 2HB3 ASP    93      -9.881  11.998   9.339  1.00  0.00           H
ATOM   1426  HB2 ASP    93      -9.511  10.343   8.804  1.00  0.00           H
ATOM   1427  N   LYS    94     -13.719  11.941   8.914  1.00  0.00           N
ATOM   1428  CA  LYS    94     -14.659  13.000   9.263  1.00  0.00           C
ATOM   1429  C   LYS    94     -14.490  13.418  10.720  1.00  0.00           C
ATOM   1430  O   LYS    94     -14.131  14.561  11.009  1.00  0.00           O
ATOM   1431  CB  LYS    94     -16.097  12.532   9.019  1.00  0.00           C
ATOM   1432  CG  LYS    94     -16.582  12.770   7.598  1.00  0.00           C
ATOM   1433  CD  LYS    94     -18.083  12.982   7.552  1.00  0.00           C
ATOM   1434  CE  LYS    94     -18.827  11.668   7.358  1.00  0.00           C
ATOM   1435  NZ  LYS    94     -20.219  11.885   6.877  1.00  0.00           N
ATOM   1436  H   LYS    94     -14.060  11.040   8.730  1.00  0.00           H
ATOM   1437  HA  LYS    94     -14.455  13.849   8.630  1.00  0.00           H
ATOM   1438 2HB3 LYS    94     -16.753  13.064   9.695  1.00  0.00           H
ATOM   1439 2HG3 LYS    94     -16.328  11.909   6.996  1.00  0.00           H
ATOM   1440 2HD3 LYS    94     -18.319  13.643   6.727  1.00  0.00           H
ATOM   1441 2HE3 LYS    94     -18.857  11.145   8.303  1.00  0.00           H
ATOM   1442 1HZ  LYS    94     -20.289  11.653   5.866  1.00  0.00           H
ATOM   1443 2HZ  LYS    94     -20.494  12.879   7.013  1.00  0.00           H
```

ATOM	1444	3HZ	LYS	94	-20.880	11.279	7.407	1.00	0.00	H
ATOM	1445	HB2	LYS	94	-16.166	11.459	9.192	1.00	0.00	H
ATOM	1446	HG2	LYS	94	-16.102	13.663	7.195	1.00	0.00	H
ATOM	1447	HD2	LYS	94	-18.418	13.427	8.489	1.00	0.00	H
ATOM	1448	HE2	LYS	94	-18.311	11.066	6.610	1.00	0.00	H
ATOM	1449	N	ASP	95	-14.748	12.487	11.633	1.00	0.00	N
ATOM	1450	CA	ASP	95	-14.620	12.760	13.059	1.00	0.00	C
ATOM	1451	C	ASP	95	-13.252	13.357	13.379	1.00	0.00	C
ATOM	1452	O	ASP	95	-13.144	14.312	14.146	1.00	0.00	O
ATOM	1453	CB	ASP	95	-14.835	11.480	13.866	1.00	0.00	C
ATOM	1454	CG	ASP	95	-13.764	10.442	13.601	1.00	0.00	C
ATOM	1455	OD1	ASP	95	-13.230	10.416	12.471	1.00	0.00	O
ATOM	1456	OD2	ASP	95	-13.457	9.655	14.521	1.00	0.00	O
ATOM	1457	H	ASP	95	-15.030	11.597	11.339	1.00	0.00	H
ATOM	1458	HA	ASP	95	-15.383	13.477	13.329	1.00	0.00	H
ATOM	1459	2HB3	ASP	95	-15.793	11.056	13.606	1.00	0.00	H
ATOM	1460	HB2	ASP	95	-14.804	11.710	14.931	1.00	0.00	H
ATOM	1461	N	GLY	96	-12.210	12.780	12.787	1.00	0.00	N
ATOM	1462	CA	GLY	96	-10.863	13.267	13.023	1.00	0.00	C
ATOM	1463	C	GLY	96	-10.234	12.657	14.259	1.00	0.00	C
ATOM	1464	O	GLY	96	-9.627	13.360	15.066	1.00	0.00	O
ATOM	1465	H	GLY	96	-12.357	12.022	12.186	1.00	0.00	H
ATOM	1466	2HA	GLY	96	-10.251	13.028	12.165	1.00	0.00	H
ATOM	1467	3HA	GLY	96	-10.896	14.339	13.142	1.00	0.00	H
ATOM	1468	N	ASN	97	-10.383	11.346	14.411	1.00	0.00	N
ATOM	1469	CA	ASN	97	-9.827	10.640	15.560	1.00	0.00	C
ATOM	1470	C	ASN	97	-8.566	9.873	15.170	1.00	0.00	C
ATOM	1471	O	ASN	97	-7.564	9.903	15.883	1.00	0.00	O
ATOM	1472	CB	ASN	97	-10.863	9.679	16.145	1.00	0.00	C
ATOM	1473	CG	ASN	97	-10.981	8.397	15.344	1.00	0.00	C
ATOM	1474	OD1	ASN	97	-11.354	8.418	14.172	1.00	0.00	O
ATOM	1475	ND2	ASN	97	-10.659	7.273	15.975	1.00	0.00	N
ATOM	1476	H	ASN	97	-10.879	10.839	13.735	1.00	0.00	H
ATOM	1477	HA	ASN	97	-9.569	11.377	16.307	1.00	0.00	H
ATOM	1478	2HB3	ASN	97	-11.828	10.164	16.160	1.00	0.00	H
ATOM	1479	1HD2	ASN	97	-10.368	7.333	16.909	1.00	0.00	H
ATOM	1480	2HD2	ASN	97	-10.725	6.429	15.481	1.00	0.00	H
ATOM	1481	HB2	ASN	97	-10.571	9.401	17.158	1.00	0.00	H
ATOM	1482	N	GLY	98	-8.626	9.187	14.033	1.00	0.00	N
ATOM	1483	CA	GLY	98	-7.484	8.420	13.569	1.00	0.00	C
ATOM	1484	C	GLY	98	-7.884	7.282	12.652	1.00	0.00	C
ATOM	1485	O	GLY	98	-7.218	7.019	11.651	1.00	0.00	O
ATOM	1486	H	GLY	98	-9.454	9.199	13.508	1.00	0.00	H
ATOM	1487	2HA	GLY	98	-6.816	9.080	13.036	1.00	0.00	H
ATOM	1488	3HA	GLY	98	-6.967	8.014	14.425	1.00	0.00	H
ATOM	1489	N	TYR	99	-8.974	6.604	12.993	1.00	0.00	N
ATOM	1490	CA	TYR	99	-9.461	5.485	12.195	1.00	0.00	C
ATOM	1491	C	TYR	99	-10.619	5.916	11.300	1.00	0.00	C
ATOM	1492	O	TYR	99	-11.532	6.614	11.741	1.00	0.00	O
ATOM	1493	CB	TYR	99	-9.904	4.338	13.104	1.00	0.00	C
ATOM	1494	CG	TYR	99	-8.752	3.571	13.716	1.00	0.00	C
ATOM	1495	CD1	TYR	99	-7.812	4.211	14.513	1.00	0.00	C
ATOM	1496	CE1	TYR	99	-6.758	3.515	15.071	1.00	0.00	C
ATOM	1497	CZ	TYR	99	-6.637	2.161	14.842	1.00	0.00	C
ATOM	1498	CE2	TYR	99	-7.560	1.502	14.056	1.00	0.00	C
ATOM	1499	CD2	TYR	99	-8.609	2.207	13.499	1.00	0.00	C
ATOM	1500	OH	TYR	99	-5.590	1.462	15.400	1.00	0.00	O
ATOM	1501	H	TYR	99	-9.465	6.862	13.801	1.00	0.00	H
ATOM	1502	HA	TYR	99	-8.648	5.144	11.572	1.00	0.00	H
ATOM	1503	2HB3	TYR	99	-10.498	3.642	12.531	1.00	0.00	H
ATOM	1504	1HD	TYR	99	-7.911	5.272	14.690	1.00	0.00	H
ATOM	1505	1HE	TYR	99	-6.035	4.030	15.687	1.00	0.00	H
ATOM	1506	2HE	TYR	99	-7.464	0.441	13.877	1.00	0.00	H
ATOM	1507	2HD	TYR	99	-9.333	1.694	12.882	1.00	0.00	H
ATOM	1508	HH	TYR	99	-4.780	1.971	15.306	1.00	0.00	H
ATOM	1509	HB2	TYR	99	-10.491	4.734	13.932	1.00	0.00	H
ATOM	1510	N	ILE	100	-10.572	5.494	10.039	1.00	0.00	N
ATOM	1511	CA	ILE	100	-11.617	5.834	9.082	1.00	0.00	C
ATOM	1512	C	ILE	100	-12.622	4.697	8.938	1.00	0.00	C
ATOM	1513	O	ILE	100	-12.378	3.726	8.218	1.00	0.00	O
ATOM	1514	CB	ILE	100	-11.028	6.165	7.698	1.00	0.00	C
ATOM	1515	CG2	ILE	100	-12.141	6.477	6.709	1.00	0.00	C
ATOM	1516	CG1	ILE	100	-10.053	7.337	7.801	1.00	0.00	C
ATOM	1517	CD1	ILE	100	-9.156	7.488	6.590	1.00	0.00	C
ATOM	1518	H	ILE	100	-9.819	4.942	9.749	1.00	0.00	H
ATOM	1519	HA	ILE	100	-12.133	6.712	9.449	1.00	0.00	H
ATOM	1520	HB	ILE	100	-10.497	5.294	7.343	1.00	0.00	H

ATOM	1521	1HG2	ILE	100	-11.718	6.935	5.825	1.00	0.00	H
ATOM	1522	2HG2	ILE	100	-12.646	5.564	6.434	1.00	0.00	H
ATOM	1523	3HG2	ILE	100	-12.846	7.156	7.164	1.00	0.00	H
ATOM	1524	2HG3	ILE	100	-9.421	7.197	8.666	1.00	0.00	H
ATOM	1525	1HD1	ILE	100	-9.003	8.538	6.385	1.00	0.00	H
ATOM	1526	2HD1	ILE	100	-8.206	7.014	6.785	1.00	0.00	H
ATOM	1527	3HD1	ILE	100	-9.625	7.019	5.738	1.00	0.00	H
ATOM	1528	HG2	ILE	100	-10.612	8.267	7.897	1.00	0.00	H
ATOM	1529	N	SER	101	-13.753	4.821	9.624	1.00	0.00	N
ATOM	1530	CA	SER	101	-14.795	3.803	9.574	1.00	0.00	C
ATOM	1531	C	SER	101	-15.385	3.697	8.170	1.00	0.00	C
ATOM	1532	O	SER	101	-15.146	4.555	7.319	1.00	0.00	O
ATOM	1533	CB	SER	101	-15.900	4.123	10.581	1.00	0.00	C
ATOM	1534	OG	SER	101	-16.464	5.399	10.331	1.00	0.00	O
ATOM	1535	H	SER	101	-13.890	5.618	10.179	1.00	0.00	H
ATOM	1536	HA	SER	101	-14.346	2.856	9.833	1.00	0.00	H
ATOM	1537	2HB3	SER	101	-15.488	4.114	11.580	1.00	0.00	H
ATOM	1538	HG	SER	101	-15.810	6.078	10.507	1.00	0.00	H
ATOM	1539	HB2	SER	101	-16.689	3.375	10.505	1.00	0.00	H
ATOM	1540	N	ALA	102	-16.155	2.639	7.937	1.00	0.00	N
ATOM	1541	CA	ALA	102	-16.780	2.422	6.638	1.00	0.00	C
ATOM	1542	C	ALA	102	-17.735	3.559	6.293	1.00	0.00	C
ATOM	1543	O	ALA	102	-17.755	4.044	5.162	1.00	0.00	O
ATOM	1544	CB	ALA	102	-17.516	1.090	6.622	1.00	0.00	C
ATOM	1545	H	ALA	102	-16.307	1.991	8.655	1.00	0.00	H
ATOM	1546	HA	ALA	102	-15.998	2.384	5.894	1.00	0.00	H
ATOM	1547	1HB	ALA	102	-16.833	0.307	6.321	1.00	0.00	H
ATOM	1548	2HB	ALA	102	-17.894	0.877	7.612	1.00	0.00	H
ATOM	1549	3HB	ALA	102	-18.336	1.141	5.925	1.00	0.00	H
ATOM	1550	N	ALA	103	-18.528	3.981	7.272	1.00	0.00	N
ATOM	1551	CA	ALA	103	-19.485	5.062	7.072	1.00	0.00	C
ATOM	1552	C	ALA	103	-18.773	6.372	6.750	1.00	0.00	C
ATOM	1553	O	ALA	103	-19.124	7.059	5.792	1.00	0.00	O
ATOM	1554	CB	ALA	103	-20.364	5.224	8.304	1.00	0.00	C
ATOM	1555	H	ALA	103	-18.465	3.554	8.154	1.00	0.00	H
ATOM	1556	HA	ALA	103	-20.119	4.794	6.240	1.00	0.00	H
ATOM	1557	1HB	ALA	103	-21.012	6.078	8.170	1.00	0.00	H
ATOM	1558	2HB	ALA	103	-20.959	4.335	8.440	1.00	0.00	H
ATOM	1559	3HB	ALA	103	-19.740	5.378	9.171	1.00	0.00	H
ATOM	1560	N	GLU	104	-17.771	6.711	7.558	1.00	0.00	N
ATOM	1561	CA	GLU	104	-17.014	7.939	7.358	1.00	0.00	C
ATOM	1562	C	GLU	104	-16.501	8.036	5.925	1.00	0.00	C
ATOM	1563	O	GLU	104	-16.770	9.011	5.220	1.00	0.00	O
ATOM	1564	CB	GLU	104	-15.838	8.004	8.336	1.00	0.00	C
ATOM	1565	CG	GLU	104	-16.223	8.510	9.716	1.00	0.00	C
ATOM	1566	CD	GLU	104	-15.020	8.732	10.612	1.00	0.00	C
ATOM	1567	OE1	GLU	104	-14.063	9.400	10.164	1.00	0.00	O
ATOM	1568	OE2	GLU	104	-15.034	8.240	11.758	1.00	0.00	O
ATOM	1569	H	GLU	104	-17.540	6.121	8.303	1.00	0.00	H
ATOM	1570	HA	GLU	104	-17.673	8.772	7.548	1.00	0.00	H
ATOM	1571	2HB3	GLU	104	-15.084	8.663	7.931	1.00	0.00	H
ATOM	1572	2HG3	GLU	104	-16.872	7.784	10.184	1.00	0.00	H
ATOM	1573	HB2	GLU	104	-15.428	7.005	8.477	1.00	0.00	H
ATOM	1574	HG2	GLU	104	-16.733	9.469	9.622	1.00	0.00	H
ATOM	1575	N	LEU	105	-15.761	7.019	5.496	1.00	0.00	N
ATOM	1576	CA	LEU	105	-15.210	6.987	4.147	1.00	0.00	C
ATOM	1577	C	LEU	105	-16.321	6.888	3.106	1.00	0.00	C
ATOM	1578	O	LEU	105	-16.315	7.605	2.106	1.00	0.00	O
ATOM	1579	CB	LEU	105	-14.247	5.809	3.994	1.00	0.00	C
ATOM	1580	CG	LEU	105	-13.834	5.461	2.564	1.00	0.00	C
ATOM	1581	CD1	LEU	105	-12.440	4.851	2.546	1.00	0.00	C
ATOM	1582	CD2	LEU	105	-14.841	4.510	1.934	1.00	0.00	C
ATOM	1583	H	LEU	105	-15.580	6.270	6.103	1.00	0.00	H
ATOM	1584	HA	LEU	105	-14.668	7.908	3.988	1.00	0.00	H
ATOM	1585	2HB3	LEU	105	-14.721	4.937	4.424	1.00	0.00	H
ATOM	1586	HG	LEU	105	-13.808	6.366	1.973	1.00	0.00	H
ATOM	1587	1HD1	LEU	105	-11.763	5.515	2.028	1.00	0.00	H
ATOM	1588	2HD1	LEU	105	-12.471	3.899	2.035	1.00	0.00	H
ATOM	1589	3HD1	LEU	105	-12.096	4.706	3.559	1.00	0.00	H
ATOM	1590	1HD2	LEU	105	-14.368	3.968	1.129	1.00	0.00	H
ATOM	1591	2HD2	LEU	105	-15.674	5.078	1.544	1.00	0.00	H
ATOM	1592	3HD2	LEU	105	-15.196	3.816	2.680	1.00	0.00	H
ATOM	1593	HB2	LEU	105	-13.309	6.039	4.499	1.00	0.00	H
ATOM	1594	N	ARG	106	-17.277	5.997	3.351	1.00	0.00	N
ATOM	1595	CA	ARG	106	-18.395	5.804	2.438	1.00	0.00	C
ATOM	1596	C	ARG	106	-18.931	7.146	1.944	1.00	0.00	C
ATOM	1597	O	ARG	106	-19.072	7.364	0.740	1.00	0.00	O

ATOM	1598	CB	ARG	106	-19.513	5.018	3.125	1.00	0.00	C
ATOM	1599	CG	ARG	106	-20.723	4.775	2.235	1.00	0.00	C
ATOM	1600	CD	ARG	106	-21.956	4.429	3.054	1.00	0.00	C
ATOM	1601	NE	ARG	106	-23.171	4.447	2.246	1.00	0.00	N
ATOM	1602	CZ	ARG	106	-24.275	3.779	2.563	1.00	0.00	C
ATOM	1603	NH1	ARG	106	-24.315	3.044	3.665	1.00	0.00	N
ATOM	1604	NH2	ARG	106	-25.342	3.849	1.778	1.00	0.00	N
ATOM	1605	H	ARG	106	-17.227	5.455	4.168	1.00	0.00	H
ATOM	1606	HA	ARG	106	-18.037	5.240	1.589	1.00	0.00	H
ATOM	1607	2HB3	ARG	106	-19.838	5.566	3.996	1.00	0.00	H
ATOM	1608	2HG3	ARG	106	-20.504	3.957	1.564	1.00	0.00	H
ATOM	1609	2HD3	ARG	106	-22.053	5.146	3.854	1.00	0.00	H
ATOM	1610	HE	ARG	106	-23.165	4.986	1.428	1.00	0.00	H
ATOM	1611	1HH1	ARG	106	-23.512	2.992	4.259	1.00	0.00	H
ATOM	1612	2HH1	ARG	106	-25.147	2.543	3.903	1.00	0.00	H
ATOM	1613	1HH2	ARG	106	-25.316	4.403	0.946	1.00	0.00	H
ATOM	1614	2HH2	ARG	106	-26.171	3.346	2.018	1.00	0.00	H
ATOM	1615	HB2	ARG	106	-19.142	4.034	3.412	1.00	0.00	H
ATOM	1616	HG2	ARG	106	-20.942	5.677	1.665	1.00	0.00	H
ATOM	1617	HD2	ARG	106	-21.851	3.423	3.460	1.00	0.00	H
ATOM	1618	N	HIS	107	-19.228	8.039	2.881	1.00	0.00	N
ATOM	1619	CA	HIS	107	-19.748	9.359	2.542	1.00	0.00	C
ATOM	1620	C	HIS	107	-18.791	10.097	1.612	1.00	0.00	C
ATOM	1621	O	HIS	107	-19.196	10.610	0.568	1.00	0.00	O
ATOM	1622	CB	HIS	107	-19.980	10.180	3.810	1.00	0.00	C
ATOM	1623	CG	HIS	107	-19.949	11.660	3.580	1.00	0.00	C
ATOM	1624	ND1	HIS	107	-20.757	12.296	2.661	1.00	0.00	N
ATOM	1625	CD2	HIS	107	-19.203	12.632	4.158	1.00	0.00	C
ATOM	1626	CE1	HIS	107	-20.508	13.593	2.681	1.00	0.00	C
ATOM	1627	NE2	HIS	107	-19.569	13.822	3.581	1.00	0.00	N
ATOM	1628	H	HIS	107	-19.093	7.807	3.824	1.00	0.00	H
ATOM	1629	HA	HIS	107	-20.690	9.223	2.034	1.00	0.00	H
ATOM	1630	2HB3	HIS	107	-19.212	9.941	4.533	1.00	0.00	H
ATOM	1631	1HD	HIS	107	-21.414	11.861	2.080	1.00	0.00	H
ATOM	1632	2HD	HIS	107	-18.458	12.494	4.927	1.00	0.00	H
ATOM	1633	1HE	HIS	107	-20.989	14.339	2.067	1.00	0.00	H
ATOM	1634	2HE	HIS	107	-19.136	14.688	3.738	1.00	0.00	H
ATOM	1635	HB2	HIS	107	-20.963	9.949	4.219	1.00	0.00	H
ATOM	1636	N	VAL	108	-17.520	10.149	1.998	1.00	0.00	N
ATOM	1637	CA	VAL	108	-16.505	10.825	1.197	1.00	0.00	C
ATOM	1638	C	VAL	108	-16.557	10.368	-0.257	1.00	0.00	C
ATOM	1639	O	VAL	108	-16.688	11.183	-1.170	1.00	0.00	O
ATOM	1640	CB	VAL	108	-15.091	10.569	1.752	1.00	0.00	C
ATOM	1641	CG1	VAL	108	-14.043	11.233	0.872	1.00	0.00	C
ATOM	1642	CG2	VAL	108	-14.986	11.067	3.187	1.00	0.00	C
ATOM	1643	H	VAL	108	-17.258	9.722	2.839	1.00	0.00	H
ATOM	1644	HA	VAL	108	-16.700	11.887	1.240	1.00	0.00	H
ATOM	1645	HB	VAL	108	-14.911	9.506	1.750	1.00	0.00	H
ATOM	1646	1HG1	VAL	108	-13.060	11.033	1.271	1.00	0.00	H
ATOM	1647	2HG1	VAL	108	-14.114	10.837	-0.131	1.00	0.00	H
ATOM	1648	3HG1	VAL	108	-14.212	12.299	0.852	1.00	0.00	H
ATOM	1649	1HG2	VAL	108	-14.568	10.289	3.808	1.00	0.00	H
ATOM	1650	2HG2	VAL	108	-14.344	11.937	3.221	1.00	0.00	H
ATOM	1651	3HG2	VAL	108	-15.967	11.331	3.550	1.00	0.00	H
ATOM	1652	N	MET	109	-16.454	9.060	-0.465	1.00	0.00	N
ATOM	1653	CA	MET	109	-16.491	8.494	-1.808	1.00	0.00	C
ATOM	1654	C	MET	109	-17.642	9.088	-2.614	1.00	0.00	C
ATOM	1655	O	MET	109	-17.469	9.463	-3.774	1.00	0.00	O
ATOM	1656	CB	MET	109	-16.632	6.973	-1.739	1.00	0.00	C
ATOM	1657	CG	MET	109	-15.407	6.274	-1.177	1.00	0.00	C
ATOM	1658	SD	MET	109	-14.208	5.836	-2.453	1.00	0.00	S
ATOM	1659	CE	MET	109	-12.981	7.120	-2.219	1.00	0.00	C
ATOM	1660	H	MET	109	-16.352	8.458	0.304	1.00	0.00	H
ATOM	1661	HA	MET	109	-15.561	8.738	-2.296	1.00	0.00	H
ATOM	1662	2HB3	MET	109	-16.810	6.595	-2.735	1.00	0.00	H
ATOM	1663	2HG3	MET	109	-15.722	5.370	-0.675	1.00	0.00	H
ATOM	1664	1HE	MET	109	-12.281	6.815	-1.457	1.00	0.00	H
ATOM	1665	2HE	MET	109	-12.455	7.288	-3.147	1.00	0.00	H
ATOM	1666	3HE	MET	109	-13.470	8.033	-1.911	1.00	0.00	H
ATOM	1667	HB2	MET	109	-17.464	6.713	-1.082	1.00	0.00	H
ATOM	1668	HG2	MET	109	-14.898	6.936	-0.477	1.00	0.00	H
ATOM	1669	N	THR	110	-18.815	9.168	-1.995	1.00	0.00	N
ATOM	1670	CA	THR	110	-19.993	9.714	-2.657	1.00	0.00	C
ATOM	1671	C	THR	110	-19.689	11.062	-3.297	1.00	0.00	C
ATOM	1672	O	THR	110	-19.880	11.249	-4.499	1.00	0.00	O
ATOM	1673	CB	THR	110	-21.165	9.879	-1.670	1.00	0.00	C
ATOM	1674	OG1	THR	110	-21.437	8.633	-1.019	1.00	0.00	O

ATOM	1675	CG2	THR	110	-22.415	10.365	-2.390	1.00	0.00	C
ATOM	1676	H	THR	110	-18.888	8.851	-1.070	1.00	0.00	H
ATOM	1677	HA	THR	110	-20.295	9.020	-3.429	1.00	0.00	H
ATOM	1678	HB	THR	110	-20.889	10.613	-0.927	1.00	0.00	H
ATOM	1679	1HG	THR	110	-20.723	8.426	-0.413	1.00	0.00	H
ATOM	1680	1HG2	THR	110	-22.262	11.379	-2.729	1.00	0.00	H
ATOM	1681	2HG2	THR	110	-23.253	10.333	-1.715	1.00	0.00	H
ATOM	1682	3HG2	THR	110	-22.609	9.727	-3.241	1.00	0.00	H
ATOM	1683	N	ASN	111	-19.213	12.003	-2.487	1.00	0.00	N
ATOM	1684	CA	ASN	111	-18.881	13.337	-2.976	1.00	0.00	C
ATOM	1685	C	ASN	111	-18.268	13.268	-4.370	1.00	0.00	C
ATOM	1686	O	ASN	111	-18.684	13.988	-5.280	1.00	0.00	O
ATOM	1687	CB	ASN	111	-17.912	14.028	-2.014	1.00	0.00	C
ATOM	1688	CG	ASN	111	-18.604	14.546	-0.769	1.00	0.00	C
ATOM	1689	OD1	ASN	111	-19.624	15.231	-0.851	1.00	0.00	O
ATOM	1690	ND2	ASN	111	-18.051	14.220	0.393	1.00	0.00	N
ATOM	1691	H	ASN	111	-19.082	11.797	-1.539	1.00	0.00	H
ATOM	1692	HA	ASN	111	-19.796	13.910	-3.024	1.00	0.00	H
ATOM	1693	2HB3	ASN	111	-17.446	14.862	-2.518	1.00	0.00	H
ATOM	1694	1HD2	ASN	111	-17.239	13.673	0.383	1.00	0.00	H
ATOM	1695	2HD2	ASN	111	-18.477	14.543	1.215	1.00	0.00	H
ATOM	1696	HB2	ASN	111	-17.150	13.318	-1.691	1.00	0.00	H
ATOM	1697	N	LEU	112	-17.278	12.397	-4.534	1.00	0.00	N
ATOM	1698	CA	LEU	112	-16.607	12.233	-5.820	1.00	0.00	C
ATOM	1699	C	LEU	112	-17.605	12.331	-6.970	1.00	0.00	C
ATOM	1700	O	LEU	112	-17.349	12.999	-7.972	1.00	0.00	O
ATOM	1701	CB	LEU	112	-15.883	10.888	-5.871	1.00	0.00	C
ATOM	1702	CG	LEU	112	-14.748	10.697	-4.864	1.00	0.00	C
ATOM	1703	CD1	LEU	112	-14.191	9.284	-4.948	1.00	0.00	C
ATOM	1704	CD2	LEU	112	-13.647	11.720	-5.104	1.00	0.00	C
ATOM	1705	H	LEU	112	-16.991	11.851	-3.774	1.00	0.00	H
ATOM	1706	HA	LEU	112	-15.883	13.027	-5.921	1.00	0.00	H
ATOM	1707	2HB3	LEU	112	-15.471	10.772	-6.864	1.00	0.00	H
ATOM	1708	HG	LEU	112	-15.132	10.846	-3.865	1.00	0.00	H
ATOM	1709	1HD1	LEU	112	-13.380	9.175	-4.243	1.00	0.00	H
ATOM	1710	2HD1	LEU	112	-13.827	9.100	-5.948	1.00	0.00	H
ATOM	1711	3HD1	LEU	112	-14.973	8.576	-4.713	1.00	0.00	H
ATOM	1712	1HD2	LEU	112	-12.685	11.238	-5.028	1.00	0.00	H
ATOM	1713	2HD2	LEU	112	-13.716	12.505	-4.362	1.00	0.00	H
ATOM	1714	3HD2	LEU	112	-13.760	12.148	-6.090	1.00	0.00	H
ATOM	1715	HB2	LEU	112	-16.586	10.087	-5.641	1.00	0.00	H
ATOM	1716	N	GLY	113	-18.743	11.661	-6.820	1.00	0.00	N
ATOM	1717	CA	GLY	113	-19.764	11.687	-7.852	1.00	0.00	C
ATOM	1718	C	GLY	113	-20.317	10.310	-8.155	1.00	0.00	C
ATOM	1719	O	GLY	113	-20.529	9.961	-9.315	1.00	0.00	O
ATOM	1720	H	GLY	113	-18.891	11.145	-6.000	1.00	0.00	H
ATOM	1721	2HA	GLY	113	-20.571	12.326	-7.528	1.00	0.00	H
ATOM	1722	3HA	GLY	113	-19.333	12.096	-8.756	1.00	0.00	H
ATOM	1723	N	GLU	114	-20.551	9.524	-7.109	1.00	0.00	N
ATOM	1724	CA	GLU	114	-21.080	8.176	-7.268	1.00	0.00	C
ATOM	1725	C	GLU	114	-21.887	7.759	-6.043	1.00	0.00	C
ATOM	1726	O	GLU	114	-21.802	8.384	-4.986	1.00	0.00	O
ATOM	1727	CB	GLU	114	-19.945	7.181	-7.506	1.00	0.00	C
ATOM	1728	CG	GLU	114	-19.185	7.420	-8.802	1.00	0.00	C
ATOM	1729	CD	GLU	114	-20.000	7.059	-10.030	1.00	0.00	C
ATOM	1730	OE1	GLU	114	-20.952	6.262	-9.895	1.00	0.00	O
ATOM	1731	OE2	GLU	114	-19.686	7.573	-11.123	1.00	0.00	O
ATOM	1732	H	GLU	114	-20.361	9.859	-6.207	1.00	0.00	H
ATOM	1733	HA	GLU	114	-21.734	8.176	-8.130	1.00	0.00	H
ATOM	1734	2HB3	GLU	114	-20.354	6.183	-7.534	1.00	0.00	H
ATOM	1735	2HG3	GLU	114	-18.288	6.822	-8.795	1.00	0.00	H
ATOM	1736	HB2	GLU	114	-19.210	7.267	-6.705	1.00	0.00	H
ATOM	1737	HG2	GLU	114	-18.934	8.478	-8.886	1.00	0.00	H
ATOM	1738	N	LYS	115	-22.674	6.698	-6.191	1.00	0.00	N
ATOM	1739	CA	LYS	115	-23.496	6.194	-5.098	1.00	0.00	C
ATOM	1740	C	LYS	115	-23.103	4.764	-4.734	1.00	0.00	C
ATOM	1741	O	LYS	115	-23.483	3.813	-5.416	1.00	0.00	O
ATOM	1742	CB	LYS	115	-24.976	6.244	-5.481	1.00	0.00	C
ATOM	1743	CG	LYS	115	-25.536	7.654	-5.559	1.00	0.00	C
ATOM	1744	CD	LYS	115	-26.741	7.725	-6.482	1.00	0.00	C
ATOM	1745	CE	LYS	115	-27.443	9.071	-6.379	1.00	0.00	C
ATOM	1746	NZ	LYS	115	-26.867	10.070	-7.321	1.00	0.00	N
ATOM	1747	H	LYS	115	-22.700	6.240	-7.059	1.00	0.00	H
ATOM	1748	HA	LYS	115	-23.333	6.828	-4.241	1.00	0.00	H
ATOM	1749	2HB3	LYS	115	-25.546	5.694	-4.746	1.00	0.00	H
ATOM	1750	2HG3	LYS	115	-24.768	8.317	-5.933	1.00	0.00	H
ATOM	1751	2HD3	LYS	115	-27.437	6.944	-6.212	1.00	0.00	H

ATOM	1752	2HE3	LYS	115	-27.341	9.440	-5.369	1.00	0.00	H
ATOM	1753	1HZ	LYS	115	-27.418	10.090	-8.204	1.00	0.00	H
ATOM	1754	2HZ	LYS	115	-25.882	9.823	-7.546	1.00	0.00	H
ATOM	1755	3HZ	LYS	115	-26.887	11.018	-6.892	1.00	0.00	H
ATOM	1756	HB2	LYS	115	-25.112	5.807	-6.470	1.00	0.00	H
ATOM	1757	HG2	LYS	115	-25.856	7.975	-4.568	1.00	0.00	H
ATOM	1758	HD2	LYS	115	-26.418	7.595	-7.514	1.00	0.00	H
ATOM	1759	HE2	LYS	115	-28.496	8.952	-6.632	1.00	0.00	H
ATOM	1760	N	LEU	116	-22.341	4.622	-3.655	1.00	0.00	N
ATOM	1761	CA	LEU	116	-21.900	3.309	-3.199	1.00	0.00	C
ATOM	1762	C	LEU	116	-22.713	2.848	-1.996	1.00	0.00	C
ATOM	1763	O	LEU	116	-23.336	3.655	-1.305	1.00	0.00	O
ATOM	1764	CB	LEU	116	-20.414	3.347	-2.838	1.00	0.00	C
ATOM	1765	CG	LEU	116	-19.476	3.902	-3.912	1.00	0.00	C
ATOM	1766	CD1	LEU	116	-18.158	4.345	-3.294	1.00	0.00	C
ATOM	1767	CD2	LEU	116	-19.236	2.866	-5.001	1.00	0.00	C
ATOM	1768	H	LEU	116	-22.071	5.419	-3.150	1.00	0.00	H
ATOM	1769	HA	LEU	116	-22.048	2.610	-4.009	1.00	0.00	H
ATOM	1770	2HB3	LEU	116	-20.102	2.336	-2.617	1.00	0.00	H
ATOM	1771	HG	LEU	116	-19.936	4.768	-4.369	1.00	0.00	H
ATOM	1772	1HD1	LEU	116	-18.323	5.221	-2.686	1.00	0.00	H
ATOM	1773	2HD1	LEU	116	-17.454	4.578	-4.078	1.00	0.00	H
ATOM	1774	3HD1	LEU	116	-17.764	3.546	-2.681	1.00	0.00	H
ATOM	1775	1HD2	LEU	116	-18.944	1.931	-4.546	1.00	0.00	H
ATOM	1776	2HD2	LEU	116	-18.451	3.209	-5.657	1.00	0.00	H
ATOM	1777	3HD2	LEU	116	-20.145	2.723	-5.568	1.00	0.00	H
ATOM	1778	HB2	LEU	116	-20.264	4.003	-1.979	1.00	0.00	H
ATOM	1779	N	THR	117	-22.706	1.541	-1.748	1.00	0.00	N
ATOM	1780	CA	THR	117	-23.442	0.971	-0.627	1.00	0.00	C
ATOM	1781	C	THR	117	-22.516	0.192	0.301	1.00	0.00	C
ATOM	1782	O	THR	117	-21.411	-0.187	-0.084	1.00	0.00	O
ATOM	1783	CB	THR	117	-24.568	0.039	-1.111	1.00	0.00	C
ATOM	1784	OG1	THR	117	-24.036	-0.953	-1.996	1.00	0.00	O
ATOM	1785	CG2	THR	117	-25.657	0.829	-1.821	1.00	0.00	C
ATOM	1786	H	THR	117	-22.191	0.948	-2.334	1.00	0.00	H
ATOM	1787	HA	THR	117	-23.889	1.784	-0.074	1.00	0.00	H
ATOM	1788	HB	THR	117	-25.003	-0.452	-0.251	1.00	0.00	H
ATOM	1789	1HG	THR	117	-24.671	-1.667	-2.096	1.00	0.00	H
ATOM	1790	1HG2	THR	117	-25.694	1.830	-1.420	1.00	0.00	H
ATOM	1791	2HG2	THR	117	-26.610	0.344	-1.669	1.00	0.00	H
ATOM	1792	3HG2	THR	117	-25.440	0.870	-2.877	1.00	0.00	H
ATOM	1793	N	ASP	118	-22.975	-0.043	1.526	1.00	0.00	N
ATOM	1794	CA	ASP	118	-22.189	-0.779	2.509	1.00	0.00	C
ATOM	1795	C	ASP	118	-21.363	-1.872	1.837	1.00	0.00	C
ATOM	1796	O	ASP	118	-20.192	-2.066	2.158	1.00	0.00	O
ATOM	1797	CB	ASP	118	-23.102	-1.392	3.571	1.00	0.00	C
ATOM	1798	CG	ASP	118	-23.366	-0.447	4.724	1.00	0.00	C
ATOM	1799	OD1	ASP	118	-23.257	0.783	4.522	1.00	0.00	O
ATOM	1800	OD2	ASP	118	-23.683	-0.933	5.829	1.00	0.00	O
ATOM	1801	H	ASP	118	-23.865	0.285	1.775	1.00	0.00	H
ATOM	1802	HA	ASP	118	-21.518	-0.081	2.985	1.00	0.00	H
ATOM	1803	2HB3	ASP	118	-22.642	-2.287	3.959	1.00	0.00	H
ATOM	1804	HB2	ASP	118	-24.068	-1.631	3.124	1.00	0.00	H
ATOM	1805	N	GLU	119	-21.985	-2.586	0.903	1.00	0.00	N
ATOM	1806	CA	GLU	119	-21.308	-3.660	0.184	1.00	0.00	C
ATOM	1807	C	GLU	119	-20.074	-3.136	-0.540	1.00	0.00	C
ATOM	1808	O	GLU	119	-18.960	-3.605	-0.307	1.00	0.00	O
ATOM	1809	CB	GLU	119	-22.263	-4.317	-0.815	1.00	0.00	C
ATOM	1810	CG	GLU	119	-23.413	-5.060	-0.160	1.00	0.00	C
ATOM	1811	CD	GLU	119	-22.950	-6.251	0.657	1.00	0.00	C
ATOM	1812	OE1	GLU	119	-22.144	-7.050	0.135	1.00	0.00	O
ATOM	1813	OE2	GLU	119	-23.391	-6.383	1.818	1.00	0.00	O
ATOM	1814	H	GLU	119	-22.920	-2.385	0.690	1.00	0.00	H
ATOM	1815	HA	GLU	119	-20.998	-4.397	0.912	1.00	0.00	H
ATOM	1816	2HB3	GLU	119	-21.705	-5.018	-1.417	1.00	0.00	H
ATOM	1817	2HG3	GLU	119	-24.083	-5.411	-0.930	1.00	0.00	H
ATOM	1818	HB2	GLU	119	-22.713	-3.550	-1.444	1.00	0.00	H
ATOM	1819	HG2	GLU	119	-23.938	-4.391	0.521	1.00	0.00	H
ATOM	1820	N	GLU	120	-20.279	-2.164	-1.422	1.00	0.00	N
ATOM	1821	CA	GLU	120	-19.183	-1.577	-2.183	1.00	0.00	C
ATOM	1822	C	GLU	120	-18.034	-1.176	-1.263	1.00	0.00	C
ATOM	1823	O	GLU	120	-16.886	-1.569	-1.477	1.00	0.00	O
ATOM	1824	CB	GLU	120	-19.671	-0.358	-2.966	1.00	0.00	C
ATOM	1825	CG	GLU	120	-20.751	-0.682	-3.986	1.00	0.00	C
ATOM	1826	CD	GLU	120	-20.465	-1.956	-4.756	1.00	0.00	C
ATOM	1827	OE1	GLU	120	-20.770	-3.047	-4.228	1.00	0.00	O
ATOM	1828	OE2	GLU	120	-19.934	-1.864	-5.881	1.00	0.00	O

ATOM	1829	H	GLU	120	-21.192	-1.833	-1.563	1.00	0.00	H
ATOM	1830	HA	GLU	120	-18.828	-2.322	-2.879	1.00	0.00	H
ATOM	1831	2HB3	GLU	120	-18.833	0.078	-3.490	1.00	0.00	H
ATOM	1832	2HG3	GLU	120	-20.823	0.137	-4.689	1.00	0.00	H
ATOM	1833	HB2	GLU	120	-20.108	0.365	-2.279	1.00	0.00	H
ATOM	1834	HG2	GLU	120	-21.705	-0.821	-3.475	1.00	0.00	H
ATOM	1835	N	VAL	121	-18.352	-0.393	-0.236	1.00	0.00	N
ATOM	1836	CA	VAL	121	-17.347	0.060	0.718	1.00	0.00	C
ATOM	1837	C	VAL	121	-16.676	-1.120	1.412	1.00	0.00	C
ATOM	1838	O	VAL	121	-15.458	-1.139	1.589	1.00	0.00	O
ATOM	1839	CB	VAL	121	-17.962	0.986	1.783	1.00	0.00	C
ATOM	1840	CG1	VAL	121	-16.926	1.355	2.834	1.00	0.00	C
ATOM	1841	CG2	VAL	121	-18.543	2.231	1.134	1.00	0.00	C
ATOM	1842	H	VAL	121	-19.283	-0.115	-0.117	1.00	0.00	H
ATOM	1843	HA	VAL	121	-16.599	0.618	0.175	1.00	0.00	H
ATOM	1844	HB	VAL	121	-18.765	0.453	2.273	1.00	0.00	H
ATOM	1845	1HG1	VAL	121	-16.954	2.421	3.006	1.00	0.00	H
ATOM	1846	2HG1	VAL	121	-17.144	0.834	3.755	1.00	0.00	H
ATOM	1847	3HG1	VAL	121	-15.943	1.073	2.484	1.00	0.00	H
ATOM	1848	1HG2	VAL	121	-17.982	2.468	0.242	1.00	0.00	H
ATOM	1849	2HG2	VAL	121	-19.577	2.054	0.872	1.00	0.00	H
ATOM	1850	3HG2	VAL	121	-18.485	3.060	1.825	1.00	0.00	H
ATOM	1851	N	ASP	122	-17.479	-2.103	1.803	1.00	0.00	N
ATOM	1852	CA	ASP	122	-16.963	-3.290	2.477	1.00	0.00	C
ATOM	1853	C	ASP	122	-15.774	-3.872	1.720	1.00	0.00	C
ATOM	1854	O	ASP	122	-14.746	-4.197	2.314	1.00	0.00	O
ATOM	1855	CB	ASP	122	-18.063	-4.344	2.613	1.00	0.00	C
ATOM	1856	CG	ASP	122	-18.844	-4.203	3.905	1.00	0.00	C
ATOM	1857	OD1	ASP	122	-18.262	-3.722	4.899	1.00	0.00	O
ATOM	1858	OD2	ASP	122	-20.036	-4.574	3.922	1.00	0.00	O
ATOM	1859	H	ASP	122	-18.442	-2.031	1.634	1.00	0.00	H
ATOM	1860	HA	ASP	122	-16.637	-2.994	3.463	1.00	0.00	H
ATOM	1861	2HB3	ASP	122	-17.616	-5.326	2.590	1.00	0.00	H
ATOM	1862	HB2	ASP	122	-18.772	-4.238	1.794	1.00	0.00	H
ATOM	1863	N	GLU	123	-15.924	-4.003	0.405	1.00	0.00	N
ATOM	1864	CA	GLU	123	-14.861	-4.547	-0.434	1.00	0.00	C
ATOM	1865	C	GLU	123	-13.599	-3.695	-0.335	1.00	0.00	C
ATOM	1866	O	GLU	123	-12.484	-4.201	-0.469	1.00	0.00	O
ATOM	1867	CB	GLU	123	-15.322	-4.627	-1.890	1.00	0.00	C
ATOM	1868	CG	GLU	123	-16.564	-5.479	-2.089	1.00	0.00	C
ATOM	1869	CD	GLU	123	-16.794	-5.844	-3.543	1.00	0.00	C
ATOM	1870	OE1	GLU	123	-15.799	-6.064	-4.263	1.00	0.00	O
ATOM	1871	OE2	GLU	123	-17.971	-5.910	-3.960	1.00	0.00	O
ATOM	1872	H	GLU	123	-16.766	-3.724	-0.011	1.00	0.00	H
ATOM	1873	HA	GLU	123	-14.638	-5.543	-0.079	1.00	0.00	H
ATOM	1874	2HB3	GLU	123	-14.524	-5.049	-2.484	1.00	0.00	H
ATOM	1875	2HG3	GLU	123	-17.423	-4.930	-1.731	1.00	0.00	H
ATOM	1876	HB2	GLU	123	-15.573	-3.629	-2.248	1.00	0.00	H
ATOM	1877	HG2	GLU	123	-16.456	-6.414	-1.539	1.00	0.00	H
ATOM	1878	N	MET	124	-13.783	-2.401	-0.097	1.00	0.00	N
ATOM	1879	CA	MET	124	-12.657	-1.478	0.020	1.00	0.00	C
ATOM	1880	C	MET	124	-11.961	-1.641	1.368	1.00	0.00	C
ATOM	1881	O	MET	124	-10.737	-1.759	1.433	1.00	0.00	O
ATOM	1882	CB	MET	124	-13.135	-0.035	-0.151	1.00	0.00	C
ATOM	1883	CG	MET	124	-12.073	0.999	0.181	1.00	0.00	C
ATOM	1884	SD	MET	124	-12.316	2.554	-0.699	1.00	0.00	S
ATOM	1885	CE	MET	124	-11.018	3.557	0.021	1.00	0.00	C
ATOM	1886	H	MET	124	-14.695	-2.055	0.000	1.00	0.00	H
ATOM	1887	HA	MET	124	-11.956	-1.712	-0.766	1.00	0.00	H
ATOM	1888	2HB3	MET	124	-13.983	0.131	0.496	1.00	0.00	H
ATOM	1889	2HG3	MET	124	-11.104	0.598	-0.084	1.00	0.00	H
ATOM	1890	1HE	MET	124	-11.429	4.505	0.336	1.00	0.00	H
ATOM	1891	2HE	MET	124	-10.600	3.047	0.875	1.00	0.00	H
ATOM	1892	3HE	MET	124	-10.245	3.724	-0.713	1.00	0.00	H
ATOM	1893	HB2	MET	124	-13.416	0.133	-1.191	1.00	0.00	H
ATOM	1894	HG2	MET	124	-12.108	1.229	1.247	1.00	0.00	H
ATOM	1895	N	ILE	125	-12.748	-1.645	2.438	1.00	0.00	N
ATOM	1896	CA	ILE	125	-12.205	-1.792	3.783	1.00	0.00	C
ATOM	1897	C	ILE	125	-11.299	-3.015	3.879	1.00	0.00	C
ATOM	1898	O	ILE	125	-10.200	-2.944	4.428	1.00	0.00	O
ATOM	1899	CB	ILE	125	-13.326	-1.914	4.832	1.00	0.00	C
ATOM	1900	CG2	ILE	125	-12.743	-2.255	6.194	1.00	0.00	C
ATOM	1901	CG1	ILE	125	-14.134	-0.616	4.900	1.00	0.00	C
ATOM	1902	CD1	ILE	125	-13.355	0.550	5.469	1.00	0.00	C
ATOM	1903	H	ILE	125	-13.716	-1.547	2.321	1.00	0.00	H
ATOM	1904	HA	ILE	125	-11.625	-0.908	4.006	1.00	0.00	H
ATOM	1905	HB	ILE	125	-13.980	-2.721	4.535	1.00	0.00	H

ATOM	1906	1HG2	ILE	125	-12.214	-1.398	6.583	1.00	0.00	H
ATOM	1907	2HG2	ILE	125	-13.539	-2.524	6.871	1.00	0.00	H
ATOM	1908	3HG2	ILE	125	-12.058	-3.086	6.096	1.00	0.00	H
ATOM	1909	2HG3	ILE	125	-15.000	-0.773	5.527	1.00	0.00	H
ATOM	1910	1HD1	ILE	125	-12.308	0.291	5.521	1.00	0.00	H
ATOM	1911	2HD1	ILE	125	-13.484	1.412	4.832	1.00	0.00	H
ATOM	1912	3HD1	ILE	125	-13.718	0.776	6.461	1.00	0.00	H
ATOM	1913	HG2	ILE	125	-14.449	-0.330	3.898	1.00	0.00	H
ATOM	1914	N	ARG	126	-11.768	-4.135	3.337	1.00	0.00	N
ATOM	1915	CA	ARG	126	-10.998	-5.374	3.361	1.00	0.00	C
ATOM	1916	C	ARG	126	-9.695	-5.220	2.584	1.00	0.00	C
ATOM	1917	O	ARG	126	-8.841	-6.106	2.603	1.00	0.00	O
ATOM	1918	CB	ARG	126	-11.823	-6.522	2.774	1.00	0.00	C
ATOM	1919	CG	ARG	126	-11.947	-6.470	1.261	1.00	0.00	C
ATOM	1920	CD	ARG	126	-12.213	-7.847	0.676	1.00	0.00	C
ATOM	1921	NE	ARG	126	-11.065	-8.737	0.825	1.00	0.00	N
ATOM	1922	CZ	ARG	126	-11.140	-10.059	0.725	1.00	0.00	C
ATOM	1923	NH1	ARG	126	-12.305	-10.643	0.473	1.00	0.00	N
ATOM	1924	NH2	ARG	126	-10.050	-10.801	0.873	1.00	0.00	N
ATOM	1925	H	ARG	126	-12.651	-4.128	2.913	1.00	0.00	H
ATOM	1926	HA	ARG	126	-10.766	-5.600	4.391	1.00	0.00	H
ATOM	1927	2HB3	ARG	126	-12.814	-6.489	3.196	1.00	0.00	H
ATOM	1928	2HG3	ARG	126	-11.028	-6.083	0.846	1.00	0.00	H
ATOM	1929	2HD3	ARG	126	-12.439	-7.740	-0.375	1.00	0.00	H
ATOM	1930	HE	ARG	126	-10.193	-8.326	1.010	1.00	0.00	H
ATOM	1931	1HH1	ARG	126	-13.127	-10.086	0.359	1.00	0.00	H
ATOM	1932	2HH1	ARG	126	-12.360	-11.639	0.397	1.00	0.00	H
ATOM	1933	1HH2	ARG	126	-9.171	-10.365	1.062	1.00	0.00	H
ATOM	1934	2HH2	ARG	126	-10.109	-11.797	0.796	1.00	0.00	H
ATOM	1935	HB2	ARG	126	-11.342	-7.471	3.007	1.00	0.00	H
ATOM	1936	HG2	ARG	126	-12.780	-5.821	0.986	1.00	0.00	H
ATOM	1937	HD2	ARG	126	-13.052	-8.310	1.198	1.00	0.00	H
ATOM	1938	N	GLU	127	-9.549	-4.089	1.901	1.00	0.00	N
ATOM	1939	CA	GLU	127	-8.350	-3.821	1.115	1.00	0.00	C
ATOM	1940	C	GLU	127	-7.268	-3.178	1.980	1.00	0.00	C
ATOM	1941	O	GLU	127	-6.139	-3.665	2.044	1.00	0.00	O
ATOM	1942	CB	GLU	127	-8.681	-2.910	-0.067	1.00	0.00	C
ATOM	1943	CG	GLU	127	-7.685	-3.010	-1.210	1.00	0.00	C
ATOM	1944	CD	GLU	127	-7.642	-1.754	-2.058	1.00	0.00	C
ATOM	1945	OE1	GLU	127	-8.683	-1.404	-2.655	1.00	0.00	O
ATOM	1946	OE2	GLU	127	-6.570	-1.119	-2.127	1.00	0.00	O
ATOM	1947	H	GLU	127	-10.264	-3.420	1.926	1.00	0.00	H
ATOM	1948	HA	GLU	127	-7.982	-4.762	0.741	1.00	0.00	H
ATOM	1949	2HB3	GLU	127	-8.700	-1.886	0.275	1.00	0.00	H
ATOM	1950	2HG3	GLU	127	-7.962	-3.842	-1.841	1.00	0.00	H
ATOM	1951	HB2	GLU	127	-9.650	-3.189	-0.482	1.00	0.00	H
ATOM	1952	HG2	GLU	127	-6.684	-3.159	-0.808	1.00	0.00	H
ATOM	1953	N	ALA	128	-7.619	-2.079	2.638	1.00	0.00	N
ATOM	1954	CA	ALA	128	-6.681	-1.370	3.500	1.00	0.00	C
ATOM	1955	C	ALA	128	-6.681	-1.951	4.909	1.00	0.00	C
ATOM	1956	O	ALA	128	-5.630	-2.094	5.532	1.00	0.00	O
ATOM	1957	CB	ALA	128	-7.017	0.112	3.537	1.00	0.00	C
ATOM	1958	H	ALA	128	-8.534	-1.739	2.548	1.00	0.00	H
ATOM	1959	HA	ALA	128	-5.693	-1.480	3.076	1.00	0.00	H
ATOM	1960	1HB	ALA	128	-7.762	0.332	2.787	1.00	0.00	H
ATOM	1961	2HB	ALA	128	-7.402	0.369	4.513	1.00	0.00	H
ATOM	1962	3HB	ALA	128	-6.125	0.689	3.340	1.00	0.00	H
ATOM	1963	N	ASP	129	-7.867	-2.284	5.407	1.00	0.00	N
ATOM	1964	CA	ASP	129	-8.002	-2.851	6.743	1.00	0.00	C
ATOM	1965	C	ASP	129	-7.145	-4.103	6.894	1.00	0.00	C
ATOM	1966	O	ASP	129	-7.445	-5.147	6.313	1.00	0.00	O
ATOM	1967	CB	ASP	129	-9.469	-3.184	7.031	1.00	0.00	C
ATOM	1968	CG	ASP	129	-9.774	-3.205	8.517	1.00	0.00	C
ATOM	1969	OD1	ASP	129	-8.821	-3.305	9.317	1.00	0.00	O
ATOM	1970	OD2	ASP	129	-10.968	-3.124	8.877	1.00	0.00	O
ATOM	1971	H	ASP	129	-8.668	-2.148	4.862	1.00	0.00	H
ATOM	1972	HA	ASP	129	-7.664	-2.111	7.454	1.00	0.00	H
ATOM	1973	2HB3	ASP	129	-9.697	-4.156	6.621	1.00	0.00	H
ATOM	1974	HB2	ASP	129	-10.110	-2.428	6.579	1.00	0.00	H
ATOM	1975	N	ILE	130	-6.076	-3.991	7.676	1.00	0.00	N
ATOM	1976	CA	ILE	130	-5.176	-5.114	7.902	1.00	0.00	C
ATOM	1977	C	ILE	130	-5.687	-6.013	9.020	1.00	0.00	C
ATOM	1978	O	ILE	130	-5.989	-7.187	8.799	1.00	0.00	O
ATOM	1979	CB	ILE	130	-3.755	-4.635	8.256	1.00	0.00	C
ATOM	1980	CG2	ILE	130	-2.781	-5.804	8.240	1.00	0.00	C
ATOM	1981	CG1	ILE	130	-3.304	-3.546	7.280	1.00	0.00	C
ATOM	1982	CD1	ILE	130	-3.066	-4.053	5.874	1.00	0.00	C

ATOM	1983	H	ILE	130	-5.891	-3.135	8.111	1.00	0.00	H
ATOM	1984	HA	ILE	130	-5.122	-5.688	6.989	1.00	0.00	H
ATOM	1985	HB	ILE	130	-3.775	-4.225	9.255	1.00	0.00	H
ATOM	1986	1HG2	ILE	130	-1.778	-5.437	8.399	1.00	0.00	H
ATOM	1987	2HG2	ILE	130	-3.040	-6.496	9.026	1.00	0.00	H
ATOM	1988	3HG2	ILE	130	-2.835	-6.305	7.285	1.00	0.00	H
ATOM	1989	2HG3	ILE	130	-2.382	-3.112	7.638	1.00	0.00	H
ATOM	1990	1HD1	ILE	130	-2.031	-4.338	5.765	1.00	0.00	H
ATOM	1991	2HD1	ILE	130	-3.698	-4.908	5.688	1.00	0.00	H
ATOM	1992	3HD1	ILE	130	-3.300	-3.272	5.166	1.00	0.00	H
ATOM	1993	HG2	ILE	130	-4.075	-2.777	7.212	1.00	0.00	H
ATOM	1994	N	ASP	131	-5.787	-5.458	10.223	1.00	0.00	N
ATOM	1995	CA	ASP	131	-6.267	-6.208	11.378	1.00	0.00	C
ATOM	1996	C	ASP	131	-7.638	-6.817	11.097	1.00	0.00	C
ATOM	1997	O	ASP	131	-7.880	-7.987	11.388	1.00	0.00	O
ATOM	1998	CB	ASP	131	-6.338	-5.304	12.607	1.00	0.00	C
ATOM	1999	CG	ASP	131	-7.400	-4.230	12.476	1.00	0.00	C
ATOM	2000	OD1	ASP	131	-7.411	-3.530	11.442	1.00	0.00	O
ATOM	2001	OD2	ASP	131	-8.220	-4.089	13.409	1.00	0.00	O
ATOM	2002	H	ASP	131	-5.531	-4.517	10.336	1.00	0.00	H
ATOM	2003	HA	ASP	131	-5.564	-7.005	11.569	1.00	0.00	H
ATOM	2004	2HB3	ASP	131	-5.381	-4.822	12.747	1.00	0.00	H
ATOM	2005	HB2	ASP	131	-6.586	-5.900	13.486	1.00	0.00	H
ATOM	2006	N	GLY	132	-8.532	-6.013	10.532	1.00	0.00	N
ATOM	2007	CA	GLY	132	-9.868	-6.488	10.224	1.00	0.00	C
ATOM	2008	C	GLY	132	-10.895	-6.034	11.242	1.00	0.00	C
ATOM	2009	O	GLY	132	-11.734	-6.820	11.682	1.00	0.00	O
ATOM	2010	H	GLY	132	-8.284	-5.088	10.322	1.00	0.00	H
ATOM	2011	2HA	GLY	132	-10.154	-6.120	9.252	1.00	0.00	H
ATOM	2012	3HA	GLY	132	-9.856	-7.568	10.200	1.00	0.00	H
ATOM	2013	N	ASP	133	-10.828	-4.762	11.622	1.00	0.00	N
ATOM	2014	CA	ASP	133	-11.756	-4.204	12.596	1.00	0.00	C
ATOM	2015	C	ASP	133	-12.770	-3.289	11.916	1.00	0.00	C
ATOM	2016	O	ASP	133	-13.451	-2.504	12.573	1.00	0.00	O
ATOM	2017	CB	ASP	133	-10.997	-3.432	13.675	1.00	0.00	C
ATOM	2018	CG	ASP	133	-10.154	-2.313	13.102	1.00	0.00	C
ATOM	2019	OD1	ASP	133	-10.314	-2.004	11.902	1.00	0.00	O
ATOM	2020	OD2	ASP	133	-9.331	-1.744	13.852	1.00	0.00	O
ATOM	2021	H	ASP	133	-10.134	-4.185	11.235	1.00	0.00	H
ATOM	2022	HA	ASP	133	-12.287	-5.025	13.056	1.00	0.00	H
ATOM	2023	2HB3	ASP	133	-10.348	-4.115	14.205	1.00	0.00	H
ATOM	2024	HB2	ASP	133	-11.708	-2.982	14.368	1.00	0.00	H
ATOM	2025	N	GLY	134	-12.863	-3.393	10.594	1.00	0.00	N
ATOM	2026	CA	GLY	134	-13.792	-2.566	9.848	1.00	0.00	C
ATOM	2027	C	GLY	134	-13.413	-1.099	9.871	1.00	0.00	C
ATOM	2028	O	GLY	134	-14.281	-0.226	9.853	1.00	0.00	O
ATOM	2029	H	GLY	134	-12.294	-4.036	10.121	1.00	0.00	H
ATOM	2030	2HA	GLY	134	-13.816	-2.905	8.822	1.00	0.00	H
ATOM	2031	3HA	GLY	134	-14.778	-2.678	10.275	1.00	0.00	H
ATOM	2032	N	GLN	135	-12.113	-0.826	9.911	1.00	0.00	N
ATOM	2033	CA	GLN	135	-11.621	0.548	9.938	1.00	0.00	C
ATOM	2034	C	GLN	135	-10.379	0.697	9.066	1.00	0.00	C
ATOM	2035	O	GLN	135	-9.741	-0.288	8.700	1.00	0.00	O
ATOM	2036	CB	GLN	135	-11.304	0.967	11.374	1.00	0.00	C
ATOM	2037	CG	GLN	135	-12.397	0.608	12.369	1.00	0.00	C
ATOM	2038	CD	GLN	135	-12.474	1.582	13.528	1.00	0.00	C
ATOM	2039	OE1	GLN	135	-13.058	2.660	13.410	1.00	0.00	O
ATOM	2040	NE2	GLN	135	-11.882	1.210	14.657	1.00	0.00	N
ATOM	2041	H	GLN	135	-11.470	-1.564	9.921	1.00	0.00	H
ATOM	2042	HA	GLN	135	-12.399	1.186	9.550	1.00	0.00	H
ATOM	2043	2HB3	GLN	135	-11.163	2.037	11.400	1.00	0.00	H
ATOM	2044	2HG3	GLN	135	-12.198	-0.379	12.760	1.00	0.00	H
ATOM	2045	1HE2	GLN	135	-11.434	0.337	14.678	1.00	0.00	H
ATOM	2046	2HE2	GLN	135	-11.916	1.819	15.421	1.00	0.00	H
ATOM	2047	HB2	GLN	135	-10.401	0.459	11.711	1.00	0.00	H
ATOM	2048	HG2	GLN	135	-13.364	0.629	11.868	1.00	0.00	H
ATOM	2049	N	VAL	136	-10.043	1.939	8.734	1.00	0.00	N
ATOM	2050	CA	VAL	136	-8.878	2.221	7.905	1.00	0.00	C
ATOM	2051	C	VAL	136	-8.062	3.375	8.476	1.00	0.00	C
ATOM	2052	O	VAL	136	-8.179	4.514	8.023	1.00	0.00	O
ATOM	2053	CB	VAL	136	-9.286	2.561	6.459	1.00	0.00	C
ATOM	2054	CG1	VAL	136	-8.061	2.896	5.623	1.00	0.00	C
ATOM	2055	CG2	VAL	136	-10.066	1.409	5.841	1.00	0.00	C
ATOM	2056	H	VAL	136	-10.591	2.685	9.057	1.00	0.00	H
ATOM	2057	HA	VAL	136	-8.261	1.333	7.884	1.00	0.00	H
ATOM	2058	HB	VAL	136	-9.927	3.430	6.483	1.00	0.00	H
ATOM	2059	1HG1	VAL	136	-7.897	3.964	5.636	1.00	0.00	H

```
ATOM   2060 2HG1 VAL   136      -7.197   2.393   6.031  1.00  0.00           H
ATOM   2061 3HG1 VAL   136      -8.221   2.571   4.606  1.00  0.00           H
ATOM   2062 1HG2 VAL   136      -9.393   0.783   5.275  1.00  0.00           H
ATOM   2063 2HG2 VAL   136     -10.527   0.824   6.625  1.00  0.00           H
ATOM   2064 3HG2 VAL   136     -10.830   1.802   5.188  1.00  0.00           H
ATOM   2065  N   ASN   137      -7.236   3.075   9.472  1.00  0.00           N
ATOM   2066  CA  ASN   137      -6.400   4.088  10.107  1.00  0.00           C
ATOM   2067  C   ASN   137      -5.349   4.611   9.133  1.00  0.00           C
ATOM   2068  O   ASN   137      -5.271   4.167   7.988  1.00  0.00           O
ATOM   2069  CB  ASN   137      -5.721   3.512  11.350  1.00  0.00           C
ATOM   2070  CG  ASN   137      -5.093   2.157  11.090  1.00  0.00           C
ATOM   2071  OD1 ASN   137      -5.758   1.124  11.176  1.00  0.00           O
ATOM   2072  ND2 ASN   137      -3.803   2.154  10.771  1.00  0.00           N
ATOM   2073  H   ASN   137      -7.188   2.148   9.790  1.00  0.00           H
ATOM   2074  HA  ASN   137      -7.041   4.905  10.403  1.00  0.00           H
ATOM   2075 2HB3 ASN   137      -6.452   3.406  12.138  1.00  0.00           H
ATOM   2076 1HD2 ASN   137      -3.337   3.014  10.721  1.00  0.00           H
ATOM   2077 2HD2 ASN   137      -3.372   1.290  10.599  1.00  0.00           H
ATOM   2078  HB2 ASN   137      -4.922   4.182  11.671  1.00  0.00           H
ATOM   2079  N   TYR   138      -4.541   5.557   9.598  1.00  0.00           N
ATOM   2080  CA  TYR   138      -3.495   6.144   8.769  1.00  0.00           C
ATOM   2081  C   TYR   138      -2.619   5.058   8.148  1.00  0.00           C
ATOM   2082  O   TYR   138      -2.490   4.975   6.927  1.00  0.00           O
ATOM   2083  CB  TYR   138      -2.631   7.096   9.597  1.00  0.00           C
ATOM   2084  CG  TYR   138      -1.546   7.779   8.797  1.00  0.00           C
ATOM   2085  CD1 TYR   138      -1.857   8.598   7.719  1.00  0.00           C
ATOM   2086  CE1 TYR   138      -0.866   9.223   6.986  1.00  0.00           C
ATOM   2087  CZ  TYR   138       0.456   9.034   7.328  1.00  0.00           C
ATOM   2088  CE2 TYR   138       0.792   8.226   8.394  1.00  0.00           C
ATOM   2089  CD2 TYR   138      -0.205   7.604   9.120  1.00  0.00           C
ATOM   2090  OH  TYR   138       1.445   9.655   6.601  1.00  0.00           O
ATOM   2091  H   TYR   138      -4.652   5.871  10.519  1.00  0.00           H
ATOM   2092  HA  TYR   138      -3.972   6.703   7.977  1.00  0.00           H
ATOM   2093 2HB3 TYR   138      -2.160   6.543  10.396  1.00  0.00           H
ATOM   2094 1HD  TYR   138      -2.895   8.745   7.457  1.00  0.00           H
ATOM   2095 1HE  TYR   138      -1.130   9.856   6.152  1.00  0.00           H
ATOM   2096 2HE  TYR   138       1.828   8.077   8.660  1.00  0.00           H
ATOM   2097 2HD  TYR   138       0.056   6.970   9.956  1.00  0.00           H
ATOM   2098  HH  TYR   138       1.820   9.031   5.973  1.00  0.00           H
ATOM   2099  HB2 TYR   138      -3.258   7.888  10.010  1.00  0.00           H
ATOM   2100  N   GLU   139      -2.021   4.230   8.999  1.00  0.00           N
ATOM   2101  CA  GLU   139      -1.159   3.150   8.534  1.00  0.00           C
ATOM   2102  C   GLU   139      -1.870   2.301   7.481  1.00  0.00           C
ATOM   2103  O   GLU   139      -1.322   2.036   6.413  1.00  0.00           O
ATOM   2104  CB  GLU   139      -0.730   2.270   9.711  1.00  0.00           C
ATOM   2105  CG  GLU   139       0.166   1.110   9.306  1.00  0.00           C
ATOM   2106  CD  GLU   139       0.004  -0.094  10.214  1.00  0.00           C
ATOM   2107  OE1 GLU   139      -1.096  -0.274  10.773  1.00  0.00           O
ATOM   2108  OE2 GLU   139       0.981  -0.857  10.364  1.00  0.00           O
ATOM   2109  H   GLU   139      -2.165   4.348   9.961  1.00  0.00           H
ATOM   2110  HA  GLU   139      -0.284   3.594   8.091  1.00  0.00           H
ATOM   2111 2HB3 GLU   139      -1.612   1.867  10.184  1.00  0.00           H
ATOM   2112 2HG3 GLU   139       1.194   1.436   9.345  1.00  0.00           H
ATOM   2113  HB2 GLU   139      -0.159   2.867  10.422  1.00  0.00           H
ATOM   2114  HG2 GLU   139      -0.090   0.789   8.296  1.00  0.00           H
ATOM   2115  N   GLU   140      -3.089   1.878   7.797  1.00  0.00           N
ATOM   2116  CA  GLU   140      -3.874   1.059   6.880  1.00  0.00           C
ATOM   2117  C   GLU   140      -4.148   1.809   5.580  1.00  0.00           C
ATOM   2118  O   GLU   140      -4.154   1.219   4.498  1.00  0.00           O
ATOM   2119  CB  GLU   140      -5.193   0.644   7.532  1.00  0.00           C
ATOM   2120  CG  GLU   140      -5.022  -0.297   8.712  1.00  0.00           C
ATOM   2121  CD  GLU   140      -6.340  -0.641   9.380  1.00  0.00           C
ATOM   2122  OE1 GLU   140      -7.206   0.252   9.479  1.00  0.00           O
ATOM   2123  OE2 GLU   140      -6.502  -1.805   9.802  1.00  0.00           O
ATOM   2124  H   GLU   140      -3.472   2.122   8.665  1.00  0.00           H
ATOM   2125  HA  GLU   140      -3.299   0.172   6.656  1.00  0.00           H
ATOM   2126 2HB3 GLU   140      -5.806   0.152   6.791  1.00  0.00           H
ATOM   2127 2HG3 GLU   140      -4.378   0.174   9.440  1.00  0.00           H
ATOM   2128  HB2 GLU   140      -5.702   1.529   7.916  1.00  0.00           H
ATOM   2129  HG2 GLU   140      -4.581  -1.232   8.369  1.00  0.00           H
ATOM   2130  N   PHE   141      -4.374   3.112   5.691  1.00  0.00           N
ATOM   2131  CA  PHE   141      -4.649   3.943   4.525  1.00  0.00           C
ATOM   2132  C   PHE   141      -3.414   4.068   3.641  1.00  0.00           C
ATOM   2133  O   PHE   141      -3.490   3.910   2.423  1.00  0.00           O
ATOM   2134  CB  PHE   141      -5.119   5.332   4.963  1.00  0.00           C
ATOM   2135  CG  PHE   141      -5.008   6.370   3.884  1.00  0.00           C
ATOM   2136  CD1 PHE   141      -5.649   6.196   2.666  1.00  0.00           C
```

ATOM	2137	CE1	PHE	141	-5.545	7.149	1.672	1.00	0.00	C
ATOM	2138	CZ	PHE	141	-4.803	8.293	1.886	1.00	0.00	C
ATOM	2139	CE2	PHE	141	-4.160	8.480	3.094	1.00	0.00	C
ATOM	2140	CD2	PHE	141	-4.265	7.523	4.086	1.00	0.00	C
ATOM	2141	H	PHE	141	-4.355	3.526	6.580	1.00	0.00	H
ATOM	2142	HA	PHE	141	-5.436	3.469	3.961	1.00	0.00	H
ATOM	2143	2HB3	PHE	141	-4.524	5.659	5.802	1.00	0.00	H
ATOM	2144	1HD	PHE	141	-6.232	5.303	2.499	1.00	0.00	H
ATOM	2145	1HE	PHE	141	-6.050	7.002	0.728	1.00	0.00	H
ATOM	2146	HZ	PHE	141	-4.721	9.038	1.109	1.00	0.00	H
ATOM	2147	2HE	PHE	141	-3.577	9.373	3.265	1.00	0.00	H
ATOM	2148	2HD	PHE	141	-3.761	7.669	5.032	1.00	0.00	H
ATOM	2149	HB2	PHE	141	-6.171	5.289	5.247	1.00	0.00	H
ATOM	2150	N	VAL	142	-2.274	4.353	4.263	1.00	0.00	N
ATOM	2151	CA	VAL	142	-1.020	4.500	3.533	1.00	0.00	C
ATOM	2152	C	VAL	142	-0.742	3.276	2.669	1.00	0.00	C
ATOM	2153	O	VAL	142	-0.452	3.398	1.478	1.00	0.00	O
ATOM	2154	CB	VAL	142	0.166	4.718	4.493	1.00	0.00	C
ATOM	2155	CG1	VAL	142	1.481	4.687	3.730	1.00	0.00	C
ATOM	2156	CG2	VAL	142	0.007	6.029	5.247	1.00	0.00	C
ATOM	2157	H	VAL	142	-2.277	4.469	5.236	1.00	0.00	H
ATOM	2158	HA	VAL	142	-1.103	5.368	2.896	1.00	0.00	H
ATOM	2159	HB	VAL	142	0.173	3.911	5.212	1.00	0.00	H
ATOM	2160	1HG1	VAL	142	1.604	3.721	3.265	1.00	0.00	H
ATOM	2161	2HG1	VAL	142	1.477	5.456	2.973	1.00	0.00	H
ATOM	2162	3HG1	VAL	142	2.298	4.861	4.415	1.00	0.00	H
ATOM	2163	1HG2	VAL	142	0.484	6.823	4.693	1.00	0.00	H
ATOM	2164	2HG2	VAL	142	-1.044	6.253	5.364	1.00	0.00	H
ATOM	2165	3HG2	VAL	142	0.466	5.942	6.220	1.00	0.00	H
ATOM	2166	N	GLN	143	-0.834	2.097	3.274	1.00	0.00	N
ATOM	2167	CA	GLN	143	-0.591	0.850	2.558	1.00	0.00	C
ATOM	2168	C	GLN	143	-1.450	0.768	1.301	1.00	0.00	C
ATOM	2169	O	GLN	143	-0.945	0.507	0.209	1.00	0.00	O
ATOM	2170	CB	GLN	143	-0.881	-0.348	3.466	1.00	0.00	C
ATOM	2171	CG	GLN	143	0.016	-0.413	4.691	1.00	0.00	C
ATOM	2172	CD	GLN	143	1.482	-0.567	4.335	1.00	0.00	C
ATOM	2173	OE1	GLN	143	2.103	0.356	3.805	1.00	0.00	O
ATOM	2174	NE2	GLN	143	2.042	-1.734	4.624	1.00	0.00	N
ATOM	2175	H	GLN	143	-1.068	2.065	4.226	1.00	0.00	H
ATOM	2176	HA	GLN	143	0.449	0.830	2.272	1.00	0.00	H
ATOM	2177	2HB3	GLN	143	-0.743	-1.256	2.898	1.00	0.00	H
ATOM	2178	2HG3	GLN	143	-0.283	-1.257	5.295	1.00	0.00	H
ATOM	2179	1HE2	GLN	143	1.487	-2.423	5.045	1.00	0.00	H
ATOM	2180	2HE2	GLN	143	2.989	-1.861	4.404	1.00	0.00	H
ATOM	2181	HB2	GLN	143	-1.903	-0.286	3.837	1.00	0.00	H
ATOM	2182	HG2	GLN	143	-0.080	0.511	5.262	1.00	0.00	H
ATOM	2183	N	MET	144	-2.751	0.994	1.461	1.00	0.00	N
ATOM	2184	CA	MET	144	-3.678	0.947	0.338	1.00	0.00	C
ATOM	2185	C	MET	144	-3.170	1.796	-0.823	1.00	0.00	C
ATOM	2186	O	MET	144	-3.088	1.327	-1.958	1.00	0.00	O
ATOM	2187	CB	MET	144	-5.063	1.432	0.771	1.00	0.00	C
ATOM	2188	CG	MET	144	-6.022	1.648	-0.387	1.00	0.00	C
ATOM	2189	SD	MET	144	-7.675	2.119	0.158	1.00	0.00	S
ATOM	2190	CE	MET	144	-7.409	3.835	0.600	1.00	0.00	C
ATOM	2191	H	MET	144	-3.094	1.197	2.357	1.00	0.00	H
ATOM	2192	HA	MET	144	-3.751	-0.079	0.014	1.00	0.00	H
ATOM	2193	2HB3	MET	144	-4.954	2.369	1.299	1.00	0.00	H
ATOM	2194	2HG3	MET	144	-6.093	0.730	-0.952	1.00	0.00	H
ATOM	2195	1HE	MET	144	-7.710	3.994	1.625	1.00	0.00	H
ATOM	2196	2HE	MET	144	-6.362	4.076	0.491	1.00	0.00	H
ATOM	2197	3HE	MET	144	-7.995	4.469	-0.049	1.00	0.00	H
ATOM	2198	HB2	MET	144	-5.525	0.686	1.416	1.00	0.00	H
ATOM	2199	HG2	MET	144	-5.653	2.454	-1.019	1.00	0.00	H
ATOM	2200	N	MET	145	-2.828	3.048	-0.531	1.00	0.00	N
ATOM	2201	CA	MET	145	-2.327	3.960	-1.551	1.00	0.00	C
ATOM	2202	C	MET	145	-1.139	3.350	-2.290	1.00	0.00	C
ATOM	2203	O	MET	145	-1.032	3.459	-3.513	1.00	0.00	O
ATOM	2204	CB	MET	145	-1.919	5.292	-0.917	1.00	0.00	C
ATOM	2205	CG	MET	145	-3.072	6.028	-0.254	1.00	0.00	C
ATOM	2206	SD	MET	145	-4.402	6.420	-1.407	1.00	0.00	S
ATOM	2207	CE	MET	145	-3.545	7.493	-2.558	1.00	0.00	C
ATOM	2208	H	MET	145	-2.914	3.363	0.392	1.00	0.00	H
ATOM	2209	HA	MET	145	-3.123	4.136	-2.258	1.00	0.00	H
ATOM	2210	2HB3	MET	145	-1.508	5.931	-1.685	1.00	0.00	H
ATOM	2211	2HG3	MET	145	-2.697	6.947	0.169	1.00	0.00	H
ATOM	2212	1HE	MET	145	-3.219	8.384	-2.043	1.00	0.00	H
ATOM	2213	2HE	MET	145	-2.689	6.975	-2.963	1.00	0.00	H

```
ATOM   2214 3HE  MET   145      -4.215   7.765  -3.361  1.00  0.00           H
ATOM   2215  HB2 MET   145      -1.176   5.112  -0.140  1.00  0.00           H
ATOM   2216  HG2 MET   145      -3.506   5.399   0.525  1.00  0.00           H
ATOM   2217  N   THR   146      -0.247   2.711  -1.541  1.00  0.00           N
ATOM   2218  CA  THR   146       0.933   2.084  -2.125  1.00  0.00           C
ATOM   2219  C   THR   146       0.735   0.581  -2.286  1.00  0.00           C
ATOM   2220  O   THR   146       1.653  -0.205  -2.049  1.00  0.00           O
ATOM   2221  CB  THR   146       2.186   2.335  -1.264  1.00  0.00           C
ATOM   2222  OG1 THR   146       1.982   1.829   0.059  1.00  0.00           O
ATOM   2223  CG2 THR   146       2.507   3.822  -1.199  1.00  0.00           C
ATOM   2224  H   THR   146      -0.388   2.657  -0.572  1.00  0.00           H
ATOM   2225  HA  THR   146       1.094   2.523  -3.098  1.00  0.00           H
ATOM   2226  HB  THR   146       3.022   1.821  -1.714  1.00  0.00           H
ATOM   2227 1HG  THR   146       1.998   2.555   0.687  1.00  0.00           H
ATOM   2228 1HG2 THR   146       2.910   4.062  -0.225  1.00  0.00           H
ATOM   2229 2HG2 THR   146       1.606   4.394  -1.365  1.00  0.00           H
ATOM   2230 3HG2 THR   146       3.234   4.066  -1.959  1.00  0.00           H
ATOM   2231  N   ALA   147      -0.466   0.187  -2.693  1.00  0.00           N
ATOM   2232  CA  ALA   147      -0.784  -1.222  -2.889  1.00  0.00           C
ATOM   2233  C   ALA   147      -1.459  -1.451  -4.238  1.00  0.00           C
ATOM   2234  O   ALA   147      -2.600  -1.038  -4.450  1.00  0.00           O
ATOM   2235  CB  ALA   147      -1.670  -1.726  -1.762  1.00  0.00           C
ATOM   2236  H   ALA   147      -1.158   0.861  -2.867  1.00  0.00           H
ATOM   2237  HA  ALA   147       0.142  -1.777  -2.864  1.00  0.00           H
ATOM   2238 1HB  ALA   147      -2.543  -2.204  -2.176  1.00  0.00           H
ATOM   2239 2HB  ALA   147      -1.121  -2.440  -1.164  1.00  0.00           H
ATOM   2240 3HB  ALA   147      -1.973  -0.895  -1.143  1.00  0.00           H
ATOM   2241  N   LYS   148      -0.748  -2.108  -5.147  1.00  0.00           N
ATOM   2242  CA  LYS   148      -1.278  -2.392  -6.475  1.00  0.00           C
ATOM   2243  C   LYS   148      -1.700  -3.854  -6.590  1.00  0.00           C
ATOM   2244  O   LYS   148      -0.836  -4.725  -6.677  1.00  0.00           O
ATOM   2245  CB  LYS   148      -0.235  -2.066  -7.545  1.00  0.00           C
ATOM   2246  CG  LYS   148       0.266  -0.634  -7.487  1.00  0.00           C
ATOM   2247  CD  LYS   148      -0.830   0.356  -7.842  1.00  0.00           C
ATOM   2248  CE  LYS   148      -1.056   0.421  -9.345  1.00  0.00           C
ATOM   2249  NZ  LYS   148       0.029   1.171 -10.037  1.00  0.00           N
ATOM   2250  H   LYS   148       0.156  -2.412  -4.917  1.00  0.00           H
ATOM   2251  HA  LYS   148      -2.145  -1.767  -6.626  1.00  0.00           H
ATOM   2252 2HB3 LYS   148      -0.670  -2.234  -8.520  1.00  0.00           H
ATOM   2253 2HG3 LYS   148       1.082  -0.519  -8.186  1.00  0.00           H
ATOM   2254 2HD3 LYS   148      -0.549   1.338  -7.486  1.00  0.00           H
ATOM   2255 2HE3 LYS   148      -1.998   0.915  -9.533  1.00  0.00           H
ATOM   2256 1HZ  LYS   148       0.272   0.703 -10.934  1.00  0.00           H
ATOM   2257 2HZ  LYS   148       0.877   1.205  -9.436  1.00  0.00           H
ATOM   2258 3HZ  LYS   148      -0.279   2.144 -10.238  1.00  0.00           H
ATOM   2259  HB2 LYS   148       0.641  -2.699  -7.404  1.00  0.00           H
ATOM   2260  HG2 LYS   148       0.610  -0.408  -6.476  1.00  0.00           H
ATOM   2261  HD2 LYS   148      -1.765   0.045  -7.378  1.00  0.00           H
ATOM   2262  HE2 LYS   148      -1.074  -0.589  -9.755  1.00  0.00           H
ATOM   2263  HXT LYS   148      -2.744  -4.128  -6.596  1.00  0.00           H
TER    2264      LYS   148
HETATM 2265 CA   CA    149      26.033  -5.982  -2.893  1.00  0.00          Ca
HETATM 2266 CA   CA    150      30.425   3.340  -5.149  1.00  0.00          Ca
HETATM 2267 CA   CA    151     -12.834   7.964  11.921  1.00  0.00          Ca
HETATM 2268 CA   CA    152      -8.178  -0.981  10.612  1.00  0.00          Ca
HETATM 2269  F1  UNK     1      14.842   2.283   2.026  1.00  0.00           F
HETATM 2270  C1  UNK     1      15.284   3.539   2.110  1.00  0.00           C
HETATM 2271  C2  UNK     1      15.439   3.944   3.597  1.00  0.00           C
HETATM 2272  O1  UNK     1      15.540   5.361   3.780  1.00  0.00           O
HETATM 2273  C3  UNK     1      16.693   6.001   3.450  1.00  0.00           C
HETATM 2274  F2  UNK     1      17.568   5.963   4.485  1.00  0.00           F
HETATM 2275  F3  UNK     1      16.425   3.638   1.423  1.00  0.00           F
HETATM 2276  F4  UNK     1      14.371   4.324   1.534  1.00  0.00           F
HETATM 2277  C4  UNK     1      16.498   3.148   4.391  1.00  0.00           C
HETATM 2278  F5  UNK     1      16.186   1.854   4.403  1.00  0.00           F
HETATM 2279  F6  UNK     1      16.515   3.568   5.654  1.00  0.00           F
HETATM 2280  F7  UNK     1      17.720   3.283   3.871  1.00  0.00           F
HETATM 2281  H1  UNK     1      16.459   7.049   3.274  1.00  0.00           H
HETATM 2282  H2  UNK     1      17.168   5.559   2.573  1.00  0.00           H
HETATM 2283  H3  UNK     1      14.480   3.707   4.063  1.00  0.00           H
ENDMDL
CONECT 2270 2269 2271 2275 2276
CONECT 2271 2270 2272 2277 2283
CONECT 2273 2272 2274 2281 2282
CONECT 2277 2271 2278 2279 2280
CONECT 2269 2270
CONECT 2274 2273
```

```
CONECT 2275 2270
CONECT 2276 2270
CONECT 2278 2277
CONECT 2279 2277
CONECT 2280 2277
CONECT 2281 2273
CONECT 2282 2273
CONECT 2283 2271
CONECT 2272 2271 2273
MODEL        2
ATOM      1  N   ALA     1      32.284  14.224   7.039  1.00  0.00           N
ATOM      2  CA  ALA     1      31.935  12.933   7.622  1.00  0.00           C
ATOM      3  C   ALA     1      32.819  12.615   8.823  1.00  0.00           C
ATOM      4  O   ALA     1      33.074  11.451   9.128  1.00  0.00           O
ATOM      5  CB  ALA     1      32.050  11.833   6.576  1.00  0.00           C
ATOM      6  HA  ALA     1      30.906  12.981   7.946  1.00  0.00           H
ATOM      7 1HB  ALA     1      33.077  11.746   6.256  1.00  0.00           H
ATOM      8 2HB  ALA     1      31.724  10.897   7.000  1.00  0.00           H
ATOM      9 3HB  ALA     1      31.428  12.078   5.726  1.00  0.00           H
ATOM     10 1H   ALA     1      32.353  14.305   6.064  1.00  0.00           H
ATOM     11  H1  ALA     1      31.593  14.910   7.301  1.00  0.00           H
ATOM     12  H2  ALA     1      33.191  14.513   7.380  1.00  0.00           H
ATOM     13  N   ASP     2      33.284  13.660   9.501  1.00  0.00           N
ATOM     14  CA  ASP     2      34.139  13.493  10.671  1.00  0.00           C
ATOM     15  C   ASP     2      33.316  13.107  11.895  1.00  0.00           C
ATOM     16  O   ASP     2      33.809  12.435  12.800  1.00  0.00           O
ATOM     17  CB  ASP     2      34.920  14.778  10.946  1.00  0.00           C
ATOM     18  CG  ASP     2      35.960  15.065   9.883  1.00  0.00           C
ATOM     19  OD1 ASP     2      36.455  14.100   9.263  1.00  0.00           O
ATOM     20  OD2 ASP     2      36.282  16.254   9.670  1.00  0.00           O
ATOM     21  H   ASP     2      33.046  14.564   9.209  1.00  0.00           H
ATOM     22  HA  ASP     2      34.840  12.697  10.457  1.00  0.00           H
ATOM     23 2HB3 ASP     2      35.419  14.691  11.900  1.00  0.00           H
ATOM     24  HB2 ASP     2      34.232  15.625  10.963  1.00  0.00           H
ATOM     25  N   GLN     3      32.061  13.542  11.917  1.00  0.00           N
ATOM     26  CA  GLN     3      31.170  13.244  13.033  1.00  0.00           C
ATOM     27  C   GLN     3      30.420  11.938  12.799  1.00  0.00           C
ATOM     28  O   GLN     3      29.274  11.781  13.220  1.00  0.00           O
ATOM     29  CB  GLN     3      30.173  14.388  13.236  1.00  0.00           C
ATOM     30  CG  GLN     3      30.768  15.591  13.952  1.00  0.00           C
ATOM     31  CD  GLN     3      31.160  15.285  15.383  1.00  0.00           C
ATOM     32  OE1 GLN     3      30.364  15.448  16.305  1.00  0.00           O
ATOM     33  NE2 GLN     3      32.394  14.835  15.573  1.00  0.00           N
ATOM     34  H   GLN     3      31.725  14.075  11.167  1.00  0.00           H
ATOM     35  HA  GLN     3      31.773  13.144  13.921  1.00  0.00           H
ATOM     36 2HB3 GLN     3      29.341  14.025  13.820  1.00  0.00           H
ATOM     37 2HG3 GLN     3      30.034  16.385  13.957  1.00  0.00           H
ATOM     38 1HE2 GLN     3      32.975  14.729  14.790  1.00  0.00           H
ATOM     39 2HE2 GLN     3      32.676  14.629  16.489  1.00  0.00           H
ATOM     40  HB2 GLN     3      29.825  14.745  12.265  1.00  0.00           H
ATOM     41  HG2 GLN     3      31.668  15.915  13.431  1.00  0.00           H
ATOM     42  N   LEU     4      31.074  11.001  12.121  1.00  0.00           N
ATOM     43  CA  LEU     4      30.470   9.705  11.829  1.00  0.00           C
ATOM     44  C   LEU     4      31.451   8.570  12.106  1.00  0.00           C
ATOM     45  O   LEU     4      31.679   7.710  11.255  1.00  0.00           O
ATOM     46  CB  LEU     4      30.008   9.651  10.371  1.00  0.00           C
ATOM     47  CG  LEU     4      28.937  10.665   9.968  1.00  0.00           C
ATOM     48  CD1 LEU     4      29.125  11.096   8.522  1.00  0.00           C
ATOM     49  CD2 LEU     4      27.547  10.083  10.177  1.00  0.00           C
ATOM     50  H   LEU     4      31.985  11.180  11.811  1.00  0.00           H
ATOM     51  HA  LEU     4      29.612   9.586  12.475  1.00  0.00           H
ATOM     52 2HB3 LEU     4      29.616   8.661  10.186  1.00  0.00           H
ATOM     53  HG  LEU     4      29.028  11.544  10.593  1.00  0.00           H
ATOM     54 1HD1 LEU     4      28.814  12.125   8.411  1.00  0.00           H
ATOM     55 2HD1 LEU     4      28.528  10.468   7.879  1.00  0.00           H
ATOM     56 3HD1 LEU     4      30.167  11.001   8.253  1.00  0.00           H
ATOM     57 1HD2 LEU     4      27.242  10.235  11.200  1.00  0.00           H
ATOM     58 2HD2 LEU     4      27.566   9.024   9.961  1.00  0.00           H
ATOM     59 3HD2 LEU     4      26.848  10.574   9.516  1.00  0.00           H
ATOM     60  HB2 LEU     4      30.850   9.873   9.715  1.00  0.00           H
ATOM     61  N   THR     5      32.031   8.574  13.302  1.00  0.00           N
ATOM     62  CA  THR     5      32.988   7.545  13.691  1.00  0.00           C
ATOM     63  C   THR     5      32.281   6.246  14.057  1.00  0.00           C
ATOM     64  O   THR     5      31.062   6.130  13.914  1.00  0.00           O
ATOM     65  CB  THR     5      33.848   8.002  14.883  1.00  0.00           C
ATOM     66  OG1 THR     5      33.127   8.955  15.673  1.00  0.00           O
ATOM     67  CG2 THR     5      35.154   8.618  14.406  1.00  0.00           C
```

ATOM	68	H	THR	5	31.807	9.286	13.936	1.00	0.00	H
ATOM	69	HA	THR	5	33.641	7.364	12.850	1.00	0.00	H
ATOM	70	HB	THR	5	34.076	7.140	15.495	1.00	0.00	H
ATOM	71	1HG	THR	5	33.583	9.093	16.507	1.00	0.00	H
ATOM	72	1HG2	THR	5	35.488	9.355	15.121	1.00	0.00	H
ATOM	73	2HG2	THR	5	34.999	9.090	13.447	1.00	0.00	H
ATOM	74	3HG2	THR	5	35.902	7.846	14.310	1.00	0.00	H
ATOM	75	N	GLU	6	33.049	5.271	14.530	1.00	0.00	N
ATOM	76	CA	GLU	6	32.494	3.979	14.915	1.00	0.00	C
ATOM	77	C	GLU	6	31.081	4.138	15.473	1.00	0.00	C
ATOM	78	O	GLU	6	30.216	3.292	15.247	1.00	0.00	O
ATOM	79	CB	GLU	6	33.389	3.305	15.955	1.00	0.00	C
ATOM	80	CG	GLU	6	34.713	2.813	15.392	1.00	0.00	C
ATOM	81	CD	GLU	6	34.533	1.741	14.335	1.00	0.00	C
ATOM	82	OE1	GLU	6	33.746	0.800	14.570	1.00	0.00	O
ATOM	83	OE2	GLU	6	35.180	1.844	13.272	1.00	0.00	O
ATOM	84	H	GLU	6	34.013	5.424	14.622	1.00	0.00	H
ATOM	85	HA	GLU	6	32.452	3.360	14.033	1.00	0.00	H
ATOM	86	2HB3	GLU	6	32.862	2.457	16.371	1.00	0.00	H
ATOM	87	2HG3	GLU	6	35.304	2.405	16.200	1.00	0.00	H
ATOM	88	HB2	GLU	6	33.638	4.018	16.741	1.00	0.00	H
ATOM	89	HG2	GLU	6	35.239	3.642	14.921	1.00	0.00	H
ATOM	90	N	GLU	7	30.858	5.228	16.199	1.00	0.00	N
ATOM	91	CA	GLU	7	29.551	5.497	16.788	1.00	0.00	C
ATOM	92	C	GLU	7	28.459	5.474	15.723	1.00	0.00	C
ATOM	93	O	GLU	7	27.475	4.744	15.846	1.00	0.00	O
ATOM	94	CB	GLU	7	29.558	6.855	17.498	1.00	0.00	C
ATOM	95	CG	GLU	7	30.559	7.840	16.921	1.00	0.00	C
ATOM	96	CD	GLU	7	31.905	7.777	17.619	1.00	0.00	C
ATOM	97	OE1	GLU	7	32.722	6.906	17.255	1.00	0.00	O
ATOM	98	OE2	GLU	7	32.139	8.600	18.528	1.00	0.00	O
ATOM	99	H	GLU	7	31.588	5.868	16.343	1.00	0.00	H
ATOM	100	HA	GLU	7	29.347	4.723	17.512	1.00	0.00	H
ATOM	101	2HB3	GLU	7	29.795	6.699	18.540	1.00	0.00	H
ATOM	102	2HG3	GLU	7	30.164	8.839	17.022	1.00	0.00	H
ATOM	103	HB2	GLU	7	28.578	7.324	17.396	1.00	0.00	H
ATOM	104	HG2	GLU	7	30.733	7.609	15.871	1.00	0.00	H
ATOM	105	N	GLN	8	28.639	6.276	14.680	1.00	0.00	N
ATOM	106	CA	GLN	8	27.668	6.349	13.595	1.00	0.00	C
ATOM	107	C	GLN	8	27.826	5.165	12.644	1.00	0.00	C
ATOM	108	O	GLN	8	26.844	4.525	12.268	1.00	0.00	O
ATOM	109	CB	GLN	8	27.828	7.662	12.823	1.00	0.00	C
ATOM	110	CG	GLN	8	27.441	8.891	13.630	1.00	0.00	C
ATOM	111	CD	GLN	8	25.957	8.949	13.931	1.00	0.00	C
ATOM	112	OE1	GLN	8	25.180	9.527	13.170	1.00	0.00	O
ATOM	113	NE2	GLN	8	25.554	8.348	15.045	1.00	0.00	N
ATOM	114	H	GLN	8	29.443	6.834	14.639	1.00	0.00	H
ATOM	115	HA	GLN	8	26.681	6.316	14.031	1.00	0.00	H
ATOM	116	2HB3	GLN	8	27.205	7.626	11.942	1.00	0.00	H
ATOM	117	2HG3	GLN	8	27.716	9.773	13.071	1.00	0.00	H
ATOM	118	1HE2	GLN	8	26.229	7.909	15.603	1.00	0.00	H
ATOM	119	2HE2	GLN	8	24.600	8.371	15.265	1.00	0.00	H
ATOM	120	HB2	GLN	8	28.873	7.796	12.544	1.00	0.00	H
ATOM	121	HG2	GLN	8	27.960	8.874	14.590	1.00	0.00	H
ATOM	122	N	ILE	9	29.065	4.884	12.259	1.00	0.00	N
ATOM	123	CA	ILE	9	29.350	3.778	11.352	1.00	0.00	C
ATOM	124	C	ILE	9	28.551	2.536	11.734	1.00	0.00	C
ATOM	125	O	ILE	9	28.002	1.850	10.871	1.00	0.00	O
ATOM	126	CB	ILE	9	30.850	3.427	11.347	1.00	0.00	C
ATOM	127	CG2	ILE	9	31.110	2.230	10.445	1.00	0.00	C
ATOM	128	CG1	ILE	9	31.676	4.634	10.894	1.00	0.00	C
ATOM	129	CD1	ILE	9	33.165	4.449	11.081	1.00	0.00	C
ATOM	130	H	ILE	9	29.806	5.432	12.591	1.00	0.00	H
ATOM	131	HA	ILE	9	29.068	4.083	10.357	1.00	0.00	H
ATOM	132	HB	ILE	9	31.138	3.159	12.352	1.00	0.00	H
ATOM	133	1HG2	ILE	9	32.036	1.759	10.733	1.00	0.00	H
ATOM	134	2HG2	ILE	9	30.301	1.524	10.542	1.00	0.00	H
ATOM	135	3HG2	ILE	9	31.178	2.562	9.419	1.00	0.00	H
ATOM	136	2HG3	ILE	9	31.374	5.502	11.464	1.00	0.00	H
ATOM	137	1HD1	ILE	9	33.680	5.356	10.797	1.00	0.00	H
ATOM	138	2HD1	ILE	9	33.375	4.224	12.116	1.00	0.00	H
ATOM	139	3HD1	ILE	9	33.508	3.634	10.460	1.00	0.00	H
ATOM	140	HG2	ILE	9	31.508	4.811	9.832	1.00	0.00	H
ATOM	141	N	ALA	10	28.489	2.254	13.031	1.00	0.00	N
ATOM	142	CA	ALA	10	27.752	1.097	13.527	1.00	0.00	C
ATOM	143	C	ALA	10	26.266	1.211	13.201	1.00	0.00	C
ATOM	144	O	ALA	10	25.641	0.242	12.774	1.00	0.00	O

ATOM	145	CB	ALA	10	27.957	0.946	15.027	1.00	0.00	C
ATOM	146	H	ALA	10	28.947	2.839	13.670	1.00	0.00	H
ATOM	147	HA	ALA	10	28.150	0.216	13.043	1.00	0.00	H
ATOM	148	1HB	ALA	10	28.589	1.745	15.385	1.00	0.00	H
ATOM	149	2HB	ALA	10	27.000	0.989	15.526	1.00	0.00	H
ATOM	150	3HB	ALA	10	28.425	-0.005	15.230	1.00	0.00	H
ATOM	151	N	GLU	11	25.712	2.400	13.406	1.00	0.00	N
ATOM	152	CA	GLU	11	24.298	2.639	13.134	1.00	0.00	C
ATOM	153	C	GLU	11	23.996	2.487	11.649	1.00	0.00	C
ATOM	154	O	GLU	11	23.146	1.687	11.253	1.00	0.00	O
ATOM	155	CB	GLU	11	23.893	4.037	13.609	1.00	0.00	C
ATOM	156	CG	GLU	11	23.565	4.105	15.091	1.00	0.00	C
ATOM	157	CD	GLU	11	22.191	3.550	15.410	1.00	0.00	C
ATOM	158	OE1	GLU	11	22.081	2.325	15.625	1.00	0.00	O
ATOM	159	OE2	GLU	11	21.224	4.340	15.445	1.00	0.00	O
ATOM	160	H	GLU	11	26.264	3.136	13.746	1.00	0.00	H
ATOM	161	HA	GLU	11	23.727	1.905	13.683	1.00	0.00	H
ATOM	162	2HB3	GLU	11	23.023	4.353	13.054	1.00	0.00	H
ATOM	163	2HG3	GLU	11	23.601	5.137	15.408	1.00	0.00	H
ATOM	164	HB2	GLU	11	24.720	4.728	13.449	1.00	0.00	H
ATOM	165	HG2	GLU	11	24.290	3.515	15.653	1.00	0.00	H
ATOM	166	N	PHE	12	24.697	3.262	10.826	1.00	0.00	N
ATOM	167	CA	PHE	12	24.502	3.214	9.381	1.00	0.00	C
ATOM	168	C	PHE	12	24.769	1.812	8.841	1.00	0.00	C
ATOM	169	O	PHE	12	24.205	1.409	7.823	1.00	0.00	O
ATOM	170	CB	PHE	12	25.422	4.222	8.689	1.00	0.00	C
ATOM	171	CG	PHE	12	24.916	5.634	8.748	1.00	0.00	C
ATOM	172	CD1	PHE	12	24.084	6.129	7.756	1.00	0.00	C
ATOM	173	CE1	PHE	12	23.619	7.431	7.808	1.00	0.00	C
ATOM	174	CZ	PHE	12	23.980	8.251	8.859	1.00	0.00	C
ATOM	175	CE2	PHE	12	24.807	7.770	9.853	1.00	0.00	C
ATOM	176	CD2	PHE	12	25.272	6.470	9.796	1.00	0.00	C
ATOM	177	H	PHE	12	25.359	3.880	11.200	1.00	0.00	H
ATOM	178	HA	PHE	12	23.477	3.477	9.177	1.00	0.00	H
ATOM	179	2HB3	PHE	12	25.527	3.949	7.650	1.00	0.00	H
ATOM	180	1HD	PHE	12	23.802	5.489	6.934	1.00	0.00	H
ATOM	181	1HE	PHE	12	22.972	7.803	7.029	1.00	0.00	H
ATOM	182	HZ	PHE	12	23.617	9.267	8.901	1.00	0.00	H
ATOM	183	2HE	PHE	12	25.091	8.409	10.677	1.00	0.00	H
ATOM	184	2HD	PHE	12	25.919	6.093	10.576	1.00	0.00	H
ATOM	185	HB2	PHE	12	26.398	4.219	9.177	1.00	0.00	H
ATOM	186	N	LYS	13	25.629	1.072	9.531	1.00	0.00	N
ATOM	187	CA	LYS	13	25.972	-0.285	9.124	1.00	0.00	C
ATOM	188	C	LYS	13	24.713	-1.113	8.879	1.00	0.00	C
ATOM	189	O	LYS	13	24.532	-1.677	7.801	1.00	0.00	O
ATOM	190	CB	LYS	13	26.836	-0.957	10.193	1.00	0.00	C
ATOM	191	CG	LYS	13	27.469	-2.259	9.732	1.00	0.00	C
ATOM	192	CD	LYS	13	28.691	-2.611	10.565	1.00	0.00	C
ATOM	193	CE	LYS	13	28.300	-3.265	11.879	1.00	0.00	C
ATOM	194	NZ	LYS	13	29.479	-3.469	12.768	1.00	0.00	N
ATOM	195	H	LYS	13	26.046	1.450	10.337	1.00	0.00	H
ATOM	196	HA	LYS	13	26.531	-0.225	8.206	1.00	0.00	H
ATOM	197	2HB3	LYS	13	26.222	-1.165	11.058	1.00	0.00	H
ATOM	198	2HG3	LYS	13	27.764	-2.160	8.699	1.00	0.00	H
ATOM	199	2HD3	LYS	13	29.245	-1.705	10.773	1.00	0.00	H
ATOM	200	2HE3	LYS	13	27.849	-4.224	11.670	1.00	0.00	H
ATOM	201	1HZ	LYS	13	30.134	-2.666	12.683	1.00	0.00	H
ATOM	202	2HZ	LYS	13	29.980	-4.341	12.503	1.00	0.00	H
ATOM	203	3HZ	LYS	13	29.170	-3.548	13.758	1.00	0.00	H
ATOM	204	HB2	LYS	13	27.660	-0.297	10.462	1.00	0.00	H
ATOM	205	HG2	LYS	13	26.748	-3.070	9.837	1.00	0.00	H
ATOM	206	HD2	LYS	13	29.318	-3.312	10.013	1.00	0.00	H
ATOM	207	HE2	LYS	13	27.595	-2.626	12.409	1.00	0.00	H
ATOM	208	N	GLU	14	23.849	-1.178	9.888	1.00	0.00	N
ATOM	209	CA	GLU	14	22.607	-1.936	9.778	1.00	0.00	C
ATOM	210	C	GLU	14	21.827	-1.530	8.532	1.00	0.00	C
ATOM	211	O	GLU	14	21.323	-2.379	7.797	1.00	0.00	O
ATOM	212	CB	GLU	14	21.743	-1.724	11.025	1.00	0.00	C
ATOM	213	CG	GLU	14	22.437	-2.110	12.320	1.00	0.00	C
ATOM	214	CD	GLU	14	22.870	-3.563	12.339	1.00	0.00	C
ATOM	215	OE1	GLU	14	22.041	-4.433	11.995	1.00	0.00	O
ATOM	216	OE2	GLU	14	24.035	-3.830	12.696	1.00	0.00	O
ATOM	217	H	GLU	14	24.048	-0.705	10.722	1.00	0.00	H
ATOM	218	HA	GLU	14	22.862	-2.983	9.704	1.00	0.00	H
ATOM	219	2HB3	GLU	14	20.847	-2.318	10.931	1.00	0.00	H
ATOM	220	2HG3	GLU	14	21.757	-1.941	13.143	1.00	0.00	H
ATOM	221	HB2	GLU	14	21.490	-0.667	11.117	1.00	0.00	H

ATOM	222	HG2	GLU	14	23.336	-1.505	12.445	1.00	0.00	H
ATOM	223	N	ALA	15	21.734	-0.224	8.299	1.00	0.00	N
ATOM	224	CA	ALA	15	21.018	0.296	7.140	1.00	0.00	C
ATOM	225	C	ALA	15	21.733	-0.070	5.843	1.00	0.00	C
ATOM	226	O	ALA	15	21.164	0.037	4.757	1.00	0.00	O
ATOM	227	CB	ALA	15	20.861	1.804	7.249	1.00	0.00	C
ATOM	228	H	ALA	15	22.158	0.404	8.918	1.00	0.00	H
ATOM	229	HA	ALA	15	20.032	-0.146	7.131	1.00	0.00	H
ATOM	230	1HB	ALA	15	20.356	2.178	6.370	1.00	0.00	H
ATOM	231	2HB	ALA	15	20.281	2.042	8.128	1.00	0.00	H
ATOM	232	3HB	ALA	15	21.837	2.262	7.326	1.00	0.00	H
ATOM	233	N	PHE	16	22.983	-0.505	5.964	1.00	0.00	N
ATOM	234	CA	PHE	16	23.777	-0.886	4.801	1.00	0.00	C
ATOM	235	C	PHE	16	23.979	-2.397	4.752	1.00	0.00	C
ATOM	236	O	PHE	16	24.518	-2.932	3.782	1.00	0.00	O
ATOM	237	CB	PHE	16	25.134	-0.180	4.829	1.00	0.00	C
ATOM	238	CG	PHE	16	25.998	-0.492	3.641	1.00	0.00	C
ATOM	239	CD1	PHE	16	25.835	0.199	2.450	1.00	0.00	C
ATOM	240	CE1	PHE	16	26.628	-0.089	1.354	1.00	0.00	C
ATOM	241	CZ	PHE	16	27.595	-1.072	1.443	1.00	0.00	C
ATOM	242	CE2	PHE	16	27.767	-1.765	2.623	1.00	0.00	C
ATOM	243	CD2	PHE	16	26.971	-1.475	3.716	1.00	0.00	C
ATOM	244	H	PHE	16	23.382	-0.570	6.858	1.00	0.00	H
ATOM	245	HA	PHE	16	23.238	-0.577	3.919	1.00	0.00	H
ATOM	246	2HB3	PHE	16	25.669	-0.477	5.718	1.00	0.00	H
ATOM	247	1HD	PHE	16	25.080	0.967	2.381	1.00	0.00	H
ATOM	248	1HE	PHE	16	26.494	0.454	0.431	1.00	0.00	H
ATOM	249	HZ	PHE	16	28.217	-1.297	0.587	1.00	0.00	H
ATOM	250	2HE	PHE	16	28.522	-2.534	2.695	1.00	0.00	H
ATOM	251	2HD	PHE	16	27.105	-2.018	4.639	1.00	0.00	H
ATOM	252	HB2	PHE	16	24.982	0.901	4.828	1.00	0.00	H
ATOM	253	N	SER	17	23.545	-3.081	5.806	1.00	0.00	N
ATOM	254	CA	SER	17	23.683	-4.530	5.887	1.00	0.00	C
ATOM	255	C	SER	17	22.461	-5.225	5.294	1.00	0.00	C
ATOM	256	O	SER	17	22.563	-6.321	4.742	1.00	0.00	O
ATOM	257	CB	SER	17	23.878	-4.966	7.342	1.00	0.00	C
ATOM	258	OG	SER	17	25.252	-4.993	7.687	1.00	0.00	O
ATOM	259	H	SER	17	23.127	-2.598	6.551	1.00	0.00	H
ATOM	260	HA	SER	17	24.555	-4.813	5.317	1.00	0.00	H
ATOM	261	2HB3	SER	17	23.466	-5.954	7.476	1.00	0.00	H
ATOM	262	HG	SER	17	25.673	-5.748	7.266	1.00	0.00	H
ATOM	263	HB2	SER	17	23.369	-4.265	8.003	1.00	0.00	H
ATOM	264	N	LEU	18	21.308	-4.582	5.415	1.00	0.00	N
ATOM	265	CA	LEU	18	20.063	-5.136	4.891	1.00	0.00	C
ATOM	266	C	LEU	18	20.015	-5.030	3.371	1.00	0.00	C
ATOM	267	O	LEU	18	19.550	-5.943	2.688	1.00	0.00	O
ATOM	268	CB	LEU	18	18.861	-4.412	5.504	1.00	0.00	C
ATOM	269	CG	LEU	18	18.397	-3.150	4.774	1.00	0.00	C
ATOM	270	CD1	LEU	18	16.920	-2.894	5.038	1.00	0.00	C
ATOM	271	CD2	LEU	18	19.232	-1.950	5.193	1.00	0.00	C
ATOM	272	H	LEU	18	21.288	-3.712	5.867	1.00	0.00	H
ATOM	273	HA	LEU	18	20.024	-6.179	5.170	1.00	0.00	H
ATOM	274	2HB3	LEU	18	19.123	-4.130	6.513	1.00	0.00	H
ATOM	275	HG	LEU	18	18.523	-3.290	3.708	1.00	0.00	H
ATOM	276	1HD1	LEU	18	16.785	-2.610	6.072	1.00	0.00	H
ATOM	277	2HD1	LEU	18	16.356	-3.792	4.835	1.00	0.00	H
ATOM	278	3HD1	LEU	18	16.573	-2.099	4.397	1.00	0.00	H
ATOM	279	1HD2	LEU	18	18.647	-1.049	5.086	1.00	0.00	H
ATOM	280	2HD2	LEU	18	20.108	-1.887	4.566	1.00	0.00	H
ATOM	281	3HD2	LEU	18	19.534	-2.066	6.223	1.00	0.00	H
ATOM	282	HB2	LEU	18	17.994	-5.071	5.494	1.00	0.00	H
ATOM	283	N	PHE	19	20.502	-3.911	2.846	1.00	0.00	N
ATOM	284	CA	PHE	19	20.520	-3.685	1.407	1.00	0.00	C
ATOM	285	C	PHE	19	21.480	-4.648	0.715	1.00	0.00	C
ATOM	286	O	PHE	19	21.138	-5.268	-0.291	1.00	0.00	O
ATOM	287	CB	PHE	19	20.919	-2.240	1.098	1.00	0.00	C
ATOM	288	CG	PHE	19	19.747	-1.308	0.982	1.00	0.00	C
ATOM	289	CD1	PHE	19	18.912	-1.356	-0.125	1.00	0.00	C
ATOM	290	CE1	PHE	19	17.835	-0.498	-0.235	1.00	0.00	C
ATOM	291	CZ	PHE	19	17.579	0.420	0.765	1.00	0.00	C
ATOM	292	CE2	PHE	19	18.401	0.476	1.873	1.00	0.00	C
ATOM	293	CD2	PHE	19	19.479	-0.383	1.977	1.00	0.00	C
ATOM	294	H	PHE	19	20.861	-3.219	3.442	1.00	0.00	H
ATOM	295	HA	PHE	19	19.523	-3.862	1.033	1.00	0.00	H
ATOM	296	2HB3	PHE	19	21.459	-2.215	0.165	1.00	0.00	H
ATOM	297	1HD	PHE	19	19.113	-2.075	-0.908	1.00	0.00	H
ATOM	298	1HE	PHE	19	17.195	-0.547	-1.103	1.00	0.00	H

ATOM	299	HZ	PHE	19	16.736	1.088	0.681	1.00	0.00	H
ATOM	300	2HE	PHE	19	18.204	1.194	2.654	1.00	0.00	H
ATOM	301	2HD	PHE	19	20.121	-0.335	2.844	1.00	0.00	H
ATOM	302	HB2	PHE	19	21.545	-1.857	1.903	1.00	0.00	H
ATOM	303	N	ASP	20	22.684	-4.768	1.264	1.00	0.00	N
ATOM	304	CA	ASP	20	23.696	-5.655	0.701	1.00	0.00	C
ATOM	305	C	ASP	20	23.301	-7.116	0.887	1.00	0.00	C
ATOM	306	O	ASP	20	23.649	-7.744	1.887	1.00	0.00	O
ATOM	307	CB	ASP	20	25.053	-5.393	1.355	1.00	0.00	C
ATOM	308	CG	ASP	20	26.103	-6.403	0.936	1.00	0.00	C
ATOM	309	OD1	ASP	20	26.170	-6.723	-0.269	1.00	0.00	O
ATOM	310	OD2	ASP	20	26.856	-6.877	1.812	1.00	0.00	O
ATOM	311	H	ASP	20	22.897	-4.248	2.067	1.00	0.00	H
ATOM	312	HA	ASP	20	23.769	-5.445	-0.354	1.00	0.00	H
ATOM	313	2HB3	ASP	20	24.943	-5.439	2.429	1.00	0.00	H
ATOM	314	HB2	ASP	20	25.415	-4.408	1.061	1.00	0.00	H
ATOM	315	N	LYS	21	22.570	-7.654	-0.084	1.00	0.00	N
ATOM	316	CA	LYS	21	22.126	-9.041	-0.029	1.00	0.00	C
ATOM	317	C	LYS	21	23.275	-9.994	-0.351	1.00	0.00	C
ATOM	318	O	LYS	21	23.421	-11.041	0.278	1.00	0.00	O
ATOM	319	CB	LYS	21	20.972	-9.268	-1.006	1.00	0.00	C
ATOM	320	CG	LYS	21	19.790	-8.339	-0.782	1.00	0.00	C
ATOM	321	CD	LYS	21	19.080	-8.649	0.525	1.00	0.00	C
ATOM	322	CE	LYS	21	17.606	-8.281	0.458	1.00	0.00	C
ATOM	323	NZ	LYS	21	16.832	-8.887	1.575	1.00	0.00	N
ATOM	324	H	LYS	21	22.323	-7.104	-0.857	1.00	0.00	H
ATOM	325	HA	LYS	21	21.781	-9.241	0.975	1.00	0.00	H
ATOM	326	2HB3	LYS	21	20.625	-10.288	-0.906	1.00	0.00	H
ATOM	327	2HG3	LYS	21	19.092	-8.455	-1.598	1.00	0.00	H
ATOM	328	2HD3	LYS	21	19.547	-8.085	1.321	1.00	0.00	H
ATOM	329	2HE3	LYS	21	17.203	-8.632	-0.481	1.00	0.00	H
ATOM	330	1HZ	LYS	21	17.472	-9.141	2.356	1.00	0.00	H
ATOM	331	2HZ	LYS	21	16.343	-9.744	1.248	1.00	0.00	H
ATOM	332	3HZ	LYS	21	16.125	-8.210	1.930	1.00	0.00	H
ATOM	333	HB2	LYS	21	21.315	-9.085	-2.024	1.00	0.00	H
ATOM	334	HG2	LYS	21	20.144	-7.309	-0.738	1.00	0.00	H
ATOM	335	HD2	LYS	21	19.150	-9.716	0.734	1.00	0.00	H
ATOM	336	HE2	LYS	21	17.497	-7.198	0.534	1.00	0.00	H
ATOM	337	N	ASP	22	24.087	-9.621	-1.333	1.00	0.00	N
ATOM	338	CA	ASP	22	25.224	-10.440	-1.737	1.00	0.00	C
ATOM	339	C	ASP	22	26.145	-10.714	-0.551	1.00	0.00	C
ATOM	340	O	ASP	22	26.622	-11.833	-0.368	1.00	0.00	O
ATOM	341	CB	ASP	22	26.004	-9.749	-2.857	1.00	0.00	C
ATOM	342	CG	ASP	22	25.097	-9.179	-3.929	1.00	0.00	C
ATOM	343	OD1	ASP	22	24.017	-9.761	-4.164	1.00	0.00	O
ATOM	344	OD2	ASP	22	25.466	-8.149	-4.533	1.00	0.00	O
ATOM	345	H	ASP	22	23.918	-8.774	-1.797	1.00	0.00	H
ATOM	346	HA	ASP	22	24.842	-11.380	-2.105	1.00	0.00	H
ATOM	347	2HB3	ASP	22	26.670	-10.464	-3.317	1.00	0.00	H
ATOM	348	HB2	ASP	22	26.578	-8.919	-2.442	1.00	0.00	H
ATOM	349	N	GLY	23	26.391	-9.682	0.250	1.00	0.00	N
ATOM	350	CA	GLY	23	27.255	-9.831	1.406	1.00	0.00	C
ATOM	351	C	GLY	23	28.709	-9.543	1.085	1.00	0.00	C
ATOM	352	O	GLY	23	29.596	-10.314	1.454	1.00	0.00	O
ATOM	353	H	GLY	23	25.984	-8.812	0.056	1.00	0.00	H
ATOM	354	2HA	GLY	23	26.927	-9.151	2.178	1.00	0.00	H
ATOM	355	3HA	GLY	23	27.173	-10.843	1.774	1.00	0.00	H
ATOM	356	N	ASP	24	28.953	-8.435	0.396	1.00	0.00	N
ATOM	357	CA	ASP	24	30.307	-8.050	0.023	1.00	0.00	C
ATOM	358	C	ASP	24	30.632	-6.647	0.529	1.00	0.00	C
ATOM	359	O	ASP	24	31.698	-6.410	1.093	1.00	0.00	O
ATOM	360	CB	ASP	24	30.481	-8.109	-1.494	1.00	0.00	C
ATOM	361	CG	ASP	24	29.479	-7.240	-2.226	1.00	0.00	C
ATOM	362	OD1	ASP	24	28.271	-7.352	-1.932	1.00	0.00	O
ATOM	363	OD2	ASP	24	29.902	-6.447	-3.094	1.00	0.00	O
ATOM	364	H	ASP	24	28.202	-7.865	0.129	1.00	0.00	H
ATOM	365	HA	ASP	24	30.991	-8.750	0.483	1.00	0.00	H
ATOM	366	2HB3	ASP	24	30.353	-9.129	-1.825	1.00	0.00	H
ATOM	367	HB2	ASP	24	31.477	-7.756	-1.761	1.00	0.00	H
ATOM	368	N	GLY	25	29.703	-5.720	0.323	1.00	0.00	N
ATOM	369	CA	GLY	25	29.907	-4.352	0.763	1.00	0.00	C
ATOM	370	C	GLY	25	29.521	-3.338	-0.296	1.00	0.00	C
ATOM	371	O	GLY	25	30.077	-2.241	-0.348	1.00	0.00	O
ATOM	372	H	GLY	25	28.870	-5.967	-0.132	1.00	0.00	H
ATOM	373	2HA	GLY	25	29.314	-4.176	1.647	1.00	0.00	H
ATOM	374	3HA	GLY	25	30.951	-4.218	1.010	1.00	0.00	H
ATOM	375	N	THR	26	28.565	-3.706	-1.143	1.00	0.00	N

ATOM	376	CA	THR	26	28.107	-2.822	-2.209	1.00	0.00	C
ATOM	377	C	THR	26	26.611	-2.980	-2.448	1.00	0.00	C
ATOM	378	O	THR	26	25.979	-3.892	-1.912	1.00	0.00	O
ATOM	379	CB	THR	26	28.857	-3.093	-3.526	1.00	0.00	C
ATOM	380	OG1	THR	26	28.628	-4.441	-3.951	1.00	0.00	O
ATOM	381	CG2	THR	26	30.349	-2.854	-3.356	1.00	0.00	C
ATOM	382	H	THR	26	28.160	-4.593	-1.051	1.00	0.00	H
ATOM	383	HA	THR	26	28.308	-1.803	-1.907	1.00	0.00	H
ATOM	384	HB	THR	26	28.483	-2.417	-4.282	1.00	0.00	H
ATOM	385	1HG	THR	26	29.228	-4.655	-4.670	1.00	0.00	H
ATOM	386	1HG2	THR	26	30.893	-3.469	-4.059	1.00	0.00	H
ATOM	387	2HG2	THR	26	30.644	-3.112	-2.350	1.00	0.00	H
ATOM	388	3HG2	THR	26	30.570	-1.814	-3.542	1.00	0.00	H
ATOM	389	N	ILE	27	26.047	-2.087	-3.255	1.00	0.00	N
ATOM	390	CA	ILE	27	24.623	-2.129	-3.566	1.00	0.00	C
ATOM	391	C	ILE	27	24.372	-1.823	-5.040	1.00	0.00	C
ATOM	392	O	ILE	27	24.961	-0.898	-5.600	1.00	0.00	O
ATOM	393	CB	ILE	27	23.828	-1.133	-2.704	1.00	0.00	C
ATOM	394	CG2	ILE	27	22.338	-1.261	-2.982	1.00	0.00	C
ATOM	395	CG1	ILE	27	24.120	-1.363	-1.220	1.00	0.00	C
ATOM	396	CD1	ILE	27	23.853	-0.150	-0.355	1.00	0.00	C
ATOM	397	H	ILE	27	26.601	-1.384	-3.652	1.00	0.00	H
ATOM	398	HA	ILE	27	24.266	-3.127	-3.352	1.00	0.00	H
ATOM	399	HB	ILE	27	24.135	-0.133	-2.972	1.00	0.00	H
ATOM	400	1HG2	ILE	27	21.849	-1.712	-2.132	1.00	0.00	H
ATOM	401	2HG2	ILE	27	21.919	-0.281	-3.159	1.00	0.00	H
ATOM	402	3HG2	ILE	27	22.186	-1.880	-3.853	1.00	0.00	H
ATOM	403	2HG3	ILE	27	25.158	-1.633	-1.102	1.00	0.00	H
ATOM	404	1HD1	ILE	27	24.448	0.680	-0.708	1.00	0.00	H
ATOM	405	2HD1	ILE	27	22.807	0.107	-0.406	1.00	0.00	H
ATOM	406	3HD1	ILE	27	24.119	-0.372	0.669	1.00	0.00	H
ATOM	407	HG2	ILE	27	23.481	-2.163	-0.843	1.00	0.00	H
ATOM	408	N	THR	28	23.493	-2.604	-5.659	1.00	0.00	N
ATOM	409	CA	THR	28	23.164	-2.415	-7.067	1.00	0.00	C
ATOM	410	C	THR	28	21.658	-2.474	-7.291	1.00	0.00	C
ATOM	411	O	THR	28	20.890	-2.735	-6.363	1.00	0.00	O
ATOM	412	CB	THR	28	23.846	-3.478	-7.950	1.00	0.00	C
ATOM	413	OG1	THR	28	23.587	-3.204	-9.331	1.00	0.00	O
ATOM	414	CG2	THR	28	23.347	-4.872	-7.602	1.00	0.00	C
ATOM	415	H	THR	28	23.057	-3.325	-5.159	1.00	0.00	H
ATOM	416	HA	THR	28	23.525	-1.442	-7.367	1.00	0.00	H
ATOM	417	HB	THR	28	24.911	-3.438	-7.776	1.00	0.00	H
ATOM	418	1HG	THR	28	23.945	-3.913	-9.873	1.00	0.00	H
ATOM	419	1HG2	THR	28	23.880	-5.239	-6.737	1.00	0.00	H
ATOM	420	2HG2	THR	28	23.515	-5.533	-8.438	1.00	0.00	H
ATOM	421	3HG2	THR	28	22.291	-4.831	-7.383	1.00	0.00	H
ATOM	422	N	THR	29	21.238	-2.229	-8.529	1.00	0.00	N
ATOM	423	CA	THR	29	19.824	-2.254	-8.877	1.00	0.00	C
ATOM	424	C	THR	29	19.148	-3.505	-8.333	1.00	0.00	C
ATOM	425	O	THR	29	17.995	-3.463	-7.894	1.00	0.00	O
ATOM	426	CB	THR	29	19.617	-2.192	-10.402	1.00	0.00	C
ATOM	427	OG1	THR	29	20.093	-3.398	-11.009	1.00	0.00	O
ATOM	428	CG2	THR	29	20.343	-0.996	-10.997	1.00	0.00	C
ATOM	429	H	THR	29	21.899	-2.026	-9.226	1.00	0.00	H
ATOM	430	HA	THR	29	19.358	-1.384	-8.435	1.00	0.00	H
ATOM	431	HB	THR	29	18.560	-2.090	-10.602	1.00	0.00	H
ATOM	432	1HG	THR	29	19.952	-4.136	-10.411	1.00	0.00	H
ATOM	433	1HG2	THR	29	20.120	-0.928	-12.051	1.00	0.00	H
ATOM	434	2HG2	THR	29	21.408	-1.119	-10.864	1.00	0.00	H
ATOM	435	3HG2	THR	29	20.020	-0.094	-10.501	1.00	0.00	H
ATOM	436	N	LYS	30	19.867	-4.622	-8.364	1.00	0.00	N
ATOM	437	CA	LYS	30	19.336	-5.890	-7.874	1.00	0.00	C
ATOM	438	C	LYS	30	19.015	-5.804	-6.384	1.00	0.00	C
ATOM	439	O	LYS	30	17.949	-6.236	-5.945	1.00	0.00	O
ATOM	440	CB	LYS	30	20.339	-7.018	-8.125	1.00	0.00	C
ATOM	441	CG	LYS	30	19.927	-8.346	-7.512	1.00	0.00	C
ATOM	442	CD	LYS	30	18.900	-9.062	-8.371	1.00	0.00	C
ATOM	443	CE	LYS	30	19.561	-9.853	-9.489	1.00	0.00	C
ATOM	444	NZ	LYS	30	18.577	-10.290	-10.517	1.00	0.00	N
ATOM	445	H	LYS	30	20.778	-4.594	-8.725	1.00	0.00	H
ATOM	446	HA	LYS	30	18.427	-6.101	-8.414	1.00	0.00	H
ATOM	447	2HB3	LYS	30	21.294	-6.733	-7.707	1.00	0.00	H
ATOM	448	2HG3	LYS	30	19.504	-8.165	-6.534	1.00	0.00	H
ATOM	449	2HD3	LYS	30	18.234	-8.329	-8.805	1.00	0.00	H
ATOM	450	2HE3	LYS	30	20.037	-10.724	-9.064	1.00	0.00	H
ATOM	451	1HZ	LYS	30	18.991	-10.206	-11.468	1.00	0.00	H
ATOM	452	2HZ	LYS	30	17.723	-9.701	-10.468	1.00	0.00	H

ATOM	453	3HZ	LYS	30	18.307	-11.282	-10.356	1.00	0.00	H
ATOM	454	HB2	LYS	30	20.430	-7.191	-9.197	1.00	0.00	H
ATOM	455	HG2	LYS	30	20.801	-8.992	-7.425	1.00	0.00	H
ATOM	456	HD2	LYS	30	18.334	-9.759	-7.755	1.00	0.00	H
ATOM	457	HE2	LYS	30	20.300	-9.227	-9.990	1.00	0.00	H
ATOM	458	N	GLU	31	19.941	-5.242	-5.615	1.00	0.00	N
ATOM	459	CA	GLU	31	19.754	-5.101	-4.175	1.00	0.00	C
ATOM	460	C	GLU	31	18.846	-3.916	-3.858	1.00	0.00	C
ATOM	461	O	GLU	31	17.752	-4.084	-3.316	1.00	0.00	O
ATOM	462	CB	GLU	31	21.104	-4.922	-3.478	1.00	0.00	C
ATOM	463	CG	GLU	31	21.995	-6.150	-3.555	1.00	0.00	C
ATOM	464	CD	GLU	31	23.471	-5.800	-3.516	1.00	0.00	C
ATOM	465	OE1	GLU	31	23.997	-5.329	-4.546	1.00	0.00	O
ATOM	466	OE2	GLU	31	24.101	-6.003	-2.457	1.00	0.00	O
ATOM	467	H	GLU	31	20.770	-4.915	-6.023	1.00	0.00	H
ATOM	468	HA	GLU	31	19.287	-6.003	-3.810	1.00	0.00	H
ATOM	469	2HB3	GLU	31	20.931	-4.694	-2.437	1.00	0.00	H
ATOM	470	2HG3	GLU	31	21.788	-6.672	-4.479	1.00	0.00	H
ATOM	471	HB2	GLU	31	21.654	-4.111	-3.956	1.00	0.00	H
ATOM	472	HG2	GLU	31	21.795	-6.801	-2.702	1.00	0.00	H
ATOM	473	N	LEU	32	19.304	-2.717	-4.201	1.00	0.00	N
ATOM	474	CA	LEU	32	18.533	-1.504	-3.954	1.00	0.00	C
ATOM	475	C	LEU	32	17.111	-1.645	-4.486	1.00	0.00	C
ATOM	476	O	LEU	32	16.154	-1.200	-3.852	1.00	0.00	O
ATOM	477	CB	LEU	32	19.217	-0.302	-4.608	1.00	0.00	C
ATOM	478	CG	LEU	32	18.479	1.033	-4.494	1.00	0.00	C
ATOM	479	CD1	LEU	32	18.397	1.476	-3.043	1.00	0.00	C
ATOM	480	CD2	LEU	32	19.165	2.098	-5.340	1.00	0.00	C
ATOM	481	H	LEU	32	20.181	-2.647	-4.632	1.00	0.00	H
ATOM	482	HA	LEU	32	18.493	-1.349	-2.887	1.00	0.00	H
ATOM	483	2HB3	LEU	32	19.341	-0.523	-5.660	1.00	0.00	H
ATOM	484	HG	LEU	32	17.470	0.911	-4.863	1.00	0.00	H
ATOM	485	1HD1	LEU	32	18.406	0.608	-2.401	1.00	0.00	H
ATOM	486	2HD1	LEU	32	17.484	2.032	-2.885	1.00	0.00	H
ATOM	487	3HD1	LEU	32	19.245	2.104	-2.808	1.00	0.00	H
ATOM	488	1HD2	LEU	32	19.995	2.516	-4.787	1.00	0.00	H
ATOM	489	2HD2	LEU	32	18.460	2.879	-5.576	1.00	0.00	H
ATOM	490	3HD2	LEU	32	19.529	1.650	-6.252	1.00	0.00	H
ATOM	491	HB2	LEU	32	20.180	-0.124	-4.129	1.00	0.00	H
ATOM	492	N	GLY	33	16.977	-2.271	-5.651	1.00	0.00	N
ATOM	493	CA	GLY	33	15.667	-2.459	-6.246	1.00	0.00	C
ATOM	494	C	GLY	33	14.835	-3.481	-5.497	1.00	0.00	C
ATOM	495	O	GLY	33	13.612	-3.523	-5.642	1.00	0.00	O
ATOM	496	H	GLY	33	17.775	-2.603	-6.112	1.00	0.00	H
ATOM	497	2HA	GLY	33	15.144	-1.514	-6.247	1.00	0.00	H
ATOM	498	3HA	GLY	33	15.792	-2.792	-7.266	1.00	0.00	H
ATOM	499	N	THR	34	15.496	-4.309	-4.698	1.00	0.00	N
ATOM	500	CA	THR	34	14.810	-5.341	-3.925	1.00	0.00	C
ATOM	501	C	THR	34	14.196	-4.758	-2.658	1.00	0.00	C
ATOM	502	O	THR	34	13.209	-5.280	-2.139	1.00	0.00	O
ATOM	503	CB	THR	34	15.765	-6.486	-3.542	1.00	0.00	C
ATOM	504	OG1	THR	34	15.062	-7.733	-3.542	1.00	0.00	O
ATOM	505	CG2	THR	34	16.377	-6.243	-2.170	1.00	0.00	C
ATOM	506	H	THR	34	16.471	-4.228	-4.624	1.00	0.00	H
ATOM	507	HA	THR	34	14.023	-5.749	-4.542	1.00	0.00	H
ATOM	508	HB	THR	34	16.562	-6.530	-4.272	1.00	0.00	H
ATOM	509	1HG	THR	34	14.674	-7.882	-2.676	1.00	0.00	H
ATOM	510	1HG2	THR	34	17.373	-6.656	-2.142	1.00	0.00	H
ATOM	511	2HG2	THR	34	15.769	-6.719	-1.416	1.00	0.00	H
ATOM	512	3HG2	THR	34	16.423	-5.181	-1.978	1.00	0.00	H
ATOM	513	N	VAL	35	14.786	-3.675	-2.164	1.00	0.00	N
ATOM	514	CA	VAL	35	14.295	-3.020	-0.955	1.00	0.00	C
ATOM	515	C	VAL	35	13.442	-1.804	-1.297	1.00	0.00	C
ATOM	516	O	VAL	35	12.455	-1.517	-0.621	1.00	0.00	O
ATOM	517	CB	VAL	35	15.457	-2.580	-0.045	1.00	0.00	C
ATOM	518	CG1	VAL	35	15.049	-1.379	0.796	1.00	0.00	C
ATOM	519	CG2	VAL	35	15.905	-3.734	0.842	1.00	0.00	C
ATOM	520	H	VAL	35	15.569	-3.306	-2.622	1.00	0.00	H
ATOM	521	HA	VAL	35	13.691	-3.734	-0.413	1.00	0.00	H
ATOM	522	HB	VAL	35	16.288	-2.290	-0.670	1.00	0.00	H
ATOM	523	1HG1	VAL	35	14.020	-1.491	1.106	1.00	0.00	H
ATOM	524	2HG1	VAL	35	15.685	-1.317	1.667	1.00	0.00	H
ATOM	525	3HG1	VAL	35	15.152	-0.479	0.207	1.00	0.00	H
ATOM	526	1HG2	VAL	35	15.042	-4.192	1.298	1.00	0.00	H
ATOM	527	2HG2	VAL	35	16.427	-4.467	0.243	1.00	0.00	H
ATOM	528	3HG2	VAL	35	16.567	-3.362	1.609	1.00	0.00	H
ATOM	529	N	MET	36	13.828	-1.095	-2.351	1.00	0.00	N

ATOM	530	CA	MET	36	13.096	0.092	-2.784	1.00	0.00	C
ATOM	531	C	MET	36	11.654	-0.260	-3.136	1.00	0.00	C
ATOM	532	O	MET	36	10.728	0.482	-2.807	1.00	0.00	O
ATOM	533	CB	MET	36	13.787	0.729	-3.990	1.00	0.00	C
ATOM	534	CG	MET	36	14.985	1.589	-3.622	1.00	0.00	C
ATOM	535	SD	MET	36	15.252	2.942	-4.782	1.00	0.00	S
ATOM	536	CE	MET	36	13.727	3.860	-4.589	1.00	0.00	C
ATOM	537	H	MET	36	14.624	-1.374	-2.852	1.00	0.00	H
ATOM	538	HA	MET	36	13.096	0.795	-1.967	1.00	0.00	H
ATOM	539	2HB3	MET	36	13.073	1.350	-4.512	1.00	0.00	H
ATOM	540	2HG3	MET	36	15.866	0.966	-3.607	1.00	0.00	H
ATOM	541	1HE	MET	36	12.990	3.489	-5.286	1.00	0.00	H
ATOM	542	2HE	MET	36	13.359	3.741	-3.579	1.00	0.00	H
ATOM	543	3HE	MET	36	13.910	4.908	-4.783	1.00	0.00	H
ATOM	544	HB2	MET	36	14.163	-0.053	-4.651	1.00	0.00	H
ATOM	545	HG2	MET	36	14.821	2.038	-2.641	1.00	0.00	H
ATOM	546	N	ARG	37	11.471	-1.393	-3.805	1.00	0.00	N
ATOM	547	CA	ARG	37	10.140	-1.839	-4.202	1.00	0.00	C
ATOM	548	C	ARG	37	9.343	-2.316	-2.989	1.00	0.00	C
ATOM	549	O	ARG	37	8.118	-2.414	-3.042	1.00	0.00	O
ATOM	550	CB	ARG	37	10.243	-2.963	-5.232	1.00	0.00	C
ATOM	551	CG	ARG	37	11.073	-4.148	-4.762	1.00	0.00	C
ATOM	552	CD	ARG	37	10.247	-5.105	-3.917	1.00	0.00	C
ATOM	553	NE	ARG	37	10.710	-6.486	-4.048	1.00	0.00	N
ATOM	554	CZ	ARG	37	9.928	-7.543	-3.850	1.00	0.00	C
ATOM	555	NH1	ARG	37	8.656	-7.379	-3.515	1.00	0.00	N
ATOM	556	NH2	ARG	37	10.422	-8.768	-3.986	1.00	0.00	N
ATOM	557	H	ARG	37	12.249	-1.942	-4.038	1.00	0.00	H
ATOM	558	HA	ARG	37	9.628	-1.000	-4.647	1.00	0.00	H
ATOM	559	2HB3	ARG	37	10.692	-2.572	-6.131	1.00	0.00	H
ATOM	560	2HG3	ARG	37	11.900	-3.783	-4.173	1.00	0.00	H
ATOM	561	2HD3	ARG	37	9.218	-5.048	-4.236	1.00	0.00	H
ATOM	562	HE	ARG	37	11.646	-6.631	-4.293	1.00	0.00	H
ATOM	563	1HH1	ARG	37	8.283	-6.458	-3.413	1.00	0.00	H
ATOM	564	2HH1	ARG	37	8.071	-8.178	-3.367	1.00	0.00	H
ATOM	565	1HH2	ARG	37	11.380	-8.896	-4.239	1.00	0.00	H
ATOM	566	2HH2	ARG	37	9.834	-9.563	-3.838	1.00	0.00	H
ATOM	567	HB2	ARG	37	9.247	-3.356	-5.445	1.00	0.00	H
ATOM	568	HG2	ARG	37	11.444	-4.696	-5.626	1.00	0.00	H
ATOM	569	HD2	ARG	37	10.332	-4.829	-2.865	1.00	0.00	H
ATOM	570	N	SER	38	10.048	-2.610	-1.903	1.00	0.00	N
ATOM	571	CA	SER	38	9.408	-3.080	-0.679	1.00	0.00	C
ATOM	572	C	SER	38	8.544	-1.985	-0.064	1.00	0.00	C
ATOM	573	O	SER	38	7.521	-2.264	0.564	1.00	0.00	O
ATOM	574	CB	SER	38	10.462	-3.541	0.328	1.00	0.00	C
ATOM	575	OG	SER	38	11.314	-4.523	-0.235	1.00	0.00	O
ATOM	576	H	SER	38	11.023	-2.512	-1.925	1.00	0.00	H
ATOM	577	HA	SER	38	8.779	-3.917	-0.937	1.00	0.00	H
ATOM	578	2HB3	SER	38	9.969	-3.962	1.193	1.00	0.00	H
ATOM	579	HG	SER	38	10.819	-5.049	-0.867	1.00	0.00	H
ATOM	580	HB2	SER	38	11.071	-2.690	0.632	1.00	0.00	H
ATOM	581	N	LEU	39	8.958	-0.737	-0.250	1.00	0.00	N
ATOM	582	CA	LEU	39	8.221	0.402	0.285	1.00	0.00	C
ATOM	583	C	LEU	39	7.090	0.811	-0.652	1.00	0.00	C
ATOM	584	O	LEU	39	5.994	1.155	-0.209	1.00	0.00	O
ATOM	585	CB	LEU	39	9.166	1.583	0.511	1.00	0.00	C
ATOM	586	CG	LEU	39	10.123	1.465	1.698	1.00	0.00	C
ATOM	587	CD1	LEU	39	11.130	2.603	1.687	1.00	0.00	C
ATOM	588	CD2	LEU	39	9.347	1.449	3.006	1.00	0.00	C
ATOM	589	H	LEU	39	9.779	-0.579	-0.761	1.00	0.00	H
ATOM	590	HA	LEU	39	7.799	0.105	1.232	1.00	0.00	H
ATOM	591	2HB3	LEU	39	8.558	2.468	0.661	1.00	0.00	H
ATOM	592	HG	LEU	39	10.668	0.534	1.619	1.00	0.00	H
ATOM	593	1HD1	LEU	39	11.492	2.773	2.689	1.00	0.00	H
ATOM	594	2HD1	LEU	39	10.657	3.503	1.318	1.00	0.00	H
ATOM	595	3HD1	LEU	39	11.958	2.345	1.043	1.00	0.00	H
ATOM	596	1HD2	LEU	39	8.288	1.412	2.797	1.00	0.00	H
ATOM	597	2HD2	LEU	39	9.572	2.342	3.569	1.00	0.00	H
ATOM	598	3HD2	LEU	39	9.630	0.579	3.582	1.00	0.00	H
ATOM	599	HB2	LEU	39	9.820	1.696	-0.354	1.00	0.00	H
ATOM	600	N	GLY	40	7.364	0.767	-1.953	1.00	0.00	N
ATOM	601	CA	GLY	40	6.357	1.131	-2.933	1.00	0.00	C
ATOM	602	C	GLY	40	6.885	2.103	-3.971	1.00	0.00	C
ATOM	603	O	GLY	40	6.161	2.993	-4.420	1.00	0.00	O
ATOM	604	H	GLY	40	8.253	0.483	-2.249	1.00	0.00	H
ATOM	605	2HA	GLY	40	6.016	0.238	-3.436	1.00	0.00	H
ATOM	606	3HA	GLY	40	5.521	1.585	-2.423	1.00	0.00	H

```
ATOM    607  N   GLN    41       8.147   1.935  -4.348  1.00  0.00           N
ATOM    608  CA  GLN    41       8.771   2.809  -5.337  1.00  0.00           C
ATOM    609  C   GLN    41       8.802   2.143  -6.709  1.00  0.00           C
ATOM    610  O   GLN    41       8.541   2.784  -7.724  1.00  0.00           O
ATOM    611  CB  GLN    41      10.192   3.172  -4.901  1.00  0.00           C
ATOM    612  CG  GLN    41      10.240   4.114  -3.709  1.00  0.00           C
ATOM    613  CD  GLN    41       9.295   5.290  -3.855  1.00  0.00           C
ATOM    614  OE1 GLN    41       9.035   5.759  -4.964  1.00  0.00           O
ATOM    615  NE2 GLN    41       8.773   5.774  -2.735  1.00  0.00           N
ATOM    616  H   GLN    41       8.670   1.210  -3.952  1.00  0.00           H
ATOM    617  HA  GLN    41       8.181   3.710  -5.400  1.00  0.00           H
ATOM    618 2HB3 GLN    41      10.697   3.648  -5.731  1.00  0.00           H
ATOM    619 2HG3 GLN    41      11.248   4.491  -3.607  1.00  0.00           H
ATOM    620 1HE2 GLN    41       9.025   5.349  -1.888  1.00  0.00           H
ATOM    621 2HE2 GLN    41       8.158   6.534  -2.800  1.00  0.00           H
ATOM    622  HB2 GLN    41      10.724   2.269  -4.602  1.00  0.00           H
ATOM    623  HG2 GLN    41       9.944   3.577  -2.809  1.00  0.00           H
ATOM    624  N   ASN    42       9.119   0.852  -6.728  1.00  0.00           N
ATOM    625  CA  ASN    42       9.184   0.101  -7.975  1.00  0.00           C
ATOM    626  C   ASN    42       9.851   0.924  -9.073  1.00  0.00           C
ATOM    627  O   ASN    42       9.322   1.081 -10.174  1.00  0.00           O
ATOM    628  CB  ASN    42       7.780  -0.317  -8.418  1.00  0.00           C
ATOM    629  CG  ASN    42       7.363  -1.652  -7.834  1.00  0.00           C
ATOM    630  OD1 ASN    42       7.903  -2.696  -8.197  1.00  0.00           O
ATOM    631  ND2 ASN    42       6.396  -1.625  -6.925  1.00  0.00           N
ATOM    632  H   ASN    42       9.315   0.396  -5.884  1.00  0.00           H
ATOM    633  HA  ASN    42       9.774  -0.787  -7.799  1.00  0.00           H
ATOM    634 2HB3 ASN    42       7.754  -0.391  -9.494  1.00  0.00           H
ATOM    635 1HD2 ASN    42       6.010  -0.757  -6.684  1.00  0.00           H
ATOM    636 2HD2 ASN    42       6.107  -2.475  -6.529  1.00  0.00           H
ATOM    637  HB2 ASN    42       7.056   0.425  -8.078  1.00  0.00           H
ATOM    638  N   PRO    43      11.040   1.462  -8.769  1.00  0.00           N
ATOM    639  CA  PRO    43      11.807   2.277  -9.715  1.00  0.00           C
ATOM    640  C   PRO    43      12.367   1.454 -10.871  1.00  0.00           C
ATOM    641  O   PRO    43      12.820   0.324 -10.678  1.00  0.00           O
ATOM    642  CD  PRO    43      11.730   1.316  -7.476  1.00  0.00           C
ATOM    643  CB  PRO    43      12.944   2.842  -8.859  1.00  0.00           C
ATOM    644  CG  PRO    43      13.105   1.859  -7.750  1.00  0.00           C
ATOM    645  HA  PRO    43      11.216   3.090 -10.110  1.00  0.00           H
ATOM    646 2HB3 PRO    43      12.671   3.815  -8.487  1.00  0.00           H
ATOM    647 2HG3 PRO    43      13.494   2.357  -6.874  1.00  0.00           H
ATOM    648 2HD3 PRO    43      11.241   1.901  -6.709  1.00  0.00           H
ATOM    649  HD2 PRO    43      11.774   0.262  -7.203  1.00  0.00           H
ATOM    650  HB2 PRO    43      13.846   2.932  -9.467  1.00  0.00           H
ATOM    651  HG2 PRO    43      13.762   1.045  -8.059  1.00  0.00           H
ATOM    652  N   THR    44      12.330   2.023 -12.070  1.00  0.00           N
ATOM    653  CA  THR    44      12.833   1.341 -13.256  1.00  0.00           C
ATOM    654  C   THR    44      14.356   1.376 -13.307  1.00  0.00           C
ATOM    655  O   THR    44      14.991   2.176 -12.619  1.00  0.00           O
ATOM    656  CB  THR    44      12.273   1.968 -14.546  1.00  0.00           C
ATOM    657  OG1 THR    44      12.623   3.355 -14.607  1.00  0.00           O
ATOM    658  CG2 THR    44      10.759   1.819 -14.607  1.00  0.00           C
ATOM    659  H   THR    44      11.955   2.924 -12.159  1.00  0.00           H
ATOM    660  HA  THR    44      12.508   0.312 -13.211  1.00  0.00           H
ATOM    661  HB  THR    44      12.705   1.458 -15.394  1.00  0.00           H
ATOM    662 1HG  THR    44      12.769   3.608 -15.522  1.00  0.00           H
ATOM    663 1HG2 THR    44      10.407   2.136 -15.576  1.00  0.00           H
ATOM    664 2HG2 THR    44      10.306   2.429 -13.840  1.00  0.00           H
ATOM    665 3HG2 THR    44      10.494   0.784 -14.449  1.00  0.00           H
ATOM    666  N   GLU    45      14.938   0.506 -14.126  1.00  0.00           N
ATOM    667  CA  GLU    45      16.388   0.440 -14.266  1.00  0.00           C
ATOM    668  C   GLU    45      16.991   1.839 -14.341  1.00  0.00           C
ATOM    669  O   GLU    45      17.847   2.201 -13.535  1.00  0.00           O
ATOM    670  CB  GLU    45      16.765  -0.358 -15.516  1.00  0.00           C
ATOM    671  CG  GLU    45      16.939  -1.845 -15.258  1.00  0.00           C
ATOM    672  CD  GLU    45      17.628  -2.561 -16.405  1.00  0.00           C
ATOM    673  OE1 GLU    45      17.044  -2.615 -17.507  1.00  0.00           O
ATOM    674  OE2 GLU    45      18.752  -3.064 -16.197  1.00  0.00           O
ATOM    675  H   GLU    45      14.378  -0.105 -14.648  1.00  0.00           H
ATOM    676  HA  GLU    45      16.782  -0.062 -13.396  1.00  0.00           H
ATOM    677 2HB3 GLU    45      17.694   0.028 -15.906  1.00  0.00           H
ATOM    678 2HG3 GLU    45      15.964  -2.289 -15.111  1.00  0.00           H
ATOM    679  HB2 GLU    45      15.974  -0.267 -16.259  1.00  0.00           H
ATOM    680  HG2 GLU    45      17.557  -1.991 -14.372  1.00  0.00           H
ATOM    681  N   ALA    46      16.538   2.620 -15.316  1.00  0.00           N
ATOM    682  CA  ALA    46      17.032   3.981 -15.497  1.00  0.00           C
ATOM    683  C   ALA    46      17.019   4.747 -14.179  1.00  0.00           C
```

ATOM	684	O	ALA	46	17.933	5.523	-13.894	1.00	0.00	O
ATOM	685	CB	ALA	46	16.202	4.709	-16.542	1.00	0.00	C
ATOM	686	H	ALA	46	15.855	2.275	-15.928	1.00	0.00	H
ATOM	687	HA	ALA	46	18.050	3.922	-15.857	1.00	0.00	H
ATOM	688	1HB	ALA	46	16.690	4.636	-17.503	1.00	0.00	H
ATOM	689	2HB	ALA	46	15.222	4.260	-16.600	1.00	0.00	H
ATOM	690	3HB	ALA	46	16.107	5.749	-16.265	1.00	0.00	H
ATOM	691	N	GLU	47	15.980	4.528	-13.380	1.00	0.00	N
ATOM	692	CA	GLU	47	15.849	5.202	-12.094	1.00	0.00	C
ATOM	693	C	GLU	47	16.936	4.743	-11.127	1.00	0.00	C
ATOM	694	O	GLU	47	17.672	5.558	-10.569	1.00	0.00	O
ATOM	695	CB	GLU	47	14.468	4.934	-11.492	1.00	0.00	C
ATOM	696	CG	GLU	47	13.320	5.434	-12.352	1.00	0.00	C
ATOM	697	CD	GLU	47	13.069	6.922	-12.182	1.00	0.00	C
ATOM	698	OE1	GLU	47	13.808	7.722	-12.793	1.00	0.00	O
ATOM	699	OE2	GLU	47	12.134	7.283	-11.438	1.00	0.00	O
ATOM	700	H	GLU	47	15.284	3.899	-13.663	1.00	0.00	H
ATOM	701	HA	GLU	47	15.959	6.262	-12.262	1.00	0.00	H
ATOM	702	2HB3	GLU	47	14.408	5.421	-10.529	1.00	0.00	H
ATOM	703	2HG3	GLU	47	12.422	4.900	-12.082	1.00	0.00	H
ATOM	704	HB2	GLU	47	14.323	3.858	-11.381	1.00	0.00	H
ATOM	705	HG2	GLU	47	13.555	5.268	-13.405	1.00	0.00	H
ATOM	706	N	LEU	48	17.030	3.431	-10.931	1.00	0.00	N
ATOM	707	CA	LEU	48	18.025	2.862	-10.032	1.00	0.00	C
ATOM	708	C	LEU	48	19.434	3.287	-10.436	1.00	0.00	C
ATOM	709	O	LEU	48	20.229	3.712	-9.600	1.00	0.00	O
ATOM	710	CB	LEU	48	17.923	1.336	-10.024	1.00	0.00	C
ATOM	711	CG	LEU	48	16.611	0.753	-9.500	1.00	0.00	C
ATOM	712	CD1	LEU	48	16.362	-0.625	-10.093	1.00	0.00	C
ATOM	713	CD2	LEU	48	16.627	0.687	-7.981	1.00	0.00	C
ATOM	714	H	LEU	48	16.416	2.831	-11.404	1.00	0.00	H
ATOM	715	HA	LEU	48	17.824	3.233	-9.036	1.00	0.00	H
ATOM	716	2HB3	LEU	48	18.725	0.953	-9.408	1.00	0.00	H
ATOM	717	HG	LEU	48	15.793	1.397	-9.799	1.00	0.00	H
ATOM	718	1HD1	LEU	48	16.732	-1.379	-9.416	1.00	0.00	H
ATOM	719	2HD1	LEU	48	16.877	-0.707	-11.040	1.00	0.00	H
ATOM	720	3HD1	LEU	48	15.303	-0.766	-10.247	1.00	0.00	H
ATOM	721	1HD2	LEU	48	17.470	1.247	-7.605	1.00	0.00	H
ATOM	722	2HD2	LEU	48	16.711	-0.344	-7.664	1.00	0.00	H
ATOM	723	3HD2	LEU	48	15.711	1.107	-7.592	1.00	0.00	H
ATOM	724	HB2	LEU	48	18.000	0.962	-11.045	1.00	0.00	H
ATOM	725	N	GLN	49	19.732	3.170	-11.726	1.00	0.00	N
ATOM	726	CA	GLN	49	21.044	3.544	-12.243	1.00	0.00	C
ATOM	727	C	GLN	49	21.360	5.001	-11.924	1.00	0.00	C
ATOM	728	O	GLN	49	22.462	5.324	-11.482	1.00	0.00	O
ATOM	729	CB	GLN	49	21.104	3.317	-13.755	1.00	0.00	C
ATOM	730	CG	GLN	49	21.306	1.861	-14.145	1.00	0.00	C
ATOM	731	CD	GLN	49	22.772	1.494	-14.279	1.00	0.00	C
ATOM	732	OE1	GLN	49	23.277	1.303	-15.387	1.00	0.00	O
ATOM	733	NE2	GLN	49	23.463	1.390	-13.150	1.00	0.00	N
ATOM	734	H	GLN	49	19.056	2.824	-12.344	1.00	0.00	H
ATOM	735	HA	GLN	49	21.780	2.916	-11.765	1.00	0.00	H
ATOM	736	2HB3	GLN	49	21.920	3.894	-14.162	1.00	0.00	H
ATOM	737	2HG3	GLN	49	20.818	1.683	-15.089	1.00	0.00	H
ATOM	738	1HE2	GLN	49	22.995	1.554	-12.304	1.00	0.00	H
ATOM	739	2HE2	GLN	49	24.410	1.153	-13.208	1.00	0.00	H
ATOM	740	HB2	GLN	49	20.162	3.624	-14.207	1.00	0.00	H
ATOM	741	HG2	GLN	49	20.881	1.216	-13.374	1.00	0.00	H
ATOM	742	N	ASP	50	20.386	5.876	-12.150	1.00	0.00	N
ATOM	743	CA	ASP	50	20.559	7.299	-11.885	1.00	0.00	C
ATOM	744	C	ASP	50	20.850	7.545	-10.407	1.00	0.00	C
ATOM	745	O	ASP	50	21.698	8.367	-10.059	1.00	0.00	O
ATOM	746	CB	ASP	50	19.313	8.076	-12.308	1.00	0.00	C
ATOM	747	CG	ASP	50	19.359	8.494	-13.764	1.00	0.00	C
ATOM	748	OD1	ASP	50	20.329	9.177	-14.155	1.00	0.00	O
ATOM	749	OD2	ASP	50	18.424	8.140	-14.514	1.00	0.00	O
ATOM	750	H	ASP	50	19.529	5.557	-12.503	1.00	0.00	H
ATOM	751	HA	ASP	50	21.402	7.644	-12.466	1.00	0.00	H
ATOM	752	2HB3	ASP	50	19.224	8.961	-11.700	1.00	0.00	H
ATOM	753	HB2	ASP	50	18.432	7.446	-12.180	1.00	0.00	H
ATOM	754	N	MET	51	20.139	6.828	-9.542	1.00	0.00	N
ATOM	755	CA	MET	51	20.321	6.969	-8.102	1.00	0.00	C
ATOM	756	C	MET	51	21.669	6.403	-7.667	1.00	0.00	C
ATOM	757	O	MET	51	22.336	6.964	-6.796	1.00	0.00	O
ATOM	758	CB	MET	51	19.191	6.260	-7.353	1.00	0.00	C
ATOM	759	CG	MET	51	17.869	7.012	-7.398	1.00	0.00	C
ATOM	760	SD	MET	51	16.591	6.228	-6.397	1.00	0.00	S

ATOM	761	CE	MET	51	15.915	5.055	-7.569	1.00	0.00	C
ATOM	762	H	MET	51	19.478	6.187	-9.880	1.00	0.00	H
ATOM	763	HA	MET	51	20.295	8.023	-7.868	1.00	0.00	H
ATOM	764	2HB3	MET	51	19.479	6.144	-6.318	1.00	0.00	H
ATOM	765	2HG3	MET	51	17.531	7.054	-8.423	1.00	0.00	H
ATOM	766	1HE	MET	51	16.700	4.705	-8.223	1.00	0.00	H
ATOM	767	2HE	MET	51	15.492	4.216	-7.036	1.00	0.00	H
ATOM	768	3HE	MET	51	15.145	5.535	-8.156	1.00	0.00	H
ATOM	769	HB2	MET	51	19.006	5.288	-7.807	1.00	0.00	H
ATOM	770	HG2	MET	51	18.008	8.019	-7.004	1.00	0.00	H
ATOM	771	N	ILE	52	22.062	5.287	-8.274	1.00	0.00	N
ATOM	772	CA	ILE	52	23.330	4.648	-7.948	1.00	0.00	C
ATOM	773	C	ILE	52	24.508	5.512	-8.379	1.00	0.00	C
ATOM	774	O	ILE	52	25.460	5.706	-7.622	1.00	0.00	O
ATOM	775	CB	ILE	52	23.450	3.265	-8.616	1.00	0.00	C
ATOM	776	CG2	ILE	52	24.858	2.714	-8.447	1.00	0.00	C
ATOM	777	CG1	ILE	52	22.420	2.298	-8.024	1.00	0.00	C
ATOM	778	CD1	ILE	52	22.093	1.135	-8.933	1.00	0.00	C
ATOM	779	H	ILE	52	21.485	4.888	-8.958	1.00	0.00	H
ATOM	780	HA	ILE	52	23.369	4.513	-6.877	1.00	0.00	H
ATOM	781	HB	ILE	52	23.257	3.380	-9.672	1.00	0.00	H
ATOM	782	1HG2	ILE	52	25.162	2.814	-7.415	1.00	0.00	H
ATOM	783	2HG2	ILE	52	24.874	1.674	-8.727	1.00	0.00	H
ATOM	784	3HG2	ILE	52	25.539	3.268	-9.077	1.00	0.00	H
ATOM	785	2HG3	ILE	52	21.505	2.836	-7.828	1.00	0.00	H
ATOM	786	1HD1	ILE	52	21.991	0.234	-8.343	1.00	0.00	H
ATOM	787	2HD1	ILE	52	21.168	1.333	-9.453	1.00	0.00	H
ATOM	788	3HD1	ILE	52	22.890	1.003	-9.653	1.00	0.00	H
ATOM	789	HG2	ILE	52	22.808	1.877	-7.096	1.00	0.00	H
ATOM	790	N	ASN	53	24.440	6.030	-9.602	1.00	0.00	N
ATOM	791	CA	ASN	53	25.503	6.875	-10.135	1.00	0.00	C
ATOM	792	C	ASN	53	25.601	8.183	-9.353	1.00	0.00	C
ATOM	793	O	ASN	53	26.694	8.703	-9.130	1.00	0.00	O
ATOM	794	CB	ASN	53	25.252	7.172	-11.616	1.00	0.00	C
ATOM	795	CG	ASN	53	25.687	6.029	-12.514	1.00	0.00	C
ATOM	796	OD1	ASN	53	26.879	5.752	-12.654	1.00	0.00	O
ATOM	797	ND2	ASN	53	24.718	5.361	-13.130	1.00	0.00	N
ATOM	798	H	ASN	53	23.655	5.839	-10.160	1.00	0.00	H
ATOM	799	HA	ASN	53	26.434	6.339	-10.035	1.00	0.00	H
ATOM	800	2HB3	ASN	53	25.803	8.056	-11.899	1.00	0.00	H
ATOM	801	1HD2	ASN	53	23.792	5.637	-12.970	1.00	0.00	H
ATOM	802	2HD2	ASN	53	24.970	4.618	-13.715	1.00	0.00	H
ATOM	803	HB2	ASN	53	24.187	7.328	-11.781	1.00	0.00	H
ATOM	804	N	GLU	54	24.451	8.707	-8.940	1.00	0.00	N
ATOM	805	CA	GLU	54	24.410	9.952	-8.185	1.00	0.00	C
ATOM	806	C	GLU	54	25.195	9.826	-6.883	1.00	0.00	C
ATOM	807	O	GLU	54	25.897	10.751	-6.475	1.00	0.00	O
ATOM	808	CB	GLU	54	22.962	10.343	-7.883	1.00	0.00	C
ATOM	809	CG	GLU	54	22.290	11.108	-9.011	1.00	0.00	C
ATOM	810	CD	GLU	54	22.722	12.560	-9.072	1.00	0.00	C
ATOM	811	OE1	GLU	54	23.936	12.823	-8.934	1.00	0.00	O
ATOM	812	OE2	GLU	54	21.848	13.432	-9.255	1.00	0.00	O
ATOM	813	H	GLU	54	23.612	8.245	-9.149	1.00	0.00	H
ATOM	814	HA	GLU	54	24.862	10.724	-8.790	1.00	0.00	H
ATOM	815	2HB3	GLU	54	22.946	10.963	-6.998	1.00	0.00	H
ATOM	816	2HG3	GLU	54	21.221	11.070	-8.867	1.00	0.00	H
ATOM	817	HB2	GLU	54	22.367	9.444	-7.726	1.00	0.00	H
ATOM	818	HG2	GLU	54	22.557	10.655	-9.966	1.00	0.00	H
ATOM	819	N	VAL	55	25.073	8.672	-6.234	1.00	0.00	N
ATOM	820	CA	VAL	55	25.772	8.422	-4.978	1.00	0.00	C
ATOM	821	C	VAL	55	27.179	7.894	-5.229	1.00	0.00	C
ATOM	822	O	VAL	55	28.109	8.191	-4.479	1.00	0.00	O
ATOM	823	CB	VAL	55	25.006	7.415	-4.100	1.00	0.00	C
ATOM	824	CG1	VAL	55	25.810	7.069	-2.856	1.00	0.00	C
ATOM	825	CG2	VAL	55	23.638	7.971	-3.724	1.00	0.00	C
ATOM	826	H	VAL	55	24.499	7.973	-6.609	1.00	0.00	H
ATOM	827	HA	VAL	55	25.839	9.357	-4.443	1.00	0.00	H
ATOM	828	HB	VAL	55	24.858	6.511	-4.671	1.00	0.00	H
ATOM	829	1HG1	VAL	55	25.237	7.320	-1.976	1.00	0.00	H
ATOM	830	2HG1	VAL	55	26.035	6.013	-2.856	1.00	0.00	H
ATOM	831	3HG1	VAL	55	26.730	7.634	-2.858	1.00	0.00	H
ATOM	832	1HG2	VAL	55	23.684	8.391	-2.729	1.00	0.00	H
ATOM	833	2HG2	VAL	55	23.356	8.740	-4.428	1.00	0.00	H
ATOM	834	3HG2	VAL	55	22.909	7.175	-3.746	1.00	0.00	H
ATOM	835	N	ASP	56	27.329	7.105	-6.288	1.00	0.00	N
ATOM	836	CA	ASP	56	28.626	6.534	-6.639	1.00	0.00	C
ATOM	837	C	ASP	56	29.703	7.613	-6.666	1.00	0.00	C

ATOM	838	O	ASP	56	29.641	8.548	-7.463	1.00	0.00	O
ATOM	839	CB	ASP	56	28.547	5.839	-7.999	1.00	0.00	C
ATOM	840	CG	ASP	56	29.612	4.773	-8.166	1.00	0.00	C
ATOM	841	OD1	ASP	56	29.579	3.778	-7.412	1.00	0.00	O
ATOM	842	OD2	ASP	56	30.480	4.935	-9.049	1.00	0.00	O
ATOM	843	H	ASP	56	26.551	6.903	-6.849	1.00	0.00	H
ATOM	844	HA	ASP	56	28.881	5.806	-5.885	1.00	0.00	H
ATOM	845	2HB3	ASP	56	28.674	6.574	-8.780	1.00	0.00	H
ATOM	846	HB2	ASP	56	27.578	5.350	-8.103	1.00	0.00	H
ATOM	847	N	ALA	57	30.692	7.476	-5.787	1.00	0.00	N
ATOM	848	CA	ALA	57	31.784	8.437	-5.711	1.00	0.00	C
ATOM	849	C	ALA	57	33.110	7.790	-6.100	1.00	0.00	C
ATOM	850	O	ALA	57	33.819	8.286	-6.977	1.00	0.00	O
ATOM	851	CB	ALA	57	31.873	9.025	-4.309	1.00	0.00	C
ATOM	852	H	ALA	57	30.686	6.709	-5.177	1.00	0.00	H
ATOM	853	HA	ALA	57	31.571	9.239	-6.400	1.00	0.00	H
ATOM	854	1HB	ALA	57	32.777	8.678	-3.831	1.00	0.00	H
ATOM	855	2HB	ALA	57	31.888	10.103	-4.373	1.00	0.00	H
ATOM	856	3HB	ALA	57	31.017	8.710	-3.733	1.00	0.00	H
ATOM	857	N	ASP	58	33.442	6.687	-5.442	1.00	0.00	N
ATOM	858	CA	ASP	58	34.684	5.974	-5.718	1.00	0.00	C
ATOM	859	C	ASP	58	34.967	5.939	-7.216	1.00	0.00	C
ATOM	860	O	ASP	58	36.092	6.177	-7.652	1.00	0.00	O
ATOM	861	CB	ASP	58	34.612	4.549	-5.168	1.00	0.00	C
ATOM	862	CG	ASP	58	33.705	3.655	-5.991	1.00	0.00	C
ATOM	863	OD1	ASP	58	32.649	4.138	-6.444	1.00	0.00	O
ATOM	864	OD2	ASP	58	34.053	2.469	-6.178	1.00	0.00	O
ATOM	865	H	ASP	58	32.835	6.341	-4.752	1.00	0.00	H
ATOM	866	HA	ASP	58	35.486	6.501	-5.225	1.00	0.00	H
ATOM	867	2HB3	ASP	58	34.235	4.580	-4.156	1.00	0.00	H
ATOM	868	HB2	ASP	58	35.605	4.101	-5.183	1.00	0.00	H
ATOM	869	N	GLY	59	33.935	5.639	-8.003	1.00	0.00	N
ATOM	870	CA	GLY	59	34.096	5.579	-9.444	1.00	0.00	C
ATOM	871	C	GLY	59	34.101	4.156	-9.966	1.00	0.00	C
ATOM	872	O	GLY	59	34.862	3.826	-10.877	1.00	0.00	O
ATOM	873	H	GLY	59	33.062	5.459	-7.600	1.00	0.00	H
ATOM	874	2HA	GLY	59	33.284	6.118	-9.907	1.00	0.00	H
ATOM	875	3HA	GLY	59	35.029	6.052	-9.712	1.00	0.00	H
ATOM	876	N	ASN	60	33.254	3.312	-9.388	1.00	0.00	N
ATOM	877	CA	ASN	60	33.165	1.915	-9.800	1.00	0.00	C
ATOM	878	C	ASN	60	31.796	1.610	-10.401	1.00	0.00	C
ATOM	879	O	ASN	60	31.695	0.994	-11.461	1.00	0.00	O
ATOM	880	CB	ASN	60	33.430	0.992	-8.609	1.00	0.00	C
ATOM	881	CG	ASN	60	32.248	0.921	-7.661	1.00	0.00	C
ATOM	882	OD1	ASN	60	31.776	1.942	-7.161	1.00	0.00	O
ATOM	883	ND2	ASN	60	31.766	-0.289	-7.406	1.00	0.00	N
ATOM	884	H	ASN	60	32.673	3.635	-8.667	1.00	0.00	H
ATOM	885	HA	ASN	60	33.923	1.743	-10.552	1.00	0.00	H
ATOM	886	2HB3	ASN	60	34.287	1.358	-8.062	1.00	0.00	H
ATOM	887	1HD2	ASN	60	32.194	-1.058	-7.840	1.00	0.00	H
ATOM	888	2HD2	ASN	60	31.003	-0.365	-6.798	1.00	0.00	H
ATOM	889	HB2	ASN	60	33.620	-0.018	-8.967	1.00	0.00	H
ATOM	890	N	GLY	61	30.746	2.048	-9.715	1.00	0.00	N
ATOM	891	CA	GLY	61	29.395	1.813	-10.195	1.00	0.00	C
ATOM	892	C	GLY	61	28.431	1.475	-9.076	1.00	0.00	C
ATOM	893	O	GLY	61	27.400	2.128	-8.916	1.00	0.00	O
ATOM	894	H	GLY	61	30.887	2.533	-8.875	1.00	0.00	H
ATOM	895	2HA	GLY	61	29.046	2.698	-10.705	1.00	0.00	H
ATOM	896	3HA	GLY	61	29.415	0.991	-10.898	1.00	0.00	H
ATOM	897	N	THR	62	28.766	0.452	-8.296	1.00	0.00	N
ATOM	898	CA	THR	62	27.921	0.029	-7.187	1.00	0.00	C
ATOM	899	C	THR	62	28.230	0.827	-5.924	1.00	0.00	C
ATOM	900	O	THR	62	29.391	1.006	-5.560	1.00	0.00	O
ATOM	901	CB	THR	62	28.099	-1.471	-6.887	1.00	0.00	C
ATOM	902	OG1	THR	62	29.467	-1.750	-6.566	1.00	0.00	O
ATOM	903	CG2	THR	62	27.673	-2.315	-8.079	1.00	0.00	C
ATOM	904	H	THR	62	29.603	-0.028	-8.472	1.00	0.00	H
ATOM	905	HA	THR	62	26.892	0.199	-7.469	1.00	0.00	H
ATOM	906	HB	THR	62	27.478	-1.731	-6.042	1.00	0.00	H
ATOM	907	1HG	THR	62	29.817	-2.391	-7.187	1.00	0.00	H
ATOM	908	1HG2	THR	62	26.597	-2.414	-8.085	1.00	0.00	H
ATOM	909	2HG2	THR	62	28.123	-3.296	-8.007	1.00	0.00	H
ATOM	910	3HG2	THR	62	27.994	-1.838	-8.990	1.00	0.00	H
ATOM	911	N	ILE	63	27.180	1.304	-5.262	1.00	0.00	N
ATOM	912	CA	ILE	63	27.339	2.081	-4.040	1.00	0.00	C
ATOM	913	C	ILE	63	28.191	1.334	-3.020	1.00	0.00	C
ATOM	914	O	ILE	63	28.183	0.104	-2.972	1.00	0.00	O

ATOM	915	CB	ILE	63	25.976	2.421	-3.408	1.00	0.00	C
ATOM	916	CG2	ILE	63	26.174	3.203	-2.117	1.00	0.00	C
ATOM	917	CG1	ILE	63	25.115	3.214	-4.392	1.00	0.00	C
ATOM	918	CD1	ILE	63	24.067	2.374	-5.089	1.00	0.00	C
ATOM	919	H	ILE	63	26.280	1.129	-5.603	1.00	0.00	H
ATOM	920	HA	ILE	63	27.835	3.007	-4.297	1.00	0.00	H
ATOM	921	HB	ILE	63	25.477	1.495	-3.165	1.00	0.00	H
ATOM	922	1HG2	ILE	63	25.218	3.343	-1.633	1.00	0.00	H
ATOM	923	2HG2	ILE	63	26.833	2.657	-1.462	1.00	0.00	H
ATOM	924	3HG2	ILE	63	26.606	4.167	-2.344	1.00	0.00	H
ATOM	925	2HG3	ILE	63	25.753	3.645	-5.151	1.00	0.00	H
ATOM	926	1HD1	ILE	63	24.538	1.766	-5.847	1.00	0.00	H
ATOM	927	2HD1	ILE	63	23.578	1.738	-4.367	1.00	0.00	H
ATOM	928	3HD1	ILE	63	23.337	3.024	-5.551	1.00	0.00	H
ATOM	929	HG2	ILE	63	24.585	4.002	-3.859	1.00	0.00	H
ATOM	930	N	ASP	64	28.922	2.084	-2.203	1.00	0.00	N
ATOM	931	CA	ASP	64	29.775	1.492	-1.180	1.00	0.00	C
ATOM	932	C	ASP	64	29.285	1.861	0.216	1.00	0.00	C
ATOM	933	O	ASP	64	28.241	2.494	0.373	1.00	0.00	O
ATOM	934	CB	ASP	64	31.223	1.953	-1.364	1.00	0.00	C
ATOM	935	CG	ASP	64	31.711	1.767	-2.786	1.00	0.00	C
ATOM	936	OD1	ASP	64	31.012	1.094	-3.572	1.00	0.00	O
ATOM	937	OD2	ASP	64	32.796	2.296	-3.116	1.00	0.00	O
ATOM	938	H	ASP	64	28.884	3.060	-2.289	1.00	0.00	H
ATOM	939	HA	ASP	64	29.732	0.420	-1.292	1.00	0.00	H
ATOM	940	2HB3	ASP	64	31.861	1.384	-0.705	1.00	0.00	H
ATOM	941	HB2	ASP	64	31.299	3.015	-1.130	1.00	0.00	H
ATOM	942	N	PHE	65	30.045	1.459	1.230	1.00	0.00	N
ATOM	943	CA	PHE	65	29.688	1.743	2.615	1.00	0.00	C
ATOM	944	C	PHE	65	29.653	3.248	2.870	1.00	0.00	C
ATOM	945	O	PHE	65	28.695	3.790	3.421	1.00	0.00	O
ATOM	946	CB	PHE	65	30.680	1.076	3.569	1.00	0.00	C
ATOM	947	CG	PHE	65	30.077	0.691	4.890	1.00	0.00	C
ATOM	948	CD1	PHE	65	29.377	1.620	5.643	1.00	0.00	C
ATOM	949	CE1	PHE	65	28.821	1.270	6.860	1.00	0.00	C
ATOM	950	CZ	PHE	65	28.959	-0.020	7.333	1.00	0.00	C
ATOM	951	CE2	PHE	65	29.654	-0.955	6.591	1.00	0.00	C
ATOM	952	CD2	PHE	65	30.208	-0.598	5.377	1.00	0.00	C
ATOM	953	H	PHE	65	30.867	0.956	1.044	1.00	0.00	H
ATOM	954	HA	PHE	65	28.703	1.339	2.791	1.00	0.00	H
ATOM	955	2HB3	PHE	65	31.496	1.757	3.762	1.00	0.00	H
ATOM	956	1HD	PHE	65	29.268	2.630	5.273	1.00	0.00	H
ATOM	957	1HE	PHE	65	28.279	2.002	7.438	1.00	0.00	H
ATOM	958	HZ	PHE	65	28.526	-0.297	8.281	1.00	0.00	H
ATOM	959	2HE	PHE	65	29.764	-1.964	6.958	1.00	0.00	H
ATOM	960	2HD	PHE	65	30.750	-1.331	4.797	1.00	0.00	H
ATOM	961	HB2	PHE	65	31.058	0.159	3.118	1.00	0.00	H
ATOM	962	N	PRO	66	30.725	3.942	2.456	1.00	0.00	N
ATOM	963	CA	PRO	66	30.842	5.392	2.626	1.00	0.00	C
ATOM	964	C	PRO	66	29.878	6.162	1.732	1.00	0.00	C
ATOM	965	O	PRO	66	29.143	7.031	2.201	1.00	0.00	O
ATOM	966	CD	PRO	66	31.903	3.362	1.790	1.00	0.00	C
ATOM	967	CB	PRO	66	32.291	5.680	2.226	1.00	0.00	C
ATOM	968	CG	PRO	66	32.656	4.570	1.303	1.00	0.00	C
ATOM	969	HA	PRO	66	30.689	5.685	3.656	1.00	0.00	H
ATOM	970	2HB3	PRO	66	32.919	5.685	3.103	1.00	0.00	H
ATOM	971	2HG3	PRO	66	33.720	4.390	1.347	1.00	0.00	H
ATOM	972	2HD3	PRO	66	32.503	2.802	2.490	1.00	0.00	H
ATOM	973	HD2	PRO	66	31.581	2.739	0.956	1.00	0.00	H
ATOM	974	HB2	PRO	66	32.346	6.656	1.739	1.00	0.00	H
ATOM	975	HG2	PRO	66	32.343	4.807	0.285	1.00	0.00	H
ATOM	976	N	GLU	67	29.884	5.838	0.444	1.00	0.00	N
ATOM	977	CA	GLU	67	29.011	6.498	-0.516	1.00	0.00	C
ATOM	978	C	GLU	67	27.548	6.356	-0.107	1.00	0.00	C
ATOM	979	O	GLU	67	26.789	7.325	-0.121	1.00	0.00	O
ATOM	980	CB	GLU	67	29.218	5.917	-1.916	1.00	0.00	C
ATOM	981	CG	GLU	67	30.672	5.898	-2.357	1.00	0.00	C
ATOM	982	CD	GLU	67	30.899	5.035	-3.581	1.00	0.00	C
ATOM	983	OE1	GLU	67	29.909	4.717	-4.275	1.00	0.00	O
ATOM	984	OE2	GLU	67	32.065	4.677	-3.849	1.00	0.00	O
ATOM	985	H	GLU	67	30.493	5.134	0.132	1.00	0.00	H
ATOM	986	HA	GLU	67	29.266	7.548	-0.530	1.00	0.00	H
ATOM	987	2HB3	GLU	67	28.657	6.509	-2.625	1.00	0.00	H
ATOM	988	2HG3	GLU	67	31.275	5.512	-1.547	1.00	0.00	H
ATOM	989	HB2	GLU	67	28.878	4.882	-1.934	1.00	0.00	H
ATOM	990	HG2	GLU	67	30.990	6.910	-2.609	1.00	0.00	H
ATOM	991	N	PHE	68	27.157	5.139	0.256	1.00	0.00	N

ATOM	992	CA	PHE	68	25.783	4.867	0.668	1.00	0.00	C
ATOM	993	C	PHE	68	25.297	5.916	1.665	1.00	0.00	C
ATOM	994	O	PHE	68	24.196	6.450	1.532	1.00	0.00	O
ATOM	995	CB	PHE	68	25.682	3.471	1.287	1.00	0.00	C
ATOM	996	CG	PHE	68	24.386	3.229	2.008	1.00	0.00	C
ATOM	997	CD1	PHE	68	23.218	2.991	1.300	1.00	0.00	C
ATOM	998	CE1	PHE	68	22.024	2.767	1.957	1.00	0.00	C
ATOM	999	CZ	PHE	68	21.988	2.780	3.340	1.00	0.00	C
ATOM	1000	CE2	PHE	68	23.144	3.015	4.057	1.00	0.00	C
ATOM	1001	CD2	PHE	68	24.334	3.240	3.393	1.00	0.00	C
ATOM	1002	H	PHE	68	27.807	4.405	0.247	1.00	0.00	H
ATOM	1003	HA	PHE	68	25.160	4.908	-0.213	1.00	0.00	H
ATOM	1004	2HB3	PHE	68	26.485	3.341	1.996	1.00	0.00	H
ATOM	1005	1HD	PHE	68	23.248	2.980	0.218	1.00	0.00	H
ATOM	1006	1HE	PHE	68	21.124	2.582	1.394	1.00	0.00	H
ATOM	1007	HZ	PHE	68	21.054	2.604	3.857	1.00	0.00	H
ATOM	1008	2HE	PHE	68	23.116	3.026	5.136	1.00	0.00	H
ATOM	1009	2HD	PHE	68	25.238	3.424	3.955	1.00	0.00	H
ATOM	1010	HB2	PHE	68	25.755	2.720	0.502	1.00	0.00	H
ATOM	1011	N	LEU	69	26.125	6.205	2.662	1.00	0.00	N
ATOM	1012	CA	LEU	69	25.781	7.189	3.682	1.00	0.00	C
ATOM	1013	C	LEU	69	25.237	8.464	3.046	1.00	0.00	C
ATOM	1014	O	LEU	69	24.401	9.155	3.630	1.00	0.00	O
ATOM	1015	CB	LEU	69	27.005	7.516	4.539	1.00	0.00	C
ATOM	1016	CG	LEU	69	27.733	6.317	5.154	1.00	0.00	C
ATOM	1017	CD1	LEU	69	28.979	6.773	5.896	1.00	0.00	C
ATOM	1018	CD2	LEU	69	26.804	5.554	6.087	1.00	0.00	C
ATOM	1019	H	LEU	69	26.991	5.747	2.712	1.00	0.00	H
ATOM	1020	HA	LEU	69	25.016	6.760	4.311	1.00	0.00	H
ATOM	1021	2HB3	LEU	69	26.682	8.157	5.344	1.00	0.00	H
ATOM	1022	HG	LEU	69	28.040	5.646	4.365	1.00	0.00	H
ATOM	1023	1HD1	LEU	69	29.754	7.011	5.183	1.00	0.00	H
ATOM	1024	2HD1	LEU	69	29.321	5.982	6.547	1.00	0.00	H
ATOM	1025	3HD1	LEU	69	28.748	7.649	6.485	1.00	0.00	H
ATOM	1026	1HD2	LEU	69	25.960	6.178	6.347	1.00	0.00	H
ATOM	1027	2HD2	LEU	69	27.340	5.283	6.987	1.00	0.00	H
ATOM	1028	3HD2	LEU	69	26.453	4.660	5.594	1.00	0.00	H
ATOM	1029	HB2	LEU	69	27.761	8.006	3.924	1.00	0.00	H
ATOM	1030	N	THR	70	25.715	8.770	1.844	1.00	0.00	N
ATOM	1031	CA	THR	70	25.276	9.962	1.127	1.00	0.00	C
ATOM	1032	C	THR	70	23.798	9.875	0.770	1.00	0.00	C
ATOM	1033	O	THR	70	23.040	10.821	0.986	1.00	0.00	O
ATOM	1034	CB	THR	70	26.093	10.176	-0.161	1.00	0.00	C
ATOM	1035	OG1	THR	70	27.477	10.350	0.159	1.00	0.00	O
ATOM	1036	CG2	THR	70	25.589	11.389	-0.926	1.00	0.00	C
ATOM	1037	H	THR	70	26.379	8.181	1.430	1.00	0.00	H
ATOM	1038	HA	THR	70	25.432	10.815	1.773	1.00	0.00	H
ATOM	1039	HB	THR	70	25.984	9.301	-0.787	1.00	0.00	H
ATOM	1040	1HG	THR	70	27.708	9.785	0.900	1.00	0.00	H
ATOM	1041	1HG2	THR	70	25.717	11.225	-1.986	1.00	0.00	H
ATOM	1042	2HG2	THR	70	26.146	12.262	-0.626	1.00	0.00	H
ATOM	1043	3HG2	THR	70	24.542	11.539	-0.711	1.00	0.00	H
ATOM	1044	N	MET	71	23.393	8.735	0.221	1.00	0.00	N
ATOM	1045	CA	MET	71	22.000	8.525	-0.164	1.00	0.00	C
ATOM	1046	C	MET	71	21.084	8.595	1.053	1.00	0.00	C
ATOM	1047	O	MET	71	20.163	9.412	1.100	1.00	0.00	O
ATOM	1048	CB	MET	71	21.841	7.172	-0.859	1.00	0.00	C
ATOM	1049	CG	MET	71	20.413	6.872	-1.285	1.00	0.00	C
ATOM	1050	SD	MET	71	20.216	5.200	-1.933	1.00	0.00	S
ATOM	1051	CE	MET	71	20.477	4.230	-0.448	1.00	0.00	C
ATOM	1052	H	MET	71	24.043	8.016	0.072	1.00	0.00	H
ATOM	1053	HA	MET	71	21.727	9.309	-0.853	1.00	0.00	H
ATOM	1054	2HB3	MET	71	22.165	6.393	-0.186	1.00	0.00	H
ATOM	1055	2HG3	MET	71	20.125	7.577	-2.050	1.00	0.00	H
ATOM	1056	1HE	MET	71	21.536	4.092	-0.289	1.00	0.00	H
ATOM	1057	2HE	MET	71	20.050	4.747	0.399	1.00	0.00	H
ATOM	1058	3HE	MET	71	20.000	3.266	-0.560	1.00	0.00	H
ATOM	1059	HB2	MET	71	22.442	7.158	-1.770	1.00	0.00	H
ATOM	1060	HG2	MET	71	19.752	6.957	-0.424	1.00	0.00	H
ATOM	1061	N	MET	72	21.341	7.738	2.032	1.00	0.00	N
ATOM	1062	CA	MET	72	20.537	7.705	3.250	1.00	0.00	C
ATOM	1063	C	MET	72	20.165	9.115	3.693	1.00	0.00	C
ATOM	1064	O	MET	72	19.175	9.312	4.399	1.00	0.00	O
ATOM	1065	CB	MET	72	21.295	6.989	4.369	1.00	0.00	C
ATOM	1066	CG	MET	72	21.198	5.473	4.296	1.00	0.00	C
ATOM	1067	SD	MET	72	21.283	4.694	5.921	1.00	0.00	S
ATOM	1068	CE	MET	72	19.675	5.121	6.584	1.00	0.00	C

```
ATOM   1069  H   MET    72      22.088   7.111   1.937  1.00  0.00           H
ATOM   1070  HA  MET    72      19.631   7.155   3.033  1.00  0.00           H
ATOM   1071 2HB3 MET    72      20.897   7.308   5.319  1.00  0.00           H
ATOM   1072 2HG3 MET    72      22.012   5.103   3.690  1.00  0.00           H
ATOM   1073 1HE  MET    72      19.435   6.140   6.319  1.00  0.00           H
ATOM   1074 2HE  MET    72      18.929   4.456   6.175  1.00  0.00           H
ATOM   1075 3HE  MET    72      19.695   5.024   7.661  1.00  0.00           H
ATOM   1076  HB2 MET    72      22.355   7.232   4.302  1.00  0.00           H
ATOM   1077  HG2 MET    72      20.242   5.190   3.857  1.00  0.00           H
ATOM   1078  N   ALA    73      20.961  10.092   3.276  1.00  0.00           N
ATOM   1079  CA  ALA    73      20.715  11.484   3.629  1.00  0.00           C
ATOM   1080  C   ALA    73      19.676  12.109   2.702  1.00  0.00           C
ATOM   1081  O   ALA    73      18.728  12.748   3.161  1.00  0.00           O
ATOM   1082  CB  ALA    73      22.010  12.280   3.583  1.00  0.00           C
ATOM   1083  H   ALA    73      21.735   9.871   2.716  1.00  0.00           H
ATOM   1084  HA  ALA    73      20.341  11.509   4.643  1.00  0.00           H
ATOM   1085 1HB  ALA    73      22.810  11.644   3.227  1.00  0.00           H
ATOM   1086 2HB  ALA    73      21.895  13.119   2.914  1.00  0.00           H
ATOM   1087 3HB  ALA    73      22.248  12.637   4.573  1.00  0.00           H
ATOM   1088  N   ARG    74      19.861  11.922   1.401  1.00  0.00           N
ATOM   1089  CA  ARG    74      18.940  12.469   0.411  1.00  0.00           C
ATOM   1090  C   ARG    74      17.665  11.635   0.336  1.00  0.00           C
ATOM   1091  O   ARG    74      16.575  12.166   0.121  1.00  0.00           O
ATOM   1092  CB  ARG    74      19.609  12.521  -0.963  1.00  0.00           C
ATOM   1093  CG  ARG    74      20.922  13.288  -0.975  1.00  0.00           C
ATOM   1094  CD  ARG    74      21.891  12.719  -1.999  1.00  0.00           C
ATOM   1095  NE  ARG    74      21.780  13.391  -3.289  1.00  0.00           N
ATOM   1096  CZ  ARG    74      22.432  14.507  -3.595  1.00  0.00           C
ATOM   1097  NH1 ARG    74      23.238  15.073  -2.708  1.00  0.00           N
ATOM   1098  NH2 ARG    74      22.278  15.059  -4.794  1.00  0.00           N
ATOM   1099  H   ARG    74      20.635  11.404   1.097  1.00  0.00           H
ATOM   1100  HA  ARG    74      18.684  13.472   0.716  1.00  0.00           H
ATOM   1101 2HB3 ARG    74      18.935  12.997  -1.660  1.00  0.00           H
ATOM   1102 2HG3 ARG    74      21.371  13.227   0.007  1.00  0.00           H
ATOM   1103 2HD3 ARG    74      21.679  11.668  -2.130  1.00  0.00           H
ATOM   1104  HE  ARG    74      21.188  12.991  -3.960  1.00  0.00           H
ATOM   1105 1HH1 ARG    74      23.357  14.657  -1.807  1.00  0.00           H
ATOM   1106 2HH1 ARG    74      23.729  15.913  -2.943  1.00  0.00           H
ATOM   1107 1HH2 ARG    74      21.672  14.637  -5.463  1.00  0.00           H
ATOM   1108 2HH2 ARG    74      22.769  15.900  -5.021  1.00  0.00           H
ATOM   1109  HB2 ARG    74      19.843  11.509  -1.295  1.00  0.00           H
ATOM   1110  HG2 ARG    74      20.734  14.329  -1.234  1.00  0.00           H
ATOM   1111  HD2 ARG    74      22.913  12.855  -1.647  1.00  0.00           H
ATOM   1112  N   LYS    75      17.807  10.327   0.515  1.00  0.00           N
ATOM   1113  CA  LYS    75      16.667   9.419   0.469  1.00  0.00           C
ATOM   1114  C   LYS    75      15.733   9.657   1.651  1.00  0.00           C
ATOM   1115  O   LYS    75      14.593   9.194   1.656  1.00  0.00           O
ATOM   1116  CB  LYS    75      17.147   7.965   0.468  1.00  0.00           C
ATOM   1117  CG  LYS    75      16.022   6.954   0.317  1.00  0.00           C
ATOM   1118  CD  LYS    75      15.327   7.092  -1.028  1.00  0.00           C
ATOM   1119  CE  LYS    75      16.234   6.664  -2.171  1.00  0.00           C
ATOM   1120  NZ  LYS    75      17.032   7.806  -2.701  1.00  0.00           N
ATOM   1121  H   LYS    75      18.701   9.963   0.682  1.00  0.00           H
ATOM   1122  HA  LYS    75      16.127   9.611  -0.448  1.00  0.00           H
ATOM   1123 2HB3 LYS    75      17.657   7.768   1.400  1.00  0.00           H
ATOM   1124 2HG3 LYS    75      15.299   7.115   1.104  1.00  0.00           H
ATOM   1125 2HD3 LYS    75      15.042   8.124  -1.171  1.00  0.00           H
ATOM   1126 2HE3 LYS    75      15.624   6.261  -2.966  1.00  0.00           H
ATOM   1127 1HZ  LYS    75      16.581   8.187  -3.557  1.00  0.00           H
ATOM   1128 2HZ  LYS    75      17.992   7.487  -2.942  1.00  0.00           H
ATOM   1129 3HZ  LYS    75      17.096   8.559  -1.988  1.00  0.00           H
ATOM   1130  HB2 LYS    75      17.820   7.804  -0.374  1.00  0.00           H
ATOM   1131  HG2 LYS    75      16.428   5.946   0.382  1.00  0.00           H
ATOM   1132  HD2 LYS    75      14.445   6.453  -1.047  1.00  0.00           H
ATOM   1133  HE2 LYS    75      16.936   5.908  -1.816  1.00  0.00           H
ATOM   1134  N   MET    76      16.224  10.385   2.648  1.00  0.00           N
ATOM   1135  CA  MET    76      15.432  10.687   3.835  1.00  0.00           C
ATOM   1136  C   MET    76      14.319  11.677   3.509  1.00  0.00           C
ATOM   1137  O   MET    76      13.247  11.645   4.112  1.00  0.00           O
ATOM   1138  CB  MET    76      16.325  11.254   4.941  1.00  0.00           C
ATOM   1139  CG  MET    76      16.778  10.210   5.950  1.00  0.00           C
ATOM   1140  SD  MET    76      15.415   9.548   6.925  1.00  0.00           S
ATOM   1141  CE  MET    76      15.240   7.918   6.201  1.00  0.00           C
ATOM   1142  H   MET    76      17.140  10.727   2.586  1.00  0.00           H
ATOM   1143  HA  MET    76      14.988   9.766   4.179  1.00  0.00           H
ATOM   1144 2HB3 MET    76      15.779  12.020   5.471  1.00  0.00           H
ATOM   1145 2HG3 MET    76      17.495  10.663   6.617  1.00  0.00           H
```

ATOM	1146	1HE	MET	76	15.156	8.010	5.128	1.00	0.00	H
ATOM	1147	2HE	MET	76	16.104	7.321	6.446	1.00	0.00	H
ATOM	1148	3HE	MET	76	14.351	7.446	6.593	1.00	0.00	H
ATOM	1149	HB2	MET	76	17.230	11.669	4.500	1.00	0.00	H
ATOM	1150	HG2	MET	76	17.228	9.367	5.425	1.00	0.00	H
ATOM	1151	N	LYS	77	14.578	12.557	2.546	1.00	0.00	N
ATOM	1152	CA	LYS	77	13.600	13.557	2.139	1.00	0.00	C
ATOM	1153	C	LYS	77	12.686	13.008	1.046	1.00	0.00	C
ATOM	1154	O	LYS	77	11.512	13.372	0.964	1.00	0.00	O
ATOM	1155	CB	LYS	77	14.307	14.819	1.640	1.00	0.00	C
ATOM	1156	CG	LYS	77	14.757	14.734	0.190	1.00	0.00	C
ATOM	1157	CD	LYS	77	13.676	15.230	-0.756	1.00	0.00	C
ATOM	1158	CE	LYS	77	13.603	16.749	-0.770	1.00	0.00	C
ATOM	1159	NZ	LYS	77	14.771	17.357	-1.462	1.00	0.00	N
ATOM	1160	H	LYS	77	15.452	12.534	2.101	1.00	0.00	H
ATOM	1161	HA	LYS	77	12.999	13.807	3.001	1.00	0.00	H
ATOM	1162	2HB3	LYS	77	15.177	14.994	2.255	1.00	0.00	H
ATOM	1163	2HG3	LYS	77	14.985	13.704	-0.045	1.00	0.00	H
ATOM	1164	2HD3	LYS	77	12.721	14.832	-0.441	1.00	0.00	H
ATOM	1165	2HE3	LYS	77	13.575	17.104	0.249	1.00	0.00	H
ATOM	1166	1HZ	LYS	77	15.378	17.849	-0.775	1.00	0.00	H
ATOM	1167	2HZ	LYS	77	14.450	18.039	-2.176	1.00	0.00	H
ATOM	1168	3HZ	LYS	77	15.331	16.617	-1.936	1.00	0.00	H
ATOM	1169	HB2	LYS	77	13.623	15.666	1.699	1.00	0.00	H
ATOM	1170	HG2	LYS	77	15.640	15.353	0.046	1.00	0.00	H
ATOM	1171	HD2	LYS	77	13.901	14.898	-1.770	1.00	0.00	H
ATOM	1172	HE2	LYS	77	12.704	17.066	-1.302	1.00	0.00	H
ATOM	1173	N	ASP	78	13.232	12.131	0.210	1.00	0.00	N
ATOM	1174	CA	ASP	78	12.464	11.531	-0.875	1.00	0.00	C
ATOM	1175	C	ASP	78	11.198	10.869	-0.344	1.00	0.00	C
ATOM	1176	O	ASP	78	10.170	10.838	-1.021	1.00	0.00	O
ATOM	1177	CB	ASP	78	13.317	10.503	-1.622	1.00	0.00	C
ATOM	1178	CG	ASP	78	14.184	11.138	-2.694	1.00	0.00	C
ATOM	1179	OD1	ASP	78	14.617	12.293	-2.498	1.00	0.00	O
ATOM	1180	OD2	ASP	78	14.426	10.480	-3.728	1.00	0.00	O
ATOM	1181	H	ASP	78	14.172	11.881	0.328	1.00	0.00	H
ATOM	1182	HA	ASP	78	12.186	12.318	-1.557	1.00	0.00	H
ATOM	1183	2HB3	ASP	78	12.668	9.780	-2.091	1.00	0.00	H
ATOM	1184	HB2	ASP	78	13.984	10.003	-0.919	1.00	0.00	H
ATOM	1185	N	THR	79	11.279	10.338	0.873	1.00	0.00	N
ATOM	1186	CA	THR	79	10.141	9.675	1.494	1.00	0.00	C
ATOM	1187	C	THR	79	9.213	10.683	2.163	1.00	0.00	C
ATOM	1188	O	THR	79	8.049	10.386	2.435	1.00	0.00	O
ATOM	1189	CB	THR	79	10.596	8.640	2.542	1.00	0.00	C
ATOM	1190	OG1	THR	79	11.425	7.650	1.923	1.00	0.00	O
ATOM	1191	CG2	THR	79	9.397	7.969	3.197	1.00	0.00	C
ATOM	1192	H	THR	79	12.126	10.394	1.362	1.00	0.00	H
ATOM	1193	HA	THR	79	9.592	9.155	0.720	1.00	0.00	H
ATOM	1194	HB	THR	79	11.165	9.150	3.305	1.00	0.00	H
ATOM	1195	1HG	THR	79	11.755	7.985	1.087	1.00	0.00	H
ATOM	1196	1HG2	THR	79	9.740	7.201	3.872	1.00	0.00	H
ATOM	1197	2HG2	THR	79	8.772	7.526	2.435	1.00	0.00	H
ATOM	1198	3HG2	THR	79	8.830	8.705	3.746	1.00	0.00	H
ATOM	1199	N	ASP	80	9.736	11.877	2.424	1.00	0.00	N
ATOM	1200	CA	ASP	80	8.953	12.929	3.060	1.00	0.00	C
ATOM	1201	C	ASP	80	7.537	12.973	2.495	1.00	0.00	C
ATOM	1202	O	ASP	80	6.584	13.296	3.202	1.00	0.00	O
ATOM	1203	CB	ASP	80	9.634	14.286	2.867	1.00	0.00	C
ATOM	1204	CG	ASP	80	9.239	15.289	3.934	1.00	0.00	C
ATOM	1205	OD1	ASP	80	8.186	15.941	3.775	1.00	0.00	O
ATOM	1206	OD2	ASP	80	9.983	15.420	4.926	1.00	0.00	O
ATOM	1207	H	ASP	80	10.669	12.052	2.184	1.00	0.00	H
ATOM	1208	HA	ASP	80	8.899	12.713	4.116	1.00	0.00	H
ATOM	1209	2HB3	ASP	80	9.357	14.684	1.903	1.00	0.00	H
ATOM	1210	HB2	ASP	80	10.717	14.161	2.921	1.00	0.00	H
ATOM	1211	N	SER	81	7.407	12.645	1.213	1.00	0.00	N
ATOM	1212	CA	SER	81	6.109	12.649	0.551	1.00	0.00	C
ATOM	1213	C	SER	81	5.020	12.137	1.486	1.00	0.00	C
ATOM	1214	O	SER	81	3.859	12.536	1.384	1.00	0.00	O
ATOM	1215	CB	SER	81	6.154	11.791	-0.716	1.00	0.00	C
ATOM	1216	OG	SER	81	7.103	12.298	-1.640	1.00	0.00	O
ATOM	1217	H	SER	81	8.206	12.395	0.699	1.00	0.00	H
ATOM	1218	HA	SER	81	5.879	13.668	0.276	1.00	0.00	H
ATOM	1219	2HB3	SER	81	5.181	11.791	-1.183	1.00	0.00	H
ATOM	1220	HG	SER	81	7.910	11.779	-1.588	1.00	0.00	H
ATOM	1221	HB2	SER	81	6.435	10.772	-0.454	1.00	0.00	H
ATOM	1222	N	GLU	82	5.401	11.247	2.397	1.00	0.00	N

ATOM	1223	CA	GLU	82	4.457	10.677	3.352	1.00	0.00	C
ATOM	1224	C	GLU	82	3.383	11.693	3.728	1.00	0.00	C
ATOM	1225	O	GLU	82	2.190	11.415	3.621	1.00	0.00	O
ATOM	1226	CB	GLU	82	5.191	10.205	4.609	1.00	0.00	C
ATOM	1227	CG	GLU	82	4.268	9.626	5.669	1.00	0.00	C
ATOM	1228	CD	GLU	82	4.974	9.400	6.994	1.00	0.00	C
ATOM	1229	OE1	GLU	82	5.945	10.130	7.283	1.00	0.00	O
ATOM	1230	OE2	GLU	82	4.553	8.490	7.740	1.00	0.00	O
ATOM	1231	H	GLU	82	6.342	10.968	2.428	1.00	0.00	H
ATOM	1232	HA	GLU	82	3.984	9.827	2.882	1.00	0.00	H
ATOM	1233	2HB3	GLU	82	5.717	11.044	5.039	1.00	0.00	H
ATOM	1234	2HG3	GLU	82	3.886	8.680	5.316	1.00	0.00	H
ATOM	1235	HB2	GLU	82	5.887	9.410	4.348	1.00	0.00	H
ATOM	1236	HG2	GLU	82	3.447	10.319	5.855	1.00	0.00	H
ATOM	1237	N	GLU	83	3.818	12.870	4.165	1.00	0.00	N
ATOM	1238	CA	GLU	83	2.894	13.926	4.558	1.00	0.00	C
ATOM	1239	C	GLU	83	1.951	14.281	3.411	1.00	0.00	C
ATOM	1240	O	GLU	83	0.732	14.287	3.573	1.00	0.00	O
ATOM	1241	CB	GLU	83	3.665	15.172	5.001	1.00	0.00	C
ATOM	1242	CG	GLU	83	4.589	14.926	6.181	1.00	0.00	C
ATOM	1243	CD	GLU	83	4.789	16.167	7.033	1.00	0.00	C
ATOM	1244	OE1	GLU	83	4.901	17.269	6.459	1.00	0.00	O
ATOM	1245	OE2	GLU	83	4.832	16.032	8.275	1.00	0.00	O
ATOM	1246	H	GLU	83	4.783	13.031	4.228	1.00	0.00	H
ATOM	1247	HA	GLU	83	2.307	13.565	5.390	1.00	0.00	H
ATOM	1248	2HB3	GLU	83	2.957	15.939	5.278	1.00	0.00	H
ATOM	1249	2HG3	GLU	83	5.550	14.604	5.810	1.00	0.00	H
ATOM	1250	HB2	GLU	83	4.300	15.517	4.186	1.00	0.00	H
ATOM	1251	HG2	GLU	83	4.160	14.160	6.827	1.00	0.00	H
ATOM	1252	N	GLU	84	2.528	14.575	2.251	1.00	0.00	N
ATOM	1253	CA	GLU	84	1.741	14.931	1.074	1.00	0.00	C
ATOM	1254	C	GLU	84	0.483	14.071	0.982	1.00	0.00	C
ATOM	1255	O	GLU	84	-0.531	14.495	0.426	1.00	0.00	O
ATOM	1256	CB	GLU	84	2.579	14.770	-0.197	1.00	0.00	C
ATOM	1257	CG	GLU	84	3.699	15.786	-0.318	1.00	0.00	C
ATOM	1258	CD	GLU	84	3.195	17.174	-0.666	1.00	0.00	C
ATOM	1259	OE1	GLU	84	3.034	17.462	-1.870	1.00	0.00	O
ATOM	1260	OE2	GLU	84	2.963	17.972	0.265	1.00	0.00	O
ATOM	1261	H	GLU	84	3.505	14.554	2.183	1.00	0.00	H
ATOM	1262	HA	GLU	84	1.447	15.965	1.173	1.00	0.00	H
ATOM	1263	2HB3	GLU	84	1.931	14.874	-1.054	1.00	0.00	H
ATOM	1264	2HG3	GLU	84	4.380	15.464	-1.093	1.00	0.00	H
ATOM	1265	HB2	GLU	84	3.057	13.791	-0.195	1.00	0.00	H
ATOM	1266	HG2	GLU	84	4.226	15.863	0.633	1.00	0.00	H
ATOM	1267	N	ILE	85	0.558	12.862	1.527	1.00	0.00	N
ATOM	1268	CA	ILE	85	-0.572	11.942	1.506	1.00	0.00	C
ATOM	1269	C	ILE	85	-1.482	12.165	2.707	1.00	0.00	C
ATOM	1270	O	ILE	85	-2.705	12.060	2.602	1.00	0.00	O
ATOM	1271	CB	ILE	85	-0.103	10.475	1.493	1.00	0.00	C
ATOM	1272	CG2	ILE	85	-1.298	9.539	1.384	1.00	0.00	C
ATOM	1273	CG1	ILE	85	0.874	10.238	0.342	1.00	0.00	C
ATOM	1274	CD1	ILE	85	1.487	8.856	0.343	1.00	0.00	C
ATOM	1275	H	ILE	85	1.395	12.582	1.955	1.00	0.00	H
ATOM	1276	HA	ILE	85	-1.134	12.128	0.603	1.00	0.00	H
ATOM	1277	HB	ILE	85	0.397	10.272	2.430	1.00	0.00	H
ATOM	1278	1HG2	ILE	85	-1.965	9.898	0.612	1.00	0.00	H
ATOM	1279	2HG2	ILE	85	-0.956	8.547	1.130	1.00	0.00	H
ATOM	1280	3HG2	ILE	85	-1.822	9.509	2.327	1.00	0.00	H
ATOM	1281	2HG3	ILE	85	1.677	10.957	0.407	1.00	0.00	H
ATOM	1282	1HD1	ILE	85	1.059	8.272	1.146	1.00	0.00	H
ATOM	1283	2HD1	ILE	85	1.286	8.370	-0.599	1.00	0.00	H
ATOM	1284	3HD1	ILE	85	2.555	8.934	0.488	1.00	0.00	H
ATOM	1285	HG2	ILE	85	0.351	10.352	-0.608	1.00	0.00	H
ATOM	1286	N	ARG	86	-0.880	12.473	3.852	1.00	0.00	N
ATOM	1287	CA	ARG	86	-1.636	12.709	5.075	1.00	0.00	C
ATOM	1288	C	ARG	86	-2.902	13.511	4.783	1.00	0.00	C
ATOM	1289	O	ARG	86	-4.010	13.065	5.073	1.00	0.00	O
ATOM	1290	CB	ARG	86	-0.774	13.451	6.099	1.00	0.00	C
ATOM	1291	CG	ARG	86	0.266	12.571	6.771	1.00	0.00	C
ATOM	1292	CD	ARG	86	-0.273	11.952	8.053	1.00	0.00	C
ATOM	1293	NE	ARG	86	-0.596	12.965	9.053	1.00	0.00	N
ATOM	1294	CZ	ARG	86	-0.625	12.726	10.360	1.00	0.00	C
ATOM	1295	NH1	ARG	86	-0.350	11.513	10.821	1.00	0.00	N
ATOM	1296	NH2	ARG	86	-0.928	13.699	11.209	1.00	0.00	N
ATOM	1297	H	ARG	86	0.098	12.542	3.873	1.00	0.00	H
ATOM	1298	HA	ARG	86	-1.919	11.750	5.482	1.00	0.00	H
ATOM	1299	2HB3	ARG	86	-1.416	13.863	6.863	1.00	0.00	H

ATOM	1300	2HG3	ARG	86	1.133	13.168	7.006	1.00	0.00	H
ATOM	1301	2HD3	ARG	86	0.473	11.285	8.456	1.00	0.00	H
ATOM	1302	HE	ARG	86	-0.805	13.868	8.736	1.00	0.00	H
ATOM	1303	1HH1	ARG	86	-0.121	10.777	10.183	1.00	0.00	H
ATOM	1304	2HH1	ARG	86	-0.372	11.335	11.806	1.00	0.00	H
ATOM	1305	1HH2	ARG	86	-1.135	14.613	10.865	1.00	0.00	H
ATOM	1306	2HH2	ARG	86	-0.949	13.517	12.191	1.00	0.00	H
ATOM	1307	HB2	ARG	86	-0.221	14.247	5.599	1.00	0.00	H
ATOM	1308	HG2	ARG	86	0.543	11.758	6.099	1.00	0.00	H
ATOM	1309	HD2	ARG	86	-1.190	11.404	7.835	1.00	0.00	H
ATOM	1310	N	GLU	87	-2.726	14.698	4.211	1.00	0.00	N
ATOM	1311	CA	GLU	87	-3.853	15.563	3.882	1.00	0.00	C
ATOM	1312	C	GLU	87	-5.058	14.739	3.436	1.00	0.00	C
ATOM	1313	O	GLU	87	-6.162	14.902	3.955	1.00	0.00	O
ATOM	1314	CB	GLU	87	-3.464	16.554	2.785	1.00	0.00	C
ATOM	1315	CG	GLU	87	-2.404	17.555	3.216	1.00	0.00	C
ATOM	1316	CD	GLU	87	-2.980	18.701	4.024	1.00	0.00	C
ATOM	1317	OE1	GLU	87	-3.739	19.509	3.449	1.00	0.00	O
ATOM	1318	OE2	GLU	87	-2.671	18.791	5.231	1.00	0.00	O
ATOM	1319	H	GLU	87	-1.817	15.000	4.005	1.00	0.00	H
ATOM	1320	HA	GLU	87	-4.121	16.113	4.773	1.00	0.00	H
ATOM	1321	2HB3	GLU	87	-4.343	17.102	2.483	1.00	0.00	H
ATOM	1322	2HG3	GLU	87	-1.928	17.958	2.335	1.00	0.00	H
ATOM	1323	HB2	GLU	87	-3.043	16.012	1.938	1.00	0.00	H
ATOM	1324	HG2	GLU	87	-1.667	17.054	3.846	1.00	0.00	H
ATOM	1325	N	ALA	88	-4.835	13.855	2.469	1.00	0.00	N
ATOM	1326	CA	ALA	88	-5.901	13.004	1.953	1.00	0.00	C
ATOM	1327	C	ALA	88	-6.567	12.214	3.074	1.00	0.00	C
ATOM	1328	O	ALA	88	-7.792	12.192	3.191	1.00	0.00	O
ATOM	1329	CB	ALA	88	-5.354	12.059	0.892	1.00	0.00	C
ATOM	1330	H	ALA	88	-3.934	13.769	2.098	1.00	0.00	H
ATOM	1331	HA	ALA	88	-6.639	13.640	1.487	1.00	0.00	H
ATOM	1332	1HB	ALA	88	-4.409	11.655	1.224	1.00	0.00	H
ATOM	1333	2HB	ALA	88	-6.056	11.255	0.733	1.00	0.00	H
ATOM	1334	3HB	ALA	88	-5.210	12.601	-0.030	1.00	0.00	H
ATOM	1335	N	PHE	89	-5.752	11.566	3.902	1.00	0.00	N
ATOM	1336	CA	PHE	89	-6.261	10.775	5.013	1.00	0.00	C
ATOM	1337	C	PHE	89	-7.240	11.586	5.855	1.00	0.00	C
ATOM	1338	O	PHE	89	-8.293	11.087	6.255	1.00	0.00	O
ATOM	1339	CB	PHE	89	-5.106	10.281	5.888	1.00	0.00	C
ATOM	1340	CG	PHE	89	-5.511	9.981	7.303	1.00	0.00	C
ATOM	1341	CD1	PHE	89	-6.134	8.788	7.621	1.00	0.00	C
ATOM	1342	CE1	PHE	89	-6.510	8.511	8.923	1.00	0.00	C
ATOM	1343	CZ	PHE	89	-6.258	9.431	9.923	1.00	0.00	C
ATOM	1344	CE2	PHE	89	-5.636	10.626	9.618	1.00	0.00	C
ATOM	1345	CD2	PHE	89	-5.266	10.896	8.315	1.00	0.00	C
ATOM	1346	H	PHE	89	-4.782	11.623	3.756	1.00	0.00	H
ATOM	1347	HA	PHE	89	-6.778	9.920	4.602	1.00	0.00	H
ATOM	1348	2HB3	PHE	89	-4.336	11.036	5.912	1.00	0.00	H
ATOM	1349	1HD	PHE	89	-6.329	8.067	6.842	1.00	0.00	H
ATOM	1350	1HE	PHE	89	-6.994	7.576	9.160	1.00	0.00	H
ATOM	1351	HZ	PHE	89	-6.550	9.218	10.940	1.00	0.00	H
ATOM	1352	2HE	PHE	89	-5.441	11.347	10.396	1.00	0.00	H
ATOM	1353	2HD	PHE	89	-4.780	11.833	8.076	1.00	0.00	H
ATOM	1354	HB2	PHE	89	-4.708	9.353	5.477	1.00	0.00	H
ATOM	1355	N	ARG	90	-6.885	12.839	6.120	1.00	0.00	N
ATOM	1356	CA	ARG	90	-7.732	13.719	6.917	1.00	0.00	C
ATOM	1357	C	ARG	90	-9.118	13.853	6.292	1.00	0.00	C
ATOM	1358	O	ARG	90	-10.132	13.648	6.957	1.00	0.00	O
ATOM	1359	CB	ARG	90	-7.085	15.099	7.049	1.00	0.00	C
ATOM	1360	CG	ARG	90	-5.864	15.117	7.952	1.00	0.00	C
ATOM	1361	CD	ARG	90	-4.957	16.297	7.642	1.00	0.00	C
ATOM	1362	NE	ARG	90	-4.040	16.586	8.742	1.00	0.00	N
ATOM	1363	CZ	ARG	90	-4.363	17.332	9.793	1.00	0.00	C
ATOM	1364	NH1	ARG	90	-5.575	17.861	9.885	1.00	0.00	N
ATOM	1365	NH2	ARG	90	-3.474	17.549	10.751	1.00	0.00	N
ATOM	1366	H	ARG	90	-6.035	13.179	5.773	1.00	0.00	H
ATOM	1367	HA	ARG	90	-7.835	13.283	7.898	1.00	0.00	H
ATOM	1368	2HB3	ARG	90	-7.815	15.787	7.452	1.00	0.00	H
ATOM	1369	2HG3	ARG	90	-5.310	14.201	7.809	1.00	0.00	H
ATOM	1370	2HD3	ARG	90	-5.570	17.167	7.460	1.00	0.00	H
ATOM	1371	HE	ARG	90	-3.139	16.206	8.692	1.00	0.00	H
ATOM	1372	1HH1	ARG	90	-6.249	17.696	9.165	1.00	0.00	H
ATOM	1373	2HH1	ARG	90	-5.818	18.421	10.679	1.00	0.00	H
ATOM	1374	1HH2	ARG	90	-2.558	17.152	10.684	1.00	0.00	H
ATOM	1375	2HH2	ARG	90	-3.719	18.111	11.541	1.00	0.00	H
ATOM	1376	HB2	ARG	90	-6.745	15.438	6.070	1.00	0.00	H

ATOM	1377	HG2	ARG	90	-6.181	15.203	8.992	1.00	0.00	H
ATOM	1378	HD2	ARG	90	-4.355	16.071	6.764	1.00	0.00	H
ATOM	1379	N	VAL	91	-9.153	14.199	5.010	1.00	0.00	N
ATOM	1380	CA	VAL	91	-10.413	14.360	4.294	1.00	0.00	C
ATOM	1381	C	VAL	91	-11.371	13.217	4.609	1.00	0.00	C
ATOM	1382	O	VAL	91	-12.509	13.442	5.024	1.00	0.00	O
ATOM	1383	CB	VAL	91	-10.190	14.427	2.772	1.00	0.00	C
ATOM	1384	CG1	VAL	91	-11.520	14.535	2.041	1.00	0.00	C
ATOM	1385	CG2	VAL	91	-9.279	15.591	2.416	1.00	0.00	C
ATOM	1386	H	VAL	91	-8.309	14.352	4.532	1.00	0.00	H
ATOM	1387	HA	VAL	91	-10.861	15.291	4.609	1.00	0.00	H
ATOM	1388	HB	VAL	91	-9.709	13.511	2.459	1.00	0.00	H
ATOM	1389	1HG1	VAL	91	-12.086	15.360	2.448	1.00	0.00	H
ATOM	1390	2HG1	VAL	91	-11.339	14.704	0.989	1.00	0.00	H
ATOM	1391	3HG1	VAL	91	-12.076	13.619	2.170	1.00	0.00	H
ATOM	1392	1HG2	VAL	91	-9.757	16.206	1.667	1.00	0.00	H
ATOM	1393	2HG2	VAL	91	-9.089	16.182	3.300	1.00	0.00	H
ATOM	1394	3HG2	VAL	91	-8.345	15.214	2.028	1.00	0.00	H
ATOM	1395	N	PHE	92	-10.906	11.989	4.405	1.00	0.00	N
ATOM	1396	CA	PHE	92	-11.722	10.809	4.666	1.00	0.00	C
ATOM	1397	C	PHE	92	-12.180	10.776	6.121	1.00	0.00	C
ATOM	1398	O	PHE	92	-13.360	10.570	6.408	1.00	0.00	O
ATOM	1399	CB	PHE	92	-10.938	9.538	4.335	1.00	0.00	C
ATOM	1400	CG	PHE	92	-10.870	9.240	2.865	1.00	0.00	C
ATOM	1401	CD1	PHE	92	-9.886	9.815	2.074	1.00	0.00	C
ATOM	1402	CE1	PHE	92	-9.820	9.543	0.722	1.00	0.00	C
ATOM	1403	CZ	PHE	92	-10.740	8.691	0.143	1.00	0.00	C
ATOM	1404	CE2	PHE	92	-11.725	8.113	0.919	1.00	0.00	C
ATOM	1405	CD2	PHE	92	-11.785	8.388	2.273	1.00	0.00	C
ATOM	1406	H	PHE	92	-9.992	11.874	4.072	1.00	0.00	H
ATOM	1407	HA	PHE	92	-12.592	10.861	4.028	1.00	0.00	H
ATOM	1408	2HB3	PHE	92	-11.410	8.698	4.824	1.00	0.00	H
ATOM	1409	1HD	PHE	92	-9.166	10.481	2.526	1.00	0.00	H
ATOM	1410	1HE	PHE	92	-9.049	9.997	0.116	1.00	0.00	H
ATOM	1411	HZ	PHE	92	-10.691	8.477	-0.915	1.00	0.00	H
ATOM	1412	2HE	PHE	92	-12.447	7.444	0.469	1.00	0.00	H
ATOM	1413	2HD	PHE	92	-12.557	7.934	2.880	1.00	0.00	H
ATOM	1414	HB2	PHE	92	-9.912	9.642	4.683	1.00	0.00	H
ATOM	1415	N	ASP	93	-11.238	10.977	7.036	1.00	0.00	N
ATOM	1416	CA	ASP	93	-11.543	10.969	8.462	1.00	0.00	C
ATOM	1417	C	ASP	93	-12.413	12.166	8.838	1.00	0.00	C
ATOM	1418	O	ASP	93	-11.909	13.269	9.052	1.00	0.00	O
ATOM	1419	CB	ASP	93	-10.251	10.985	9.281	1.00	0.00	C
ATOM	1420	CG	ASP	93	-10.455	10.467	10.691	1.00	0.00	C
ATOM	1421	OD1	ASP	93	-11.330	9.597	10.884	1.00	0.00	O
ATOM	1422	OD2	ASP	93	-9.740	10.931	11.603	1.00	0.00	O
ATOM	1423	H	ASP	93	-10.316	11.135	6.745	1.00	0.00	H
ATOM	1424	HA	ASP	93	-12.087	10.063	8.682	1.00	0.00	H
ATOM	1425	2HB3	ASP	93	-9.881	11.997	9.339	1.00	0.00	H
ATOM	1426	HB2	ASP	93	-9.510	10.343	8.806	1.00	0.00	H
ATOM	1427	N	LYS	94	-13.720	11.941	8.915	1.00	0.00	N
ATOM	1428	CA	LYS	94	-14.660	13.000	9.262	1.00	0.00	C
ATOM	1429	C	LYS	94	-14.490	13.418	10.720	1.00	0.00	C
ATOM	1430	O	LYS	94	-14.131	14.560	11.009	1.00	0.00	O
ATOM	1431	CB	LYS	94	-16.097	12.533	9.020	1.00	0.00	C
ATOM	1432	CG	LYS	94	-16.581	12.770	7.600	1.00	0.00	C
ATOM	1433	CD	LYS	94	-18.084	12.984	7.551	1.00	0.00	C
ATOM	1434	CE	LYS	94	-18.827	11.668	7.358	1.00	0.00	C
ATOM	1435	NZ	LYS	94	-20.220	11.884	6.877	1.00	0.00	N
ATOM	1436	H	LYS	94	-14.060	11.040	8.730	1.00	0.00	H
ATOM	1437	HA	LYS	94	-14.454	13.849	8.631	1.00	0.00	H
ATOM	1438	2HB3	LYS	94	-16.753	13.065	9.694	1.00	0.00	H
ATOM	1439	2HG3	LYS	94	-16.328	11.909	6.996	1.00	0.00	H
ATOM	1440	2HD3	LYS	94	-18.319	13.642	6.727	1.00	0.00	H
ATOM	1441	2HE3	LYS	94	-18.857	11.146	8.304	1.00	0.00	H
ATOM	1442	1HZ	LYS	94	-20.289	11.652	5.866	1.00	0.00	H
ATOM	1443	2HZ	LYS	94	-20.494	12.879	7.013	1.00	0.00	H
ATOM	1444	3HZ	LYS	94	-20.880	11.279	7.407	1.00	0.00	H
ATOM	1445	HB2	LYS	94	-16.166	11.459	9.192	1.00	0.00	H
ATOM	1446	HG2	LYS	94	-16.102	13.663	7.195	1.00	0.00	H
ATOM	1447	HD2	LYS	94	-18.418	13.426	8.489	1.00	0.00	H
ATOM	1448	HE2	LYS	94	-18.311	11.066	6.610	1.00	0.00	H
ATOM	1449	N	ASP	95	-14.748	12.488	11.632	1.00	0.00	N
ATOM	1450	CA	ASP	95	-14.621	12.760	13.059	1.00	0.00	C
ATOM	1451	C	ASP	95	-13.252	13.357	13.379	1.00	0.00	C
ATOM	1452	O	ASP	95	-13.144	14.312	14.146	1.00	0.00	O
ATOM	1453	CB	ASP	95	-14.836	11.478	13.865	1.00	0.00	C

ATOM	1454	CG	ASP	95	-13.763	10.442	13.601	1.00	0.00	C
ATOM	1455	OD1	ASP	95	-13.230	10.416	12.471	1.00	0.00	O
ATOM	1456	OD2	ASP	95	-13.457	9.655	14.521	1.00	0.00	O
ATOM	1457	H	ASP	95	-15.030	11.597	11.339	1.00	0.00	H
ATOM	1458	HA	ASP	95	-15.383	13.476	13.329	1.00	0.00	H
ATOM	1459	2HB3	ASP	95	-15.793	11.056	13.607	1.00	0.00	H
ATOM	1460	HB2	ASP	95	-14.803	11.709	14.931	1.00	0.00	H
ATOM	1461	N	GLY	96	-12.210	12.780	12.787	1.00	0.00	N
ATOM	1462	CA	GLY	96	-10.864	13.266	13.022	1.00	0.00	C
ATOM	1463	C	GLY	96	-10.235	12.657	14.260	1.00	0.00	C
ATOM	1464	O	GLY	96	-9.627	13.360	15.066	1.00	0.00	O
ATOM	1465	H	GLY	96	-12.357	12.022	12.186	1.00	0.00	H
ATOM	1466	2HA	GLY	96	-10.251	13.029	12.166	1.00	0.00	H
ATOM	1467	3HA	GLY	96	-10.895	14.339	13.142	1.00	0.00	H
ATOM	1468	N	ASN	97	-10.383	11.346	14.410	1.00	0.00	N
ATOM	1469	CA	ASN	97	-9.827	10.640	15.561	1.00	0.00	C
ATOM	1470	C	ASN	97	-8.566	9.873	15.170	1.00	0.00	C
ATOM	1471	O	ASN	97	-7.564	9.903	15.883	1.00	0.00	O
ATOM	1472	CB	ASN	97	-10.863	9.679	16.145	1.00	0.00	C
ATOM	1473	CG	ASN	97	-10.980	8.396	15.344	1.00	0.00	C
ATOM	1474	OD1	ASN	97	-11.354	8.418	14.172	1.00	0.00	O
ATOM	1475	ND2	ASN	97	-10.659	7.273	15.975	1.00	0.00	N
ATOM	1476	H	ASN	97	-10.878	10.838	13.734	1.00	0.00	H
ATOM	1477	HA	ASN	97	-9.569	11.376	16.307	1.00	0.00	H
ATOM	1478	2HB3	ASN	97	-11.828	10.163	16.159	1.00	0.00	H
ATOM	1479	1HD2	ASN	97	-10.369	7.333	16.909	1.00	0.00	H
ATOM	1480	2HD2	ASN	97	-10.726	6.428	15.480	1.00	0.00	H
ATOM	1481	HB2	ASN	97	-10.571	9.400	17.157	1.00	0.00	H
ATOM	1482	N	GLY	98	-8.626	9.186	14.033	1.00	0.00	N
ATOM	1483	CA	GLY	98	-7.485	8.420	13.569	1.00	0.00	C
ATOM	1484	C	GLY	98	-7.884	7.282	12.652	1.00	0.00	C
ATOM	1485	O	GLY	98	-7.218	7.018	11.651	1.00	0.00	O
ATOM	1486	H	GLY	98	-9.454	9.198	13.508	1.00	0.00	H
ATOM	1487	2HA	GLY	98	-6.816	9.080	13.036	1.00	0.00	H
ATOM	1488	3HA	GLY	98	-6.967	8.014	14.425	1.00	0.00	H
ATOM	1489	N	TYR	99	-8.974	6.604	12.993	1.00	0.00	N
ATOM	1490	CA	TYR	99	-9.461	5.486	12.195	1.00	0.00	C
ATOM	1491	C	TYR	99	-10.619	5.916	11.300	1.00	0.00	C
ATOM	1492	O	TYR	99	-11.532	6.614	11.740	1.00	0.00	O
ATOM	1493	CB	TYR	99	-9.904	4.339	13.104	1.00	0.00	C
ATOM	1494	CG	TYR	99	-8.752	3.571	13.716	1.00	0.00	C
ATOM	1495	CD1	TYR	99	-7.812	4.211	14.513	1.00	0.00	C
ATOM	1496	CE1	TYR	99	-6.758	3.515	15.071	1.00	0.00	C
ATOM	1497	CZ	TYR	99	-6.638	2.160	14.842	1.00	0.00	C
ATOM	1498	CE2	TYR	99	-7.560	1.502	14.056	1.00	0.00	C
ATOM	1499	CD2	TYR	99	-8.608	2.207	13.499	1.00	0.00	C
ATOM	1500	OH	TYR	99	-5.589	1.462	15.400	1.00	0.00	O
ATOM	1501	H	TYR	99	-9.465	6.862	13.800	1.00	0.00	H
ATOM	1502	HA	TYR	99	-8.647	5.144	11.573	1.00	0.00	H
ATOM	1503	2HB3	TYR	99	-10.497	3.642	12.530	1.00	0.00	H
ATOM	1504	1HD	TYR	99	-7.911	5.272	14.689	1.00	0.00	H
ATOM	1505	1HE	TYR	99	-6.035	4.030	15.686	1.00	0.00	H
ATOM	1506	2HE	TYR	99	-7.464	0.441	13.877	1.00	0.00	H
ATOM	1507	2HD	TYR	99	-9.333	1.693	12.883	1.00	0.00	H
ATOM	1508	HH	TYR	99	-4.780	1.971	15.306	1.00	0.00	H
ATOM	1509	HB2	TYR	99	-10.491	4.734	13.932	1.00	0.00	H
ATOM	1510	N	ILE	100	-10.572	5.494	10.039	1.00	0.00	N
ATOM	1511	CA	ILE	100	-11.617	5.834	9.082	1.00	0.00	C
ATOM	1512	C	ILE	100	-12.621	4.697	8.938	1.00	0.00	C
ATOM	1513	O	ILE	100	-12.378	3.727	8.218	1.00	0.00	O
ATOM	1514	CB	ILE	100	-11.027	6.165	7.698	1.00	0.00	C
ATOM	1515	CG2	ILE	100	-12.141	6.477	6.710	1.00	0.00	C
ATOM	1516	CG1	ILE	100	-10.052	7.337	7.801	1.00	0.00	C
ATOM	1517	CD1	ILE	100	-9.156	7.488	6.591	1.00	0.00	C
ATOM	1518	H	ILE	100	-9.819	4.942	9.749	1.00	0.00	H
ATOM	1519	HA	ILE	100	-12.133	6.712	9.450	1.00	0.00	H
ATOM	1520	HB	ILE	100	-10.496	5.294	7.343	1.00	0.00	H
ATOM	1521	1HG2	ILE	100	-11.719	6.935	5.825	1.00	0.00	H
ATOM	1522	2HG2	ILE	100	-12.646	5.564	6.434	1.00	0.00	H
ATOM	1523	3HG2	ILE	100	-12.846	7.156	7.165	1.00	0.00	H
ATOM	1524	2HG3	ILE	100	-9.422	7.197	8.666	1.00	0.00	H
ATOM	1525	1HD1	ILE	100	-9.002	8.538	6.384	1.00	0.00	H
ATOM	1526	2HD1	ILE	100	-8.206	7.013	6.785	1.00	0.00	H
ATOM	1527	3HD1	ILE	100	-9.625	7.019	5.738	1.00	0.00	H
ATOM	1528	HG2	ILE	100	-10.612	8.267	7.897	1.00	0.00	H
ATOM	1529	N	SER	101	-13.754	4.821	9.625	1.00	0.00	N
ATOM	1530	CA	SER	101	-14.795	3.802	9.573	1.00	0.00	C

ATOM	1531	C	SER	101	-15.385	3.697	8.171	1.00	0.00	C
ATOM	1532	O	SER	101	-15.146	4.555	7.319	1.00	0.00	O
ATOM	1533	CB	SER	101	-15.900	4.123	10.581	1.00	0.00	C
ATOM	1534	OG	SER	101	-16.464	5.399	10.331	1.00	0.00	O
ATOM	1535	H	SER	101	-13.890	5.619	10.178	1.00	0.00	H
ATOM	1536	HA	SER	101	-14.346	2.855	9.833	1.00	0.00	H
ATOM	1537	2HB3	SER	101	-15.488	4.114	11.580	1.00	0.00	H
ATOM	1538	HG	SER	101	-15.811	6.078	10.507	1.00	0.00	H
ATOM	1539	HB2	SER	101	-16.689	3.375	10.505	1.00	0.00	H
ATOM	1540	N	ALA	102	-16.155	2.639	7.937	1.00	0.00	N
ATOM	1541	CA	ALA	102	-16.780	2.422	6.638	1.00	0.00	C
ATOM	1542	C	ALA	102	-17.735	3.559	6.293	1.00	0.00	C
ATOM	1543	O	ALA	102	-17.754	4.044	5.162	1.00	0.00	O
ATOM	1544	CB	ALA	102	-17.516	1.090	6.622	1.00	0.00	C
ATOM	1545	H	ALA	102	-16.307	1.991	8.654	1.00	0.00	H
ATOM	1546	HA	ALA	102	-15.998	2.384	5.894	1.00	0.00	H
ATOM	1547	1HB	ALA	102	-16.833	0.307	6.321	1.00	0.00	H
ATOM	1548	2HB	ALA	102	-17.894	0.877	7.613	1.00	0.00	H
ATOM	1549	3HB	ALA	102	-18.336	1.141	5.925	1.00	0.00	H
ATOM	1550	N	ALA	103	-18.528	3.981	7.273	1.00	0.00	N
ATOM	1551	CA	ALA	103	-19.485	5.061	7.072	1.00	0.00	C
ATOM	1552	C	ALA	103	-18.773	6.373	6.750	1.00	0.00	C
ATOM	1553	O	ALA	103	-19.124	7.060	5.792	1.00	0.00	O
ATOM	1554	CB	ALA	103	-20.364	5.224	8.304	1.00	0.00	C
ATOM	1555	H	ALA	103	-18.465	3.554	8.154	1.00	0.00	H
ATOM	1556	HA	ALA	103	-20.118	4.794	6.239	1.00	0.00	H
ATOM	1557	1HB	ALA	103	-21.012	6.078	8.170	1.00	0.00	H
ATOM	1558	2HB	ALA	103	-20.958	4.335	8.440	1.00	0.00	H
ATOM	1559	3HB	ALA	103	-19.740	5.378	9.171	1.00	0.00	H
ATOM	1560	N	GLU	104	-17.771	6.711	7.558	1.00	0.00	N
ATOM	1561	CA	GLU	104	-17.013	7.939	7.358	1.00	0.00	C
ATOM	1562	C	GLU	104	-16.501	8.036	5.925	1.00	0.00	C
ATOM	1563	O	GLU	104	-16.770	9.010	5.220	1.00	0.00	O
ATOM	1564	CB	GLU	104	-15.838	8.004	8.336	1.00	0.00	C
ATOM	1565	CG	GLU	104	-16.223	8.510	9.716	1.00	0.00	C
ATOM	1566	CD	GLU	104	-15.020	8.732	10.612	1.00	0.00	C
ATOM	1567	OE1	GLU	104	-14.063	9.400	10.165	1.00	0.00	O
ATOM	1568	OE2	GLU	104	-15.034	8.240	11.758	1.00	0.00	O
ATOM	1569	H	GLU	104	-17.540	6.121	8.303	1.00	0.00	H
ATOM	1570	HA	GLU	104	-17.673	8.772	7.549	1.00	0.00	H
ATOM	1571	2HB3	GLU	104	-15.086	8.665	7.931	1.00	0.00	H
ATOM	1572	2HG3	GLU	104	-16.872	7.784	10.183	1.00	0.00	H
ATOM	1573	HB2	GLU	104	-15.427	7.004	8.477	1.00	0.00	H
ATOM	1574	HG2	GLU	104	-16.733	9.468	9.623	1.00	0.00	H
ATOM	1575	N	LEU	105	-15.762	7.020	5.497	1.00	0.00	N
ATOM	1576	CA	LEU	105	-15.209	6.986	4.146	1.00	0.00	C
ATOM	1577	C	LEU	105	-16.321	6.888	3.106	1.00	0.00	C
ATOM	1578	O	LEU	105	-16.315	7.606	2.105	1.00	0.00	O
ATOM	1579	CB	LEU	105	-14.247	5.810	3.993	1.00	0.00	C
ATOM	1580	CG	LEU	105	-13.833	5.461	2.564	1.00	0.00	C
ATOM	1581	CD1	LEU	105	-12.439	4.852	2.546	1.00	0.00	C
ATOM	1582	CD2	LEU	105	-14.841	4.511	1.933	1.00	0.00	C
ATOM	1583	H	LEU	105	-15.580	6.270	6.102	1.00	0.00	H
ATOM	1584	HA	LEU	105	-14.667	7.907	3.988	1.00	0.00	H
ATOM	1585	2HB3	LEU	105	-14.721	4.937	4.424	1.00	0.00	H
ATOM	1586	HG	LEU	105	-13.808	6.366	1.973	1.00	0.00	H
ATOM	1587	1HD1	LEU	105	-11.763	5.515	2.028	1.00	0.00	H
ATOM	1588	2HD1	LEU	105	-12.471	3.901	2.034	1.00	0.00	H
ATOM	1589	3HD1	LEU	105	-12.097	4.705	3.559	1.00	0.00	H
ATOM	1590	1HD2	LEU	105	-14.368	3.969	1.129	1.00	0.00	H
ATOM	1591	2HD2	LEU	105	-15.674	5.078	1.544	1.00	0.00	H
ATOM	1592	3HD2	LEU	105	-15.196	3.816	2.680	1.00	0.00	H
ATOM	1593	HB2	LEU	105	-13.309	6.039	4.499	1.00	0.00	H
ATOM	1594	N	ARG	106	-17.277	5.996	3.351	1.00	0.00	N
ATOM	1595	CA	ARG	106	-18.396	5.804	2.438	1.00	0.00	C
ATOM	1596	C	ARG	106	-18.931	7.146	1.944	1.00	0.00	C
ATOM	1597	O	ARG	106	-19.072	7.364	0.740	1.00	0.00	O
ATOM	1598	CB	ARG	106	-19.512	5.019	3.125	1.00	0.00	C
ATOM	1599	CG	ARG	106	-20.723	4.775	2.235	1.00	0.00	C
ATOM	1600	CD	ARG	106	-21.955	4.428	3.053	1.00	0.00	C
ATOM	1601	NE	ARG	106	-23.172	4.448	2.245	1.00	0.00	N
ATOM	1602	CZ	ARG	106	-24.275	3.779	2.563	1.00	0.00	C
ATOM	1603	NH1	ARG	106	-24.316	3.044	3.665	1.00	0.00	N
ATOM	1604	NH2	ARG	106	-25.343	3.850	1.778	1.00	0.00	N
ATOM	1605	H	ARG	106	-17.227	5.455	4.168	1.00	0.00	H
ATOM	1606	HA	ARG	106	-18.038	5.241	1.589	1.00	0.00	H
ATOM	1607	2HB3	ARG	106	-19.838	5.566	3.996	1.00	0.00	H

ATOM	1608	2HG3	ARG	106	-20.505	3.958	1.564	1.00	0.00	H
ATOM	1609	2HD3	ARG	106	-22.052	5.146	3.853	1.00	0.00	H
ATOM	1610	HE	ARG	106	-23.165	4.986	1.428	1.00	0.00	H
ATOM	1611	1HH1	ARG	106	-23.512	2.993	4.260	1.00	0.00	H
ATOM	1612	2HH1	ARG	106	-25.146	2.543	3.903	1.00	0.00	H
ATOM	1613	1HH2	ARG	106	-25.316	4.403	0.945	1.00	0.00	H
ATOM	1614	2HH2	ARG	106	-26.171	3.346	2.017	1.00	0.00	H
ATOM	1615	HB2	ARG	106	-19.141	4.035	3.412	1.00	0.00	H
ATOM	1616	HG2	ARG	106	-20.942	5.677	1.665	1.00	0.00	H
ATOM	1617	HD2	ARG	106	-21.850	3.422	3.460	1.00	0.00	H
ATOM	1618	N	HIS	107	-19.228	8.040	2.881	1.00	0.00	N
ATOM	1619	CA	HIS	107	-19.747	9.359	2.543	1.00	0.00	C
ATOM	1620	C	HIS	107	-18.791	10.096	1.612	1.00	0.00	C
ATOM	1621	O	HIS	107	-19.196	10.610	0.568	1.00	0.00	O
ATOM	1622	CB	HIS	107	-19.980	10.180	3.810	1.00	0.00	C
ATOM	1623	CG	HIS	107	-19.949	11.660	3.581	1.00	0.00	C
ATOM	1624	ND1	HIS	107	-20.757	12.296	2.661	1.00	0.00	N
ATOM	1625	CD2	HIS	107	-19.203	12.632	4.157	1.00	0.00	C
ATOM	1626	CE1	HIS	107	-20.507	13.593	2.681	1.00	0.00	C
ATOM	1627	NE2	HIS	107	-19.569	13.822	3.580	1.00	0.00	N
ATOM	1628	H	HIS	107	-19.094	7.806	3.824	1.00	0.00	H
ATOM	1629	HA	HIS	107	-20.689	9.223	2.035	1.00	0.00	H
ATOM	1630	2HB3	HIS	107	-19.212	9.941	4.533	1.00	0.00	H
ATOM	1631	1HD	HIS	107	-21.414	11.861	2.080	1.00	0.00	H
ATOM	1632	2HD	HIS	107	-18.458	12.494	4.927	1.00	0.00	H
ATOM	1633	1HE	HIS	107	-20.989	14.338	2.068	1.00	0.00	H
ATOM	1634	2HE	HIS	107	-19.137	14.687	3.739	1.00	0.00	H
ATOM	1635	HB2	HIS	107	-20.963	9.947	4.220	1.00	0.00	H
ATOM	1636	N	VAL	108	-17.519	10.150	1.997	1.00	0.00	N
ATOM	1637	CA	VAL	108	-16.504	10.825	1.198	1.00	0.00	C
ATOM	1638	C	VAL	108	-16.557	10.368	-0.257	1.00	0.00	C
ATOM	1639	O	VAL	108	-16.688	11.183	-1.170	1.00	0.00	O
ATOM	1640	CB	VAL	108	-15.092	10.570	1.752	1.00	0.00	C
ATOM	1641	CG1	VAL	108	-14.042	11.233	0.872	1.00	0.00	C
ATOM	1642	CG2	VAL	108	-14.986	11.068	3.188	1.00	0.00	C
ATOM	1643	H	VAL	108	-17.258	9.722	2.840	1.00	0.00	H
ATOM	1644	HA	VAL	108	-16.700	11.887	1.239	1.00	0.00	H
ATOM	1645	HB	VAL	108	-14.911	9.506	1.750	1.00	0.00	H
ATOM	1646	1HG1	VAL	108	-13.059	11.034	1.271	1.00	0.00	H
ATOM	1647	2HG1	VAL	108	-14.114	10.837	-0.131	1.00	0.00	H
ATOM	1648	3HG1	VAL	108	-14.211	12.298	0.852	1.00	0.00	H
ATOM	1649	1HG2	VAL	108	-14.568	10.289	3.808	1.00	0.00	H
ATOM	1650	2HG2	VAL	108	-14.344	11.937	3.221	1.00	0.00	H
ATOM	1651	3HG2	VAL	108	-15.967	11.331	3.550	1.00	0.00	H
ATOM	1652	N	MET	109	-16.453	9.060	-0.465	1.00	0.00	N
ATOM	1653	CA	MET	109	-16.492	8.495	-1.808	1.00	0.00	C
ATOM	1654	C	MET	109	-17.642	9.087	-2.615	1.00	0.00	C
ATOM	1655	O	MET	109	-17.469	9.464	-3.775	1.00	0.00	O
ATOM	1656	CB	MET	109	-16.632	6.973	-1.739	1.00	0.00	C
ATOM	1657	CG	MET	109	-15.406	6.273	-1.176	1.00	0.00	C
ATOM	1658	SD	MET	109	-14.208	5.835	-2.453	1.00	0.00	S
ATOM	1659	CE	MET	109	-12.934	5.054	-1.468	1.00	0.00	C
ATOM	1660	H	MET	109	-16.352	8.458	0.304	1.00	0.00	H
ATOM	1661	HA	MET	109	-15.561	8.739	-2.296	1.00	0.00	H
ATOM	1662	2HB3	MET	109	-16.810	6.595	-2.735	1.00	0.00	H
ATOM	1663	2HG3	MET	109	-15.722	5.370	-0.676	1.00	0.00	H
ATOM	1664	1HE	MET	109	-13.170	4.011	-1.333	1.00	0.00	H
ATOM	1665	2HE	MET	109	-11.981	5.147	-1.970	1.00	0.00	H
ATOM	1666	3HE	MET	109	-12.879	5.537	-0.501	1.00	0.00	H
ATOM	1667	HB2	MET	109	-17.463	6.713	-1.082	1.00	0.00	H
ATOM	1668	HG2	MET	109	-14.898	6.936	-0.477	1.00	0.00	H
ATOM	1669	N	THR	110	-18.815	9.167	-1.995	1.00	0.00	N
ATOM	1670	CA	THR	110	-19.993	9.714	-2.657	1.00	0.00	C
ATOM	1671	C	THR	110	-19.688	11.063	-3.296	1.00	0.00	C
ATOM	1672	O	THR	110	-19.880	11.249	-4.499	1.00	0.00	O
ATOM	1673	CB	THR	110	-21.165	9.878	-1.670	1.00	0.00	C
ATOM	1674	OG1	THR	110	-21.437	8.633	-1.019	1.00	0.00	O
ATOM	1675	CG2	THR	110	-22.414	10.365	-2.390	1.00	0.00	C
ATOM	1676	H	THR	110	-18.888	8.851	-1.070	1.00	0.00	H
ATOM	1677	HA	THR	110	-20.295	9.021	-3.429	1.00	0.00	H
ATOM	1678	HB	THR	110	-20.889	10.613	-0.926	1.00	0.00	H
ATOM	1679	1HG	THR	110	-20.722	8.426	-0.414	1.00	0.00	H
ATOM	1680	1HG2	THR	110	-22.262	11.379	-2.730	1.00	0.00	H
ATOM	1681	2HG2	THR	110	-23.253	10.333	-1.715	1.00	0.00	H
ATOM	1682	3HG2	THR	110	-22.609	9.727	-3.241	1.00	0.00	H
ATOM	1683	N	ASN	111	-19.213	12.004	-2.487	1.00	0.00	N
ATOM	1684	CA	ASN	111	-18.881	13.337	-2.976	1.00	0.00	C

ATOM	1685	C	ASN	111	-18.268	13.268	-4.370	1.00	0.00	C
ATOM	1686	O	ASN	111	-18.684	13.988	-5.280	1.00	0.00	O
ATOM	1687	CB	ASN	111	-17.911	14.028	-2.013	1.00	0.00	C
ATOM	1688	CG	ASN	111	-18.604	14.546	-0.769	1.00	0.00	C
ATOM	1689	OD1	ASN	111	-19.624	15.232	-0.851	1.00	0.00	O
ATOM	1690	ND2	ASN	111	-18.051	14.220	0.394	1.00	0.00	N
ATOM	1691	H	ASN	111	-19.081	11.796	-1.538	1.00	0.00	H
ATOM	1692	HA	ASN	111	-19.796	13.910	-3.024	1.00	0.00	H
ATOM	1693	2HB3	ASN	111	-17.446	14.862	-2.518	1.00	0.00	H
ATOM	1694	1HD2	ASN	111	-17.238	13.673	0.383	1.00	0.00	H
ATOM	1695	2HD2	ASN	111	-18.478	14.542	1.215	1.00	0.00	H
ATOM	1696	HB2	ASN	111	-17.150	13.317	-1.692	1.00	0.00	H
ATOM	1697	N	LEU	112	-17.278	12.397	-4.534	1.00	0.00	N
ATOM	1698	CA	LEU	112	-16.607	12.233	-5.820	1.00	0.00	C
ATOM	1699	C	LEU	112	-17.605	12.331	-6.970	1.00	0.00	C
ATOM	1700	O	LEU	112	-17.349	12.999	-7.971	1.00	0.00	O
ATOM	1701	CB	LEU	112	-15.883	10.888	-5.870	1.00	0.00	C
ATOM	1702	CG	LEU	112	-14.748	10.697	-4.865	1.00	0.00	C
ATOM	1703	CD1	LEU	112	-14.191	9.284	-4.948	1.00	0.00	C
ATOM	1704	CD2	LEU	112	-13.647	11.720	-5.103	1.00	0.00	C
ATOM	1705	H	LEU	112	-16.992	11.851	-3.775	1.00	0.00	H
ATOM	1706	HA	LEU	112	-15.883	13.027	-5.921	1.00	0.00	H
ATOM	1707	2HB3	LEU	112	-15.471	10.773	-6.863	1.00	0.00	H
ATOM	1708	HG	LEU	112	-15.132	10.846	-3.866	1.00	0.00	H
ATOM	1709	1HD1	LEU	112	-13.380	9.174	-4.243	1.00	0.00	H
ATOM	1710	2HD1	LEU	112	-13.827	9.100	-5.948	1.00	0.00	H
ATOM	1711	3HD1	LEU	112	-14.972	8.576	-4.713	1.00	0.00	H
ATOM	1712	1HD2	LEU	112	-12.684	11.239	-5.027	1.00	0.00	H
ATOM	1713	2HD2	LEU	112	-13.716	12.504	-4.361	1.00	0.00	H
ATOM	1714	3HD2	LEU	112	-13.760	12.148	-6.089	1.00	0.00	H
ATOM	1715	HB2	LEU	112	-16.586	10.087	-5.641	1.00	0.00	H
ATOM	1716	N	GLY	113	-18.743	11.661	-6.819	1.00	0.00	N
ATOM	1717	CA	GLY	113	-19.764	11.687	-7.852	1.00	0.00	C
ATOM	1718	C	GLY	113	-20.317	10.310	-8.154	1.00	0.00	C
ATOM	1719	O	GLY	113	-20.529	9.961	-9.315	1.00	0.00	O
ATOM	1720	H	GLY	113	-18.891	11.145	-6.000	1.00	0.00	H
ATOM	1721	2HA	GLY	113	-20.572	12.326	-7.528	1.00	0.00	H
ATOM	1722	3HA	GLY	113	-19.333	12.096	-8.756	1.00	0.00	H
ATOM	1723	N	GLU	114	-20.551	9.524	-7.108	1.00	0.00	N
ATOM	1724	CA	GLU	114	-21.080	8.176	-7.268	1.00	0.00	C
ATOM	1725	C	GLU	114	-21.887	7.758	-6.043	1.00	0.00	C
ATOM	1726	O	GLU	114	-21.803	8.383	-4.986	1.00	0.00	O
ATOM	1727	CB	GLU	114	-19.944	7.180	-7.507	1.00	0.00	C
ATOM	1728	CG	GLU	114	-19.186	7.420	-8.802	1.00	0.00	C
ATOM	1729	CD	GLU	114	-20.000	7.060	-10.029	1.00	0.00	C
ATOM	1730	OE1	GLU	114	-20.951	6.261	-9.895	1.00	0.00	O
ATOM	1731	OE2	GLU	114	-19.686	7.573	-11.123	1.00	0.00	O
ATOM	1732	H	GLU	114	-20.361	9.859	-6.207	1.00	0.00	H
ATOM	1733	HA	GLU	114	-21.733	8.177	-8.130	1.00	0.00	H
ATOM	1734	2HB3	GLU	114	-20.355	6.183	-7.534	1.00	0.00	H
ATOM	1735	2HG3	GLU	114	-18.288	6.822	-8.794	1.00	0.00	H
ATOM	1736	HB2	GLU	114	-19.210	7.266	-6.704	1.00	0.00	H
ATOM	1737	HG2	GLU	114	-18.934	8.478	-8.886	1.00	0.00	H
ATOM	1738	N	LYS	115	-22.674	6.698	-6.191	1.00	0.00	N
ATOM	1739	CA	LYS	115	-23.496	6.193	-5.097	1.00	0.00	C
ATOM	1740	C	LYS	115	-23.103	4.765	-4.735	1.00	0.00	C
ATOM	1741	O	LYS	115	-23.482	3.813	-5.416	1.00	0.00	O
ATOM	1742	CB	LYS	115	-24.975	6.244	-5.481	1.00	0.00	C
ATOM	1743	CG	LYS	115	-25.536	7.654	-5.560	1.00	0.00	C
ATOM	1744	CD	LYS	115	-26.741	7.726	-6.483	1.00	0.00	C
ATOM	1745	CE	LYS	115	-27.444	9.072	-6.379	1.00	0.00	C
ATOM	1746	NZ	LYS	115	-26.867	10.070	-7.321	1.00	0.00	N
ATOM	1747	H	LYS	115	-22.700	6.240	-7.059	1.00	0.00	H
ATOM	1748	HA	LYS	115	-23.333	6.828	-4.241	1.00	0.00	H
ATOM	1749	2HB3	LYS	115	-25.546	5.694	-4.746	1.00	0.00	H
ATOM	1750	2HG3	LYS	115	-24.768	8.317	-5.932	1.00	0.00	H
ATOM	1751	2HD3	LYS	115	-27.437	6.944	-6.211	1.00	0.00	H
ATOM	1752	2HE3	LYS	115	-27.341	9.441	-5.369	1.00	0.00	H
ATOM	1753	1HZ	LYS	115	-27.418	10.089	-8.203	1.00	0.00	H
ATOM	1754	2HZ	LYS	115	-25.882	9.824	-7.547	1.00	0.00	H
ATOM	1755	3HZ	LYS	115	-26.887	11.018	-6.892	1.00	0.00	H
ATOM	1756	HB2	LYS	115	-25.111	5.808	-6.471	1.00	0.00	H
ATOM	1757	HG2	LYS	115	-25.855	7.975	-4.568	1.00	0.00	H
ATOM	1758	HD2	LYS	115	-26.417	7.596	-7.514	1.00	0.00	H
ATOM	1759	HE2	LYS	115	-28.496	8.953	-6.632	1.00	0.00	H
ATOM	1760	N	LEU	116	-22.342	4.622	-3.655	1.00	0.00	N
ATOM	1761	CA	LEU	116	-21.899	3.310	-3.199	1.00	0.00	C

ATOM	1762	C	LEU	116	-22.714	2.848	-1.995	1.00	0.00	C
ATOM	1763	O	LEU	116	-23.336	3.655	-1.305	1.00	0.00	O
ATOM	1764	CB	LEU	116	-20.414	3.348	-2.838	1.00	0.00	C
ATOM	1765	CG	LEU	116	-19.476	3.902	-3.912	1.00	0.00	C
ATOM	1766	CD1	LEU	116	-18.158	4.344	-3.295	1.00	0.00	C
ATOM	1767	CD2	LEU	116	-19.236	2.866	-5.001	1.00	0.00	C
ATOM	1768	H	LEU	116	-22.072	5.418	-3.150	1.00	0.00	H
ATOM	1769	HA	LEU	116	-22.048	2.610	-4.009	1.00	0.00	H
ATOM	1770	2HB3	LEU	116	-20.103	2.337	-2.617	1.00	0.00	H
ATOM	1771	HG	LEU	116	-19.936	4.769	-4.369	1.00	0.00	H
ATOM	1772	1HD1	LEU	116	-18.322	5.221	-2.687	1.00	0.00	H
ATOM	1773	2HD1	LEU	116	-17.454	4.578	-4.078	1.00	0.00	H
ATOM	1774	3HD1	LEU	116	-17.764	3.547	-2.682	1.00	0.00	H
ATOM	1775	1HD2	LEU	116	-18.944	1.931	-4.546	1.00	0.00	H
ATOM	1776	2HD2	LEU	116	-18.450	3.209	-5.656	1.00	0.00	H
ATOM	1777	3HD2	LEU	116	-20.145	2.723	-5.568	1.00	0.00	H
ATOM	1778	HB2	LEU	116	-20.264	4.002	-1.979	1.00	0.00	H
ATOM	1779	N	THR	117	-22.706	1.541	-1.748	1.00	0.00	N
ATOM	1780	CA	THR	117	-23.442	0.971	-0.627	1.00	0.00	C
ATOM	1781	C	THR	117	-22.516	0.192	0.301	1.00	0.00	C
ATOM	1782	O	THR	117	-21.411	-0.187	-0.084	1.00	0.00	O
ATOM	1783	CB	THR	117	-24.568	0.038	-1.111	1.00	0.00	C
ATOM	1784	OG1	THR	117	-24.035	-0.954	-1.997	1.00	0.00	O
ATOM	1785	CG2	THR	117	-25.657	0.829	-1.821	1.00	0.00	C
ATOM	1786	H	THR	117	-22.192	0.947	-2.333	1.00	0.00	H
ATOM	1787	HA	THR	117	-23.889	1.783	-0.073	1.00	0.00	H
ATOM	1788	HB	THR	117	-25.003	-0.452	-0.251	1.00	0.00	H
ATOM	1789	1HG	THR	117	-24.671	-1.666	-2.096	1.00	0.00	H
ATOM	1790	1HG2	THR	117	-25.694	1.830	-1.420	1.00	0.00	H
ATOM	1791	2HG2	THR	117	-26.610	0.344	-1.669	1.00	0.00	H
ATOM	1792	3HG2	THR	117	-25.440	0.870	-2.877	1.00	0.00	H
ATOM	1793	N	ASP	118	-22.974	-0.043	1.526	1.00	0.00	N
ATOM	1794	CA	ASP	118	-22.188	-0.779	2.509	1.00	0.00	C
ATOM	1795	C	ASP	118	-21.363	-1.872	1.836	1.00	0.00	C
ATOM	1796	O	ASP	118	-20.193	-2.065	2.157	1.00	0.00	O
ATOM	1797	CB	ASP	118	-23.102	-1.392	3.571	1.00	0.00	C
ATOM	1798	CG	ASP	118	-23.366	-0.447	4.723	1.00	0.00	C
ATOM	1799	OD1	ASP	118	-23.257	0.783	4.522	1.00	0.00	O
ATOM	1800	OD2	ASP	118	-23.683	-0.933	5.829	1.00	0.00	O
ATOM	1801	H	ASP	118	-23.865	0.285	1.775	1.00	0.00	H
ATOM	1802	HA	ASP	118	-21.518	-0.081	2.985	1.00	0.00	H
ATOM	1803	2HB3	ASP	118	-22.640	-2.289	3.960	1.00	0.00	H
ATOM	1804	HB2	ASP	118	-24.068	-1.632	3.125	1.00	0.00	H
ATOM	1805	N	GLU	119	-21.985	-2.585	0.903	1.00	0.00	N
ATOM	1806	CA	GLU	119	-21.308	-3.661	0.184	1.00	0.00	C
ATOM	1807	C	GLU	119	-20.074	-3.136	-0.540	1.00	0.00	C
ATOM	1808	O	GLU	119	-18.958	-3.606	-0.307	1.00	0.00	O
ATOM	1809	CB	GLU	119	-22.263	-4.316	-0.815	1.00	0.00	C
ATOM	1810	CG	GLU	119	-23.413	-5.060	-0.160	1.00	0.00	C
ATOM	1811	CD	GLU	119	-22.950	-6.251	0.657	1.00	0.00	C
ATOM	1812	OE1	GLU	119	-22.144	-7.051	0.134	1.00	0.00	O
ATOM	1813	OE2	GLU	119	-23.391	-6.383	1.818	1.00	0.00	O
ATOM	1814	H	GLU	119	-22.920	-2.385	0.690	1.00	0.00	H
ATOM	1815	HA	GLU	119	-20.997	-4.397	0.912	1.00	0.00	H
ATOM	1816	2HB3	GLU	119	-21.705	-5.018	-1.416	1.00	0.00	H
ATOM	1817	2HG3	GLU	119	-24.084	-5.410	-0.930	1.00	0.00	H
ATOM	1818	HB2	GLU	119	-22.712	-3.549	-1.444	1.00	0.00	H
ATOM	1819	HG2	GLU	119	-23.938	-4.391	0.521	1.00	0.00	H
ATOM	1820	N	GLU	120	-20.279	-2.163	-1.422	1.00	0.00	N
ATOM	1821	CA	GLU	120	-19.184	-1.577	-2.183	1.00	0.00	C
ATOM	1822	C	GLU	120	-18.034	-1.176	-1.263	1.00	0.00	C
ATOM	1823	O	GLU	120	-16.886	-1.568	-1.476	1.00	0.00	O
ATOM	1824	CB	GLU	120	-19.671	-0.358	-2.968	1.00	0.00	C
ATOM	1825	CG	GLU	120	-20.752	-0.682	-3.985	1.00	0.00	C
ATOM	1826	CD	GLU	120	-20.465	-1.956	-4.756	1.00	0.00	C
ATOM	1827	OE1	GLU	120	-20.771	-3.047	-4.227	1.00	0.00	O
ATOM	1828	OE2	GLU	120	-19.935	-1.865	-5.882	1.00	0.00	O
ATOM	1829	H	GLU	120	-21.191	-1.833	-1.563	1.00	0.00	H
ATOM	1830	HA	GLU	120	-18.827	-2.322	-2.879	1.00	0.00	H
ATOM	1831	2HB3	GLU	120	-18.832	0.079	-3.489	1.00	0.00	H
ATOM	1832	2HG3	GLU	120	-20.823	0.137	-4.689	1.00	0.00	H
ATOM	1833	HB2	GLU	120	-20.108	0.365	-2.279	1.00	0.00	H
ATOM	1834	HG2	GLU	120	-21.704	-0.822	-3.475	1.00	0.00	H
ATOM	1835	N	VAL	121	-18.352	-0.394	-0.235	1.00	0.00	N
ATOM	1836	CA	VAL	121	-17.347	0.060	0.717	1.00	0.00	C
ATOM	1837	C	VAL	121	-16.675	-1.119	1.412	1.00	0.00	C
ATOM	1838	O	VAL	121	-15.458	-1.139	1.589	1.00	0.00	O

ATOM	1839	CB	VAL	121	-17.962	0.986	1.784	1.00	0.00	C
ATOM	1840	CG1	VAL	121	-16.926	1.355	2.834	1.00	0.00	C
ATOM	1841	CG2	VAL	121	-18.544	2.231	1.133	1.00	0.00	C
ATOM	1842	H	VAL	121	-19.283	-0.115	-0.117	1.00	0.00	H
ATOM	1843	HA	VAL	121	-16.598	0.618	0.174	1.00	0.00	H
ATOM	1844	HB	VAL	121	-18.765	0.453	2.272	1.00	0.00	H
ATOM	1845	1HG1	VAL	121	-16.954	2.421	3.006	1.00	0.00	H
ATOM	1846	2HG1	VAL	121	-17.144	0.834	3.756	1.00	0.00	H
ATOM	1847	3HG1	VAL	121	-15.943	1.072	2.484	1.00	0.00	H
ATOM	1848	1HG2	VAL	121	-17.982	2.467	0.242	1.00	0.00	H
ATOM	1849	2HG2	VAL	121	-19.577	2.055	0.871	1.00	0.00	H
ATOM	1850	3HG2	VAL	121	-18.485	3.060	1.825	1.00	0.00	H
ATOM	1851	N	ASP	122	-17.479	-2.103	1.803	1.00	0.00	N
ATOM	1852	CA	ASP	122	-16.963	-3.290	2.477	1.00	0.00	C
ATOM	1853	C	ASP	122	-15.775	-3.871	1.719	1.00	0.00	C
ATOM	1854	O	ASP	122	-14.746	-4.197	2.314	1.00	0.00	O
ATOM	1855	CB	ASP	122	-18.062	-4.345	2.613	1.00	0.00	C
ATOM	1856	CG	ASP	122	-18.844	-4.203	3.905	1.00	0.00	C
ATOM	1857	OD1	ASP	122	-18.262	-3.722	4.899	1.00	0.00	O
ATOM	1858	OD2	ASP	122	-20.036	-4.574	3.922	1.00	0.00	O
ATOM	1859	H	ASP	122	-18.441	-2.031	1.634	1.00	0.00	H
ATOM	1860	HA	ASP	122	-16.636	-2.994	3.463	1.00	0.00	H
ATOM	1861	2HB3	ASP	122	-17.615	-5.326	2.590	1.00	0.00	H
ATOM	1862	HB2	ASP	122	-18.772	-4.238	1.794	1.00	0.00	H
ATOM	1863	N	GLU	123	-15.923	-4.002	0.405	1.00	0.00	N
ATOM	1864	CA	GLU	123	-14.862	-4.546	-0.434	1.00	0.00	C
ATOM	1865	C	GLU	123	-13.599	-3.694	-0.334	1.00	0.00	C
ATOM	1866	O	GLU	123	-12.484	-4.202	-0.469	1.00	0.00	O
ATOM	1867	CB	GLU	123	-15.321	-4.627	-1.890	1.00	0.00	C
ATOM	1868	CG	GLU	123	-16.564	-5.478	-2.090	1.00	0.00	C
ATOM	1869	CD	GLU	123	-16.794	-5.844	-3.543	1.00	0.00	C
ATOM	1870	OE1	GLU	123	-15.799	-6.063	-4.263	1.00	0.00	O
ATOM	1871	OE2	GLU	123	-17.970	-5.910	-3.961	1.00	0.00	O
ATOM	1872	H	GLU	123	-16.766	-3.725	-0.011	1.00	0.00	H
ATOM	1873	HA	GLU	123	-14.638	-5.543	-0.080	1.00	0.00	H
ATOM	1874	2HB3	GLU	123	-14.523	-5.048	-2.484	1.00	0.00	H
ATOM	1875	2HG3	GLU	123	-17.423	-4.930	-1.731	1.00	0.00	H
ATOM	1876	HB2	GLU	123	-15.573	-3.629	-2.249	1.00	0.00	H
ATOM	1877	HG2	GLU	123	-16.457	-6.414	-1.539	1.00	0.00	H
ATOM	1878	N	MET	124	-13.783	-2.401	-0.096	1.00	0.00	N
ATOM	1879	CA	MET	124	-12.657	-1.478	0.020	1.00	0.00	C
ATOM	1880	C	MET	124	-11.961	-1.641	1.368	1.00	0.00	C
ATOM	1881	O	MET	124	-10.737	-1.759	1.434	1.00	0.00	O
ATOM	1882	CB	MET	124	-13.135	-0.036	-0.152	1.00	0.00	C
ATOM	1883	CG	MET	124	-12.072	0.999	0.181	1.00	0.00	C
ATOM	1884	SD	MET	124	-12.315	2.554	-0.700	1.00	0.00	S
ATOM	1885	CE	MET	124	-11.493	2.197	-2.249	1.00	0.00	C
ATOM	1886	H	MET	124	-14.695	-2.056	0.000	1.00	0.00	H
ATOM	1887	HA	MET	124	-11.956	-1.712	-0.766	1.00	0.00	H
ATOM	1888	2HB3	MET	124	-13.983	0.131	0.496	1.00	0.00	H
ATOM	1889	2HG3	MET	124	-11.104	0.598	-0.084	1.00	0.00	H
ATOM	1890	1HE	MET	124	-10.814	3.003	-2.490	1.00	0.00	H
ATOM	1891	2HE	MET	124	-10.939	1.275	-2.160	1.00	0.00	H
ATOM	1892	3HE	MET	124	-12.230	2.101	-3.034	1.00	0.00	H
ATOM	1893	HB2	MET	124	-13.417	0.134	-1.191	1.00	0.00	H
ATOM	1894	HG2	MET	124	-12.109	1.229	1.247	1.00	0.00	H
ATOM	1895	N	ILE	125	-12.747	-1.645	2.437	1.00	0.00	N
ATOM	1896	CA	ILE	125	-12.205	-1.791	3.783	1.00	0.00	C
ATOM	1897	C	ILE	125	-11.299	-3.014	3.880	1.00	0.00	C
ATOM	1898	O	ILE	125	-10.201	-2.944	4.428	1.00	0.00	O
ATOM	1899	CB	ILE	125	-13.326	-1.914	4.832	1.00	0.00	C
ATOM	1900	CG2	ILE	125	-12.742	-2.255	6.193	1.00	0.00	C
ATOM	1901	CG1	ILE	125	-14.134	-0.617	4.901	1.00	0.00	C
ATOM	1902	CD1	ILE	125	-13.355	0.550	5.469	1.00	0.00	C
ATOM	1903	H	ILE	125	-13.716	-1.546	2.320	1.00	0.00	H
ATOM	1904	HA	ILE	125	-11.625	-0.908	4.006	1.00	0.00	H
ATOM	1905	HB	ILE	125	-13.980	-2.721	4.535	1.00	0.00	H
ATOM	1906	1HG2	ILE	125	-12.214	-1.397	6.583	1.00	0.00	H
ATOM	1907	2HG2	ILE	125	-13.538	-2.522	6.871	1.00	0.00	H
ATOM	1908	3HG2	ILE	125	-12.059	-3.086	6.095	1.00	0.00	H
ATOM	1909	2HG3	ILE	125	-15.001	-0.773	5.527	1.00	0.00	H
ATOM	1910	1HD1	ILE	125	-12.309	0.291	5.521	1.00	0.00	H
ATOM	1911	2HD1	ILE	125	-13.484	1.412	4.832	1.00	0.00	H
ATOM	1912	3HD1	ILE	125	-13.718	0.776	6.461	1.00	0.00	H
ATOM	1913	HG2	ILE	125	-14.449	-0.330	3.898	1.00	0.00	H
ATOM	1914	N	ARG	126	-11.768	-4.136	3.336	1.00	0.00	N
ATOM	1915	CA	ARG	126	-10.998	-5.374	3.362	1.00	0.00	C

ATOM	1916	C	ARG	126	-9.694	-5.220	2.584	1.00	0.00	C
ATOM	1917	O	ARG	126	-8.842	-6.107	2.603	1.00	0.00	O
ATOM	1918	CB	ARG	126	-11.823	-6.522	2.774	1.00	0.00	C
ATOM	1919	CG	ARG	126	-11.947	-6.469	1.261	1.00	0.00	C
ATOM	1920	CD	ARG	126	-12.214	-7.847	0.676	1.00	0.00	C
ATOM	1921	NE	ARG	126	-11.065	-8.737	0.825	1.00	0.00	N
ATOM	1922	CZ	ARG	126	-11.140	-10.059	0.725	1.00	0.00	C
ATOM	1923	NH1	ARG	126	-12.304	-10.642	0.473	1.00	0.00	N
ATOM	1924	NH2	ARG	126	-10.050	-10.801	0.873	1.00	0.00	N
ATOM	1925	H	ARG	126	-12.650	-4.129	2.913	1.00	0.00	H
ATOM	1926	HA	ARG	126	-10.766	-5.600	4.391	1.00	0.00	H
ATOM	1927	2HB3	ARG	126	-12.814	-6.489	3.196	1.00	0.00	H
ATOM	1928	2HG3	ARG	126	-11.027	-6.083	0.846	1.00	0.00	H
ATOM	1929	2HD3	ARG	126	-12.439	-7.740	-0.375	1.00	0.00	H
ATOM	1930	HE	ARG	126	-10.193	-8.326	1.010	1.00	0.00	H
ATOM	1931	1HH1	ARG	126	-13.127	-10.086	0.360	1.00	0.00	H
ATOM	1932	2HH1	ARG	126	-12.360	-11.639	0.397	1.00	0.00	H
ATOM	1933	1HH2	ARG	126	-9.171	-10.365	1.062	1.00	0.00	H
ATOM	1934	2HH2	ARG	126	-10.109	-11.797	0.796	1.00	0.00	H
ATOM	1935	HB2	ARG	126	-11.341	-7.471	3.006	1.00	0.00	H
ATOM	1936	HG2	ARG	126	-12.780	-5.821	0.986	1.00	0.00	H
ATOM	1937	HD2	ARG	126	-13.052	-8.309	1.197	1.00	0.00	H
ATOM	1938	N	GLU	127	-9.548	-4.089	1.902	1.00	0.00	N
ATOM	1939	CA	GLU	127	-8.351	-3.820	1.116	1.00	0.00	C
ATOM	1940	C	GLU	127	-7.268	-3.178	1.981	1.00	0.00	C
ATOM	1941	O	GLU	127	-6.140	-3.667	2.043	1.00	0.00	O
ATOM	1942	CB	GLU	127	-8.680	-2.909	-0.067	1.00	0.00	C
ATOM	1943	CG	GLU	127	-7.684	-3.010	-1.210	1.00	0.00	C
ATOM	1944	CD	GLU	127	-7.642	-1.754	-2.058	1.00	0.00	C
ATOM	1945	OE1	GLU	127	-8.683	-1.405	-2.654	1.00	0.00	O
ATOM	1946	OE2	GLU	127	-6.570	-1.119	-2.128	1.00	0.00	O
ATOM	1947	H	GLU	127	-10.264	-3.420	1.926	1.00	0.00	H
ATOM	1948	HA	GLU	127	-7.981	-4.763	0.742	1.00	0.00	H
ATOM	1949	2HB3	GLU	127	-8.700	-1.886	0.275	1.00	0.00	H
ATOM	1950	2HG3	GLU	127	-7.961	-3.842	-1.841	1.00	0.00	H
ATOM	1951	HB2	GLU	127	-9.650	-3.189	-0.482	1.00	0.00	H
ATOM	1952	HG2	GLU	127	-6.684	-3.159	-0.808	1.00	0.00	H
ATOM	1953	N	ALA	128	-7.619	-2.079	2.638	1.00	0.00	N
ATOM	1954	CA	ALA	128	-6.681	-1.370	3.500	1.00	0.00	C
ATOM	1955	C	ALA	128	-6.681	-1.951	4.909	1.00	0.00	C
ATOM	1956	O	ALA	128	-5.629	-2.094	5.532	1.00	0.00	O
ATOM	1957	CB	ALA	128	-7.017	0.112	3.536	1.00	0.00	C
ATOM	1958	H	ALA	128	-8.534	-1.739	2.548	1.00	0.00	H
ATOM	1959	HA	ALA	128	-5.693	-1.481	3.077	1.00	0.00	H
ATOM	1960	1HB	ALA	128	-7.763	0.333	2.786	1.00	0.00	H
ATOM	1961	2HB	ALA	128	-7.402	0.369	4.513	1.00	0.00	H
ATOM	1962	3HB	ALA	128	-6.124	0.689	3.340	1.00	0.00	H
ATOM	1963	N	ASP	129	-7.867	-2.284	5.408	1.00	0.00	N
ATOM	1964	CA	ASP	129	-8.002	-2.852	6.743	1.00	0.00	C
ATOM	1965	C	ASP	129	-7.144	-4.103	6.895	1.00	0.00	C
ATOM	1966	O	ASP	129	-7.445	-5.147	6.313	1.00	0.00	O
ATOM	1967	CB	ASP	129	-9.469	-3.184	7.031	1.00	0.00	C
ATOM	1968	CG	ASP	129	-9.774	-3.204	8.517	1.00	0.00	C
ATOM	1969	OD1	ASP	129	-8.821	-3.305	9.317	1.00	0.00	O
ATOM	1970	OD2	ASP	129	-10.968	-3.125	8.877	1.00	0.00	O
ATOM	1971	H	ASP	129	-8.668	-2.148	4.862	1.00	0.00	H
ATOM	1972	HA	ASP	129	-7.664	-2.111	7.454	1.00	0.00	H
ATOM	1973	2HB3	ASP	129	-9.698	-4.157	6.622	1.00	0.00	H
ATOM	1974	HB2	ASP	129	-10.110	-2.427	6.579	1.00	0.00	H
ATOM	1975	N	ILE	130	-6.076	-3.992	7.676	1.00	0.00	N
ATOM	1976	CA	ILE	130	-5.175	-5.115	7.902	1.00	0.00	C
ATOM	1977	C	ILE	130	-5.687	-6.014	9.021	1.00	0.00	C
ATOM	1978	O	ILE	130	-5.989	-7.188	8.799	1.00	0.00	O
ATOM	1979	CB	ILE	130	-3.755	-4.635	8.256	1.00	0.00	C
ATOM	1980	CG2	ILE	130	-2.782	-5.804	8.240	1.00	0.00	C
ATOM	1981	CG1	ILE	130	-3.304	-3.546	7.280	1.00	0.00	C
ATOM	1982	CD1	ILE	130	-3.066	-4.053	5.874	1.00	0.00	C
ATOM	1983	H	ILE	130	-5.891	-3.134	8.112	1.00	0.00	H
ATOM	1984	HA	ILE	130	-5.122	-5.689	6.989	1.00	0.00	H
ATOM	1985	HB	ILE	130	-3.775	-4.225	9.255	1.00	0.00	H
ATOM	1986	1HG2	ILE	130	-1.777	-5.437	8.399	1.00	0.00	H
ATOM	1987	2HG2	ILE	130	-3.039	-6.496	9.027	1.00	0.00	H
ATOM	1988	3HG2	ILE	130	-2.836	-6.304	7.286	1.00	0.00	H
ATOM	1989	2HG3	ILE	130	-2.381	-3.112	7.638	1.00	0.00	H
ATOM	1990	1HD1	ILE	130	-2.031	-4.338	5.765	1.00	0.00	H
ATOM	1991	2HD1	ILE	130	-3.698	-4.908	5.688	1.00	0.00	H
ATOM	1992	3HD1	ILE	130	-3.301	-3.272	5.166	1.00	0.00	H

ATOM	1993	HG2	ILE	130	-4.075	-2.777	7.212	1.00	0.00	H
ATOM	1994	N	ASP	131	-5.786	-5.459	10.223	1.00	0.00	N
ATOM	1995	CA	ASP	131	-6.267	-6.208	11.378	1.00	0.00	C
ATOM	1996	C	ASP	131	-7.637	-6.817	11.098	1.00	0.00	C
ATOM	1997	O	ASP	131	-7.879	-7.988	11.388	1.00	0.00	O
ATOM	1998	CB	ASP	131	-6.339	-5.302	12.608	1.00	0.00	C
ATOM	1999	CG	ASP	131	-7.400	-4.230	12.476	1.00	0.00	C
ATOM	2000	OD1	ASP	131	-7.411	-3.530	11.443	1.00	0.00	O
ATOM	2001	OD2	ASP	131	-8.220	-4.089	13.409	1.00	0.00	O
ATOM	2002	H	ASP	131	-5.531	-4.517	10.336	1.00	0.00	H
ATOM	2003	HA	ASP	131	-5.564	-7.006	11.568	1.00	0.00	H
ATOM	2004	2HB3	ASP	131	-5.380	-4.822	12.748	1.00	0.00	H
ATOM	2005	HB2	ASP	131	-6.587	-5.901	13.484	1.00	0.00	H
ATOM	2006	N	GLY	132	-8.532	-6.013	10.532	1.00	0.00	N
ATOM	2007	CA	GLY	132	-9.868	-6.488	10.224	1.00	0.00	C
ATOM	2008	C	GLY	132	-10.895	-6.033	11.243	1.00	0.00	C
ATOM	2009	O	GLY	132	-11.735	-6.821	11.681	1.00	0.00	O
ATOM	2010	H	GLY	132	-8.284	-5.088	10.322	1.00	0.00	H
ATOM	2011	2HA	GLY	132	-10.154	-6.120	9.252	1.00	0.00	H
ATOM	2012	3HA	GLY	132	-9.856	-7.568	10.201	1.00	0.00	H
ATOM	2013	N	ASP	133	-10.828	-4.762	11.623	1.00	0.00	N
ATOM	2014	CA	ASP	133	-11.756	-4.204	12.596	1.00	0.00	C
ATOM	2015	C	ASP	133	-12.770	-3.289	11.916	1.00	0.00	C
ATOM	2016	O	ASP	133	-13.452	-2.503	12.573	1.00	0.00	O
ATOM	2017	CB	ASP	133	-10.997	-3.433	13.675	1.00	0.00	C
ATOM	2018	CG	ASP	133	-10.154	-2.313	13.102	1.00	0.00	C
ATOM	2019	OD1	ASP	133	-10.314	-2.004	11.902	1.00	0.00	O
ATOM	2020	OD2	ASP	133	-9.332	-1.745	13.851	1.00	0.00	O
ATOM	2021	H	ASP	133	-10.134	-4.186	11.235	1.00	0.00	H
ATOM	2022	HA	ASP	133	-12.286	-5.024	13.057	1.00	0.00	H
ATOM	2023	2HB3	ASP	133	-10.348	-4.115	14.206	1.00	0.00	H
ATOM	2024	HB2	ASP	133	-11.708	-2.982	14.368	1.00	0.00	H
ATOM	2025	N	GLY	134	-12.863	-3.393	10.594	1.00	0.00	N
ATOM	2026	CA	GLY	134	-13.792	-2.566	9.847	1.00	0.00	C
ATOM	2027	C	GLY	134	-13.413	-1.099	9.871	1.00	0.00	C
ATOM	2028	O	GLY	134	-14.280	-0.226	9.852	1.00	0.00	O
ATOM	2029	H	GLY	134	-12.294	-4.036	10.121	1.00	0.00	H
ATOM	2030	2HA	GLY	134	-13.816	-2.905	8.823	1.00	0.00	H
ATOM	2031	3HA	GLY	134	-14.778	-2.678	10.274	1.00	0.00	H
ATOM	2032	N	GLN	135	-12.112	-0.827	9.911	1.00	0.00	N
ATOM	2033	CA	GLN	135	-11.621	0.548	9.938	1.00	0.00	C
ATOM	2034	C	GLN	135	-10.379	0.697	9.066	1.00	0.00	C
ATOM	2035	O	GLN	135	-9.741	-0.288	8.700	1.00	0.00	O
ATOM	2036	CB	GLN	135	-11.303	0.967	11.375	1.00	0.00	C
ATOM	2037	CG	GLN	135	-12.395	0.610	12.370	1.00	0.00	C
ATOM	2038	CD	GLN	135	-12.474	1.582	13.527	1.00	0.00	C
ATOM	2039	OE1	GLN	135	-13.058	2.659	13.410	1.00	0.00	O
ATOM	2040	NE2	GLN	135	-11.881	1.210	14.656	1.00	0.00	N
ATOM	2041	H	GLN	135	-11.470	-1.564	9.921	1.00	0.00	H
ATOM	2042	HA	GLN	135	-12.399	1.185	9.550	1.00	0.00	H
ATOM	2043	2HB3	GLN	135	-11.162	2.037	11.400	1.00	0.00	H
ATOM	2044	2HG3	GLN	135	-12.198	-0.379	12.760	1.00	0.00	H
ATOM	2045	1HE2	GLN	135	-11.434	0.337	14.678	1.00	0.00	H
ATOM	2046	2HE2	GLN	135	-11.917	1.819	15.422	1.00	0.00	H
ATOM	2047	HB2	GLN	135	-10.401	0.460	11.712	1.00	0.00	H
ATOM	2048	HG2	GLN	135	-13.365	0.628	11.869	1.00	0.00	H
ATOM	2049	N	VAL	136	-10.043	1.939	8.734	1.00	0.00	N
ATOM	2050	CA	VAL	136	-8.877	2.221	7.906	1.00	0.00	C
ATOM	2051	C	VAL	136	-8.062	3.375	8.476	1.00	0.00	C
ATOM	2052	O	VAL	136	-8.178	4.514	8.023	1.00	0.00	O
ATOM	2053	CB	VAL	136	-9.286	2.562	6.459	1.00	0.00	C
ATOM	2054	CG1	VAL	136	-8.061	2.896	5.623	1.00	0.00	C
ATOM	2055	CG2	VAL	136	-10.066	1.409	5.841	1.00	0.00	C
ATOM	2056	H	VAL	136	-10.591	2.685	9.057	1.00	0.00	H
ATOM	2057	HA	VAL	136	-8.262	1.334	7.883	1.00	0.00	H
ATOM	2058	HB	VAL	136	-9.927	3.430	6.483	1.00	0.00	H
ATOM	2059	1HG1	VAL	136	-7.897	3.964	5.636	1.00	0.00	H
ATOM	2060	2HG1	VAL	136	-7.197	2.393	6.031	1.00	0.00	H
ATOM	2061	3HG1	VAL	136	-8.221	2.571	4.606	1.00	0.00	H
ATOM	2062	1HG2	VAL	136	-9.392	0.783	5.275	1.00	0.00	H
ATOM	2063	2HG2	VAL	136	-10.527	0.824	6.624	1.00	0.00	H
ATOM	2064	3HG2	VAL	136	-10.829	1.802	5.187	1.00	0.00	H
ATOM	2065	N	ASN	137	-7.236	3.075	9.472	1.00	0.00	N
ATOM	2066	CA	ASN	137	-6.400	4.088	10.107	1.00	0.00	C
ATOM	2067	C	ASN	137	-5.349	4.611	9.133	1.00	0.00	C
ATOM	2068	O	ASN	137	-5.272	4.167	7.988	1.00	0.00	O
ATOM	2069	CB	ASN	137	-5.720	3.512	11.350	1.00	0.00	C

ATOM	2070	CG	ASN	137	-5.093	2.158	11.089	1.00	0.00	C
ATOM	2071	OD1	ASN	137	-5.758	1.124	11.176	1.00	0.00	O
ATOM	2072	ND2	ASN	137	-3.803	2.154	10.771	1.00	0.00	N
ATOM	2073	H	ASN	137	-7.188	2.148	9.790	1.00	0.00	H
ATOM	2074	HA	ASN	137	-7.042	4.905	10.402	1.00	0.00	H
ATOM	2075	2HB3	ASN	137	-6.452	3.407	12.137	1.00	0.00	H
ATOM	2076	1HD2	ASN	137	-3.337	3.014	10.721	1.00	0.00	H
ATOM	2077	2HD2	ASN	137	-3.372	1.290	10.599	1.00	0.00	H
ATOM	2078	HB2	ASN	137	-4.924	4.181	11.671	1.00	0.00	H
ATOM	2079	N	TYR	138	-4.540	5.558	9.598	1.00	0.00	N
ATOM	2080	CA	TYR	138	-3.494	6.144	8.769	1.00	0.00	C
ATOM	2081	C	TYR	138	-2.619	5.057	8.149	1.00	0.00	C
ATOM	2082	O	TYR	138	-2.490	4.974	6.927	1.00	0.00	O
ATOM	2083	CB	TYR	138	-2.632	7.097	9.597	1.00	0.00	C
ATOM	2084	CG	TYR	138	-1.545	7.779	8.798	1.00	0.00	C
ATOM	2085	CD1	TYR	138	-1.857	8.598	7.719	1.00	0.00	C
ATOM	2086	CE1	TYR	138	-0.866	9.223	6.987	1.00	0.00	C
ATOM	2087	CZ	TYR	138	0.455	9.035	7.327	1.00	0.00	C
ATOM	2088	CE2	TYR	138	0.792	8.226	8.393	1.00	0.00	C
ATOM	2089	CD2	TYR	138	-0.205	7.604	9.121	1.00	0.00	C
ATOM	2090	OH	TYR	138	1.447	9.654	6.600	1.00	0.00	O
ATOM	2091	H	TYR	138	-4.652	5.871	10.519	1.00	0.00	H
ATOM	2092	HA	TYR	138	-3.972	6.703	7.976	1.00	0.00	H
ATOM	2093	2HB3	TYR	138	-2.160	6.542	10.395	1.00	0.00	H
ATOM	2094	1HD	TYR	138	-2.895	8.745	7.456	1.00	0.00	H
ATOM	2095	1HE	TYR	138	-1.129	9.856	6.153	1.00	0.00	H
ATOM	2096	2HE	TYR	138	1.828	8.078	8.660	1.00	0.00	H
ATOM	2097	2HD	TYR	138	0.056	6.970	9.956	1.00	0.00	H
ATOM	2098	HH	TYR	138	1.821	9.031	5.973	1.00	0.00	H
ATOM	2099	HB2	TYR	138	-3.258	7.888	10.011	1.00	0.00	H
ATOM	2100	N	GLU	139	-2.021	4.230	8.999	1.00	0.00	N
ATOM	2101	CA	GLU	139	-1.159	3.150	8.534	1.00	0.00	C
ATOM	2102	C	GLU	139	-1.870	2.301	7.482	1.00	0.00	C
ATOM	2103	O	GLU	139	-1.322	2.036	6.413	1.00	0.00	O
ATOM	2104	CB	GLU	139	-0.729	2.270	9.710	1.00	0.00	C
ATOM	2105	CG	GLU	139	0.166	1.110	9.306	1.00	0.00	C
ATOM	2106	CD	GLU	139	0.004	-0.094	10.214	1.00	0.00	C
ATOM	2107	OE1	GLU	139	-1.097	-0.274	10.773	1.00	0.00	O
ATOM	2108	OE2	GLU	139	0.981	-0.857	10.364	1.00	0.00	O
ATOM	2109	H	GLU	139	-2.165	4.348	9.961	1.00	0.00	H
ATOM	2110	HA	GLU	139	-0.283	3.594	8.091	1.00	0.00	H
ATOM	2111	2HB3	GLU	139	-1.612	1.867	10.185	1.00	0.00	H
ATOM	2112	2HG3	GLU	139	1.194	1.436	9.345	1.00	0.00	H
ATOM	2113	HB2	GLU	139	-0.159	2.867	10.422	1.00	0.00	H
ATOM	2114	HG2	GLU	139	-0.090	0.789	8.297	1.00	0.00	H
ATOM	2115	N	GLU	140	-3.090	1.878	7.797	1.00	0.00	N
ATOM	2116	CA	GLU	140	-3.873	1.059	6.880	1.00	0.00	C
ATOM	2117	C	GLU	140	-4.147	1.809	5.579	1.00	0.00	C
ATOM	2118	O	GLU	140	-4.154	1.219	4.498	1.00	0.00	O
ATOM	2119	CB	GLU	140	-5.193	0.645	7.531	1.00	0.00	C
ATOM	2120	CG	GLU	140	-5.023	-0.297	8.711	1.00	0.00	C
ATOM	2121	CD	GLU	140	-6.340	-0.641	9.381	1.00	0.00	C
ATOM	2122	OE1	GLU	140	-7.206	0.252	9.479	1.00	0.00	O
ATOM	2123	OE2	GLU	140	-6.502	-1.805	9.802	1.00	0.00	O
ATOM	2124	H	GLU	140	-3.472	2.122	8.666	1.00	0.00	H
ATOM	2125	HA	GLU	140	-3.299	0.172	6.655	1.00	0.00	H
ATOM	2126	2HB3	GLU	140	-5.806	0.152	6.791	1.00	0.00	H
ATOM	2127	2HG3	GLU	140	-4.379	0.174	9.439	1.00	0.00	H
ATOM	2128	HB2	GLU	140	-5.702	1.529	7.916	1.00	0.00	H
ATOM	2129	HG2	GLU	140	-4.581	-1.233	8.369	1.00	0.00	H
ATOM	2130	N	PHE	141	-4.373	3.113	5.691	1.00	0.00	N
ATOM	2131	CA	PHE	141	-4.649	3.943	4.524	1.00	0.00	C
ATOM	2132	C	PHE	141	-3.414	4.069	3.641	1.00	0.00	C
ATOM	2133	O	PHE	141	-3.490	3.909	2.423	1.00	0.00	O
ATOM	2134	CB	PHE	141	-5.119	5.332	4.963	1.00	0.00	C
ATOM	2135	CG	PHE	141	-5.008	6.371	3.883	1.00	0.00	C
ATOM	2136	CD1	PHE	141	-5.648	6.195	2.666	1.00	0.00	C
ATOM	2137	CE1	PHE	141	-5.545	7.149	1.672	1.00	0.00	C
ATOM	2138	CZ	PHE	141	-4.803	8.293	1.886	1.00	0.00	C
ATOM	2139	CE2	PHE	141	-4.159	8.480	3.094	1.00	0.00	C
ATOM	2140	CD2	PHE	141	-4.265	7.523	4.086	1.00	0.00	C
ATOM	2141	H	PHE	141	-4.354	3.527	6.580	1.00	0.00	H
ATOM	2142	HA	PHE	141	-5.435	3.469	3.961	1.00	0.00	H
ATOM	2143	2HB3	PHE	141	-4.524	5.659	5.802	1.00	0.00	H
ATOM	2144	1HD	PHE	141	-6.232	5.303	2.498	1.00	0.00	H
ATOM	2145	1HE	PHE	141	-6.050	7.001	0.729	1.00	0.00	H
ATOM	2146	HZ	PHE	141	-4.721	9.038	1.110	1.00	0.00	H

ATOM	2147	2HE	PHE	141	-3.576	9.374	3.265	1.00	0.00	H
ATOM	2148	2HD	PHE	141	-3.761	7.669	5.032	1.00	0.00	H
ATOM	2149	HB2	PHE	141	-6.171	5.289	5.247	1.00	0.00	H
ATOM	2150	N	VAL	142	-2.274	4.353	4.263	1.00	0.00	N
ATOM	2151	CA	VAL	142	-1.020	4.500	3.533	1.00	0.00	C
ATOM	2152	C	VAL	142	-0.742	3.276	2.668	1.00	0.00	C
ATOM	2153	O	VAL	142	-0.453	3.398	1.478	1.00	0.00	O
ATOM	2154	CB	VAL	142	0.166	4.718	4.493	1.00	0.00	C
ATOM	2155	CG1	VAL	142	1.482	4.687	3.729	1.00	0.00	C
ATOM	2156	CG2	VAL	142	0.008	6.030	5.247	1.00	0.00	C
ATOM	2157	H	VAL	142	-2.276	4.469	5.236	1.00	0.00	H
ATOM	2158	HA	VAL	142	-1.103	5.368	2.896	1.00	0.00	H
ATOM	2159	HB	VAL	142	0.173	3.911	5.210	1.00	0.00	H
ATOM	2160	1HG1	VAL	142	1.604	3.721	3.265	1.00	0.00	H
ATOM	2161	2HG1	VAL	142	1.477	5.457	2.973	1.00	0.00	H
ATOM	2162	3HG1	VAL	142	2.298	4.861	4.415	1.00	0.00	H
ATOM	2163	1HG2	VAL	142	0.484	6.823	4.692	1.00	0.00	H
ATOM	2164	2HG2	VAL	142	-1.043	6.253	5.363	1.00	0.00	H
ATOM	2165	3HG2	VAL	142	0.466	5.943	6.221	1.00	0.00	H
ATOM	2166	N	GLN	143	-0.833	2.098	3.274	1.00	0.00	N
ATOM	2167	CA	GLN	143	-0.592	0.850	2.558	1.00	0.00	C
ATOM	2168	C	GLN	143	-1.449	0.768	1.301	1.00	0.00	C
ATOM	2169	O	GLN	143	-0.945	0.508	0.209	1.00	0.00	O
ATOM	2170	CB	GLN	143	-0.881	-0.348	3.466	1.00	0.00	C
ATOM	2171	CG	GLN	143	0.016	-0.412	4.691	1.00	0.00	C
ATOM	2172	CD	GLN	143	1.482	-0.567	4.335	1.00	0.00	C
ATOM	2173	OE1	GLN	143	2.103	0.355	3.805	1.00	0.00	O
ATOM	2174	NE2	GLN	143	2.043	-1.733	4.625	1.00	0.00	N
ATOM	2175	H	GLN	143	-1.067	2.065	4.226	1.00	0.00	H
ATOM	2176	HA	GLN	143	0.449	0.830	2.272	1.00	0.00	H
ATOM	2177	2HB3	GLN	143	-0.743	-1.256	2.898	1.00	0.00	H
ATOM	2178	2HG3	GLN	143	-0.282	-1.257	5.295	1.00	0.00	H
ATOM	2179	1HE2	GLN	143	1.486	-2.423	5.046	1.00	0.00	H
ATOM	2180	2HE2	GLN	143	2.990	-1.860	4.404	1.00	0.00	H
ATOM	2181	HB2	GLN	143	-1.904	-0.285	3.836	1.00	0.00	H
ATOM	2182	HG2	GLN	143	-0.080	0.511	5.261	1.00	0.00	H
ATOM	2183	N	MET	144	-2.751	0.994	1.461	1.00	0.00	N
ATOM	2184	CA	MET	144	-3.678	0.947	0.337	1.00	0.00	C
ATOM	2185	C	MET	144	-3.170	1.796	-0.824	1.00	0.00	C
ATOM	2186	O	MET	144	-3.087	1.328	-1.958	1.00	0.00	O
ATOM	2187	CB	MET	144	-5.063	1.432	0.771	1.00	0.00	C
ATOM	2188	CG	MET	144	-6.022	1.647	-0.388	1.00	0.00	C
ATOM	2189	SD	MET	144	-7.675	2.119	0.157	1.00	0.00	S
ATOM	2190	CE	MET	144	-8.516	2.284	-1.415	1.00	0.00	C
ATOM	2191	H	MET	144	-3.092	1.196	2.357	1.00	0.00	H
ATOM	2192	HA	MET	144	-3.752	-0.078	0.014	1.00	0.00	H
ATOM	2193	2HB3	MET	144	-4.954	2.369	1.299	1.00	0.00	H
ATOM	2194	2HG3	MET	144	-6.093	0.730	-0.952	1.00	0.00	H
ATOM	2195	1HE	MET	144	-8.945	3.273	-1.492	1.00	0.00	H
ATOM	2196	2HE	MET	144	-7.809	2.134	-2.217	1.00	0.00	H
ATOM	2197	3HE	MET	144	-9.301	1.547	-1.482	1.00	0.00	H
ATOM	2198	HB2	MET	144	-5.524	0.685	1.416	1.00	0.00	H
ATOM	2199	HG2	MET	144	-5.653	2.454	-1.019	1.00	0.00	H
ATOM	2200	N	MET	145	-2.827	3.048	-0.531	1.00	0.00	N
ATOM	2201	CA	MET	145	-2.327	3.960	-1.551	1.00	0.00	C
ATOM	2202	C	MET	145	-1.139	3.350	-2.290	1.00	0.00	C
ATOM	2203	O	MET	145	-1.031	3.459	-3.513	1.00	0.00	O
ATOM	2204	CB	MET	145	-1.920	5.291	-0.918	1.00	0.00	C
ATOM	2205	CG	MET	145	-3.072	6.028	-0.253	1.00	0.00	C
ATOM	2206	SD	MET	145	-4.402	6.420	-1.407	1.00	0.00	S
ATOM	2207	CE	MET	145	-4.867	4.780	-1.956	1.00	0.00	C
ATOM	2208	H	MET	145	-2.914	3.363	0.393	1.00	0.00	H
ATOM	2209	HA	MET	145	-3.123	4.136	-2.257	1.00	0.00	H
ATOM	2210	2HB3	MET	145	-1.508	5.931	-1.685	1.00	0.00	H
ATOM	2211	2HG3	MET	145	-2.697	6.947	0.169	1.00	0.00	H
ATOM	2212	1HE	MET	145	-4.038	4.327	-2.480	1.00	0.00	H
ATOM	2213	2HE	MET	145	-5.129	4.176	-1.102	1.00	0.00	H
ATOM	2214	3HE	MET	145	-5.716	4.853	-2.622	1.00	0.00	H
ATOM	2215	HB2	MET	145	-1.176	5.112	-0.139	1.00	0.00	H
ATOM	2216	HG2	MET	145	-3.505	5.400	0.525	1.00	0.00	H
ATOM	2217	N	THR	146	-0.247	2.710	-1.541	1.00	0.00	N
ATOM	2218	CA	THR	146	0.933	2.084	-2.125	1.00	0.00	C
ATOM	2219	C	THR	146	0.735	0.581	-2.287	1.00	0.00	C
ATOM	2220	O	THR	146	1.653	-0.205	-2.049	1.00	0.00	O
ATOM	2221	CB	THR	146	2.187	2.336	-1.264	1.00	0.00	C
ATOM	2222	OG1	THR	146	1.982	1.828	0.059	1.00	0.00	O
ATOM	2223	CG2	THR	146	2.507	3.822	-1.199	1.00	0.00	C

```
ATOM   2224  H   THR   146      -0.388   2.657  -0.572  1.00  0.00           H
ATOM   2225  HA  THR   146       1.094   2.523  -3.098  1.00  0.00           H
ATOM   2226  HB  THR   146       3.022   1.821  -1.714  1.00  0.00           H
ATOM   2227 1HG  THR   146       1.998   2.555   0.687  1.00  0.00           H
ATOM   2228 1HG2 THR   146       2.911   4.060  -0.224  1.00  0.00           H
ATOM   2229 2HG2 THR   146       1.606   4.394  -1.365  1.00  0.00           H
ATOM   2230 3HG2 THR   146       3.233   4.066  -1.959  1.00  0.00           H
ATOM   2231  N   ALA   147      -0.466   0.187  -2.693  1.00  0.00           N
ATOM   2232  CA  ALA   147      -0.784  -1.223  -2.889  1.00  0.00           C
ATOM   2233  C   ALA   147      -1.459  -1.451  -4.238  1.00  0.00           C
ATOM   2234  O   ALA   147      -2.600  -1.038  -4.450  1.00  0.00           O
ATOM   2235  CB  ALA   147      -1.670  -1.725  -1.761  1.00  0.00           C
ATOM   2236  H   ALA   147      -1.158   0.861  -2.867  1.00  0.00           H
ATOM   2237  HA  ALA   147       0.141  -1.778  -2.865  1.00  0.00           H
ATOM   2238 1HB  ALA   147      -2.544  -2.204  -2.176  1.00  0.00           H
ATOM   2239 2HB  ALA   147      -1.121  -2.440  -1.163  1.00  0.00           H
ATOM   2240 3HB  ALA   147      -1.973  -0.894  -1.142  1.00  0.00           H
ATOM   2241  N   LYS   148      -0.748  -2.108  -5.147  1.00  0.00           N
ATOM   2242  CA  LYS   148      -1.278  -2.393  -6.475  1.00  0.00           C
ATOM   2243  C   LYS   148      -1.699  -3.855  -6.590  1.00  0.00           C
ATOM   2244  O   LYS   148      -0.837  -4.724  -6.677  1.00  0.00           O
ATOM   2245  CB  LYS   148      -0.235  -2.066  -7.545  1.00  0.00           C
ATOM   2246  CG  LYS   148       0.267  -0.632  -7.487  1.00  0.00           C
ATOM   2247  CD  LYS   148      -0.830   0.357  -7.843  1.00  0.00           C
ATOM   2248  CE  LYS   148      -1.056   0.421  -9.345  1.00  0.00           C
ATOM   2249  NZ  LYS   148       0.029   1.171 -10.036  1.00  0.00           N
ATOM   2250  H   LYS   148       0.156  -2.412  -4.917  1.00  0.00           H
ATOM   2251  HA  LYS   148      -2.145  -1.767  -6.626  1.00  0.00           H
ATOM   2252 2HB3 LYS   148      -0.669  -2.235  -8.520  1.00  0.00           H
ATOM   2253 2HG3 LYS   148       1.082  -0.519  -8.186  1.00  0.00           H
ATOM   2254 2HD3 LYS   148      -0.549   1.337  -7.487  1.00  0.00           H
ATOM   2255 2HE3 LYS   148      -1.998   0.915  -9.533  1.00  0.00           H
ATOM   2256 1HZ  LYS   148       0.271   0.704 -10.934  1.00  0.00           H
ATOM   2257 2HZ  LYS   148       0.878   1.205  -9.436  1.00  0.00           H
ATOM   2258 3HZ  LYS   148      -0.280   2.144 -10.237  1.00  0.00           H
ATOM   2259  HB2 LYS   148       0.640  -2.700  -7.404  1.00  0.00           H
ATOM   2260  HG2 LYS   148       0.610  -0.408  -6.476  1.00  0.00           H
ATOM   2261  HD2 LYS   148      -1.765   0.044  -7.378  1.00  0.00           H
ATOM   2262  HE2 LYS   148      -1.074  -0.589  -9.754  1.00  0.00           H
ATOM   2263  HXT LYS   148      -2.745  -4.128  -6.596  1.00  0.00           H
TER    2264      LYS   148
HETATM 2265 CA   CA    149      26.033  -5.982  -2.894  1.00  0.00          Ca
HETATM 2266 CA   CA    150      30.426   3.339  -5.149  1.00  0.00          Ca
HETATM 2267 CA   CA    151     -12.834   7.964  11.921  1.00  0.00          Ca
HETATM 2268 CA   CA    152      -8.178  -0.981  10.612  1.00  0.00          Ca
HETATM 2269  F1  UNK     1     -10.438   7.038  -3.872  1.00  0.00           F
HETATM 2270  C1  UNK     1      -9.340   6.280  -3.838  1.00  0.00           C
HETATM 2271  C2  UNK     1      -8.460   6.620  -2.619  1.00  0.00           C
HETATM 2272  O1  UNK     1      -9.230   6.319  -1.455  1.00  0.00           O
HETATM 2273  C3  UNK     1      -8.563   6.029  -0.306  1.00  0.00           C
HETATM 2274  F2  UNK     1      -9.426   5.486   0.584  1.00  0.00           F
HETATM 2275  F3  UNK     1      -9.702   5.000  -3.777  1.00  0.00           F
HETATM 2276  F4  UNK     1      -8.658   6.455  -4.970  1.00  0.00           F
HETATM 2277  C4  UNK     1      -7.944   8.081  -2.627  1.00  0.00           C
HETATM 2278  F5  UNK     1      -7.175   8.282  -3.698  1.00  0.00           F
HETATM 2279  F6  UNK     1      -7.197   8.335  -1.554  1.00  0.00           F
HETATM 2280  F7  UNK     1      -8.951   8.952  -2.664  1.00  0.00           F
HETATM 2281  H1  UNK     1      -8.162   6.938   0.135  1.00  0.00           H
HETATM 2282  H2  UNK     1      -7.785   5.290  -0.497  1.00  0.00           H
HETATM 2283  H3  UNK     1      -7.599   5.951  -2.652  1.00  0.00           H
ENDMDL
CONECT 2270 2269 2271 2275 2276
CONECT 2271 2270 2272 2277 2283
CONECT 2273 2272 2274 2281 2282
CONECT 2277 2271 2278 2279 2280
CONECT 2269 2270
CONECT 2274 2273
CONECT 2275 2270
CONECT 2276 2270
CONECT 2278 2277
CONECT 2279 2277
CONECT 2280 2277
CONECT 2281 2273
CONECT 2282 2273
CONECT 2283 2271
CONECT 2272 2271 2273
END
```

Supplemental References:

1. Franks, N. P. & Lieb, W. R. (1994). Molecular and cellular mechanisms of general anesthesia. *Nature* **367**, 607-614.
2. Streiff, J. H., Juranic, N. O., Macura, S. I., Warner, D. O., Jones, K. A. & Perkins, W. J. (2004). Saturation transfer difference nuclear magnetic resonance spectroscopy as a method for screening proteins for anesthetic binding. *Mol. Pharmacol.* **66**, 929-935.
